# Supplementary figures and images for: High throughput detection and genetic epidemiology of SARS-CoV-2 using COVIDSeq next-generation sequencing
Source: PLoS One. 2021 Feb 17;16(2):e0247115. doi: 10.1371/journal.pone.0247115 (PMC7888613; doi:10.1371/journal.pone.0247115)

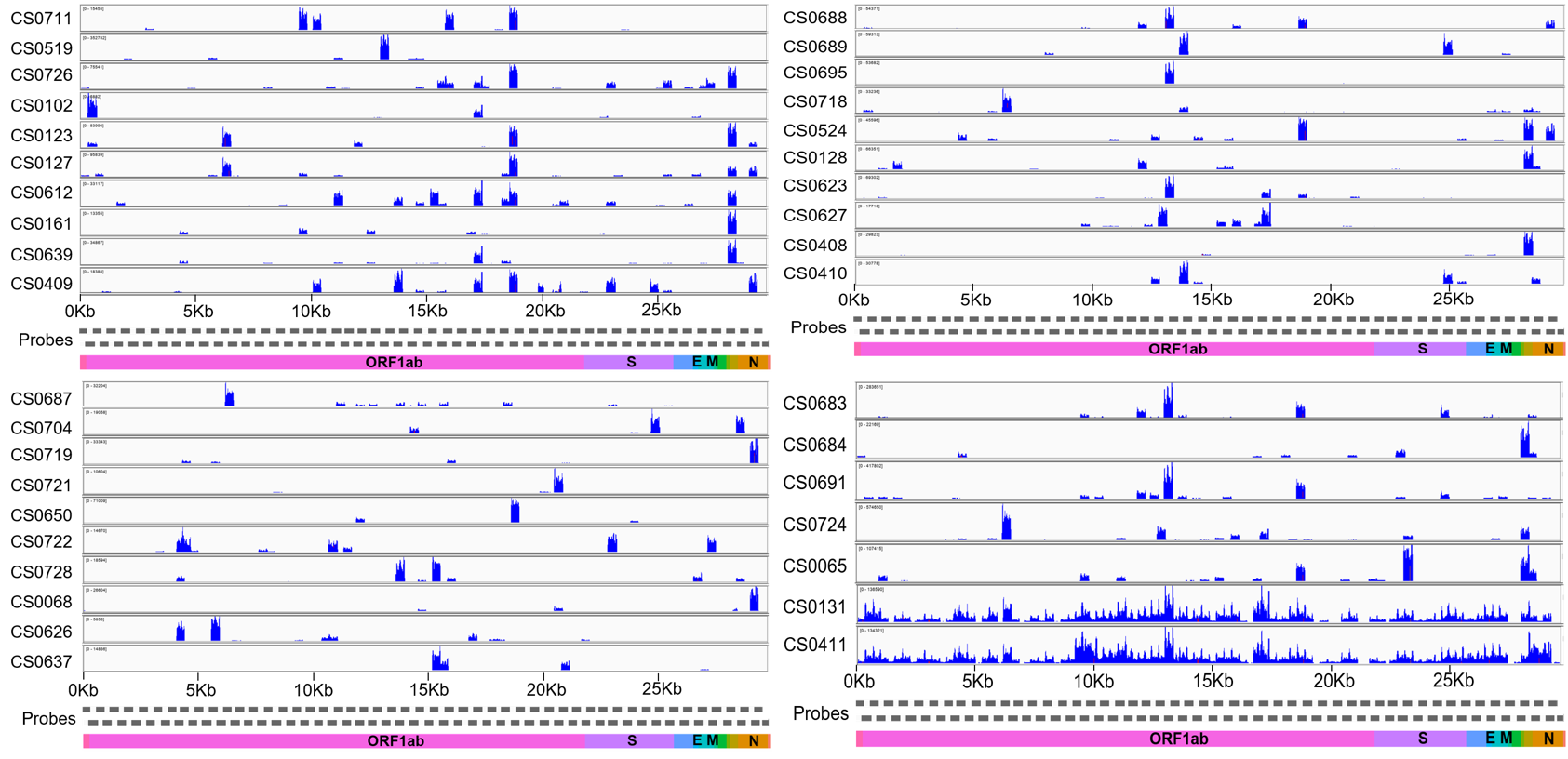

Supplement: S1 Fig — (TIF) [file pone.0247115.s001.tif]
