# Supplementary material for: High throughput detection and genetic epidemiology of SARS-CoV-2 using COVIDSeq next-generation sequencing
Source: PLoS One. 2021 Feb 17;16(2):e0247115. doi: 10.1371/journal.pone.0247115 (PMC7888613; doi:10.1371/journal.pone.0247115)
Supplement: S1 Table — (PDF) [file pone.0247115.s002.pdf]

Task_name	duration (min:sec)	Duration (seconds)
LookForFastqFolderInCovid19SeqTask	00:00.5	0.57
LookForFastqFolderInValidationTask	00:02.3	2.40
SampleSheetValidationTask	03:27.6	207.66
RunQcTask	02:40.7	160.79
ResetFPGATask	00:15.6	15.70
FindDragenLicenseInstanceLocationTask	00:05.0	5.01
LookForFastqFolderInAnalysisTask	00:05.0	5.01
FindDragenLicenseTask	00:03.9	3.98
FirstTileOnlyTask	00:03.9	3.98
SingleLaneOnlyTask	00:03.9	3.98
FastModeTask	00:03.9	3.98
DragenFastqGenerationTask	27:51.0	1671.07
DragenKmerTask	20:23.7	1223.77
CopyTargetBedTask	00:02.9	2.92
FindSampleValidityTask	05:56.9	356.94
DragenMapAlignTask	29:16.8	8956.87
DragenVariantCallingTask	26:15.1	8775.14
ConsensusFastaTask	09:37.0	577.01
ReportGateKeeperTask	00:01.3	1.30
JsonTsvReportTask	07:46.0	466.09
StartReportEngineTask	00:12.1	12.18
RenderReportTask	00:08.7	8.75
StopReportEngineTask	00:00.5	0.57
DsdmErrorSummaryTask	00:02.5	2.52
Total Time (in seconds)		22468.19
Total Time (in minutes)		374.47
Total Time (in hours)		6.24
