# Supplementary material for: High throughput detection and genetic epidemiology of SARS-CoV-2 using COVIDSeq next-generation sequencing
Source: PLoS One. 2021 Feb 17;16(2):e0247115. doi: 10.1371/journal.pone.0247115 (PMC7888613; doi:10.1371/journal.pone.0247115)
Supplement: S2 Table — NA- Not Applicable. (PDF) [file pone.0247115.s003.pdf]

Sample IDs Replicate 1	Sample IDs Replicate 2	RT-PCR: RT (Pos/Neg) replicate1	RT (Pos/Neg) replicate 2	DRAGEN COVIDSeq Test Pipeline: DRAGEN SARS-CoV-2 Detection Replicate 1	DRAGEN SARS-CoV-2 Detection Replicate 2
CS0531_1	CS0531_2	Positive	Positive	SARS-CoV-2 Detected	SARS-CoV-2 Detected
CS0530_1	CS0530_2	Positive	Positive	SARS-CoV-2 Detected	SARS-CoV-2 Detected
CS0529_1	CS0529_2	Positive	Positive	SARS-CoV-2 Detected	SARS-CoV-2 Detected
CS0533_1	CS0533_2	Positive	Positive	SARS-CoV-2 Detected	SARS-CoV-2 Detected
CS0545_1	CS0545_2	Positive	Positive	SARS-CoV-2 Detected	SARS-CoV-2 Detected
CS0532_1	CS0532_2	Positive	Positive	SARS-CoV-2 Detected	SARS-CoV-2 Detected
CS0544_1	CS0544_2	Positive	Positive	SARS-CoV-2 Detected	SARS-CoV-2 Detected
CS0543_1	CS0543_2	Positive	Positive	SARS-CoV-2 Detected	SARS-CoV-2 Detected
CS0542_1	CS0542_2	Positive	Positive	SARS-CoV-2 Detected	SARS-CoV-2 Detected
CS0541_1	CS0541_2	Positive	Positive	SARS-CoV-2 Detected	SARS-CoV-2 Detected
CS0540_1	CS0540_2	Positive	Positive	SARS-CoV-2 Detected	SARS-CoV-2 Detected
CS0539_1	CS0539_2	Positive	Positive	SARS-CoV-2 Detected	SARS-CoV-2 Detected
CS0538_1	CS0538_2	Positive	Positive	SARS-CoV-2 Detected	SARS-CoV-2 Detected
CS0537_1	CS0537_2	Positive	Positive	SARS-CoV-2 Detected	SARS-CoV-2 Detected
CS0536_1	CS0536_2	Positive	Positive	SARS-CoV-2 Detected	SARS-CoV-2 Detected
CS0535_1	CS0535_2	Positive	Positive	SARS-CoV-2 Detected	SARS-CoV-2 Detected
CS0534_1	CS0534_2	Positive	Positive	SARS-CoV-2 Detected	SARS-CoV-2 Detected
CS0551_1	CS0551_2	Positive	Positive	SARS-CoV-2 Detected	SARS-CoV-2 Detected
CS0550_1	CS0550_2	Positive	Positive	SARS-CoV-2 Detected	SARS-CoV-2 Detected
CS0549_1	CS0549_2	Positive	Positive	SARS-CoV-2 Detected	SARS-CoV-2 Detected
CS0548_1	CS0548_2	Positive	Positive	SARS-CoV-2 Detected	SARS-CoV-2 Detected
CS0547_1	CS0547_2	Positive	Positive	SARS-CoV-2 Detected	SARS-CoV-2 Detected
CS0546_1	CS0546_2	Positive	Positive	SARS-CoV-2 Detected	SARS-CoV-2 Detected
[illegible]	[illegible]	[illegible]	[illegible]	[illegible]	[illegible]

In - Depth Custom Pipeline (Merged)

Sample IDs Replicate 1	Total Reads_merged	Trimmed Reads q30-30bp_merged	Hisat2 Human % _merged	Hisat2 Covidall % _merged	Hisat2 Unmapped Covid Read#_merged	Hisat2 Unmapped Covid %_merged	BasePairescovered_merged	X coverage _merged	Genome coverage_merged	# bases with 0 coverage_merged	# bases with >0 coverage_merged	# bases with <10x coverage_merged	# bases with <100x coverage_merged	VARSCAN Variant Count_merged	N's_merged
CS0531_1	18352585	17194961	1.11	98.08	17003620	99.19	599305340	20041.64599	99.96321439	11	29892	56	88	13	57
CS0530_1	14110540	13190264	4.28	94.65	12625755	98.89	443779374	14840.63051	99.86289001	41	29862	49	241	15	206
CS0529_1	17064503	16003804	4.27	94.67	15320304	98.89	538524076	18009.03174	99.85954586	42	29861	60	259	14	234
CS0533_1	16363129	15369240	1.1	97.96	15200834	99.04	535287906	17900.80948	99.78263051	65	29838	81	251	13	311
CS0545_1	19052128	17733506	1.04	98.03	17548399	99.06	617693386	20656.56911	99.95987025	12	29891	58	78	12	67
CS0532_1	19216801	17895898	7.34	91.17	16581942	98.4	579840423	19390.71073	99.96990269	9	29894	57	99	14	259
CS0544_1	20077876	18870898	0.93	98.21	18694583	99.14	658874134	22033.71347	99.95987025	12	29891	56	198	10	317
CS0543_1	19486012	18416819	4.68	94.27	17555450	98.89	617222854	20640.83383	99.85620172	43	29860	76	238	15	300
CS0542_1	14463699	13604022	8.34	89.96	12469734	98.14	435003119	14547.13972	99.96655854	10	29893	50	115	15	332
CS0541_1	20335078	18823699	2.99	95.95	18260096	98.91	641771847	21461.78801	99.96321439	11	29892	52	82	15	60
CS0540_1	18167494	17053272	3.3	95.75	16490328	99.02	580458127	19411.36766	99.94649366	16	29887	55	133	12	262
CS0539_1	17827645	16711870	1.43	97.77	16472549	99.19	580858888	19424.76969	99.85285757	44	29859	69	90	10	258
CS0538_1	16879824	15895954	5.42	93.21	15034264	98.55	526674561	17612.76665	99.96655854	10	29893	51	76	12	129
CS0537_1	17737571	16645780	0.9	97.79	16495243	98.69	578638743	19350.5248	99.97659098	7	29896	51	251	12	312
CS0536_1	17579963	16466263	0.85	97.66	16325680	98.5	571621499	19115.85791	99.93980537	18	29885	74	240	11	319
CS0535_1	15300331	14133495	6.11	92.6	13269910	98.63	465018803	15550.90804	99.99665585	1	29902	44	436	12	295
CS0534_1	25164430	23582985	0.9	98.26	23369824	99.16	823617472	27542.97134	100	0	29903	46	85	12	68
CS0551_1	16531650	15499827	5	93.83	14725197	98.77	516972692	17288.32197	99.82944855	51	29852	80	323	15	287
CS0550_1	15491718	14475534	8.47	89.74	13250077	98.04	461733778	15441.052	99.93980537	18	29885	73	347	13	331
CS0549_1	15309478	14350099	8.03	90.77	13197442	98.7	463052057	15485.13718	99.80603953	58	29845	63	359	14	274
CS0548_1	16752053	15821204	4.22	94.48	15153999	98.64	531402859	17770.88784	99.86289001	41	29862	48	229	13	261
CS0547_1	18879927	17512705	13.67	84.91	15118632	98.35	528478130	17673.08063	99.34454737	196	29707	202	805	12	487
CS0546_1	19661605	18478539	17.55	80.72	15234837	97.9	530272507	17733.08722	99.85954586	42	29861	330	875	12	908
[illegible]	[illegible]	[illegible]	[illegible]	[illegible]	[illegible]	[illegible]	[illegible]	[illegible]	[illegible]	[illegible]	[illegible]	[illegible]	[illegible]	[illegible]	[illegible]

In - Depth Custom Pipeline (Replicate 1)

Sample IDs Replicate 1	Total Reads_Replicate1	Trimmed Reads q30-30bp_Replicate1	Kraken2 coronavirus %_Replicate1	Hisat2 Human % _Replicate1	Hisat2 Covidall % _Replicate1	Hisat2 Unmapped Covid Read#_Replicate1	Hisat2 Unmapped Covid %_Replicate1	BasePairescovered_Replicate1	X coverage _Replicate1	Genome coverage_Replicate1	# bases with 0 coverage_Replicate1	# bases with >0 coverage_Replicate1	# bases with <10x coverage_Replicate1	# bases with <100x coverage_Replicate1	VARSCAN Variant Count_Replicate1	N's_Replicate1
CS0531_1	9227700	8702460	0.54	1.12	98.08	8605386	99.19	303345230	10144.30759	99.93980537	18	29885	71	110	13	58
CS0530_1	7083108	6666395	0.54	4.3	94.63	6379569	98.88	224250778	7499.273585	99.84616928	46	29857	52	472	15	231
CS0529_1	8561478	8080890	0.56	4.29	94.65	7734194	98.89	271879245	9092.039093	99.83613684	49	29854	71	562	15	287
CS0533_1	8195160	7741017	0.63	1.1	97.94	7655629	99.03	269581080	9015.185098	99.76256563	71	29832	111	917	13	323
CS0545_1	9570945	8971591	0.59	1.05	98.03	8877643	99.06	312526290	10451.33565	99.94314952	17	29886	63	180	11	112
CS0532_1	9623488	9027824	0.5	7.39	91.11	8360737	98.38	292354092	9776.747885	99.95318195	14	29889	59	243	14	302
CS0544_1	10051772	9506119	0.58	0.94	98.2	9417024	99.13	331917289	11099.79898	99.95987025	12	29891	70	567	11	321
CS0543_1	9758391	9274190	0.55	4.7	94.24	8838312	98.89	310759485	10392.25111	99.78597465	64	29839	82	517	15	309
CS0542_1	7248976	6858700	0.52	8.4	89.87	6282236	98.12	219121755	7327.751563	99.96321439	11	29892	55	932	14	435
CS0541_1	10211538	9525628	0.54	3.01	95.94	9239217	98.91	324752808	10860.20827	99.85620172	43	29860	52	221	15	176
CS0540_1	9132247	8627809	0.55	3.32	95.74	8341717	99.02	293641172	9819.78972	99.94649366	16	29887	58	330	12	309
CS0539_1	8928587	8422471	0.56	1.44	97.77	8301302	99.19	292750428	9790.00194	99.84951343	45	29858	79	175	10	293
CS0538_1	8449838	8002331	0.57	5.47	93.15	7564696	98.54	264999422	8861.967762	99.84616928	46	29857	56	91	12	236
CS0537_1	8890929	8395117	0.57	0.9	97.79	8319425	98.68	291850025	9759.891148	99.96990269	9	29894	72	446	12	331
CS0536_1	8811763	8307149	0.58	0.86	97.65	8235646	98.49	288366492	9643.396716	99.93980537	18	29885	89	870	11	341
CS0535_1	7670372	7143534	0.53	6.15	92.56	6704270	98.62	234946158	7856.942715	99.90636391	28	29875	54	718	12	283
CS0534_1	12583680	11868828	0.56	0.91	98.25	11761142	99.15	414515586	13862.00669	99.86289001	41	29862	52	95	13	149
CS0551_1	8286688	7819397	0.56	5.02	93.8	7426489	98.76	260742208	8719.600308	99.82944855	51	29852	86	685	14	303
CS0550_1	7764394	7303538	0.56	8.51	89.69	6681686	98.04	232849458	7786.826004	99.92642879	22	29881	89	484	13	410
CS0549_1	7678627	7244057	0.56	8.08	90.71	6658487	98.69	233618391	7812.540247	99.80603953	58	29845	64	408	14	329
CS0548_1	8399372	7978578	0.54	4.24	94.45	7640508	98.63	267918001	8959.569307	99.85954586	42	29861	56	389	13	274
CS0547_1	9482750	8863335	0.56	13.74	84.83	7645762	98.34	267248116	8937.167375	99.34454737	196	29707	311	2075	12	632
CS0546_1	9843174	9303864	0.62	17.61	80.66	7665533	97.9	266805900	8922.379026	99.75253319	74	29829	425	1040	10	1233
[illegible]	[illegible]	[illegible]	[illegible]	[illegible]	[illegible]	[illegible]	[illegible]	[illegible]	[illegible]	[illegible]	[illegible]	[illegible]	[illegible]	[illegible]	[illegible]	[illegible]

In - Depth Custom Pipeline (Replicate 2)

Sample IDs Replicate 2	Total Reads_Replicate2	Trimmed Reads q30-30bp_Replicate2	Hisat2 Human % _Replicate2	Hisat2 Covidall % _Replicate2	Hisat2 Unmapped Covid Read#_Replicate2	Hisat2 Unmapped Covid %_Replicate2	BasePairescovered_Replicate2	X coverage _Replicate2	Genome coverage_Replicate2	# bases with 0 coverage_Replicate2	# bases with >0 coverage_Replicate2	# bases with <10x coverage_Replicate2	# bases with <100x coverage_Replicate2	VARSCAN Variant Count_Replicate2	N's_Replicate2
CS0531_2	9124885	8492501	1.11	98.08	8398317	99.18	295960050	9897.336388	99.94649366	16	29887	70	134	13	116
CS0530_2	7027432	6523869	4.25	94.68	6246688	98.88	219528086	7341.339866	99.86289001	41	29862	52	494	14	271
CS0529_2	8503025	7922914	4.24	94.69	7587053	98.88	266644678	8916.987526	99.85954586	42	29861	64	553	15	281
CS0533_2	8167969	7628223	1.09	97.97	7545317	99.05	265706286	8885.606327	99.78263051	65	29838	86	400	14	427
CS0545_2	9481183	8761915	1.04	98.03	8670766	99.06	305167094	10205.23339	99.83948099	48	29855	64	167	12	79
CS0532_2	9593313	8868074	7.29	91.23	8221760	98.4	287485997	9613.951677	99.9565261	13	29890	59	264	12	315
CS0544_2	10026104	9364779	0.93	98.22	9277565	99.14	326956731	10933.91068	99.83948099	48	29855	78	546	11	294
CS0543_2	9727621	9142629	4.65	94.29	8717514	98.89	306463155	10248.57556	99.83948099	48	29855	76	498	15	302
CS0542_2	7214723	6745322	8.26	90.04	6187897	98.16	215881175	7219.381835	99.85285757	44	29859	54	303	15	374
CS0541_2	10123540	9298071	2.98	95.97	9021154	98.91	317018877	10601.57432	99.95318195	14	29889	63	186	15	225
CS0540_2	9035247	8425463	3.28	95.77	8148805	99.02	286816010	9591.546333	99.82944855	51	29852	59	574	12	264
CS0539_2	8899058	8289399	1.42	97.78	8171425	99.19	288108101	9634.755744	99.81272782	56	29847	77	233	10	293
CS0538_2	8429986	7893623	5.37	93.26	7469614	98.55	261674350	8750.772498	99.96321439	11	29892	53	119	12	209
CS0537_2	8846642	8250663	0.91	97.79	8175800	98.69	286788422	9590.62375	99.83948099	48	29855	60	481	12	268
CS0536_2	8768200	8159114	0.84	97.67	8090175	98.51	283254782	9472.453667	99.92308464	23	29880	80	440	11	349
CS0535_2	7629959	6989961	6.07	92.65	6565956	98.63	230072232	7693.95151	99.99331171	2	29901	51	693	11	311
CS0534_2	12580750	11714157	0.9	98.27	11608682	99.16	409101607	13680.95532	100	0	29903	61	88	13	214
CS0551_2	8244962	7680430	4.96	93.86	7299097	98.77	256230474	8568.721332	99.7893188	63	29840	93	608	15	304
CS0550_2	7727324	7171996	8.42	89.8	6568399	98.05	228884346	7654.226867	99.82944855	51	29852	115	1388	13	371
CS0549_2	7630851	7106042	7.97	90.83	6540027	98.7	229433427	7672.588938	99.80603953	58	29845	116	938	14	322
CS0548_2	8352681	7842626	4.17	94.51	7515251	98.63	263483953	8811.288265	99.86289001	41	29862	51	906	13	271
CS0547_2	9397177	8649370	13.6	84.99	7473324	98.36	261229991	8735.912484	99.34454737	196	29707	280	1776	12	668
CS0546_2	9818431	9174675	17.5	80.78	7569082	97.92	263466548	8810.706217	99.67227368	98	29805	479	4370	10	1393
[illegible]	[illegible]	[illegible]	[illegible]	[illegible]	[illegible]	[illegible]	[illegible]	[illegible]	[illegible]	[illegible]	[illegible]	[illegible]	[illegible]	[illegible]	[illegible]

Sample IDs Replicate 1	Sample IDs Replicate 2	RT-PCR: RT (Pos/Neg) replicate1	RT (Pos/Neg) replicate 2	DRAGEN COVIDSeq Test Pipeline: DRAGEN SARS-CoV-2 Detection Replicate 1	DRAGEN SARS-CoV-2 Detection Replicate 2
CS0036_1	CS0036_2	Positive	Positive	SARS-CoV-2 Detected	SARS-CoV-2 Detected
CS0279_1	CS0279_2	Positive	Positive	SARS-CoV-2 Detected	SARS-CoV-2 Detected
CS0280_1	CS0280_2	Positive	Positive	SARS-CoV-2 Detected	SARS-CoV-2 Detected
CS0724_1	CS0724_2	Inconclusive	Inconclusive	SARS-CoV-2 Detected	SARS-CoV-2 Detected
CS0281_1	CS0281_2	Positive	Positive	SARS-CoV-2 Detected	SARS-CoV-2 Detected
CS0725_1	CS0725_2	Inconclusive	Inconclusive	SARS-CoV-2 Not Detected	SARS-CoV-2 Not Detected
CS0726_1	CS0726_2	Inconclusive	Inconclusive	SARS-CoV-2 Detected	SARS-CoV-2 Detected
CS0282_1	CS0282_2	Positive	Positive	SARS-CoV-2 Detected	SARS-CoV-2 Detected
CS0523_1	CS0523_2	Pan Sarbeco	Pan Sarbeco	SARS-CoV-2 Not Detected	SARS-CoV-2 Not Detected
CS0037_1	CS0037_2	Positive	Positive	SARS-CoV-2 Detected	SARS-CoV-2 Detected
CS0038_1	CS0038_2	Positive	Positive	SARS-CoV-2 Detected	SARS-CoV-2 Detected
CS0039_1	CS0039_2	Positive	Positive	SARS-CoV-2 Detected	SARS-CoV-2 Detected
CS0286_1	CS0286_2	Positive	Positive	SARS-CoV-2 Detected	SARS-CoV-2 Detected
CS0727_1	CS0727_2	Positive	Positive	SARS-CoV-2 Detected	SARS-CoV-2 Detected
CS0287_1	CS0287_2	Positive	Positive	SARS-CoV-2 Detected	SARS-CoV-2 Detected
CS0040_1	CS0040_2	Positive	Positive	SARS-CoV-2 Detected	SARS-CoV-2 Detected
CS0288_1	CS0288_2	Positive	Positive	SARS-CoV-2 Detected	SARS-CoV-2 Detected
[illegible]	[illegible]	[illegible]	[illegible]	[illegible]	[illegible]

In - Depth Custom Pipeline (Merged)

Sample IDs Replicate 1	Total Reads_merged	Trimmed Reads q30-30bp_merged	Hisat2 Human % _merged	Hisat2 Covidall % _merged	Hisat2 Unmapped Covid Read#_merged	Hisat2 Unmapped Covid %_merged	BasePairescovered_merged	X coverage _merged	Genome coverage_merged	# bases with 0 coverage_merged	# bases with >0 coverage_merged	# bases with <10x coverage_merged	# bases with <100x coverage_merged	VARSCAN Variant Count_merged	N's_merged
CS0036_1	19610113	18531332	0.01	99.28	18529153	99.3	654195859	21877.26512	99.77594221	67	29836	82	479	13	292
CS0279_1	22713385	21446803	1.93	97.07	21033550	98.97	740178330	24752.64455	99.98996756	3	29900	50	350	14	258
CS0280_1	11836612	11170908	69.51	25.87	3405751	84.85	102828343	3438.729994	87.84068488	3636	26267	23840	25585	8	22152
CS0724_1	17673223	16508247	37.14	59.44	10377623	94.56	349034127	11672.21105	96.42845199	1068	28835	14113	16243	8	14153
CS0281_1	17976729	16954611	0.19	98.31	16922135	98.5	592600727	19817.43394	99.8327927	50	29853	71	275	14	291
CS0725_1	23651483	22319401	94.91	0.08	1135164	1.53	618761	20.69227168	5.654951008	28212	1691	29086	29131	0	29103
CS0726_1	17094792	16128502	73.81	14.56	4224636	55.59	83579185	2795.010032	37.63836404	18648	11255	19489	20806	8	19491
CS0282_1	17313673	16090024	1.86	96.84	15791248	98.67	553834724	18521.04217	99.87292245	38	29865	109	830	12	352
CS0523_1	6890935	6443455	98.3	0.53	109574	31.45	1216695	40.68805805	7.370497943	27699	2204	29463	29497	1	29457
CS0037_1	22511342	21031966	0.08	35.31	21015731	35.33	263933228	8826.312678	99.84616928	46	29857	198	1613	6	547
CS0038_1	17844959	16720627	2.44	96.52	16312285	98.94	573768505	19187.65692	99.73915661	78	29825	269	1046	11	556
CS0039_1	15467905	14592754	0.77	98.29	14479740	99.06	509885087	17051.30211	99.86289001	41	29862	50	551	12	82
CS0286_1	15286579	14188010	90.69	1.58	1320671	16.96	7956276	266.0694914	32.77597565	20102	9801	22395	24561	7	22030
CS0727_1	19224302	18104882	89.47	4.04	1906491	38.33	26005097	869.6484299	19.69367622	24014	5889	25336	26235	2	25307
CS0287_1	15101207	14243393	5.84	93.17	13412076	98.94	471744791	15775.8349	99.85285757	44	29859	58	88	11	94
CS0040_1	10203051	9651882	59.65	31.97	3894268	79.24	109812460	3672.289068	45.34327659	16344	13559	17691	18946	6	17352
CS0288_1	15855572	14952756	5.29	93.61	14162260	98.84	497649790	16642.13591	99.80603953	58	29845	74	90	16	217
[illegible]	[illegible]	[illegible]	[illegible]	[illegible]	[illegible]	[illegible]	[illegible]	[illegible]	[illegible]	[illegible]	[illegible]	[illegible]	[illegible]	[illegible]	[illegible]

In - Depth Custom Pipeline (Replicate 1)

Sample IDs Replicate 1	Total Reads_Replicate1	Trimmed Reads q30-30bp_Replicate1	Kraken2 coronavirus %_Replicate1	Hisat2 Human % _Replicate1	Hisat2 Covidall % _Replicate1	Hisat2 Unmapped Covid Read#_Replicate1	Hisat2 Unmapped Covid %_Replicate1	BasePairescovered_Replicate1	X coverage _Replicate1	Genome coverage_Replicate1	# bases with 0 coverage_Replicate1	# bases with >0 coverage_Replicate1	# bases with <10x coverage_Replicate1	# bases with <100x coverage_Replicate1	VARSCAN Variant Count_Replicate1	N's_Replicate1
CS0036_1	9827918	9323575	0.63	0.01	99.29	9322467	99.31	329206234	11009.13734	99.77594221	67	29836	89	641	13	312
CS0279_1	11363497	10775703	0.65	1.93	97.07	10567579	98.98	371944247	12438.35893	99.9565261	13	29890	67	804	14	289
CS0280_1	5955397	5640253	0.21	69.47	25.85	1721693	84.68	51883114	1735.047119	22.5662977	23155	6748	24270	25838	7	22431
CS0724_1	8876822	8345614	0.59	37.14	59.34	5246255	94.39	176161522	5891.098619	55.61983747	13271	16632	15111	17566	7	14038
CS0281_1	9014238	8538114	0.65	0.19	98.33	8521825	98.51	298501911	9982.339932	99.8327927	50	29853	73	398	14	291
CS0725_1	11895106	11284772	0	94.88	0.08	577264	1.51	309130	10.33775875	4.093234792	28679	1224	29113	29297	0	29112
CS0726_1	8589161	8148078	0.1	73.8	14.49	2134407	55.32	42030229	1405.552252	36.58830218	18962	10941	19677	21326	8	19636
CS0282_1	8664377	8094378	0.67	1.86	96.84	7944115	98.68	278667322	9319.042303	99.76256563	71	29832	235	1823	12	368
CS0523_1	3465844	3261191	0	98.3	0.54	55527	31.49	616934	20.63117413	1.625254991	29417	486	29466	29497	1	24032
CS0037_1	11314435	10626972	0.23	0.08	35.35	10618739	35.38	133553710	4466.231147	99.8261044	52	29851	673	3076	6	702
CS0038_1	8906634	8385381	0.66	2.45	96.53	8180164	98.95	287791760	9624.176838	99.69902685	90	29813	332	2186	11	912
CS0039_1	7787632	7376760	0.6	0.78	98.3	7319347	99.07	257789857	8620.869378	99.85620172	43	29860	53	817	12	218
CS0286_1	7708675	7187096	0.01	90.67	1.57	670344	16.82	4005108	133.9366619	31.27779821	20550	9353	22972	25426	7	22818
CS0727_1	9694055	9180476	0.06	89.38	4.02	975276	37.88	13148638	439.7096612	18.64027021	24329	5574	25503	26354	1	25461
CS0287_1	7584878	7182018	0.56	5.87	93.14	6760349	98.95	237817640	7952.969267	99.84616928	46	29857	69	92	13	252
CS0040_1	5128036	4867698	0.43	59.69	31.95	1962062	79.26	55342191	1850.723707	44.38350667	16631	13272	18110	19614	5	17813
CS0288_1	7938173	7518412	0.59	5.28	93.62	7121479	98.84	250278762	8369.687389	99.79935124	60	29843	85	244	16	290
[illegible]	[illegible]	[illegible]	[illegible]	[illegible]	[illegible]	[illegible]	[illegible]	[illegible]	[illegible]	[illegible]	[illegible]	[illegible]	[illegible]	[illegible]	[illegible]	[illegible]

In - Depth Custom Pipeline (Replicate 2)

Sample IDs Replicate 2	Total Reads_Replicate2	Trimmed Reads q30-30bp_Replicate2	Hisat2 Human % _Replicate2	Hisat2 Covidall % _Replicate2	Hisat2 Unmapped Covid Read#_Replicate2	Hisat2 Unmapped Covid %_Replicate2	BasePairescovered_Replicate2	X coverage _Replicate2	Genome coverage_Replicate2	# bases with 0 coverage_Replicate2	# bases with >0 coverage_Replicate2	# bases with <10x coverage_Replicate2	# bases with <100x coverage_Replicate2	VARSCAN Variant Count_Replicate2	N's_Replicate2
CS0036_2	9782195	9207757	0.01	99.27	9206686	99.29	324989637	10868.12818	99.76925392	69	29834	87	892	13	303
CS0279_2	11349888	10671100	1.92	97.06	10465971	98.97	368234083	12314.28562	99.92308464	23	29880	56	488	15	290
CS0280_2	5881215	5530655	69.48	25.89	1687837	84.83	50945229	1703.682875	87.35912785	3780	26123	24220	25891	7	21199
CS0724_2	8796401	8162633	36.46	59.55	5186238	93.73	172872605	5781.11243	96.41841956	1071	28832	14911	17667	7	14911
CS0281_2	8962491	8416497	0.19	98.29	8400310	98.48	294098825	9835.094305	99.80938367	57	29846	82	403	14	293
CS0725_2	11756377	11034629	94.94	0.08	558044	1.56	309631	10.35451293	4.534662074	28547	1356	29113	29262	0	29106
CS0726_2	8505631	7980424	73.81	14.63	2090237	55.85	41548956	1389.45778	36.65852924	18941	10962	19614	21295	8	19582
CS0282_2	8649296	7995646	1.86	96.83	7847217	98.66	275167398	9201.999732	99.85285757	44	29859	220	1900	12	446
CS0523_2	3425091	3182264	98.3	0.53	54047	31.41	599761	20.05688392	7.246764539	27736	2167	29467	29497	1	29467
CS0037_2	11196907	10404994	0.08	35.26	10397003	35.29	130379528	4360.081865	99.72912417	81	29822	708	2845	6	713
CS0038_2	8938325	8335246	2.44	96.51	8132126	98.92	285976709	9563.478882	99.67227368	98	29805	347	1784	11	941
CS0039_2	7680273	7215994	0.77	98.28	7160665	99.04	252095276	8430.434271	99.85954586	42	29861	52	817	12	224
CS0286_2	7577904	7000914	90.25	1.59	682901	16.29	3951168	132.1328295	31.18081798	20579	9324	22892	25394	6	22793
CS0727_2	9530247	8924406	89.23	4.05	961344	37.58	12856459	429.9387687	17.19894325	24760	5143	25533	26391	2	25119
CS0287_2	7516329	7061375	5.77	93.2	6653598	98.91	233926652	7822.848945	99.85285757	44	29859	68	351	13	194
CS0040_2	5075015	4784184	59.65	32	1930261	79.31	54470269	1821.565361	43.73808648	16824	13079	18136	19735	6	17810
CS0288_2	7917399	7434344	5.28	93.6	7041747	98.82	247371946	8272.479216	99.80603953	58	29845	80	482	16	296
[illegible]	[illegible]	[illegible]	[illegible]	[illegible]	[illegible]	[illegible]	[illegible]	[illegible]	[illegible]	[illegible]	[illegible]	[illegible]	[illegible]	[illegible]	[illegible]

		RT-PCR		DRAGEN COVIDSeq Test Pipeline		In - Depth Custom Pipeline (Merged)														
Sample IDs Replicate 1	Sample IDs Replicate 2	RT (Pos/Neg) replicate1	RT (Pos/Neg) replicate 2	DRAGEN SARS-CoV-2 Detection Replicate 1	DRAGEN SARS-CoV-2 Detection Replicate 2	Total Reads_merged	Trimmed Reads q30-30bp_merged	Hisat2 Human % _merged	Hisat2 Covidall % _merged	Hisat2 Unmapped Covid Read#_merged	Hisat2 Unmapped Covid %_merged	BasePairescovered_merged	X coverage _merged	Genome coverage_merged	# bases with 0 coverage_merged	# bases with >0 coverage_merged	# bases with <10x coverage_merged	# bases with <100x coverage_merged	VARSCAN Variant Count_merged	N's_merged
CS0169_1	CS0169_2	Positive	Positive	SARS-CoV-2 Detected	SARS-CoV-2 Detected	17106701	15999130	25.14	72.12	11977706	96.33	410138680	13715.63656	99.86623416	40	29863	267	876	11	551
CS0170_1	CS0170_2	Positive	Positive	SARS-CoV-2 Detected	SARS-CoV-2 Detected	18186377	17160358	22.3	74.93	13333215	96.44	457142079	15287.49888	99.94314952	17	29886	79	137	12	339
CS0171_1	CS0171_2	Positive	Positive	SARS-CoV-2 Detected	SARS-CoV-2 Detected	20802936	19354938	14.71	83.36	16508099	97.73	573319608	19172.64515	99.96655854	10	29893	72	211	16	286
CS0634_1	CS0634_2	Positive	Positive	SARS-CoV-2 Detected	SARS-CoV-2 Detected	20894380	19726581	86.6	7.11	2643399	53.03	49862528	1667.475772	82.29274655	5295	24608	7935	11951	15	7687
CS0172_1	CS0172_2	Positive	Positive	SARS-CoV-2 Detected	SARS-CoV-2 Detected	18079813	17124816	5.67	93.16	16153350	98.77	567252335	18969.74668	99.97324683	8	29895	71	232	13	282
CS0173_1	CS0173_2	Positive	Positive	SARS-CoV-2 Detected	SARS-CoV-2 Detected	15501956	14578844	20.24	74.24	11627360	93.08	384774999	12867.43802	99.28435274	214	29689	360	927	12	844
CS0174_1	CS0174_2	Positive	Positive	SARS-CoV-2 Detected	SARS-CoV-2 Detected	16263368	15197179	29.34	64.05	10738776	90.64	345979895	11570.07307	99.82276026	53	29850	355	1042	17	583
CS0572_1	CS0572_2	Positive	Positive	SARS-CoV-2 Detected	SARS-CoV-2 Detected	17761726	15990317	87.33	2.71	2026391	21.41	15405581	515.1851319	43.9721767	16754	13149	17739	19549	9	17662
CS0175_1	CS0175_2	Positive	Positive	SARS-CoV-2 Detected	SARS-CoV-2 Detected	20695812	19421234	1.32	97.78	19165750	99.09	674810342	22566.64355	99.86623416	40	29863	46	62	13	8
CS0635_1	CS0635_2	Positive	Positive	SARS-CoV-2 Detected	SARS-CoV-2 Detected	27501943	25926776	59.97	35.27	10379486	88.1	325218156	10875.77019	99.84616928	46	29857	131	2609	15	1368
CS0405_1	CS0405_2	Pan Sarbeco	Pan Sarbeco	SARS-CoV-2 Not Detected	SARS-CoV-2 Not Detected	13893480	13037813	96.68	0.01	432484	0.35	53233	1.780189279	8.146339832	27467	2436	28588	29876	2	28543
CS0406_1	CS0406_2	Pan Sarbeco	Pan Sarbeco	SARS-CoV-2 Not Detected	SARS-CoV-2 Not Detected	18349251	17288917	95.34	0.07	806148	1.42	408321	13.65485068	7.179881617	27756	2147	28767	29098	0	28764
CS0636_1	CS0636_2	Positive	Positive	SARS-CoV-2 Detected	SARS-CoV-2 Detected	19481569	18355194	86.47	8.45	2482681	62.47	55114928	1843.1237	83.57689864	4911	24992	11012	13855	10	10888
CS0176_1	CS0176_2	Positive	Positive	SARS-CoV-2 Detected	SARS-CoV-2 Detected	12941379	12224521	33.1	62.58	8177765	93.55	271990674	9095.765442	99.4850015	154	29749	426	946	12	845
CS0637_1	CS0637_2	Inconclusive	Inconclusive	SARS-CoV-2 Detected	SARS-CoV-2 Detected	14647798	13744693	93.87	1.16	843126	18.98	5689268	190.2574324	9.226499013	27144	2759	28022	28395	0	28014
CS0177_1	CS0177_2	Positive	Positive	SARS-CoV-2 Detected	SARS-CoV-2 Detected	17541381	16532526	30.78	60.95	11442990	88.06	358259794	11980.73083	99.87961074	36	29867	68	399	15	560
CS0178_1	CS0178_2	Positive	Positive	SARS-CoV-2 Detected	SARS-CoV-2 Detected	18369317	17153727	38.01	57.14	10634209	92.18	348430758	11652.03351	99.86289001	41	29862	243	647	11	747
[illegible]																				

Sample IDs Replicate 1	In - Depth Custom Pipeline (Replicate 1)															
	Total Reads_Replicate1	Trimmed Reads q30-30bp_Replicate1	Kraken2 coronavirus %_Replicate1	Hisat2 Human % _Replicate1	Hisat2 Covidall % _Replicate1	Hisat2 Unmapped Covid Read#_Replicate1	Hisat2 Unmapped Covid %_Replicate1	BasePairescovered_Replicate1	X coverage _Replicate1	Genome coverage_Replicate1	# bases with 0 coverage_Replicate1	# bases with >0 coverage_Replicate1	# bases with <10x coverage_Replicate1	# bases with <100x coverage_Replicate1	VARSCAN Variant Count_Replicate1	N's_Replicate1
CS0169_1	8551635	8034941	0.49	25.12	72.12	6016369	96.32	205992751	6888.698492	99.70571515	88	29815	578	1784	12	864
CS0170_1	9093586	8613614	0.49	22.32	74.9	6690913	96.42	229378952	7670.767214	99.88295489	35	29868	85	381	10	386
CS0171_1	10422576	9751219	0.5	14.72	83.34	8315788	97.72	288796238	9657.76805	99.94983781	15	29888	79	462	16	485
CS0634_1	10544326	10009901	0.07	86.52	7.07	1349072	52.43	25157094	841.2899709	80.56716717	5811	24092	9014	14722	13	8627
CS0172_1	9074924	8630511	0.57	5.67	93.17	8141060	98.77	285921721	9561.640003	99.86289001	41	29862	80	758	13	291
CS0173_1	7790329	7357527	0.53	20.23	74.23	5869331	93.06	194197809	6494.258402	99.21078153	236	29667	549	4196	13	1325
CS0174_1	8141485	7643857	0.43	29.33	64.04	5402144	90.61	173997072	5818.716249	99.54854028	135	29768	540	2448	16	876
CS0572_1	8997291	8171288	0.03	87.29	2.68	1038425	21.12	7790955	260.5409156	43.20302311	16984	12919	18110	21495	9	18036
CS0175_1	10371955	9783033	0.57	1.32	97.79	9654038	99.09	339970561	11369.11216	99.86289001	41	29862	50	76	13	12
CS0635_1	13811749	13094066	0.26	59.93	35.17	5247278	87.75	163769770	5476.700331	99.81272782	56	29847	652	4223	14	2056
CS0405_1	6992268	6600439	0	96.66	0.01	220584	0.34	26824	0.8970337424	6.75183092	27884	2019	28924	29903	2	28863
CS0406_1	9253675	8768311	0	95.22	0.06	418862	1.36	202672	6.777647728	5.912450256	28135	1768	28803	29297	0	28769
CS0636_1	9828961	9314287	0.05	86.5	8.4	1257366	62.24	27812473	930.0897234	68.29080694	9482	20421	11650	15943	9	11498
CS0176_1	6483938	6147561	0.4	33.12	62.56	4111493	93.55	136751625	4573.174096	99.34120322	197	29706	629	4712	13	1109
CS0637_1	7367128	6955083	0.01	93.83	1.16	428989	18.78	2864054	95.77814935	8.142995686	27468	2435	28052	28491	0	25369
CS0177_1	8819290	8347920	0.42	30.83	60.9	5773859	88.04	180762352	6044.957095	99.73915661	78	29825	410	3611	15	846
CS0178_1	9172347	8603805	0.39	37.98	57.12	5336363	92.1	174710764	5842.583152	99.64886466	105	29798	314	2413	12	1266
[illegible]																

Sample IDs Replicate 1	In - Depth Custom Pipeline (Replicate 2)														
	Total Reads_Replicate2	Trimmed Reads q30-30bp_Replicate2	Hisat2 Human % _Replicate2	Hisat2 Covidall % _Replicate2	Hisat2 Unmapped Covid Read#_Replicate2	Hisat2 Unmapped Covid %_Replicate2	BasePairescovered_Replicate2	X coverage _Replicate2	Genome coverage_Replicate2	# bases with 0 coverage_Replicate2	# bases with >0 coverage_Replicate2	# bases with <10x coverage_Replicate2	# bases with <100x coverage_Replicate2	VARSCAN Variant Count_Replicate2	N's_Replicate2
CS0169_1	8555066	7964189	25.05	72.12	5968893	96.23	204145939	6826.938401	99.84951343	45	29858	470	2269	12	862
CS0170_1	9092791	8546744	22.26	74.97	6644229	96.43	227762653	7616.715814	99.85285757	44	29859	117	640	11	373
CS0171_1	10380360	9603719	14.68	83.38	8194277	97.72	284520148	9514.769354	99.95987025	12	29891	81	399	15	512
CS0634_1	10350054	9716680	86.01	7.15	1359482	51.1	24705434	826.1858008	79.65755944	6083	23820	9037	14936	15	8551
CS0172_1	9004889	8494305	5.68	93.16	8011763	98.77	281330301	9408.096211	99.93646122	19	29884	77	390	13	337
CS0173_1	7711627	7221317	20.22	74.24	5761147	93.06	190577156	6373.178477	99.17734007	246	29657	510	1130	13	1318
CS0174_1	8121883	7553322	29.29	64.07	5340997	90.6	171982727	5751.35361	99.74250075	77	29826	557	1215	16	878
CS0572_1	8764435	7819029	87.25	2.74	997120	21.51	7614626	254.6442163	43.36019797	16937	12966	18168	21509	9	18080
CS0175_1	10323857	9638201	1.31	97.78	9512241	99.08	334839427	11197.51955	99.85954586	42	29861	50	75	13	12
CS0635_1	13690194	12832710	59.91	35.38	5145245	88.24	161448392	5399.07006	99.84616928	46	29857	257	4568	15	1981
CS0405_1	6901212	6437374	96.6	0.01	218644	0.34	26409	0.8831555362	6.738454336	27888	2015	29062	29903	1	28921
CS0406_1	9095576	8520606	95.13	0.07	414873	1.39	205649	6.877202956	6.052904391	28093	1810	28803	29284	0	28797
CS0636_1	9652608	9040907	85.8	8.5	1284256	59.83	27302455	913.0339765	82.86793967	5123	24780	11658	15973	9	11509
CS0176_1	6457441	6076960	33.01	62.6	4071086	93.45	135239048	4522.591312	99.25759957	222	29681	543	3095	13	1039
CS0637_1	7280670	6789610	93.87	1.17	416419	19.09	2825214	94.47928302	7.661438652	27612	2291	28051	28486	0	28025
CS0177_1	8722091	8184606	30.74	61	5668261	88.08	177497173	5935.764739	99.85620172	43	29860	184	478	15	848
CS0178_1	9196970	8549922	37.96	57.17	5303953	92.15	173720039	5809.451861	99.73915661	78	29825	401	2519	12	1163
[illegible]															

Sample IDs Replicate 1	Sample IDs Replicate 2	RT (Pos/Neg) replicate1	RT (Pos/Neg) replicate 2	DRAGEN SARS-CoV-2 Detection Replicate 1	DRAGEN SARS-CoV-2 Detection Replicate 2	Total Reads_merged	Trimmed Reads q30-30bp_merged	Hisat2 Human % _merged	Hisat2 Covidall % _merged	Hisat2 Unmapped Covid Read#_merged	Hisat2 Unmapped Covid %_merged	BasePairescovered_merged	X coverage _merged	Genome coverage_merged	# bases with 0 coverage_merged	# bases with >0 coverage_merged
[illegible]																
CS0443_1	CS0443_2	Positive	Positive	SARS-CoV-2 Detected	SARS-CoV-2 Detected	19276563	17885237	1.57	97.74	17604362	99.3	621254979	20775.67398	99.95987025	12	29891
CS0444_1	CS0444_2	Positive	Positive	SARS-CoV-2 Detected	SARS-CoV-2 Detected	19855705	18154357	2.98	96.15	17614103	99.1	620195717	20740.25071	99.84951343	45	29858
CS0445_1	CS0445_2	Positive	Positive	SARS-CoV-2 Detected	SARS-CoV-2 Detected	22756475	21231408	0.19	98.91	21190560	99.1	746295208	24957.20189	99.86289001	41	29862
CS0446_1	CS0446_2	Positive	Positive	SARS-CoV-2 Detected	SARS-CoV-2 Detected	19794201	18595343	4.56	94.47	17747219	98.99	624636907	20888.77059	99.7458449	76	29827
CS0447_1	CS0447_2	Positive	Positive	SARS-CoV-2 Detected	SARS-CoV-2 Detected	21119505	19852007	0.14	98.97	19824334	99.11	698394528	23355.33318	99.96321439	11	29892
CS0448_1	CS0448_2	Positive	Positive	SARS-CoV-2 Detected	SARS-CoV-2 Detected	19901730	18672407	14.53	83.66	15958713	97.88	555234557	18567.85463	99.97324683	8	29895
CS0449_1	CS0449_2	Positive	Positive	SARS-CoV-2 Detected	SARS-CoV-2 Detected	20536449	19201318	1.2	97.87	18970107	99.06	667896751	22335.44297	99.97993512	6	29897
CS0450_1	CS0450_2	Positive	Positive	SARS-CoV-2 Detected	SARS-CoV-2 Detected	17292847	16298665	1.92	97.41	15985442	99.32	564475699	18876.89192	99.82276026	53	29850
CS0451_1	CS0451_2	Positive	Positive	SARS-CoV-2 Detected	SARS-CoV-2 Detected	7872993	7395599	3.31	95.8	7150575	99.08	251844353	8422.043039	99.89298733	32	29871
CS0452_1	CS0452_2	Positive	Positive	SARS-CoV-2 Detected	SARS-CoV-2 Detected	5971867	5513632	0.53	98.81	5484587	99.33	193620170	6474.94131	99.81941611	54	29849
CS0453_1	CS0453_2	Positive	Positive	SARS-CoV-2 Detected	SARS-CoV-2 Detected	19973691	18361035	0.13	99.18	18337508	99.31	646891552	21632.99843	99.96990269	9	29894
CS0454_1	CS0454_2	Positive	Positive	SARS-CoV-2 Detected	SARS-CoV-2 Detected	9411693	8830188	5.22	94.16	8369042	99.35	295941530	9896.717052	25.5425877	22265	7638
CS0455_1	CS0455_2	Positive	Positive	SARS-CoV-2 Detected	SARS-CoV-2 Detected	18388508	17211852	0.32	98.96	17156134	99.28	605654090	20253.95746	99.80603953	58	29845
CS0456_1	CS0456_2	Positive	Positive	SARS-CoV-2 Detected	SARS-CoV-2 Detected	18021328	16879037	3.18	95.84	16342890	98.99	575078270	19231.45738	99.82944855	51	29852
CS0457_1	CS0457_2	Positive	Positive	SARS-CoV-2 Detected	SARS-CoV-2 Detected	21413495	20174653	1.41	97.79	19890734	99.19	701332398	23453.57984	99.97324683	8	29895
CS0458_1	CS0458_2	Positive	Positive	SARS-CoV-2 Detected	SARS-CoV-2 Detected	19776653	18605873	0.42	99.02	18528509	99.43	655019987	21904.82517	99.96655854	10	29893
CS0459_1	CS0459_2	Positive	Positive	SARS-CoV-2 Detected	SARS-CoV-2 Detected	26225941	24661720	0.08	99.23	24641127	99.31	870043470	29095.52453	99.8762666	37	29866
CS0460_1	CS0460_2	Positive	Positive	SARS-CoV-2 Detected	SARS-CoV-2 Detected	18938574	17765551	0.07	99.29	17752683	99.36	627102363	20971.21904	99.97659098	7	29896
CS0461_1	CS0461_2	Positive	Positive	SARS-CoV-2 Detected	SARS-CoV-2 Detected	17702569	16695946	1.93	97.29	16373433	99.21	577551461	19314.1645	99.88295489	35	29868
CS0462_1	CS0462_2	Positive	Positive	SARS-CoV-2 Detected	SARS-CoV-2 Detected	17023225	15698260	0.57	98.57	15609335	99.13	549849342	18387.76517	99.85620172	43	29860
CS0463_1	CS0463_2	Positive	Positive	SARS-CoV-2 Detected	SARS-CoV-2 Detected	17748575	16676971	0.52	98.71	16589883	99.23	585236741	19571.17149	99.85954586	42	29861
CS0464_1	CS0464_2	Positive	Positive	SARS-CoV-2 Detected	SARS-CoV-2 Detected	20358141	19017900	0.01	99.32	19016891	99.32	671353993	22451.05819	99.80603953	58	29845
CS0465_1	CS0465_2	Positive	Positive	SARS-CoV-2 Detected	SARS-CoV-2 Detected	18196953	17111987	0.41	98.83	17041403	99.24	601180463	20104.35284	99.94314952	17	29886
CS0466_1	CS0466_2	Positive	Positive	SARS-CoV-2 Detected	SARS-CoV-2 Detected	17928413	16859816	0.33	98.83	16803499	99.16	592469429	19813.04314	99.95987025	12	29891
CS0467_1	CS0467_2	Positive	Positive	SARS-CoV-2 Detected	SARS-CoV-2 Detected	17793316	16719443	0.85	98.52	16577909	99.36	585643211	19584.76444	99.97659098	7	29896
CS0468_1	CS0468_2	Positive	Positive	SARS-CoV-2 Detected	SARS-CoV-2 Detected	21058725	18616777	0.38	98.85	18545461	99.23	653248039	21845.56864	99.97993512	6	29897
CS0469_1	CS0469_2	Positive	Positive	SARS-CoV-2 Not Detected	SARS-CoV-2 Not Detected	11464038	10786698	97.76	0	241318	0.14	9859	0.3296993613	1.989766913	29308	595
CS0470_1	CS0470_2	Positive	Positive	SARS-CoV-2 Detected	SARS-CoV-2 Detected	20290311	19090894	7.26	91.86	17704042	99.05	623594078	20853.89687	99.75922148	72	29831
CS0471_1	CS0471_2	Positive	Positive	SARS-CoV-2 Detected	SARS-CoV-2 Detected	21212513	19978433	3.98	95.17	19183345	99.11	676381372	22619.18109	99.10376885	268	29635
CS0472_1	CS0472_2	Positive	Positive	Invalid	Invalid	3142467	2951313	96.92	0.04	90878	1.31	34859	1.165735879	0.739056282	29682	221
CS0473_1	CS0473_2	Positive	Positive	SARS-CoV-2 Detected	SARS-CoV-2 Detected	12092863	11323040	17.37	82	9356511	99.23	330435012	11050.22948	26.45219543	21993	7910
CS0474_1	CS0474_2	Positive	Positive	SARS-CoV-2 Detected	SARS-CoV-2 Detected	20538872	19053579	2.97	96.08	18488182	99.02	650610925	21757.37969	99.75253319	74	29829
CS0475_1	CS0475_2	Positive	Positive	SARS-CoV-2 Detected	SARS-CoV-2 Detected	20593918	19344727	0.37	98.9	19273411	99.27	680165916	22745.74177	99.76590977	70	29833
CS0476_1	CS0476_2	Positive	Positive	SARS-CoV-2 Detected	SARS-CoV-2 Detected	21167623	19855977	24.01	74.08	15088265	97.49	523106100	17493.4321	99.85954586	42	29861
CS0477_1	CS0477_2	Positive	Positive	SARS-CoV-2 Detected	SARS-CoV-2 Detected	18930062	17648760	46.9	35.67	9372136	67.17	223846343	7485.748687	97.76945457	667	29236
CS0478_1	CS0478_2	Positive	Positive	SARS-CoV-2 Detected	SARS-CoV-2 Detected	24115599	22400518	6.9	92.14	20854848	98.96	733711783	24536.39377	99.87961074	36	29867
CS0479_1	CS0479_2	Positive	Positive	SARS-CoV-2 Detected	SARS-CoV-2 Detected	23471550	22163429	88.54	7.78	2540993	67.84	61355148	2051.805772	51.40286928	14532	15371
CS0480_1	CS0480_2	Positive	Positive	SARS-CoV-2 Detected	SARS-CoV-2 Detected	16137692	15194174	81.78	15.18	2768009	83.33	82029193	2743.176036	59.2147945	12196	17707
CS0481_1	CS0481_2	Positive	Positive	SARS-CoV-2 Detected	SARS-CoV-2 Detected	21194786	19890942	0.01	98.99	19889729	99	699833156	23403.443	99.75587734	73	29830
CS0482_1	CS0482_2	Positive	Positive	SARS-CoV-2 Detected	SARS-CoV-2 Detected	5094403	4800314	62.65	36.12	1792787	96.72	61693484	2063.120222	11.1928569	26556	3347
CS0483_1	CS0483_2	Positive	Positive	SARS-CoV-2 Detected	SARS-CoV-2 Detected	14655179	13727122	0.59	98.48	13646162	99.06	480378616	16064.56262	99.84616928	46	29857
CS0484_1	CS0484_2	Positive	Positive	SARS-CoV-2 Detected	SARS-CoV-2 Detected	2671780	2507545	73.56	22.15	662941	83.79	19706581	659.0168545	49.12884995	15212	14691
CS0485_1	CS0485_2	Positive	Positive	SARS-CoV-2 Detected	SARS-CoV-2 Detected	18791154	17684253	0.13	98.88	17660667	99.01	621489220	20783.50734	99.87292245	38	29865
CS0486_1	CS0486_2	Positive	Positive	SARS-CoV-2 Not Detected	SARS-CoV-2 Not Detected	20183146	18990453	94.98	0.28	953643	5.6	1901010	63.57255125	4.728622546	28489	1414
CS0487_1	CS0487_2	Positive	Positive	SARS-CoV-2 Detected	SARS-CoV-2 Detected	13010313	12245812	83.15	11.99	2063634	71.17	52255337	1747.4948	18.44630973	24387	5516
CS0488_1	CS0488_2	Positive	Positive	SARS-CoV-2 Detected	SARS-CoV-2 Detected	5767283	5429818	61.68	37.03	2080693	96.65	71561048	2393.105976	22.72347256	23108	6795
CS0489_1	CS0489_2	Positive	Positive	SARS-CoV-2 Detected	SARS-CoV-2 Detected	17175628	16127259	0.03	99.14	16122721	99.17	568359366	19006.76741	99.97324683	8	29895
CS0490_1	CS0490_2	Positive	Positive	SARS-CoV-2 Detected	SARS-CoV-2 Detected	19641783	18520220	0.18	98.91	18486460	99.09	651098626	21773.68913	99.98662342	4	29899
CS0491_1	CS0491_2	Positive	Positive	SARS-CoV-2 Detected	SARS-CoV-2 Detected	15354757	14378774	15.65	82.93	12128451	98.32	423834898	14173.65809	97.65240946	702	29201
CS0492_1	CS0492_2	Positive	Positive	SARS-CoV-2 Detected	SARS-CoV-2 Detected	8768639	8227162	56.9	41.87	3546210	97.15	122502251	4096.654215	45.76798315	16217	13686
CS0493_1	CS0493_2	Positive	Positive	SARS-CoV-2 Detected	SARS-CoV-2 Detected	19364573	18165561	0.68	98.74	18042405	99.42	638031986	21336.7216	99.70905929	87	29816
CS0494_1	CS0494_2	Positive	Positive	SARS-CoV-2 Not Detected	SARS-CoV-2 Not Detected	15059788	14129702	88.33	6.64	1649347	56.91	33319434	1114.250543	4.574791827	28535	1368
CS0495_1	CS0495_2	Positive	Positive	SARS-CoV-2 Detected	SARS-CoV-2 Detected	21906558	20610543	24.52	73.86	15557328	97.85	541411531	18105.59245	99.7458449	76	29827
CS0496_1	CS0496_2	Positive	Positive	Invalid	Invalid	1195537	1116210	92.1	0.14	88203	1.75	45525	1.522422499	0.6621409223	29705	198
CS0497_1	CS0497_2	Positive	Positive	SARS-CoV-2 Detected	SARS-CoV-2 Detected	16598014	15634388	0.4	98.85	15572452	99.24	549388740	18372.36197	99.92308464	23	29880
CS0498_1	CS0498_2	Positive	Positive	SARS-CoV-2 Detected	SARS-CoV-2 Detected	16495378	15499028	2.51	96.7	15109699	99.19	532936029	17822.15928	99.2743203	217	29686
[illegible]																

		RT-PCR		DRAGEN COVIDSeq Test Pipeline	
Sample IDs Replicate 1	Sample IDs Replicate 2	RT (Pos/Neg) replicate1	RT (Pos/Neg) replicate 2	DRAGEN SARS-CoV-2 Detection Replicate 1	DRAGEN SARS-CoV-2 Detection Replicate 2

In - Depth Custom Pipeline (Merged)														
Total Reads_merged	Trimmed Reads q30-30bp_merged	Hisat2 Human % _merged	Hisat2 Covidall % _merged	Hisat2 Unmapped Covid Read#_merged	Hisat2 Unmapped Covid %_merged	BasePairescovered_merged	X coverage _merged	Genome coverage_merged	# bases with 0 coverage_merged	# bases with >0 coverage_merged	# bases with <10x coverage_merged	# bases with <100x coverage_merged	VARSCAN Variant Count_merged	N's_merged

In - Depth Custom Pipeline (Replicate 1)															
Total Reads_Replicate1	Trimmed Reads q30-30bp_Replicate1	Kraken2 coronavirus %_Replicate1	Hisat2 Human % _Replicate1	Hisat2 Covidall % _Replicate1	Hisat2 Unmapped Covid Read#_Replicate1	Hisat2 Unmapped Covid %_Replicate1	BasePairescovered_Replicate1	X coverage _Replicate1	Genome coverage_Replicate1	# bases with 0 coverage_Replicate1	# bases with >0 coverage_Replicate1	# bases with <10x coverage_Replicate1	# bases with <100x coverage_Replicate1	VARSCAN Variant Count_Replicate1	N's_Replicate1

In - Depth Custom Pipeline (Replicate 2)															
Total Reads_Replicate2	Trimmed Reads q30-30bp_Replicate2	Hisat2 Human % _Replicate2	Hisat2 Covidall % _Replicate2	Hisat2 Unmapped Covid Read#_Replicate2	Hisat2 Unmapped Covid %_Replicate2	BasePairescovered_Replicate2	X coverage _Replicate2	Genome coverage_Replicate2	# bases with 0 coverage_Replicate2	# bases with >0 coverage_Replicate2	# bases with <10x coverage_Replicate2	# bases with <100x coverage_Replicate2	VARSCAN Variant Count_Replicate2	N's_Replicate2	
