# Supplementary material for: High throughput detection and genetic epidemiology of SARS-CoV-2 using COVIDSeq next-generation sequencing
Source: PLoS One. 2021 Feb 17;16(2):e0247115. doi: 10.1371/journal.pone.0247115 (PMC7888613; doi:10.1371/journal.pone.0247115)
Supplement: S3 Table — (PDF) [file pone.0247115.s004.pdf]

Chr Start End Ref Alt Func.refGene Gene.refGene GeneDetail.refGene
ExonicFunc.refGene AAChange.refGene GERP++_RS PhyoP44wayCoV_score
PhastCons44wayCoV_score SIFT_score SIFT_pred UNIPROT_Disulphite_Bonds
UNIPROT_domains UNIPROT_glyphos UNIPROT_Transmembrane b_CellEpitopes
cd4_Epitopes cd4_Epitope_Score cd8_Epitopes cd8_Epitope_Score MPDI_Potential
Immunogenic Regions MPDI_Potential Immunodominant Epitopes
ARTIC_Primers RT_PCR-Primer_Probe_Name
RT_PCR-Primer_Probe_Organization Sequencing_Error_Sites Homoplasic_positions
Hypermutable_Sites Otherinfo
1 21 21 C T upstream ORF1adist=245 . . 1.38
2.76482 1 . . . .
. . . . . . sequencing/mapping error site . .

1 105 105 G T upstream ORF1adist=161 . . 1.57
4.03232 1 . . . .
. . . .
1 106 106 C T upstream ORF1adist=160 . . -3.15
0.00613386 0.992126 . . . .
. . . .

1 128 128 T C upstream ORF1adist=138 . . 1.6
2.16835 1 . . . .
. . . .
1 147 147 C T upstream ORF1adist=119 . . 0.736
1.87011 1 . . . .
. . . .
1 203 203 C T upstream ORF1adist=63. . 1.6
3.21217 1 . . . .
. . . .
1 222 222 C T upstream ORF1adist=44. . 0.741
1.87011 1 . . . .
. . . .
1 241 241 C T upstream ORF1adist=25. . 1.61
3.28673 1 . . . .
. . . .
1 313 313 C T exonic ORF1a. synonymous SNV
ORF1a:cds-YP_009724389.1:exon1:c.C48T:p.L16L,ORF1a:cds-YP_009725295.1:exon1:c.C48
T:p.L16L 1.65 3.28673 1 0.84 T . . . . . .
orf1ab polyprotein_KTHVQLSLPVLQVRD 19 . . . . . .
. . . .
1 343 343 G A exonic ORF1a. synonymous SNV
ORF1a:cds-YP_009724389.1:exon1:c.G78A:p.V26V,ORF1a:cds-YP_009725295.1:exon1:c.G7
8A:p.V26V 1.65 4.256 1 0.66 T . . . . . .

. . . .

1 344 344 C T exonic ORF1a. nonsynonymous SNV
ORF1a:cds-YP_009724389.1:exon1:c.C79T:p.L27F,ORF1a:cds-YP_009725295.1:exon1:c.C79
T:p.L27F -1.14 0.677165 1 0.02 D . . . . .
. . . .

1 368 368 G T exonic ORF1a. nonsynonymous SNV
ORF1a:cds-YP_009724389.1:exon1:c.G103T:p.V35L,ORF1a:cds-YP_009725295.1:exon1:c.G1
03T:p.V35L 1.65 4.256 1 0.04 D . . . . . .
. . . .

1 370 370 G T exonic ORF1a. synonymous SNV
ORF1a:cds-YP_009724389.1:exon1:c.G105T:p.V35V,ORF1a:cds-YP_009725295.1:exon1:c.G
105T:p.V35V -1.58 0.975402 1 1 T . . . . .
. . . .

1 395 395 C T exonic ORF1a. stopgain
ORF1a:cds-YP_009724389.1:exon1:c.C130T:p.Q44X,ORF1a:cds-YP_009725295.1:exon1:c.C
130T:p.Q44X 0.73 1.3482 1 . . . .
. . . . . nCoV-2019_1_RIGHT . . . .
.
1 408 408 A G exonic ORF1a. nonsynonymous SNV
ORF1a:cds-YP_009724389.1:exon1:c.A143G:p.D48G,ORF1a:cds-YP_009725295.1:exon1:c.A
143G:p.D48G -1.92 0.155252 1 0.29 T . . . . .
. . . . . . nCoV-2019_1_RIGHT . . .
. .
1 442 442 C T exonic ORF1a. synonymous SNV
ORF1a:cds-YP_009724389.1:exon1:c.C177T:p.G59G,ORF1a:cds-YP_009725295.1:exon1:c.C
177T:p.G59G -3.3 -0.51578 0.700787 0.81 T . . . .
. . . orf1ab_GLVEVEKGV 0.12 . . . .
. .
1 469 469 T C exonic ORF1a. synonymous SNV
ORF1a:cds-YP_009724389.1:exon1:c.T204C:p.Y68Y,ORF1a:cds-YP_009725295.1:exon1:c.T2
04C:p.Y68Y -2.18 0.0806929 1 0.49 T . . . . .
orf1ab polyprotein_QPYVFIKRSDARTAP 19 . . . .
. . . .
1 487 487 G A exonic ORF1a. synonymous SNV
ORF1a:cds-YP_009724389.1:exon1:c.G222A:p.S74S,ORF1a:cds-YP_009725295.1:exon1:c.G
222A:p.S74S 1.65 0.900843 1 1 T . . . . .
orf1ab polyprotein_QPYVFIKRSDARTAP 19 . . . .
. . . .
1 527 527 C T exonic ORF1a. synonymous SNV

ORF1a:cds-YP_009724389.1:exon1:c.C262T:p.L88L,ORF1a:cds-YP_009725295.1:exon1:c.C2
62T:p.L88L -3.3 -2.37976 0 1 T . . . . .
. . orf1ab_APHGHVMVEL 0.03 . . . . . .
. .
1 574 574 A G exonic ORF1a. synonymous SNV
ORF1a:cds-YP_009724389.1:exon1:c.A309G:p.T103T,ORF1a:cds-YP_009725295.1:exon1:c.A
309G:p.T103T -3.3 -0.739457 0.976378 1 T . . . .
. . . orf1ab_TLGVLVPHV 0.1 . . . . . .
. .
1 593 593 C T exonic ORF1a. nonsynonymous SNV
ORF1a:cds-YP_009724389.1:exon1:c.C328T:p.H110Y,ORF1a:cds-YP_009725295.1:exon1:c.C
328T:p.H110Y -0.157 1.19908 1 1 T . . . . .
. . orf1ab_TLGVLVPHV 0.1 . . . .
.
1 610 610 A T exonic ORF1a. synonymous SNV
ORF1a:cds-YP_009724389.1:exon1:c.A345T:p.P115P,ORF1a:cds-YP_009725295.1:exon1:c.A
345T:p.P115P 0.512 1.04996 0.992126 0.19 T . . . .
. . . orf1ab_VPHVGEIPVAY -1 . . . . .
. . .
1 626 626 G A exonic ORF1a. nonsynonymous SNV
ORF1a:cds-YP_009724389.1:exon1:c.G361A:p.V121I,ORF1a:cds-YP_009725295.1:exon1:c.G
361A:p.V121I 1.65 4.256 1 0.46 T . . . . . .
. orf1ab_GEIPVAYRKVLL -1 . . . .
.
1 679 679 C T exonic ORF1a. synonymous SNV
ORF1a:cds-YP_009724389.1:exon1:c.C414T:p.A138A,ORF1a:cds-YP_009725295.1:exon1:c.C
414T:p.A138A 0.776 1.94467 1 0.53 T . . . . .
. . orf1ab_SYGADLKSF -1 . . . .
.
1 753 753 C A exonic ORF1a. nonsynonymous SNV
ORF1a:cds-YP_009724389.1:exon1:c.C488A:p.T163N,ORF1a:cds-YP_009725295.1:exon1:c.C
488A:p.T163N 1.65 3.28673 1 0.06 T . . . . .
. . . .

1 761 761 A G exonic ORF1a. nonsynonymous SNV
ORF1a:cds-YP_009724389.1:exon1:c.A496G:p.S166G,ORF1a:cds-YP_009725295.1:exon1:c.
A496G:p.S166G 0.737 2.6157 1 0.58 T . . . . .
. . . .

1 774 774 C T exonic ORF1a. nonsynonymous SNV
ORF1a:cds-YP_009724389.1:exon1:c.C509T:p.T170I,ORF1a:cds-YP_009725295.1:exon1:c.C
509T:p.T170I -1.96 -0.0684252 0.0551181 0.16 T . . . .
. . . .

1 823 823 C T exonic ORF1a. synonymous SNV
ORF1a:cds-YP_009724389.1:exon1:c.C558T:p.V186V,ORF1a:cds-YP_009725295.1:exon1:c.C
558T:p.V186V -3.3 -0.0684252 0.992126 1 T . . . .
. . . orf1ab_YVDNNFCGPDGY 0.05 . . . . .
. . .
1 835 835 C T exonic ORF1a. synonymous SNV
ORF1a:cds-YP_009724389.1:exon1:c.C570T:p.F190F,ORF1a:cds-YP_009725295.1:exon1:c.C
570T:p.F190F -3.3 -0.590339 0.944882 0.42 T . . . .
. . . orf1ab_YVDNNFCGPDGY 0.05 . . . . .
. . .
1 840 840 G A exonic ORF1a. nonsynonymous SNV
ORF1a:cds-YP_009724389.1:exon1:c.G575A:p.G192D,ORF1a:cds-YP_009725295.1:exon1:c.
G575A:p.G192D 1.65 4.256 1 0 D . . . . .
. . orf1ab_YVDNNFCGPDGY 0.05 . . . . . .
. .
1 841 841 C T exonic ORF1a. synonymous SNV
ORF1a:cds-YP_009724389.1:exon1:c.C576T:p.G192G,ORF1a:cds-YP_009725295.1:exon1:c.
C576T:p.G192G -0.238 1.04996 1 1 T . . . .
. . . orf1ab_YVDNNFCGPDGY 0.05 . . . . .
. . .
1 874 874 C T exonic ORF1a. synonymous SNV
ORF1a:cds-YP_009724389.1:exon1:c.C609T:p.D203D,ORF1a:cds-YP_009725295.1:exon1:c.
C609T:p.D203D -3.3 0.155252 0.992126 0.83 T . . .
. . . .
.
1 886 886 T G exonic ORF1a. synonymous SNV
ORF1a:cds-YP_009724389.1:exon1:c.T621G:p.R207R,ORF1a:cds-YP_009725295.1:exon1:c.T
621G:p.R207R -3.3 -1.63417 0.661417 0.83 T . . .
. . . .
.
1 904 904 C T exonic ORF1a. synonymous SNV
ORF1a:cds-YP_009724389.1:exon1:c.C639T:p.C213C,ORF1a:cds-YP_009725295.1:exon1:c.
C639T:p.C213C -1.3 1.79555 1 0.28 T . . . .
. . . .

1 912 912 C A exonic ORF1a. nonsynonymous SNV
ORF1a:cds-YP_009724389.1:exon1:c.C647A:p.S216Y,ORF1a:cds-YP_009725295.1:exon1:c.C
647A:p.S216Y 1.65 3.28673 1 0.01 D . . . . .
. . . .

1 933 933 A G exonic ORF1a. nonsynonymous SNV
ORF1a:cds-YP_009724389.1:exon1:c.A668G:p.D223G,ORF1a:cds-YP_009725295.1:exon1:c.

A668G:p.D223G 1.65 2.24291 0.984252 0.05 D . . .
. . . . orf1ab_FIDTKRGVY 0.05 . . . . .
. . .
1 936 936 C T exonic ORF1a. nonsynonymous SNV
ORF1a:cds-YP_009724389.1:exon1:c.C671T:p.T224I,ORF1a:cds-YP_009725295.1:exon1:c.C
671T:p.T224I 1.65 3.28673 1 0.05 D . . . . .
. . orf1ab_FIDTKRGVY 0.05 . . . .
.
1 944 944 G A exonic ORF1a. nonsynonymous SNV
ORF1a:cds-YP_009724389.1:exon1:c.G679A:p.G227S,ORF1a:cds-YP_009725295.1:exon1:c.
G679A:p.G227S 1.65 4.256 1 0.56 T . . . . .
. . orf1ab_FIDTKRGVY 0.05 . . nCoV-2019_4_LEFT . .
. . .
1 950 950 T C exonic ORF1a. nonsynonymous SNV
ORF1a:cds-YP_009724389.1:exon1:c.T685C:p.Y229H,ORF1a:cds-YP_009725295.1:exon1:c.T
685C:p.Y229H 1.65 2.16835 1 0 D . . . .
. . . orf1ab_FIDTKRGVY 0.05 . . nCoV-2019_4_LEFT .
. . . .
1 959 959 C T exonic ORF1a. nonsynonymous SNV
ORF1a:cds-YP_009724389.1:exon1:c.C694T:p.R232C,ORF1a:cds-YP_009725295.1:exon1:c.
C694T:p.R232C 1.65 3.28673 1 0 D . . . .
. . . orf1ab_REHEHEIAWY -1 . . nCoV-2019_4_LEFT
. . . . .
1 1018 1018 G T exonic ORF1a. nonsynonymous SNV
ORF1a:cds-YP_009724389.1:exon1:c.G753T:p.L251F,ORF1a:cds-YP_009725295.1:exon1:c.G
753T:p.L251F -3.3 -1.03769 0.133858 0.03 D . . . .
. . . orf1ab_YELQTPFEIKL 0.18 . .
nCoV-2019_3_RIGHT . . . . .
1 1052 1052 T C exonic ORF1a. nonsynonymous SNV
ORF1a:cds-YP_009724389.1:exon1:c.T787C:p.F263L,ORF1a:cds-YP_009725295.1:exon1:c.T
787C:p.F263L 1.65 2.20517 1 0.44 T . . . . .
. . orf1ab_FEIKLAKKF -1 . . . .
.
1 1059 1059 C T exonic ORF1a. nonsynonymous SNV
ORF1a:cds-YP_009724389.1:exon1:c.C794T:p.T265I,ORF1a:cds-YP_009725295.1:exon1:c.C
794T:p.T265I 1.65 3.30383 0.992126 0.03 D . . . .
. . . .
hypermutable low-fitness site
1 1101 1101 C T exonic ORF1a. nonsynonymous SNV
ORF1a:cds-YP_009724389.1:exon1:c.C836T:p.S279F,ORF1a:cds-YP_009725295.1:exon1:c.C
836T:p.S279F 1.65 3.30383 0.440945 0.01 D . . . .
. orf1ab polyprotein_PNFVFPLNSIIKTIQ 15 . . . . .
. . . . .

1 1148 1148 G T exonic ORF1a. nonsynonymous SNV
ORF1a:cds-YP_009724389.1:exon1:c.G883T:p.G295C,ORF1a:cds-YP_009725295.1:exon1:c.
G883T:p.G295C 0.745 2.71787 0.992126 0.02 D . . .
. . . .
.
1 1150 1150 C T exonic ORF1a. synonymous SNV
ORF1a:cds-YP_009724389.1:exon1:c.C885T:p.G295G,ORF1a:cds-YP_009725295.1:exon1:c.
C885T:p.G295G -2.5 0.00784252 1 1 T . . . .
. orf1ab polyprotein_FMGRIRSVYPVASPN 20 . . . . .
. . . . .
1 1161 1161 G A exonic ORF1a. nonsynonymous SNV
ORF1a:cds-YP_009724389.1:exon1:c.G896A:p.R299K,ORF1a:cds-YP_009725295.1:exon1:c.
G896A:p.R299K 1.65 4.256 0.984252 0.99 T . . . . .
. orf1ab polyprotein_FMGRIRSVYPVASPN 20 . . . . .
. . . . .
1 1167 1167 G C exonic ORF1a. nonsynonymous SNV
ORF1a:cds-YP_009724389.1:exon1:c.G902C:p.R301P,ORF1a:cds-YP_009725295.1:exon1:c.
G902C:p.R301P 1.65 4.256 1 0.08 T . . . . .
orf1ab polyprotein_FMGRIRSVYPVASPN 20 . . . . . .
. . . .
1 1218 1218 C T exonic ORF1a. nonsynonymous SNV
ORF1a:cds-YP_009724389.1:exon1:c.C953T:p.S318L,ORF1a:cds-YP_009725295.1:exon1:c.C
953T:p.S318L 1.65 3.30383 1 0.08 T . . . . .
. . . .

1 1242 1242 G T exonic ORF1a. nonsynonymous SNV
ORF1a:cds-YP_009724389.1:exon1:c.G977T:p.C326F,ORF1a:cds-YP_009725295.1:exon1:c.
G977T:p.C326F 1.65 4.256 1 0 D . . . . .
. . . . . . nCoV-2019_5_LEFT . . . .
.
1 1261 1261 G T exonic ORF1a. nonsynonymous SNV
ORF1a:cds-YP_009724389.1:exon1:c.G996T:p.Q332H,ORF1a:cds-YP_009725295.1:exon1:c.
G996T:p.Q332H 1.65 4.256 1 0.12 T . . . . .
. . . . . . nCoV-2019_5_LEFT . . . .
.
1 1263 1263 C T exonic ORF1a. nonsynonymous SNV
ORF1a:cds-YP_009724389.1:exon1:c.C998T:p.T333M,ORF1a:cds-YP_009725295.1:exon1:c.
C998T:p.T333M 1.65 3.30383 1 0.09 T . . . .
. . . . nCoV-2019_5_LEFT . . .
. .
1 1269 1269 A G exonic ORF1a. nonsynonymous SNV
ORF1a:cds-YP_009724389.1:exon1:c.A1004G:p.D335G,ORF1a:cds-YP_009725295.1:exon1:c
.A1004G:p.D335G 1.65 2.27841 0.96063 0.03 D . . .

. . . .
.
1 1281 1281 C T exonic ORF1a. nonsynonymous SNV
ORF1a:cds-YP_009724389.1:exon1:c.C1016T:p.A339V,ORF1a:cds-YP_009725295.1:exon1:c.
C1016T:p.A339V 1.65 3.30383 1 0.27 T . . . .
. . . .

1 1288 1288 C T exonic ORF1a. synonymous SNV
ORF1a:cds-YP_009724389.1:exon1:c.C1023T:p.C341C,ORF1a:cds-YP_009725295.1:exon1:c.
C1023T:p.C341C 0.326 0.300819 1 1 T . . . .
. . . .

1 1292 1292 T C exonic ORF1a. nonsynonymous SNV
ORF1a:cds-YP_009724389.1:exon1:c.T1027C:p.F343L,ORF1a:cds-YP_009725295.1:exon1:c.
T1027C:p.F343L -1.58 0.154331 0.929134 0.68 T . . .
. . . .
.
1 1314 1314 C T exonic ORF1a. nonsynonymous SNV
ORF1a:cds-YP_009724389.1:exon1:c.C1049T:p.T350I,ORF1a:cds-YP_009725295.1:exon1:c.
C1049T:p.T350I -0.766 0.0810866 0 0.23 T . . . .
. . . . nCoV-2019_4_RIGHT . .
. . .
1 1326 1326 C T exonic ORF1a. nonsynonymous SNV
ORF1a:cds-YP_009724389.1:exon1:c.C1061T:p.A354V,ORF1a:cds-YP_009725295.1:exon1:c.
C1061T:p.A354V 1.65 3.30383 1 1 T . . . .
. . . . nCoV-2019_4_RIGHT . .
. . .
1 1354 1354 T C exonic ORF1a. synonymous SNV
ORF1a:cds-YP_009724389.1:exon1:c.T1089C:p.N363N,ORF1a:cds-YP_009725295.1:exon1:c.
T1089C:p.N363N 1.65 2.20517 1 1 T . . . .
. . . .

1 1361 1361 G T exonic ORF1a. nonsynonymous SNV
ORF1a:cds-YP_009724389.1:exon1:c.G1096T:p.V366F,ORF1a:cds-YP_009725295.1:exon1:c.
G1096T:p.V366F -0.211 1.17975 0.992126 0.19 T . . .
. . . .
.
1 1387 1387 C T exonic ORF1a. synonymous SNV
ORF1a:cds-YP_009724389.1:exon1:c.C1122T:p.H374H,ORF1a:cds-YP_009725295.1:exon1:c.
C1122T:p.H374H -3.3 -0.871087 0.0314961 0.23 T . . .
. . . . orf1ab_ACHNSEVGPEHSL 0.15 . . . .
. . . .
1 1392 1392 C T exonic ORF1a. nonsynonymous SNV

ORF1a:cds-YP_009724389.1:exon1:c.C1127T:p.S376L,ORF1a:cds-YP_009725295.1:exon1:c.
C1127T:p.S376L -2.2 0.667039 0 0.1 T . . . .
. . . orf1ab_ACHNSEVGPEHSL 0.15 . . . . .
. . .
1 1415 1415 C T exonic ORF1a. nonsynonymous SNV
ORF1a:cds-YP_009724389.1:exon1:c.C1150T:p.L384F,ORF1a:cds-YP_009725295.1:exon1:c.
C1150T:p.L384F 0.712 1.47272 1 0.02 D . . . .
. . . orf1ab_ACHNSEVGPEHSL 0.15 . . . . .
. . .
1 1421 1421 G T exonic ORF1a. stopgain
ORF1a:cds-YP_009724389.1:exon1:c.G1156T:p.E386X,ORF1a:cds-YP_009725295.1:exon1:c.
G1156T:p.E386X 1.65 4.256 0.795276 . . . . . .
. . . orf1ab_SEVGPEHSLAEY -1 . . . . .
. . .
1 1441 1441 C T exonic ORF1a. synonymous SNV
ORF1a:cds-YP_009724389.1:exon1:c.C1176T:p.G392G,ORF1a:cds-YP_009725295.1:exon1:c.
C1176T:p.G392G -3.3 0.00784252 0.968504 0.65 T . . .
. . . . orf1ab_AEYHNESGL 0.04 . . . . .
. . .
1 1473 1473 C T exonic ORF1a. nonsynonymous SNV
ORF1a:cds-YP_009724389.1:exon1:c.C1208T:p.T403I,ORF1a:cds-YP_009725295.1:exon1:c.
C1208T:p.T403I -0.348 0.960016 0.976378 0 D . . .
. . . .
.
1 1519 1519 C T exonic ORF1a. synonymous SNV
ORF1a:cds-YP_009724389.1:exon1:c.C1254T:p.N418N,ORF1a:cds-YP_009725295.1:exon1:c.
C1254T:p.N418N -1.07 0.667039 1 1 T . . . .
. . . .

1 1547 1547 A G exonic ORF1a. nonsynonymous SNV
ORF1a:cds-YP_009724389.1:exon1:c.A1282G:p.S428G,ORF1a:cds-YP_009725295.1:exon1:c.
A1282G:p.S428G 0.482 1.17975 1 0.38 T . . . .
. . . .

1 1558 1558 A G exonic ORF1a. nonsynonymous SNV
ORF1a:cds-YP_009724389.1:exon1:c.A1293G:p.I431M,ORF1a:cds-YP_009725295.1:exon1:c.
A1293G:p.I431M -3.3 -1.09082 0.700787 0 D . . .
. . . .
.
1 1599 1599 G T exonic ORF1a. nonsynonymous SNV
ORF1a:cds-YP_009724389.1:exon1:c.G1334T:p.G445V,ORF1a:cds-YP_009725295.1:exon1:c.
G1334T:p.G445V -0.232 0.520551 0.850394 0.19 T . . .
. . . . orf1ab_SEGLNDNLL 0.18 . . . . .

. . .
1 1612 1612 C T exonic ORF1a. synonymous SNV
ORF1a:cds-YP_009724389.1:exon1:c.C1347T:p.N449N,ORF1a:cds-YP_009725295.1:exon1:c.
C1347T:p.N449N -1.83 0.00784252 0.992126 0.52 T . . .
. . . . orf1ab_SEGLNDNLL 0.18 . . . . .
. . .
1 1639 1639 A G exonic ORF1a. synonymous SNV
ORF1a:cds-YP_009724389.1:exon1:c.A1374G:p.K458K,ORF1a:cds-YP_009725295.1:exon1:c.
A1374G:p.K458K -0.876 0.00784252 0.992126 0.48 T . . .
. . . . nCoV-2019_5_RIGHT .
. . . .
1 1642 1642 C T exonic ORF1a. synonymous SNV
ORF1a:cds-YP_009724389.1:exon1:c.C1377T:p.V459V,ORF1a:cds-YP_009725295.1:exon1:c.
C1377T:p.V459V 0.474 0.447307 0.984252 0.42 T . . .
. . . . nCoV-2019_5_RIGHT .
. . . .
1 1686 1686 C T exonic ORF1a. nonsynonymous SNV
ORF1a:cds-YP_009724389.1:exon1:c.C1421T:p.A474V,ORF1a:cds-YP_009725295.1:exon1:c.
C1421T:p.A474V 0.761 1.83894 0.992126 0.15 T . . .
. . orf1ab polyprotein_INIVGDFKLNEEIAI 16 orf1ab_KLNEEIAII 0.05
. . . .
1 1729 1729 G T exonic ORF1a. synonymous SNV
ORF1a:cds-YP_009724389.1:exon1:c.G1464T:p.V488V,ORF1a:cds-YP_009725295.1:exon1:c.
G1464T:p.V488V -2.35 -0.0654016 0.976378 1 T . . .
. . orf1ab polyprotein_ILASFSASTSAFVET 9.4 orf1ab_STSAFVETVK
0.09 . . . .
1 1738 1738 G T exonic ORF1a. synonymous SNV
ORF1a:cds-YP_009724389.1:exon1:c.G1473T:p.V491V,ORF1a:cds-YP_009725295.1:exon1:c.
G1473T:p.V491V -3.3 -0.138646 0.968504 0.84 T . . .
. . . . orf1ab_STSAFVETVK 0.09 . . . .
. . . .
1 1793 1793 A G exonic ORF1a. nonsynonymous SNV
ORF1a:cds-YP_009724389.1:exon1:c.A1528G:p.K510E,ORF1a:cds-YP_009725295.1:exon1:c.
A1528G:p.K510E -0.65 0.447307 0.976378 0.08 T . . .
. . . . orf1ab_KVTKGKAKK 0.14 . . . . .
. . .
1 1849 1849 A T exonic ORF1a. synonymous SNV
ORF1a:cds-YP_009724389.1:exon1:c.A1584T:p.S528S,ORF1a:cds-YP_009725295.1:exon1:c.
A1584T:p.S528S -3.3 -1.53028 0 0.95 T . . . .
. . . orf1ab_GEQKSILSPL 0.13 . . . . . .
. .
1 1864 1864 T C exonic ORF1a. synonymous SNV
ORF1a:cds-YP_009724389.1:exon1:c.T1599C:p.L533L,ORF1a:cds-YP_009725295.1:exon1:c.

T1599C:p.L533L	-0.452	0.960016	1	1	T	.	.	.	.
.	orf1ab polyprotein_SPLYAFASEAARVVR	7.8	orf1ab_GEQKSILSPL 0.13	.
.	.	.	.	.	.	.
1	1884	1884	C	T	exonic	ORF1a.	nonsynonymous SNV
ORF1a:cds-YP_009724389.1:exon1:c.C1619T:p.A540V,ORF1a:cds-YP_009725295.1:exon1:c.
C1619T:p.A540V	1.65	3.30383	1	0.1	T	.	.	.	.
.	orf1ab polyprotein_SPLYAFASEAARVVR	7.8	.	.	.	.
nCoV-2019_7_LEFT_alt0	.	.	.	.	.
1	1889	1889	C	T	exonic	ORF1a.	nonsynonymous SNV
ORF1a:cds-YP_009724389.1:exon1:c.C1624T:p.R542C,ORF1a:cds-YP_009725295.1:exon1:c.
C1624T:p.R542C	-3.12	0.00784252	0.984252	0.04	D	.	.	.
.	.	orf1ab polyprotein_SPLYAFASEAARVVR	7.8	orf1ab_RVVRSIFSR	0.09
.	.	nCoV-2019_7_LEFT_alt0	.	.	.	.	.

1	1895	1895	G	A	exonic	ORF1a.	nonsynonymous SNV
ORF1a:cds-YP_009724389.1:exon1:c.G1630A:p.V544I,ORF1a:cds-YP_009725295.1:exon1:c.
G1630A:p.V544I	0.555	0.886772	0.629921	0.75	T	.	.	.
.	.	orf1ab polyprotein_SPLYAFASEAARVVR	7.8	orf1ab_RVVRSIFSR	0.09
.	.	nCoV-2019_7_LEFT	.	.	.	.	.

1	1914	1914	G	A	exonic	ORF1a.	nonsynonymous SNV
ORF1a:cds-YP_009724389.1:exon1:c.G1649A:p.R550H,ORF1a:cds-YP_009725295.1:exon1:c.
G1649A:p.R550H	1.65	4.256	1	0.01	D	.	.	.	.	.
orf1ab polyprotein_ARVVRSIFSRTLETA	14	orf1ab_RVVRSIFSR	0.09	.	.
.	.	.	.	.	.
1	1919	1919	C	A	exonic	ORF1a.	nonsynonymous SNV
ORF1a:cds-YP_009724389.1:exon1:c.C1654A:p.L552I,ORF1a:cds-YP_009725295.1:exon1:c.
C1654A:p.L552I	0.737	1.7657	0.992126	0.21	T	.	.	.	.
.	orf1ab polyprotein_ARVVRSIFSRTLETA	14	.	.	.	.	.
.	.	.	.	.
1	1927	1927	T	C	exonic	ORF1a.	synonymous SNV
ORF1a:cds-YP_009724389.1:exon1:c.T1662C:p.T554T,ORF1a:cds-YP_009725295.1:exon1:c.
T1662C:p.T554T	0.468	0.374063	1	1	T	.	.	.	.
.	orf1ab polyprotein_ARVVRSIFSRTLETA	14	.	.	.	.	.
.	.	.	.	.
1	1936	1936	T	C	exonic	ORF1a.	synonymous SNV
ORF1a:cds-YP_009724389.1:exon1:c.T1671C:p.N557N,ORF1a:cds-YP_009725295.1:exon1:c.
T1671C:p.N557N	-3.3	-0.0654016	0.622047	0.77	T	.	.	.
.	.	orf1ab polyprotein_QNSVRVLQKAAITIL	20	.	.	.	.
.	.	.	.	.	.
1	1947	1947	T	C	exonic	ORF1a.	nonsynonymous SNV
ORF1a:cds-YP_009724389.1:exon1:c.T1682C:p.V561A,ORF1a:cds-YP_009725295.1:exon1:c.
T1682C:p.V561A	-2.44	-0.285134	0	0.77	T	.	.	.	.

. orf1ab polyprotein_QNSVRVLQKAAITIL 20 . . . .
nCoV-2019_6_RIGHT . . . . .
1 1976 1976 G T exonic ORF1a. nonsynonymous SNV
ORF1a:cds-YP_009724389.1:exon1:c.G1711T:p.D571Y,ORF1a:cds-YP_009725295.1:exon1:c.
G1711T:p.D571Y 0.656 2.20517 0.976378 0.1 T . . .
. . orf1ab polyprotein_AITILDGISQYSLRL 13 orf1ab_TILDGISQY 0.06
. . . .
1 1983 1983 T C exonic ORF1a. nonsynonymous SNV
ORF1a:cds-YP_009724389.1:exon1:c.T1718C:p.I573T,ORF1a:cds-YP_009725295.1:exon1:c.T
1718C:p.I573T 1.65 2.20517 1 0.01 D . . . .
. orf1ab polyprotein_AITILDGISQYSLRL 13 orf1ab_TILDGISQY 0.06 .
. . . .
1 2022 2022 T G exonic ORF1a. nonsynonymous SNV
ORF1a:cds-YP_009724389.1:exon1:c.T1757G:p.F586C,ORF1a:cds-YP_009725295.1:exon1:c.
T1757G:p.F586C -0.777 0.0810866 0.015748 0.01 D . . .
. . orf1ab polyprotein_YSLRLIDAMMFTSDL 7.8 orf1ab_RLIDAMMFT 0.11
. . . .
1 2061 2061 C T exonic ORF1a. nonsynonymous SNV
ORF1a:cds-YP_009724389.1:exon1:c.C1796T:p.A599V,ORF1a:cds-YP_009725295.1:exon1:c.
C1796T:p.A599V 1.65 3.34816 1 0.05 D . . . .
. . . orf1ab_TSDLATNNLVVMAY 0.04 . . . . .
. . .
1 2062 2062 C T exonic ORF1a. synonymous SNV
ORF1a:cds-YP_009724389.1:exon1:c.C1797T:p.A599A,ORF1a:cds-YP_009725295.1:exon1:c.
C1797T:p.A599A -2.44 -0.585827 0.984252 1 T . . .
. . . . orf1ab_TSDLATNNLVVMAY 0.04 . . . .
. . . .
1 2086 2086 G T exonic ORF1a. nonsynonymous SNV
ORF1a:cds-YP_009724389.1:exon1:c.G1821T:p.Q607H,ORF1a:cds-YP_009725295.1:exon1:c.
G1821T:p.Q607H -3.3 -0.131906 0.401575 0.13 T . . .
. . . . orf1ab_YITGGVVQL 0.08 . . . . .
. . .
1 2110 2110 C T exonic ORF1a. synonymous SNV
ORF1a:cds-YP_009724389.1:exon1:c.C1845T:p.N615N,ORF1a:cds-YP_009725295.1:exon1:c.
C1845T:p.N615N -3.3 -1.03975 0 1 T . . . .
. . . orf1ab_NIFGTVYEK -1 . . . .
. .
1 2121 2121 C T exonic ORF1a. nonsynonymous SNV
ORF1a:cds-YP_009724389.1:exon1:c.C1856T:p.T619I,ORF1a:cds-YP_009725295.1:exon1:c.
C1856T:p.T619I 1.65 3.34816 0.984252 0.16 T . . .
. . . . orf1ab_NIFGTVYEK -1 . . . . .
. . .
1 2143 2143 C T exonic ORF1a. synonymous SNV

ORF1a:cds-YP_009724389.1:exon1:c.C1878T:p.P626P,ORF1a:cds-YP_009725295.1:exon1:c.
C1878T:p.P626P -3.3 -0.888441 0 1 T . . . .
. . . orf1ab_YEKLKPVLDW -1 . . . . .
. . .
1 2151 2151 A G exonic ORF1a. nonsynonymous SNV
ORF1a:cds-YP_009724389.1:exon1:c.A1886G:p.D629G,ORF1a:cds-YP_009725295.1:exon1:c
.A1886G:p.D629G -3.3 -0.585827 0 0.24 T . . . .
. . . orf1ab_YEKLKPVLDW -1 . . . . .
. . .
1 2164 2164 G T exonic ORF1a. nonsynonymous SNV
ORF1a:cds-YP_009724389.1:exon1:c.G1899T:p.E633D,ORF1a:cds-YP_009725295.1:exon1:c.
G1899T:p.E633D -3.3 -1.41802 0 0.29 T . . . .
. . . orf1ab_EEKFKEGVEF -1 . . . . .
. . .
1 2197 2197 C T exonic ORF1a. synonymous SNV
ORF1a:cds-YP_009724389.1:exon1:c.C1932T:p.D644D,ORF1a:cds-YP_009725295.1:exon1:c.
C1932T:p.D644D -2.65 -0.131906 0.992126 1 T . . .
. . . . nCoV-2019_8_LEFT . .
. . .
1 2232 2232 C T exonic ORF1a. nonsynonymous SNV
ORF1a:cds-YP_009724389.1:exon1:c.C1967T:p.A656V,ORF1a:cds-YP_009725295.1:exon1:c.
C1967T:p.A656V 0.552 0.700283 0.992126 0.54 T . . .
. . orf1ab polyprotein_WEIVKFISTCACEIV 14 . . . .
. . . . . .
1 2260 2260 C T exonic ORF1a. synonymous SNV
ORF1a:cds-YP_009724389.1:exon1:c.C1995T:p.V665V,ORF1a:cds-YP_009725295.1:exon1:c.
C1995T:p.V665V -2.93 0.246362 0.456693 0.37 T . . .
. . . . nCoV-2019_7_RIGHT_alt5 .
. . . .
1 2325 2325 C T exonic ORF1a. nonsynonymous SNV
ORF1a:cds-YP_009724389.1:exon1:c.C2060T:p.A687V,ORF1a:cds-YP_009725295.1:exon1:c.
C2060T:p.A687V 0.509 0.322016 0.96063 0.09 T . . .
. . orf1ab polyprotein_QTFFKLVNKFLALCA 8.8 orf1ab_KLVNKFLAL -1
. . . .
1 2335 2335 T C exonic ORF1a. synonymous SNV
ORF1a:cds-YP_009724389.1:exon1:c.T2070C:p.A690A,ORF1a:cds-YP_009725295.1:exon1:c.
T2070C:p.A690A -2.22 0.0194016 0.992126 0.19 T . . .
. . orf1ab polyprotein_QTFFKLVNKFLALCA 8.8 . . . .
. . . . . .
1 2385 2385 A C exonic ORF1a. nonsynonymous SNV
ORF1a:cds-YP_009724389.1:exon1:c.A2120C:p.E707A,ORF1a:cds-YP_009725295.1:exon1:c.
A2120C:p.E707A 1.65 2.28901 0.952756 0.21 T . . .
. . orf1ab polyprotein_GETFVTHSKGLYRKC 19 orf1ab_ALNLGETFV 0.16

. . . .
1 2388 2388 C T exonic ORF1a. nonsynonymous SNV
ORF1a:cds-YP_009724389.1:exon1:c.C2123T:p.T708I,ORF1a:cds-YP_009725295.1:exon1:c.
C2123T:p.T708I 0.474 0.473323 0.00787402 0.2 T . . .
. . orf1ab polyprotein_GETFVTHSKGLYRKC 19 orf1ab_ALNLGETFV 0.16
. . . .
1 2395 2395 C T exonic ORF1a. synonymous SNV
ORF1a:cds-YP_009724389.1:exon1:c.C2130T:p.V710V,ORF1a:cds-YP_009725295.1:exon1:c.
C2130T:p.V710V -3.3 -1.03975 0.0314961 0.48 T . . .
. . orf1ab polyprotein_GETFVTHSKGLYRKC 19 orf1ab_ALNLGETFV 0.16
. . . .
1 2401 2401 C T exonic ORF1a. synonymous SNV
ORF1a:cds-YP_009724389.1:exon1:c.C2136T:p.H712H,ORF1a:cds-YP_009725295.1:exon1:c.
C2136T:p.H712H -2.08 -0.207559 0.952756 0.58 T . . .
. . orf1ab polyprotein_GETFVTHSKGLYRKC 19 . . . .
. . . . . .
1 2430 2430 A G exonic ORF1a. nonsynonymous SNV
ORF1a:cds-YP_009724389.1:exon1:c.A2165G:p.K722R,ORF1a:cds-YP_009725295.1:exon1:c.
A2165G:p.K722R 0.737 2.59162 1 0.74 T . . . .
. . . .

1 2434 2434 C T exonic ORF1a. synonymous SNV
ORF1a:cds-YP_009724389.1:exon1:c.C2169T:p.S723S,ORF1a:cds-YP_009725295.1:exon1:c.
C2169T:p.S723S -2.3 0.246362 0.992126 0.87 T . . .
. . . .
.
1 2447 2447 G T exonic ORF1a. nonsynonymous SNV
ORF1a:cds-YP_009724389.1:exon1:c.G2182T:p.G728C,ORF1a:cds-YP_009725295.1:exon1:c.
G2182T:p.G728C 0.746 1.0029 0.976378 0.04 D . . . .
. . . .

1 2455 2455 C T exonic ORF1a. synonymous SNV
ORF1a:cds-YP_009724389.1:exon1:c.C2190T:p.L730L,ORF1a:cds-YP_009725295.1:exon1:c.
C2190T:p.L730L -3.3 -0.283213 0.984252 0.91 T . . .
. . . . orf1ab_MPLKAPKEI 0.1 . . . . .
. . .
1 2509 2509 C T exonic ORF1a. synonymous SNV
ORF1a:cds-YP_009724389.1:exon1:c.C2244T:p.P748P,ORF1a:cds-YP_009725295.1:exon1:c.
C2244T:p.P748P -3.3 -0.358866 0.905512 0.38 T . . .
. . . . orf1ab_GETLPTEVL 0.01 . .
nCoV-2019_9_LEFT_alt4 . . . . .
1 2525 2525 G A exonic ORF1a. nonsynonymous SNV
ORF1a:cds-YP_009724389.1:exon1:c.G2260A:p.E754K,ORF1a:cds-YP_009725295.1:exon1:c.

G2260A:p.E754K 1.65 4.256 0.984252 0.22 T . . . .
. . . . nCoV-2019_9_LEFT_alt4 . .
. . .
1 2536 2536 C T exonic ORF1a. synonymous SNV
ORF1a:cds-YP_009724389.1:exon1:c.C2271T:p.V757V,ORF1a:cds-YP_009725295.1:exon1:c.
C2271T:p.V757V -3.3 -0.056252 0.929134 0.08 T . . .
. . . .
.
1 2581 2581 A C exonic ORF1a. nonsynonymous SNV
ORF1a:cds-YP_009724389.1:exon1:c.A2316C:p.E772D,ORF1a:cds-YP_009725295.1:exon1:c.
A2316C:p.E772D -2.31 -0.358866 0.984252 0.94 T . . .
. . . . nCoV-2019_8_RIGHT .
. . . .
1 2593 2593 T A exonic ORF1a. synonymous SNV
ORF1a:cds-YP_009724389.1:exon1:c.T2328A:p.A776A,ORF1a:cds-YP_009725295.1:exon1:c.
T2328A:p.A776A -3.3 -2.02324 0.984252 1 T . . .
. . . .
.
1 2638 2638 C T exonic ORF1a. synonymous SNV
ORF1a:cds-YP_009724389.1:exon1:c.C2373T:p.L791L,ORF1a:cds-YP_009725295.1:exon1:c.
C2373T:p.L791L -3.3 -1.34236 0.574803 1 T . . .
. . . . orf1ab_LEIKDTEKY -1 . . . . .
. . .
1 2675 2675 C T exonic ORF1a. nonsynonymous SNV
ORF1a:cds-YP_009724389.1:exon1:c.C2410T:p.P804S,ORF1a:cds-YP_009725295.1:exon1:c.
C2410T:p.P804S 1.65 3.34816 0.88189 0.31 T . . .
. . . .
.
1 2686 2686 G C exonic ORF1a. nonsynonymous SNV
ORF1a:cds-YP_009724389.1:exon1:c.G2421C:p.M807I,ORF1a:cds-YP_009725295.1:exon1:c.
G2421C:p.M807I -3.3 -0.207559 0.433071 0.35 T . . .
. . . . orf1ab_VTNNTFTLK -1 . . . . .
. . .
1 2710 2710 C T exonic ORF1a. synonymous SNV
ORF1a:cds-YP_009724389.1:exon1:c.C2445T:p.L815L,ORF1a:cds-YP_009725295.1:exon1:c.
C2445T:p.L815L -3.3 -1.03975 0.425197 1 T . . .
. . . . orf1ab_VTNNTFTLK -1 . . . . .
. . .
1 2720 2720 G A exonic ORF1a. nonsynonymous SNV
ORF1a:cds-YP_009724389.1:exon1:c.G2455A:p.A819T,ORF1a:cds-YP_009725295.1:exon1:c.
G2455A:p.A819T -1.22 1.22986 1 0.02 D . . . .
. . . orf1ab_TLKGGAPTK 0.04 . . . . . .
. .

1 2755 2755 G T exonic ORF1a. synonymous SNV
ORF1a:cds-YP_009724389.1:exon1:c.G2490T:p.V830V,ORF1a:cds-YP_009725295.1:exon1:c.
G2490T:p.V830V -0.831 0.0950551 0.267717 1 T . . .
. . . . orf1ab_TVIEVQGYK -1 . . . . .
. . .
1 2779 2779 T C exonic ORF1a. synonymous SNV
ORF1a:cds-YP_009724389.1:exon1:c.T2514C:p.S838S,ORF1a:cds-YP_009725295.1:exon1:c.
T2514C:p.S838S 1.65 2.21335 1 0.85 T . . . .
. . . orf1ab_IEVQGYKSV 0.11 . . . . . .
. .
1 2782 2782 G T exonic ORF1a. synonymous SNV
ORF1a:cds-YP_009724389.1:exon1:c.G2517T:p.V839V,ORF1a:cds-YP_009725295.1:exon1:c.
G2517T:p.V839V -3.19 0.548976 1 1 T . . . .
. . . orf1ab_IEVQGYKSV 0.11 . . . . . .
. .
1 2836 2836 C T exonic ORF1a. synonymous SNV
ORF1a:cds-YP_009724389.1:exon1:c.C2571T:p.C857C,ORF1a:cds-YP_009725295.1:exon1:c.
C2571T:p.C857C -3.3 -0.510173 0.937008 0.17 T . . .
. . . . nCoV-2019_10_LEFT . .
. . .
1 2910 2910 C T exonic ORF1a. nonsynonymous SNV
ORF1a:cds-YP_009724389.1:exon1:c.C2645T:p.T882I,ORF1a:cds-YP_009725295.1:exon1:c.
C2645T:p.T882I 1.65 3.34816 1 0.14 T . . . .
. . . .

1 2945 2945 G T exonic ORF1a. nonsynonymous SNV
ORF1a:cds-YP_009724389.1:exon1:c.G2680T:p.G894C,ORF1a:cds-YP_009725295.1:exon1:c.
G2680T:p.G894C 1.65 4.256 1 0.03 D . . . . . .
. . orf1ab_SELLTPLGI 0.05 . . . .
.
1 2973 2973 C T exonic ORF1a. nonsynonymous SNV
ORF1a:cds-YP_009724389.1:exon1:c.C2708T:p.A903V,ORF1a:cds-YP_009725295.1:exon1:c.
C2708T:p.A903V 1.65 3.34816 1 0.26 T . . . .
. . . orf1ab_DEWSMATYY -1 . . . . . .
. . .
1 3037 3037 C T exonic ORF1a. synonymous SNV
ORF1a:cds-YP_009724389.1:exon1:c.C2772T:p.F924F,ORF1a:cds-YP_009725295.1:exon1:c.
C2772T:p.F924F -3.3 -2.0989 0.00787402 0.66 T . . .
. . . .
hypermutable low-fitness site
1 3045 3045 C T exonic ORF1a. nonsynonymous SNV
ORF1a:cds-YP_009724389.1:exon1:c.C2780T:p.P927L,ORF1a:cds-YP_009725295.1:exon1:c.
C2780T:p.P927L 1.65 3.34816 0.992126 0.22 T . . .

. . . .
.
1 3085 3085 G T exonic ORF1a. nonsynonymous SNV
ORF1a:cds-YP_009724389.1:exon1:c.G2820T:p.E940D,ORF1a:cds-YP_009725295.1:exon1:c.
G2820T:p.E940D -2.35 -0.659772 0.00787402 0.08 T . . .
. . . . orf1ab_GDCEEEEFEPSTQY -1 . . . .
. . . .
1 3096 3096 C T exonic ORF1a. nonsynonymous SNV
ORF1a:cds-YP_009724389.1:exon1:c.C2831T:p.S944L,ORF1a:cds-YP_009725295.1:exon1:c.
C2831T:p.S944L 1.65 0.171063 0.992126 0.25 T . . .
. . . . orf1ab_GDCEEEEFEPSTQY -1 . . . .
. . . .
1 3104 3104 T C exonic ORF1a. nonsynonymous SNV
ORF1a:cds-YP_009724389.1:exon1:c.T2839C:p.Y947H,ORF1a:cds-YP_009725295.1:exon1:c.
T2839C:p.Y947H -0.381 0.517244 0.976378 0.55 T . . .
. . . . orf1ab_GDCEEEEFEPSTQY -1 . . . .
. . . .
1 3140 3140 C T exonic ORF1a. nonsynonymous SNV
ORF1a:cds-YP_009724389.1:exon1:c.C2875T:p.P959S,ORF1a:cds-YP_009725295.1:exon1:c.
C2875T:p.P959S -0.127 1.20961 0.84252 0 D . . .
. . . . orf1ab_DYQGKPLEF -1 . . . . .
. . .
1 3157 3157 C T exonic ORF1a. synonymous SNV
ORF1a:cds-YP_009724389.1:exon1:c.C2892T:p.A964A,ORF1a:cds-YP_009725295.1:exon1:c.
C2892T:p.A964A -3.3 -1.14443 0.19685 0.78 T . . .
. . . . orf1ab_KPLEFGATSAAL -1 . .
nCoV-2019_11_LEFT . . . . .
1 3176 3176 C T exonic ORF1a. nonsynonymous SNV
ORF1a:cds-YP_009724389.1:exon1:c.C2911T:p.P971S,ORF1a:cds-YP_009725295.1:exon1:c.
C2911T:p.P971S -0.268 0.309535 0.0314961 0.4 T . . .
. . . .
.
1 3177 3177 C T exonic ORF1a. nonsynonymous SNV
ORF1a:cds-YP_009724389.1:exon1:c.C2912T:p.P971L,ORF1a:cds-YP_009725295.1:exon1:c.
C2912T:p.P971L -2.65 0.0325905 0.0866142 0.2 T . . .
. . . .
.
1 3181 3181 A C exonic ORF1a. nonsynonymous SNV
ORF1a:cds-YP_009724389.1:exon1:c.A2916C:p.E972D,ORF1a:cds-YP_009725295.1:exon1:c.
A2916C:p.E972D -2.98 -1.90602 0.811024 0.45 T . . .
. . . .
.
1 3206 3206 G T exonic ORF1a. nonsynonymous SNV

ORF1a:cds-YP_009724389.1:exon1:c.G2941T:p.D981Y,ORF1a:cds-YP_009725295.1:exon1:c.
G2941T:p.D981Y 1.33 2.59433 0.92126 0.03 D . . .
. . . . nCoV-2019_10_RIGHT .
. . . .
1 3251 3251 G T exonic ORF1a. nonsynonymous SNV
ORF1a:cds-YP_009724389.1:exon1:c.G2986T:p.D996Y,ORF1a:cds-YP_009725295.1:exon1:c.
G2986T:p.D996Y . 0.240299 0 0.01 D . . . .
. . . orf1ab_SEDNQTTTI 0.09 . . . . . .
. .
1 3256 3256 T C exonic ORF1a. synonymous SNV
ORF1a:cds-YP_009724389.1:exon1:c.T2991C:p.N997N,ORF1a:cds-YP_009725295.1:exon1:c.
T2991C:p.N997N . 0.101827 0.015748 0.49 T . . .
. . . . orf1ab_SEDNQTTTI 0.09 . . . . .
. . .
1 3259 3259 G A exonic ORF1a. synonymous SNV
ORF1a:cds-YP_009724389.1:exon1:c.G2994A:p.Q998Q,ORF1a:cds-YP_009725295.1:exon1:c
.G2994A:p.Q998Q . 0.240299 0.015748 0.57 T . . .
. . . . orf1ab_SEDNQTTTI 0.09 . . . . .
. . .
1 3259 3259 G T exonic ORF1a. nonsynonymous SNV
ORF1a:cds-YP_009724389.1:exon1:c.G2994T:p.Q998H,ORF1a:cds-YP_009725295.1:exon1:c.
G2994T:p.Q998H . 0.240299 0.015748 0.17 T . . .
. . . . orf1ab_SEDNQTTTI 0.09 . . . . .
. . .
1 3261 3261 C T exonic ORF1a. nonsynonymous SNV
ORF1a:cds-YP_009724389.1:exon1:c.C2996T:p.T999I,ORF1a:cds-YP_009725295.1:exon1:c.
C2996T:p.T999I . 0.171063 0.015748 0.24 T . . .
. . . . orf1ab_SEDNQTTTI 0.09 . . . . .
. . .
1 3267 3267 C T exonic ORF1a. nonsynonymous SNV
ORF1a:cds-YP_009724389.1:exon1:c.C3002T:p.T1001I,ORF1a:cds-YP_009725295.1:exon1:c.
C3002T:p.T1001I . -1.14443 0 0.03 D . . . .
. . . orf1ab_SEDNQTTTI 0.09 . . . . . .
. .
1 3277 3277 A G exonic ORF1a. synonymous SNV
ORF1a:cds-YP_009724389.1:exon1:c.A3012G:p.T1004T,ORF1a:cds-YP_009725295.1:exon1:
c.A3012G:p.T1004T . 0.171063 0.015748 0.53 T . . .
. . . .
.
1 3315 3315 C T exonic ORF1a. nonsynonymous SNV
ORF1a:cds-YP_009724389.1:exon1:c.C3050T:p.T1017I,ORF1a:cds-YP_009725295.1:exon1:c.
C3050T:p.T1017I -1.9 -0.0366457 0 0.03 D . . . .
. . . orf1ab_LEMELTPVVQTI 0.1 . . . . .

. . .
1 3328 3328 G T exonic ORF1a. nonsynonymous SNV
ORF1a:cds-YP_009724389.1:exon1:c.G3063T:p.Q1021H,ORF1a:cds-YP_009725295.1:exon1:
c.G3063T:p.Q1021H -0.308 0.171063 0.795276 0.04 D . . .
. . . . orf1ab_LEMELTPVVQTI 0.1 . . . .
. . . .
1 3330 3330 C T exonic ORF1a. nonsynonymous SNV
ORF1a:cds-YP_009724389.1:exon1:c.C3065T:p.T1022I,ORF1a:cds-YP_009725295.1:exon1:c.
C3065T:p.T1022I 0.154 0.655717 0.866142 0.13 T . . .
. . . . orf1ab_LEMELTPVVQTI 0.1 . . . .
. . . .
1 3340 3340 G T exonic ORF1a. synonymous SNV
ORF1a:cds-YP_009724389.1:exon1:c.G3075T:p.V1025V,ORF1a:cds-YP_009725295.1:exon1:
c.G3075T:p.V1025V -2.04 -1.2829 0.141732 0.61 T . Macro .
. . orf1ab polyprotein_NSFSGYLKLTDNVYI 14 orf1ab_IEVNSFSGY -1
. . . .
1 3431 3431 G T exonic ORF1a. nonsynonymous SNV
ORF1a:cds-YP_009724389.1:exon1:c.G3166T:p.V1056L,ORF1a:cds-YP_009725295.1:exon1:c
.G3166T:p.V1056L 0.737 2.7328 0.968504 0.03 D . Macro . .
. orf1ab polyprotein_KVKPTVVVNAANVYL 17 . . . . .
. . . . .
1 3442 3442 T C exonic ORF1a. synonymous SNV
ORF1a:cds-YP_009724389.1:exon1:c.T3177C:p.N1059N,ORF1a:cds-YP_009725295.1:exon1:
c.T3177C:p.N1059N -2.11 0.0325905 0.992126 1 T . Macro .
. . orf1ab polyprotein_KVKPTVVVNAANVYL 17 orf1ab_VVNAANVYLK
0.06 . . . .
1 3457 3457 C T exonic ORF1a. synonymous SNV
ORF1a:cds-YP_009724389.1:exon1:c.C3192T:p.Y1064Y,ORF1a:cds-YP_009725295.1:exon1:
c.C3192T:p.Y1064Y -3.3 0.101827 0.984252 0.12 T . Macro .
. . orf1ab polyprotein_KVKPTVVVNAANVYL 17 orf1ab_VVNAANVYLK
0.06 . . . .
1 3485 3485 G A exonic ORF1a. nonsynonymous SNV
ORF1a:cds-YP_009724389.1:exon1:c.G3220A:p.A1074T,ORF1a:cds-YP_009725295.1:exon1:
c.G3220A:p.A1074T 1.65 4.256 1 0.01 D . Macro . . .
. . . .

1 3499 3499 T C exonic ORF1a. synonymous SNV
ORF1a:cds-YP_009724389.1:exon1:c.T3234C:p.A1078A,ORF1a:cds-YP_009725295.1:exon1:
c.T3234C:p.A1078A -3.3 0.0325905 0.992126 1 T . Macro .
. . . .
.
1 3542 3542 A G exonic ORF1a. nonsynonymous SNV
ORF1a:cds-YP_009724389.1:exon1:c.A3277G:p.T1093A,ORF1a:cds-YP_009725295.1:exon1:

c.A3277G:p.T1093A -0.4 0.240299 0.015748 0.79 T . Macro .
. . . .
.
1 3583 3583 C T exonic ORF1a. synonymous SNV
ORF1a:cds-YP_009724389.1:exon1:c.C3318T:p.S1106S,ORF1a:cds-YP_009725295.1:exon1:
c.C3318T:p.S1106S 0.404 0.171063 0.992126 0.8 T . Macro .
. . . . orf1ab_VLSGHNLAK 0.02 . . . . .
. . .
1 3587 3587 C T exonic ORF1a. nonsynonymous SNV
ORF1a:cds-YP_009724389.1:exon1:c.C3322T:p.H1108Y,ORF1a:cds-YP_009725295.1:exon1:
c.C3322T:p.H1108Y 0.771 1.9712 0.992126 0.1 T . Macro . .
. . . orf1ab_VLSGHNLAK 0.02 . . . . . .
. .
1 3619 3619 C T exonic ORF1a. synonymous SNV
ORF1a:cds-YP_009724389.1:exon1:c.C3354T:p.V1118V,ORF1a:cds-YP_009725295.1:exon1:
c.C3354T:p.V1118V 1.65 0.794189 1 1 T . Macro . .
. . . orf1ab_HVVGPNVNK -1 . . . . . .
. .
1 3620 3620 G T exonic ORF1a. nonsynonymous SNV
ORF1a:cds-YP_009724389.1:exon1:c.G3355T:p.G1119C,ORF1a:cds-YP_009725295.1:exon1:
c.G3355T:p.G1119C 1.65 4.256 1 0 D . Macro . . .
. . orf1ab_HVVGPNVNK -1 . . . .
.
1 3629 3629 G A exonic ORF1a. nonsynonymous SNV
ORF1a:cds-YP_009724389.1:exon1:c.G3364A:p.V1122I,ORF1a:cds-YP_009725295.1:exon1:c
.G3364A:p.V1122I -1.4 0.240299 0.992126 0.42 T . Macro .
. . . . orf1ab_HVVGPNVNK -1 . . . . .
. . .
1 3634 3634 C T exonic ORF1a. synonymous SNV
ORF1a:cds-YP_009724389.1:exon1:c.C3369T:p.N1123N,ORF1a:cds-YP_009725295.1:exon1:
c.C3369T:p.N1123N -0.764 0.240299 1 0.4 T . Macro . .
. . . orf1ab_HVVGPNVNK -1 . . . . . .
. .
1 3646 3646 C T exonic ORF1a. synonymous SNV
ORF1a:cds-YP_009724389.1:exon1:c.C3381T:p.D1127D,ORF1a:cds-YP_009725295.1:exon1:
c.C3381T:p.D1127D -3.3 -1.35213 0.433071 1 T . Macro .
. . . .
.
1 3648 3648 T G exonic ORF1a. nonsynonymous SNV
ORF1a:cds-YP_009724389.1:exon1:c.T3383G:p.I1128S,ORF1a:cds-YP_009725295.1:exon1:c.
T3383G:p.I1128S 1.65 2.17891 0.771654 0.02 D . Macro .
. . . .
.

1 3688 3688 C T exonic ORF1a. synonymous SNV
ORF1a:cds-YP_009724389.1:exon1:c.C3423T:p.H1141H,ORF1a:cds-YP_009725295.1:exon1:
c.C3423T:p.H1141H -0.519 0.724953 1 0.55 T . Macro . .
. . . orf1ab_YENFNQHEVL 0.04 . . . . .
. . .
1 3692 3692 G T exonic ORF1a. nonsynonymous SNV
ORF1a:cds-YP_009724389.1:exon1:c.G3427T:p.V1143F,ORF1a:cds-YP_009725295.1:exon1:
c.G3427T:p.V1143F -0.583 1.27884 0.015748 0.05 D . Macro .
. . . . orf1ab_YENFNQHEVL 0.04 . . . .
. . . .
1 3729 3729 G A exonic ORF1a. nonsynonymous SNV
ORF1a:cds-YP_009724389.1:exon1:c.G3464A:p.G1155D,ORF1a:cds-YP_009725295.1:exon1:
c.G3464A:p.G1155D 1.65 4.256 1 0.04 D . Macro . . .
orf1ab polyprotein_AGIFGADPIHSLRVC 15 . . . . . .
. . . .
1 3732 3732 C A exonic ORF1a. nonsynonymous SNV
ORF1a:cds-YP_009724389.1:exon1:c.C3467A:p.A1156D,ORF1a:cds-YP_009725295.1:exon1:
c.C3467A:p.A1156D -0.356 0.932661 0.992126 0.05 D . Macro .
. . orf1ab polyprotein_AGIFGADPIHSLRVC 15 . . . .
. . . . . .
1 3737 3737 C T exonic ORF1a. nonsynonymous SNV
ORF1a:cds-YP_009724389.1:exon1:c.C3472T:p.P1158S,ORF1a:cds-YP_009725295.1:exon1:
c.C3472T:p.P1158S 1.65 3.28669 0.992126 0.1 T . Macro .
. . orf1ab polyprotein_AGIFGADPIHSLRVC 15 . . . .
. . . . . .
1 3768 3768 C T exonic ORF1a. nonsynonymous SNV
ORF1a:cds-YP_009724389.1:exon1:c.C3503T:p.T1168I,ORF1a:cds-YP_009725295.1:exon1:c.
C3503T:p.T1168I -1.65 0.171063 0 0.2 T . Macro . .
. . . .

1 3774 3774 G A exonic ORF1a. nonsynonymous SNV
ORF1a:cds-YP_009724389.1:exon1:c.G3509A:p.R1170H,ORF1a:cds-YP_009725295.1:exon1:
c.G3509A:p.R1170H -1.61 1.14037 0.992126 0.13 T . Macro .
. . . . nCoV-2019_13_LEFT. .
. . .
1 3817 3817 C T exonic ORF1a. synonymous SNV
ORF1a:cds-YP_009724389.1:exon1:c.C3552T:p.D1184D,ORF1a:cds-YP_009725295.1:exon1:
c.C3552T:p.D1184D -3.3 -0.105882 0.992126 0.65 T . Macro .
. . . . orf1ab_AVFDKNLYDK -1 . . . .
. . . .
1 3871 3871 G T exonic ORF1a. nonsynonymous SNV
ORF1a:cds-YP_009724389.1:exon1:c.G3606T:p.K1202N,ORF1a:cds-YP_009725295.1:exon1:
c.G3606T:p.K1202N -3.3 3.28669 0.992126 0.24 T . . .

. . . . orf1ab_SEKQVEQKI -1 . . . . .
. . .
1 3882 3882 T C exonic ORF1a. nonsynonymous SNV
ORF1a:cds-YP_009724389.1:exon1:c.T3617C:p.I1206T,ORF1a:cds-YP_009725295.1:exon1:c.
T3617C:p.I1206T -1.15 -0.86748 0 0.1 T . . . .
. . . orf1ab_AEIPKEEVKPF -1 . . . . .
. . .
1 3892 3892 G T exonic ORF1a. nonsynonymous SNV
ORF1a:cds-YP_009724389.1:exon1:c.G3627T:p.E1209D,ORF1a:cds-YP_009725295.1:exon1:
c.G3627T:p.E1209D -2.97 0.58648 0.314961 0.5 T . . .
. . . . orf1ab_AEIPKEEVKPF -1 . . . .
. . . .
1 3947 3947 G T exonic ORF1a. nonsynonymous SNV
ORF1a:cds-YP_009724389.1:exon1:c.G3682T:p.D1228Y,ORF1a:cds-YP_009725295.1:exon1:
c.G3682T:p.D1228Y 0.646 2.31739 0.685039 0.07 T . . .
. . . .
.
1 3955 3955 G T exonic ORF1a. nonsynonymous SNV
ORF1a:cds-YP_009724389.1:exon1:c.G3690T:p.K1230N,ORF1a:cds-YP_009725295.1:exon1:
c.G3690T:p.K1230N -3.22 0.171063 0.811024 0.44 T . . .
. . . .
.
1 3969 3969 G A exonic ORF1a. nonsynonymous SNV
ORF1a:cds-YP_009724389.1:exon1:c.G3704A:p.C1235Y,ORF1a:cds-YP_009725295.1:exon1:
c.G3704A:p.C1235Y -0.321 1.7635 0.937008 0.06 T . . . .
. . . .

1 4012 4012 C T exonic ORF1a. synonymous SNV
ORF1a:cds-YP_009724389.1:exon1:c.C3747T:p.L1249L,ORF1a:cds-YP_009725295.1:exon1:c
.C3747T:p.L1249L -2.52 0.0325905 0.992126 0.81 T . . .
. . . . orf1ab_LTENLLLY 0.05 . . . . .
. . .
1 4036 4036 T A exonic ORF1a. synonymous SNV
ORF1a:cds-YP_009724389.1:exon1:c.T3771A:p.I1257I,ORF1a:cds-YP_009725295.1:exon1:c.
T3771A:p.I1257I -2.44 0.0325905 0.992126 0.5 T . . .
. . orf1ab polyprotein_ENLLLYIDINGNLHP 7.9 orf1ab_LYIDINGNL 0.08
. . . .
1 4077 4077 T C exonic ORF1a. nonsynonymous SNV
ORF1a:cds-YP_009724389.1:exon1:c.T3812C:p.V1271A,ORF1a:cds-YP_009725295.1:exon1:
c.T3812C:p.V1271A 1.65 2.17891 0.669291 0.05 D . . .
. . . . orf1ab_LVSDIDITFLK-1 . . nCoV-2019_14_LEFT
. . . . .
1 4105 4105 G T exonic ORF1a. nonsynonymous SNV

ORF1a:cds-YP_009724389.1:exon1:c.G3840T:p.K1280N,ORF1a:cds-YP_009725295.1:exon1:
c.G3840T:p.K1280N -1.72 0.516961 0.992126 0.02 D . . .
. . orf1ab polyprotein_ITFLKKDAPYIVGDV 8.2 orf1ab_LVSDIDITFLK-1
. . . .
1 4144 4144 G A exonic ORF1a. synonymous SNV
ORF1a:cds-YP_009724389.1:exon1:c.G3879A:p.E1293E,ORF1a:cds-YP_009725295.1:exon1:
c.G3879A:p.E1293E -2.53 -0.0520236 0.937008 1 T . . .
. . . . nCoV-2019_13_RIGHT .
. . . .
1 4189 4189 C T exonic ORF1a. synonymous SNV
ORF1a:cds-YP_009724389.1:exon1:c.C3924T:p.G1308G,ORF1a:cds-YP_009725295.1:exon1:
c.C3924T:p.G1308G -3.3 -0.864858 0.944882 1 T . . .
. . . .
.
1 4240 4240 T C exonic ORF1a. synonymous SNV
ORF1a:cds-YP_009724389.1:exon1:c.T3975C:p.Y1325Y,ORF1a:cds-YP_009725295.1:exon1:
c.T3975C:p.Y1325Y -0.834 0.354394 0.992126 0.56 T . . .
. . . . orf1ab_ALRKVPTDNYITTY 0.04 . . . .
. . . .
1 4255 4255 G A exonic ORF1a. synonymous SNV
ORF1a:cds-YP_009724389.1:exon1:c.G3990A:p.P1330P,ORF1a:cds-YP_009725295.1:exon1:
c.G3990A:p.P1330P -3.3 -1.43384 0.889764 0.97 T . . .
. . . . orf1ab_YPGQGLNGY0.01 . . . . .
. . .
1 4276 4276 C T exonic ORF1a. synonymous SNV
ORF1a:cds-YP_009724389.1:exon1:c.C4011T:p.Y1337Y,ORF1a:cds-YP_009725295.1:exon1:
c.C4011T:p.Y1337Y -1.92 0.0292598 0.992126 1 T . . .
. . . . orf1ab_YPGQGLNGY0.01 . . . . .
. . .
1 4300 4300 G T exonic ORF1a. synonymous SNV
ORF1a:cds-YP_009724389.1:exon1:c.G4035T:p.V1345V,ORF1a:cds-YP_009725295.1:exon1:
c.G4035T:p.V1345V -3.3 0.110543 0.992126 0.84 T . . .
. . . . nCoV-2019_15_LEFT. .
. . .
1 4320 4320 C T exonic ORF1a. nonsynonymous SNV
ORF1a:cds-YP_009724389.1:exon1:c.C4055T:p.A1352V,ORF1a:cds-YP_009725295.1:exon1:
c.C4055T:p.A1352V -2.31 0.27311 0.440945 0.4 T . . .
. . orf1ab polyprotein_SAFYILPSIISNEKQ 14 . . . .
nCoV-2019_15_LEFT. . . . .
1 4345 4345 C A exonic ORF1a. synonymous SNV
ORF1a:cds-YP_009724389.1:exon1:c.C4080A:p.I1360I,ORF1a:cds-YP_009725295.1:exon1:c.
C4080A:p.I1360I -0.736 0.0292598 0.291339 0.3 T . . .
. . orf1ab polyprotein_SAFYILPSIISNEKQ 14 orf1ab_SNEKQEILGTVSW

-1 . . . .
1 4354 4354 G A exonic ORF1a. synonymous SNV
ORF1a:cds-YP_009724389.1:exon1:c.G4089A:p.E1363E,ORF1a:cds-YP_009725295.1:exon1:
c.G4089A:p.E1363E -3.3 -0.621008 0.448819 1 T . . .
. . orf1ab polyprotein_SAFYILPSIISNEKQ 14 orf1ab_SNEKQEILGTVSW
-1 . . . .
1 4364 4364 A G exonic ORF1a. nonsynonymous SNV
ORF1a:cds-YP_009724389.1:exon1:c.A4099G:p.I1367V,ORF1a:cds-YP_009725295.1:exon1:c
.A4099G:p.I1367V -3.3 -0.133307 0.984252 0.43 T . . .
. . orf1ab polyprotein_EILGTVSWNLREMLA 17 orf1ab_SNEKQEILGTVSW
-1 . . . .
1 4372 4372 A G exonic ORF1a. synonymous SNV
ORF1a:cds-YP_009724389.1:exon1:c.A4107G:p.G1369G,ORF1a:cds-YP_009725295.1:exon1:
c.A4107G:p.G1369G -3.3 -0.295874 0.992126 1 T . . .
. . orf1ab polyprotein_EILGTVSWNLREMLA 17 orf1ab_SNEKQEILGTVSW
-1 . . . .
1 4397 4397 A C exonic ORF1a. nonsynonymous SNV
ORF1a:cds-YP_009724389.1:exon1:c.A4132C:p.M1378L,ORF1a:cds-YP_009725295.1:exon1:
c.A4132C:p.M1378L 1.65 2.22391 0.992126 0 D . . .
. . orf1ab polyprotein_EILGTVSWNLREMLA 17 . . . .
. . . . . .
1 4423 4423 C T exonic ORF1a. synonymous SNV
ORF1a:cds-YP_009724389.1:exon1:c.C4158T:p.R1386R,ORF1a:cds-YP_009725295.1:exon1:
c.C4158T:p.R1386R -0.741 -0.0520236 0.992126 0.38 T . . .
. . . . . orf1ab_KLMPVCVETK 0.09 . .
nCoV-2019_14_RIGHT_alt2 . . . . . .
1 4455 4455 C T exonic ORF1a. nonsynonymous SNV
ORF1a:cds-YP_009724389.1:exon1:c.C4190T:p.A1397V,ORF1a:cds-YP_009725295.1:exon1:
c.C4190T:p.A1397V 1.65 3.2806 0.952756 0.01 D . . . .
. . . orf1ab_AIVSTIQRK -1 . . . . . .
. .
1 4467 4467 C T exonic ORF1a. nonsynonymous SNV
ORF1a:cds-YP_009724389.1:exon1:c.C4202T:p.T1401I,ORF1a:cds-YP_009725295.1:exon1:c.
C4202T:p.T1401I 1.65 3.2806 0.984252 0.11 T . . . .
. . . orf1ab_AIVSTIQRK -1 . . . . . .
. .
1 4475 4475 C T exonic ORF1a. nonsynonymous SNV
ORF1a:cds-YP_009724389.1:exon1:c.C4210T:p.R1404C,ORF1a:cds-YP_009725295.1:exon1:
c.C4210T:p.R1404C 1.65 3.2806 1 0.02 D . . . . .
. . orf1ab_AIVSTIQRK -1 . . . .
.
1 4510 4510 G A exonic ORF1a. synonymous SNV
ORF1a:cds-YP_009724389.1:exon1:c.G4245A:p.V1415V,ORF1a:cds-YP_009725295.1:exon1:

c.G4245A:p.V1415V -3.3 -4.19748 0 1 T . . . .
. . . orf1ab_QEGVVDYGARF -1 . . . . .
. . .
1 4528 4528 A G exonic ORF1a. synonymous SNV
ORF1a:cds-YP_009724389.1:exon1:c.A4263G:p.R1421R,ORF1a:cds-YP_009725295.1:exon1:
c.A4263G:p.R1421R -3.3 -0.133307 0.992126 1 T . . .
. . orf1ab polyprotein_RFYFYTSKTTVASLI 18 orf1ab_QEGVVDYGARF
-1 . . . .
1 4540 4540 C T exonic ORF1a. synonymous SNV
ORF1a:cds-YP_009724389.1:exon1:c.C4275T:p.Y1425Y,ORF1a:cds-YP_009725295.1:exon1:
c.C4275T:p.Y1425Y -3.3 -0.133307 0.992126 0.36 T . . .
. . orf1ab polyprotein_RFYFYTSKTTVASLI 18 . . . .
. . . . . .
1 4543 4543 C T exonic ORF1a. synonymous SNV
ORF1a:cds-YP_009724389.1:exon1:c.C4278T:p.T1426T,ORF1a:cds-YP_009725295.1:exon1:c
.C4278T:p.T1426T -0.944 0.354394 1 1 T . . . .
. orf1ab polyprotein_RFYFYTSKTTVASLI 18 . . . . .
. . . . .
1 4547 4547 A G exonic ORF1a. nonsynonymous SNV
ORF1a:cds-YP_009724389.1:exon1:c.A4282G:p.K1428E,ORF1a:cds-YP_009725295.1:exon1:
c.A4282G:p.K1428E 1.65 2.22391 0.992126 0.07 T . . .
. . orf1ab polyprotein_RFYFYTSKTTVASLI 18 . . . .
. . . . . .
1 4560 4560 C T exonic ORF1a. nonsynonymous SNV
ORF1a:cds-YP_009724389.1:exon1:c.C4295T:p.A1432V,ORF1a:cds-YP_009725295.1:exon1:
c.C4295T:p.A1432V 1.65 3.2806 1 0.24 T . . . . .
orf1ab polyprotein_RFYFYTSKTTVASLI 18 . . . . . .
. . . .
1 4570 4570 C T exonic ORF1a. synonymous SNV
ORF1a:cds-YP_009724389.1:exon1:c.C4305T:p.I1435I,ORF1a:cds-YP_009725295.1:exon1:c.
C4305T:p.I1435I -2.37 0.191827 0.15748 1 T . . .
. . orf1ab polyprotein_RFYFYTSKTTVASLI 18 . . . .
. . . . . .
1 4579 4579 T A exonic ORF1a. synonymous SNV
ORF1a:cds-YP_009724389.1:exon1:c.T4314A:p.L1438L,ORF1a:cds-YP_009725295.1:exon1:c
.T4314A:p.L1438L -1.93 0.0292598 0.488189 1 T . . .
. . . . orf1ab_TLNDLNETL 0.04 . . . . .
. . .
1 4586 4586 C T exonic ORF1a. synonymous SNV
ORF1a:cds-YP_009724389.1:exon1:c.C4321T:p.L1441L,ORF1a:cds-YP_009725295.1:exon1:c
.C4321T:p.L1441L -3.3 -0.864858 0 1 T . . . .
. . . orf1ab_TLNDLNETL 0.04 . . . . . .
. .

1 4633 4633 C T exonic ORF1a. synonymous SNV
ORF1a:cds-YP_009724389.1:exon1:c.C4368T:p.G1456G,ORF1a:cds-YP_009725295.1:exon1:
c.C4368T:p.G1456G -3.3 -0.458441 0.811024 1 T . . .
. . . .
.
1 4698 4698 C T exonic ORF1a. nonsynonymous SNV
ORF1a:cds-YP_009724389.1:exon1:c.C4433T:p.S1478F,ORF1a:cds-YP_009725295.1:exon1:c
.C4433T:p.S1478F 1.65 3.2806 1 0.02 D . . . . .
. . . .

1 4754 4754 C T exonic ORF1a. nonsynonymous SNV
ORF1a:cds-YP_009724389.1:exon1:c.C4489T:p.P1497S,ORF1a:cds-YP_009725295.1:exon1:
c.C4489T:p.P1497S -3.3 -0.539724 0.716535 0.55 T . . .
. . . .
.
1 4755 4755 C T exonic ORF1a. nonsynonymous SNV
ORF1a:cds-YP_009724389.1:exon1:c.C4490T:p.P1497L,ORF1a:cds-YP_009725295.1:exon1:c
.C4490T:p.P1497L -0.132 1.16723 0.968504 0.01 D . . .
. . . .
.
1 4774 4774 A G exonic ORF1a. synonymous SNV
ORF1a:cds-YP_009724389.1:exon1:c.A4509G:p.E1503E,ORF1a:cds-YP_009725295.1:exon1:
c.A4509G:p.E1503E -3.23 0.110543 0.992126 1 T . . .
. . . . orf1ab_TISLAGSYK -1 . . . . .
. . .
1 4788 4788 C T exonic ORF1a. nonsynonymous SNV
ORF1a:cds-YP_009724389.1:exon1:c.C4523T:p.A1508V,ORF1a:cds-YP_009725295.1:exon1:
c.C4523T:p.A1508V 1.65 3.2806 0.992126 0.16 T . . . .
. . . orf1ab_TISLAGSYK -1 . . . . . .
. .
1 4795 4795 C T exonic ORF1a. synonymous SNV
ORF1a:cds-YP_009724389.1:exon1:c.C4530T:p.S1510S,ORF1a:cds-YP_009725295.1:exon1:
c.C4530T:p.S1510S -3.3 -1.35256 0 0.82 T . . . .
. . . orf1ab_TISLAGSYK -1 . . . . . .
. .
1 4809 4809 C T exonic ORF1a. nonsynonymous SNV
ORF1a:cds-YP_009724389.1:exon1:c.C4544T:p.S1515F,ORF1a:cds-YP_009725295.1:exon1:c
.C4544T:p.S1515F -1.02 0.760811 0.992126 0.04 D . . .
. . . .
.
1 4838 4838 A G exonic ORF1a. nonsynonymous SNV
ORF1a:cds-YP_009724389.1:exon1:c.A4573G:p.I1525V,ORF1a:cds-YP_009725295.1:exon1:c
.A4573G:p.I1525V 0.695 1.73621 1 1 T . . . .

. . . orf1ab_STQLGIEFLK 0.05 . . . . . .
. .
1 4870 4870 A G exonic ORF1a. synonymous SNV
ORF1a:cds-YP_009724389.1:exon1:c.A4605G:p.V1535V,ORF1a:cds-YP_009725295.1:exon1:
c.A4605G:p.V1535V -3.3 -2.65309 0.0787402 0.92 T . . .
. . orf1ab polyprotein_GDKSVYYTSNPTTFH 18 orf1ab_VYYTSNPTTF
-1 . . . .
1 4881 4881 G A exonic ORF1a. nonsynonymous SNV
ORF1a:cds-YP_009724389.1:exon1:c.G4616A:p.S1539N,ORF1a:cds-YP_009725295.1:exon1:
c.G4616A:p.S1539N -0.597 1.08594 0.0708661 0.15 T . . .
. . orf1ab polyprotein_GDKSVYYTSNPTTFH 18 orf1ab_VYYTSNPTTF
-1 . . . .
1 4910 4910 G A exonic ORF1a. nonsynonymous SNV
ORF1a:cds-YP_009724389.1:exon1:c.G4645A:p.E1549K,ORF1a:cds-YP_009725295.1:exon1:
c.G4645A:p.E1549K 1.65 4.256 1 0.15 T . . . . .
. . orf1ab_GEVITFDNL 0.03 . . . .
.
1 4918 4918 C A exonic ORF1a. synonymous SNV
ORF1a:cds-YP_009724389.1:exon1:c.C4653A:p.I1551I,ORF1a:cds-YP_009725295.1:exon1:c.
C4653A:p.I1551I -3.3 -0.702291 0.0314961 0.21 T . . .
. . . . orf1ab_GEVITFDNL 0.03 . . . . .
. . .
1 5008 5008 G T exonic ORF1a. synonymous SNV
ORF1a:cds-YP_009724389.1:exon1:c.G4743T:p.T1581T,ORF1a:cds-YP_009725295.1:exon1:c
.G4743T:p.T1581T -3.3 -0.946142 0.724409 1 T . . .
. . . . orf1ab_QVVDMSMTY 0.03 . .
nCoV-2019_16_RIGHT . . . . .
1 5009 5009 C T exonic ORF1a. stopgain
ORF1a:cds-YP_009724389.1:exon1:c.C4744T:p.Q1582X,ORF1a:cds-YP_009725295.1:exon1:
c.C4744T:p.Q1582X 0.778 1.89878 0.755906 . . . . . .
. . . . orf1ab_QVVDMSMTY 0.03 . .
nCoV-2019_16_RIGHT . . . . .
1 5023 5023 G T exonic ORF1a. nonsynonymous SNV
ORF1a:cds-YP_009724389.1:exon1:c.G4758T:p.M1586I,ORF1a:cds-YP_009725295.1:exon1:c
.G4758T:p.M1586I 1.65 4.256 1 0.1 T . . . . .
. . orf1ab_QVVDMSMTY 0.03 . . . .
. .
1 5084 5084 A G exonic ORF1a. nonsynonymous SNV
ORF1a:cds-YP_009724389.1:exon1:c.A4819G:p.I1607V,ORF1a:cds-YP_009725295.1:exon1:c
.A4819G:p.I1607V -0.727 0.516961 0.606299 0.68 T . . .
. . . . orf1ab_YLDGADVTKI 0.11 . . . . .
. . .
1 5128 5128 A G exonic ORF1a. synonymous SNV

ORF1a:cds-YP_009724389.1:exon1:c.A4863G:p.L1621L,ORF1a:cds-YP_009725295.1:exon1:c
.A4863G:p.L1621L 0.509 0.458094 1 1 T . . . .
. . . orf1ab_HEGKTFYVL 0.03 . . . . . .
. .
1 5129 5129 C T exonic ORF1a. nonsynonymous SNV
ORF1a:cds-YP_009724389.1:exon1:c.C4864T:p.P1622S,ORF1a:cds-YP_009725295.1:exon1:
c.C4864T:p.P1622S 1.65 3.32318 1 0.25 T . . . .
. . . orf1ab_LPNDDTLRVEAFEY 0.09 . . . . .
. . .
1 5165 5165 T C exonic ORF1a. nonsynonymous SNV
ORF1a:cds-YP_009724389.1:exon1:c.T4900C:p.Y1634H,ORF1a:cds-YP_009725295.1:exon1:
c.T4900C:p.Y1634H 1.65 2.19047 1 0.01 D . Peptidase_C16
. . . . . orf1ab_LPNDDTLRVEAFEY 0.09 . . .
. . . . .
1 5167 5167 C T exonic ORF1a. synonymous SNV
ORF1a:cds-YP_009724389.1:exon1:c.C4902T:p.Y1634Y,ORF1a:cds-YP_009725295.1:exon1:
c.C4902T:p.Y1634Y -1.25 0.657984 1 1 T . Peptidase_C16
. . . . . orf1ab_LPNDDTLRVEAFEY 0.09 . . .
. . . . .
1 5173 5173 C T exonic ORF1a. synonymous SNV
ORF1a:cds-YP_009724389.1:exon1:c.C4908T:p.H1636H,ORF1a:cds-YP_009725295.1:exon1:
c.C4908T:p.H1636H -3.3 -0.541354 0.968504 0.5 T .
Peptidase_C16 . . . . . orf1ab_EYYHTTDPSF -1
. . . .
1 5212 5212 A T exonic ORF1a. synonymous SNV
ORF1a:cds-YP_009724389.1:exon1:c.A4947T:p.A1649A,ORF1a:cds-YP_009725295.1:exon1:c
.A4947T:p.A1649A -3.3 -2.60688 0.590551 0.18 T .
Peptidase_C16 . . . .
. . . . .
1 5220 5220 A G exonic ORF1a. nonsynonymous SNV
ORF1a:cds-YP_009724389.1:exon1:c.A4955G:p.H1652R,ORF1a:cds-YP_009725295.1:exon1:
c.A4955G:p.H1652R 1.65 2.2571 1 0.38 T . Peptidase_C16 .
. . . .
.
1 5230 5230 G C exonic ORF1a. nonsynonymous SNV
ORF1a:cds-YP_009724389.1:exon1:c.G4965C:p.K1655N,ORF1a:cds-YP_009725295.1:exon1:
c.G4965C:p.K1655N -3.16 0.058315 1 0.03 D . Peptidase_C16
. . . . nCoV-2019_18_LEFT.
. . . .
1 5284 5284 C T exonic ORF1a. synonymous SNV
ORF1a:cds-YP_009724389.1:exon1:c.C5019T:p.N1673N,ORF1a:cds-YP_009725295.1:exon1:
c.C5019T:p.N1673N -3.3 -0.741244 0.968504 1 T .
Peptidase_C16 . . . .

nCoV-2019_18_LEFT_alt2 . . . . .
1 5344 5344 T C exonic ORF1a. synonymous SNV
ORF1a:cds-YP_009724389.1:exon1:c.T5079C:p.P1693P,ORF1a:cds-YP_009725295.1:exon1:
c.T5079C:p.P1693P -3.3 -0.874504 0.952756 0.3 T .
Peptidase_C16 . . . . . orf1ab_IELKFNPPAL -1 .
. . . .
1 5365 5365 C T exonic ORF1a. synonymous SNV
ORF1a:cds-YP_009724389.1:exon1:c.C5100T:p.Y1700Y,ORF1a:cds-YP_009725295.1:exon1:
c.C5100T:p.Y1700Y -0.753 0.391465 1 0.59 T . Peptidase_C16
. . . . . orf1ab_YYRARAGEAANF 0.09 . . .
. . . . .
1 5411 5411 G T exonic ORF1a. nonsynonymous SNV
ORF1a:cds-YP_009724389.1:exon1:c.G5146T:p.A1716S,ORF1a:cds-YP_009725295.1:exon1:
c.G5146T:p.A1716S 1.65 4.256 0.992126 0 D . Peptidase_C16
. . . .
. .
1 5425 5425 G T exonic ORF1a. nonsynonymous SNV
ORF1a:cds-YP_009724389.1:exon1:c.G5160T:p.K1720N,ORF1a:cds-YP_009725295.1:exon1:
c.G5160T:p.K1720N -3.13 -0.141575 0.992126 0.19 T .
Peptidase_C16 . . . .
. . . . .
1 5468 5468 T G exonic ORF1a. nonsynonymous SNV
ORF1a:cds-YP_009724389.1:exon1:c.T5203G:p.L1735V,ORF1a:cds-YP_009725295.1:exon1:c
.T5203G:p.L1735V 0.627 0.791244 0.992126 0.62 T .
Peptidase_C16 . . . . . orf1ab_SYLFQHANL -1 .
. . . .
1 5512 5512 C T exonic ORF1a. synonymous SNV
ORF1a:cds-YP_009724389.1:exon1:c.C5247T:p.N1749N,ORF1a:cds-YP_009725295.1:exon1:
c.C5247T:p.N1749N 1.65 0.857874 1 0.47 T . Peptidase_C16
. . . .
. .
1 5553 5553 A G exonic ORF1a. nonsynonymous SNV
ORF1a:cds-YP_009724389.1:exon1:c.A5288G:p.K1763R,ORF1a:cds-YP_009725295.1:exon1:
c.A5288G:p.K1763R -3.3 -0.674614 0.102362 0.15 T .
Peptidase_C16 . . . .
. . . . .
1 5554 5554 G T exonic ORF1a. nonsynonymous SNV
ORF1a:cds-YP_009724389.1:exon1:c.G5289T:p.K1763N,ORF1a:cds-YP_009725295.1:exon1:
c.G5289T:p.K1763N -3.3 0.324835 0.291339 0.05 D .
Peptidase_C16 . . . .
. . . . .
1 5572 5572 G T exonic ORF1a. nonsynonymous SNV
ORF1a:cds-YP_009724389.1:exon1:c.G5307T:p.M1769I,ORF1a:cds-YP_009725295.1:exon1:c

.G5307T:p.M1769I 1.65 4.256 1 0.29 T . Peptidase_C16 .
. . . . orf1ab_AVMYMGTLSY 0.09 . .
nCoV-2019_19_LEFT . . . . .
1 5575 5575 C T exonic ORF1a. synonymous SNV
ORF1a:cds-YP_009724389.1:exon1:c.C5310T:p.Y1770Y,ORF1a:cds-YP_009725295.1:exon1:
c.C5310T:p.Y1770Y -3.3 -0.807874 0.929134 1 T .
Peptidase_C16 . . . . . orf1ab_AVMYMGTLSY 0.09
. . nCoV-2019_19_LEFT . . . . .

1 5653 5653 T C exonic ORF1a. synonymous SNV
ORF1a:cds-YP_009724389.1:exon1:c.T5388C:p.Y1796Y,ORF1a:cds-YP_009725295.1:exon1:
c.T5388C:p.Y1796Y -3.3 -0.0749449 0.574803 0.81 T .
Peptidase_C16 . . . .
. . . . .
1 5668 5668 G A exonic ORF1a. synonymous SNV
ORF1a:cds-YP_009724389.1:exon1:c.G5403A:p.E1801E,ORF1a:cds-YP_009725295.1:exon1:
c.G5403A:p.E1801E -0.214 1.85732 1 0.92 T . Peptidase_C16
. . . orf1ab polyprotein_ESPFVMMSAPPAQYE 11 . . .
. . . .
1 5700 5700 C A exonic ORF1a. nonsynonymous SNV
ORF1a:cds-YP_009724389.1:exon1:c.C5435A:p.A1812D,ORF1a:cds-YP_009725295.1:exon1:
c.C5435A:p.A1812D 1.65 3.32318 1 0.38 T . Peptidase_C16
. . . orf1ab polyprotein_ESPFVMMSAPPAQYE 11 . . .
. . . .
1 5701 5701 T C exonic ORF1a. synonymous SNV
ORF1a:cds-YP_009724389.1:exon1:c.T5436C:p.A1812A,ORF1a:cds-YP_009725295.1:exon1:
c.T5436C:p.A1812A -1.33 0.391465 0.992126 1 T .
Peptidase_C16 . . . orf1ab polyprotein_ESPFVMMSAPPAQYE 11
. . . .

1 5722 5722 T C exonic ORF1a. synonymous SNV
ORF1a:cds-YP_009724389.1:exon1:c.T5457C:p.G1819G,ORF1a:cds-YP_009725295.1:exon1:
c.T5457C:p.G1819G -2.21 0.058315 0.92126 1 T .
Peptidase_C16 . . . .
. . . . .
1 5729 5729 A G exonic ORF1a. nonsynonymous SNV
ORF1a:cds-YP_009724389.1:exon1:c.A5464G:p.T1822A,ORF1a:cds-YP_009725295.1:exon1:
c.A5464G:p.T1822A -2.19 0.058315 0.0393701 0.29 T .
Peptidase_C16 . . . .
. . . . .
1 5730 5730 C T exonic ORF1a. nonsynonymous SNV
ORF1a:cds-YP_009724389.1:exon1:c.C5465T:p.T1822I,ORF1a:cds-YP_009725295.1:exon1:c.
C5465T:p.T1822I 1.65 0.857874 0.0551181 0.24 T .

Peptidase_C16	.	.	.	.	.	.	.	.	.	.
.	.	.	.	.
1	5795	5795	A	G	exonic	ORF1a.	nonsynonymous SNV
ORF1a:cds-YP_009724389.1:exon1:c.A5530G:p.T1844A,ORF1a:cds-YP_009725295.1:exon1:
c.A5530G:p.T1844A	1.65	2.2571	1	0.56	T	.	Peptidase_C16	.
.	.	.	.	.	.	.	.	.	.	.	.	.
.
1	5806	5806	C	T	exonic	ORF1a.	synonymous SNV
ORF1a:cds-YP_009724389.1:exon1:c.C5541T:p.C1847C,ORF1a:cds-YP_009725295.1:exon1:
c.C5541T:p.C1847C	-2.85	0.058315	0.992126	0.08	T	.
Peptidase_C16	.	.	.	.	.	.	.	.	.	.
.	.	.	.	.
1	5822	5822	C	T	exonic	ORF1a.	nonsynonymous SNV
ORF1a:cds-YP_009724389.1:exon1:c.C5557T:p.L1853F,ORF1a:cds-YP_009725295.1:exon1:c
.C5557T:p.L1853F	0.68	1.5908	0.976378	0.04	D	.	Peptidase_C16
.	.	.	.	.	.	.	.	.	.	.	.	.
.	.
1	5826	5826	C	T	exonic	ORF1a.	nonsynonymous SNV
ORF1a:cds-YP_009724389.1:exon1:c.C5561T:p.T1854I,ORF1a:cds-YP_009725295.1:exon1:c.
C5561T:p.T1854I	1.65	3.32318	1	0.04	D	.	Peptidase_C16
.	.	.	.	.	.	.	.	.	.	.	.	.
.	.
1	5878	5878	C	T	exonic	ORF1a.	synonymous SNV
ORF1a:cds-YP_009724389.1:exon1:c.C5613T:p.N1871N,ORF1a:cds-YP_009725295.1:exon1:
c.C5613T:p.N1871N	-2.08	-0.208205	0.984252	0.19	T	.
Peptidase_C16	.	.	.	.	.	orf1ab_ITDVFYKENSY	0.04
.	.	nCoV-2019_20_LEFT	.	.	.	.	.

1	5884	5884	C	T	exonic	ORF1a.	synonymous SNV
ORF1a:cds-YP_009724389.1:exon1:c.C5619T:p.Y1873Y,ORF1a:cds-YP_009725295.1:exon1:
c.C5619T:p.Y1873Y	0.731	1.12439	1	1	T	.	Peptidase_C16
.	.	.	.	.	orf1ab_ITDVFYKENSY	0.04	.	.
nCoV-2019_20_LEFT	.	.	.	.	.
1	6026	6026	C	T	exonic	ORF1a.	nonsynonymous SNV
ORF1a:cds-YP_009724389.1:exon1:c.C5761T:p.P1921S,ORF1a:cds-YP_009725295.1:exon1:
c.C5761T:p.P1921S	1.61	3.25655	0.984252	0.5	T	.	.	.
.	.	.	.	orf1ab_PYPNASFDNFKF	-1	.	.	.	.
.	.	.	.
1	6027	6027	C	T	exonic	ORF1a.	nonsynonymous SNV
ORF1a:cds-YP_009724389.1:exon1:c.C5762T:p.P1921L,ORF1a:cds-YP_009725295.1:exon1:c
.C5762T:p.P1921L	1.61	3.25655	0.992126	0.47	T	.	.	.
.	.	.	.	orf1ab_PYPNASFDNFKF	-1	.	.	.	.
.	.	.	.

1 6033 6033 C T exonic ORF1a. nonsynonymous SNV
ORF1a:cds-YP_009724389.1:exon1:c.C5768T:p.A1923V,ORF1a:cds-YP_009725295.1:exon1:
c.C5768T:p.A1923V 1.61 3.25655 1 0.03 D . . . .
. . . orf1ab_PYPNASFDNFKF -1 . . . . .
. . .
1 6037 6037 C A exonic ORF1a. nonsynonymous SNV
ORF1a:cds-YP_009724389.1:exon1:c.C5772A:p.S1924R,ORF1a:cds-YP_009725295.1:exon1:
c.C5772A:p.S1924R 0.432 0.458094 1 0.09 T . . . .
. . . orf1ab_PYPNASFDNFKF -1 . . . . .
. . .
1 6040 6040 C T exonic ORF1a. synonymous SNV
ORF1a:cds-YP_009724389.1:exon1:c.C5775T:p.F1925F,ORF1a:cds-YP_009725295.1:exon1:c
.C5775T:p.F1925F -0.823 0.391465 1 0.57 T . . . .
. orf1ab polyprotein_DNFKFVCDNIKFADD 16 orf1ab_PYPNASFDNFKF -1
. . . .
1 6166 6166 G A exonic ORF1a. synonymous SNV
ORF1a:cds-YP_009724389.1:exon1:c.G5901A:p.V1967V,ORF1a:cds-YP_009725295.1:exon1:
c.G5901A:p.V1967V -0.839 0.631654 1 1 T . . . .
. orf1ab polyprotein_KVTFFPDLNGDVVAI 19 . . . . .
. . . . .
1 6255 6255 C T exonic ORF1a. nonsynonymous SNV
ORF1a:cds-YP_009724389.1:exon1:c.C5990T:p.A1997V,ORF1a:cds-YP_009725295.1:exon1:
c.C5990T:p.A1997V 0.694 1.69764 1 0.33 T . . . .
. . . . nCoV-2019_20_RIGHT . .
. . .
1 6258 6258 C T exonic ORF1a. nonsynonymous SNV
ORF1a:cds-YP_009724389.1:exon1:c.C5993T:p.T1998I,ORF1a:cds-YP_009725295.1:exon1:c.
C5993T:p.T1998I 1.61 3.29661 1 0.49 T . . . .
. . . . nCoV-2019_20_RIGHT . .
. . .
1 6309 6309 G T exonic ORF1a. nonsynonymous SNV
ORF1a:cds-YP_009724389.1:exon1:c.G6044T:p.S2015I,ORF1a:cds-YP_009725295.1:exon1:c.
G6044T:p.S2015I 0.695 2.65702 1 0.01 D . . . .
. . . .

1 6310 6310 C T exonic ORF1a. synonymous SNV
ORF1a:cds-YP_009724389.1:exon1:c.C6045T:p.S2015S,ORF1a:cds-YP_009725295.1:exon1:
c.C6045T:p.S2015S -3.22 -0.114535 0.992126 0.88 T . . .
. . . . orf1ab_TKPVETSNSF -1 . . . .
. . . .
1 6312 6312 C A exonic ORF1a. nonsynonymous SNV
ORF1a:cds-YP_009724389.1:exon1:c.C6047A:p.T2016K,ORF1a:cds-YP_009725295.1:exon1:
c.C6047A:p.T2016K 1.61 3.29661 1 0.03 D . . . .

. . . orf1ab_TKPVETSNSF -1 . . . . .
. . .
1 6317 6317 C T exonic ORF1a. nonsynonymous SNV
ORF1a:cds-YP_009724389.1:exon1:c.C6052T:p.P2018S,ORF1a:cds-YP_009725295.1:exon1:
c.C6052T:p.P2018S 1.61 3.29661 1 0.12 T . . . .
. . . orf1ab_TKPVETSNSF -1 . . . . .
. . .
1 6349 6349 G T exonic ORF1a. synonymous SNV
ORF1a:cds-YP_009724389.1:exon1:c.G6084T:p.L2028L,ORF1a:cds-YP_009725295.1:exon1:c
.G6084T:p.L2028L 0.673 2.55043 0.992126 1 T . . .
. . . . orf1ab_TSNSFDVLK 0.09 . . . . .
. . .
1 6352 6352 G T exonic ORF1a. nonsynonymous SNV
ORF1a:cds-YP_009724389.1:exon1:c.G6087T:p.K2029N,ORF1a:cds-YP_009725295.1:exon1:
c.G6087T:p.K2029N -2.52 -0.327732 0.88189 0.46 T . . .
. . . . orf1ab_TSNSFDVLK 0.09 . . . . .
. . .
1 6433 6433 C T exonic ORF1a. synonymous SNV
ORF1a:cds-YP_009724389.1:exon1:c.C6168T:p.T2056T,ORF1a:cds-YP_009725295.1:exon1:c
.C6168T:p.T2056T 1.61 3.29661 1 0.98 T . . . .
. . . orf1ab_VVENPTIQK -1 . . . . . .
. .
1 6445 6445 C T exonic ORF1a. synonymous SNV
ORF1a:cds-YP_009724389.1:exon1:c.C6180T:p.D2060D,ORF1a:cds-YP_009725295.1:exon1:
c.C6180T:p.D2060D 0.557 0.525055 0.952756 0.48 T . . .
. . . .
.
1 6446 6446 G T exonic ORF1a. nonsynonymous SNV
ORF1a:cds-YP_009724389.1:exon1:c.G6181T:p.V2061F,ORF1a:cds-YP_009725295.1:exon1:
c.G6181T:p.V2061F -0.756 0.631654 0.889764 0.01 D . . .
. . . .
.
1 6451 6451 T C exonic ORF1a. synonymous SNV
ORF1a:cds-YP_009724389.1:exon1:c.T6186C:p.L2062L,ORF1a:cds-YP_009725295.1:exon1:c
.T6186C:p.L2062L 0.424 0.311858 0.929134 0.34 T . . .
. . . .
.
1 6466 6466 A G exonic ORF1a. synonymous SNV
ORF1a:cds-YP_009724389.1:exon1:c.A6201G:p.K2067K,ORF1a:cds-YP_009725295.1:exon1:
c.A6201G:p.K2067K 1.61 2.23063 1 1 T . . . .
. . . . nCoV-2019_22_LEFT . . .
. .
1 6476 6476 G T exonic ORF1a. nonsynonymous SNV

ORF1a:cds-YP_009724389.1:exon1:c.G6211T:p.V2071F,ORF1a:cds-YP_009725295.1:exon1:
c.G6211T:p.V2071F 1.61 4.1494 1 0.16 T . . . . .
. . orf1ab_TEVVGDIIL 0.03 . . nCoV-2019_22_LEFT . .
. . .
1 6484 6484 A T exonic ORF1a . synonymous SNV
ORF1a:cds-YP_009724389.1:exon1:c.A6219T:p.G2073G,ORF1a:cds-YP_009725295.1:exon1:
c.A6219T:p.G2073G -0.293 0.418457 1 1 T . . . .
. . . orf1ab_TEVVGDIIL 0.03 . . nCoV-2019_22_LEFT .
. . . .
1 6491 6491 A G exonic ORF1a . nonsynonymous SNV
ORF1a:cds-YP_009724389.1:exon1:c.A6226G:p.I2076V,ORF1a:cds-YP_009725295.1:exon1:c
.A6226G:p.I2076V 1.61 2.23063 1 0.71 T . . . .
. . . orf1ab_TEVVGDIIL 0.03 . . nCoV-2019_22_LEFT .
. . . .
1 6500 6500 C T exonic ORF1a . nonsynonymous SNV
ORF1a:cds-YP_009724389.1:exon1:c.C6235T:p.P2079S,ORF1a:cds-YP_009725295.1:exon1:
c.C6235T:p.P2079S 0.747 1.91083 1 0.26 T . . . .
. . . orf1ab_ILKPANNSL 0.09 . . . . . .
. .
1 6538 6538 C T exonic ORF1a . synonymous SNV
ORF1a:cds-YP_009724389.1:exon1:c.C6273T:p.G2091G,ORF1a:cds-YP_009725295.1:exon1:
c.C6273T:p.G2091G -3.22 -0.860724 0.574803 1 T . . .
. . . . orf1ab_ITEEVGHTDLMAAY 0.02 . .
nCoV-2019_21_RIGHT_alt0 . . . . .
1 6573 6573 C T exonic ORF1a . nonsynonymous SNV
ORF1a:cds-YP_009724389.1:exon1:c.C6308T:p.S2103F,ORF1a:cds-YP_009725295.1:exon1:c
.C6308T:p.S2103F 0.737 1.80424 0.952756 0.36 T . . .
. . . .
.
1 6582 6582 C T exonic ORF1a . nonsynonymous SNV
ORF1a:cds-YP_009724389.1:exon1:c.C6317T:p.T2106I,ORF1a:cds-YP_009725295.1:exon1:c.
C6317T:p.T2106I 0.786 1.91083 1 0.42 T . . . .
. . . orf1ab_TIKKPNEL 0.04 . . . . . .
. .
1 6616 6616 A G exonic ORF1a . synonymous SNV
ORF1a:cds-YP_009724389.1:exon1:c.A6351G:p.L2117L,ORF1a:cds-YP_009725295.1:exon1:c
.A6351G:p.L2117L -2.02 0.525055 0.952756 1 T . . .
. . orf1ab polyprotein_NELSRVLGLKTLATH 19 orf1ab_KKPNELSRVL
0.06 . . . .
1 6633 6633 C T exonic ORF1a . nonsynonymous SNV
ORF1a:cds-YP_009724389.1:exon1:c.C6368T:p.A2123V,ORF1a:cds-YP_009725295.1:exon1:
c.C6368T:p.A2123V -0.128 1.16465 0.984252 0.71 T . . .
. . orf1ab polyprotein_NELSRVLGLKTLATH 19 . . . .

. . . . . .
1 6646 6646 A G exonic ORF1a. synonymous SNV
ORF1a:cds-YP_009724389.1:exon1:c.A6381G:p.L2127L,ORF1a:cds-YP_009725295.1:exon1:c
.A6381G:p.L2127L -3.3 -0.327732 0.275591 0.56 T . . .
. . . .
.
1 6681 6681 C T exonic ORF1a. nonsynonymous SNV
ORF1a:cds-YP_009724389.1:exon1:c.C6416T:p.A2139V,ORF1a:cds-YP_009725295.1:exon1:
c.C6416T:p.A2139V -0.71 0.20526 0.00787402 0.44 T . . .
. . . . orf1ab_VPWDTIANY 0.01 . . . . .
. . .
1 6745 6745 T C exonic ORF1a. synonymous SNV
ORF1a:cds-YP_009724389.1:exon1:c.T6480C:p.C2160C,ORF1a:cds-YP_009725295.1:exon1:
c.T6480C:p.C2160C -3.3 -2.5663 0 0.28 T . . . .
. . . . nCoV-2019_23_LEFT . . .
. .
1 6781 6781 C T exonic ORF1a. synonymous SNV
ORF1a:cds-YP_009724389.1:exon1:c.C6516T:p.F2172F,ORF1a:cds-YP_009725295.1:exon1:c
.C6516T:p.F2172F -3.3 -0.114535 0.0314961 0.29 T . . .
. . . . orf1ab_NYMPYFFTL -1 . . . . .
. . .
1 6790 6790 A G exonic ORF1a. synonymous SNV
ORF1a:cds-YP_009724389.1:exon1:c.A6525G:p.L2175L,ORF1a:cds-YP_009725295.1:exon1:c
.A6525G:p.L2175L 0.438 0.84485 0.370079 1 T . . .
. . . . orf1ab_NYMPYFFTL -1 . . . . .
. . .
1 6802 6802 G T exonic ORF1a. nonsynonymous SNV
ORF1a:cds-YP_009724389.1:exon1:c.G6537T:p.L2179F,ORF1a:cds-YP_009725295.1:exon1:c
.G6537T:p.L2179F 0.646 2.23063 1 0.3 T . . . .
. . . .

1 6816 6816 G A exonic ORF1a. nonsynonymous SNV
ORF1a:cds-YP_009724389.1:exon1:c.G6551A:p.R2184K,ORF1a:cds-YP_009725295.1:exon1:
c.G6551A:p.R2184K -0.75 0.418457 0.0866142 1 T . . .
. . . .
.
1 6825 6825 A C exonic ORF1a. nonsynonymous SNV
ORF1a:cds-YP_009724389.1:exon1:c.A6560C:p.N2187T,ORF1a:cds-YP_009725295.1:exon1:
c.A6560C:p.N2187T -0.742 0.418457 0.811024 0.21 T . . .
. . . . orf1ab_NSRIKASM 0.07 . . . . .
. . .
1 6865 6865 G T exonic ORF1a. nonsynonymous SNV
ORF1a:cds-YP_009724389.1:exon1:c.G6600T:p.K2200N,ORF1a:cds-YP_009725295.1:exon1:

c.G6600T:p.K2200N -0.689 0.20526 1 0.45 T . . . .
. . . orf1ab_KASMPTTIAK-1 . . nCoV-2019_22_RIGHT
. . . . .
1 6884 6884 G A exonic ORF1a. nonsynonymous SNV
ORF1a:cds-YP_009724389.1:exon1:c.G6619A:p.G2207S,ORF1a:cds-YP_009725295.1:exon1:
c.G6619A:p.G2207S 0.695 2.55043 0.795276 0.29 T . . .
. . . .
.
1 6884 6884 G T exonic ORF1a. nonsynonymous SNV
ORF1a:cds-YP_009724389.1:exon1:c.G6619T:p.G2207C,ORF1a:cds-YP_009725295.1:exon1:
c.G6619T:p.G2207C 0.695 2.55043 0.795276 0.21 T . . .
. . . .
.
1 6896 6896 C T exonic ORF1a. synonymous SNV
ORF1a:cds-YP_009724389.1:exon1:c.C6631T:p.L2211L,ORF1a:cds-YP_009725295.1:exon1:c
.C6631T:p.L2211L -3.15 -0.00793701 0.992126 1 T . . .
. . orf1ab polyprotein_LEASFNYLKSPNFSK 7.2 . . . .
. . . . . .
1 6901 6901 G T exonic ORF1a. nonsynonymous SNV
ORF1a:cds-YP_009724389.1:exon1:c.G6636T:p.E2212D,ORF1a:cds-YP_009725295.1:exon1:
c.G6636T:p.E2212D -3.3 -0.967323 0.637795 0.67 T . . .
. . orf1ab polyprotein_LEASFNYLKSPNFSK 7.2 orf1ab_ASFNYLKSPNFSK
-1 . . . .
1 6926 6926 C T exonic ORF1a. nonsynonymous SNV
ORF1a:cds-YP_009724389.1:exon1:c.C6661T:p.P2221S,ORF1a:cds-YP_009725295.1:exon1:
c.C6661T:p.P2221S 0.761 1.80424 1 0.65 T . . . .
. orf1ab polyprotein_LEASFNYLKSPNFSK 7.2 orf1ab_ASFNYLKSPNFSK -1
. . . .
1 6935 6935 T G exonic ORF1a. nonsynonymous SNV
ORF1a:cds-YP_009724389.1:exon1:c.T6670G:p.S2224A,ORF1a:cds-YP_009725295.1:exon1:
c.T6670G:p.S2224A 1.65 2.12403 1 0.19 T . . . .
. orf1ab polyprotein_LEASFNYLKSPNFSK 7.2 orf1ab_ASFNYLKSPNFSK -1
. . . .
1 6990 6990 C T exonic ORF1a. nonsynonymous SNV
ORF1a:cds-YP_009724389.1:exon1:c.C6725T:p.S2242F,ORF1a:cds-YP_009725295.1:exon1:c
.C6725T:p.S2242F -1.24 0.525055 0.858268 0 D . . .
Transmembrane . orf1ab polyprotein_GSLIYSTAALGVLMS 19 . .
. . . . hypermutable low-fitness site

1 7000 7000 C T exonic ORF1a. synonymous SNV
ORF1a:cds-YP_009724389.1:exon1:c.C6735T:p.Y2245Y,ORF1a:cds-YP_009725295.1:exon1:
c.C6735T:p.Y2245Y 0.475 0.951449 1 1 T . . .
Transmembrane . orf1ab polyprotein_GSLIYSTAALGVLMS 19 . .

. . . .
1 7067 7067 G T exonic ORF1a. stopgain
ORF1a:cds-YP_009724389.1:exon1:c.G6802T:p.E2268X,ORF1a:cds-YP_009725295.1:exon1:
c.G6802T:p.E2268X 1.65 4.256 0.992126 . . . Lumenal .
. . . .
.
1 7071 7071 G A exonic ORF1a. nonsynonymous SNV
ORF1a:cds-YP_009724389.1:exon1:c.G6806A:p.G2269D,ORF1a:cds-YP_009725295.1:exon1:
c.G6806A:p.G2269D -3.3 -1.18052 0 0.35 T . Lumenal .
. . . .
.
1 7071 7071 G T exonic ORF1a. nonsynonymous SNV
ORF1a:cds-YP_009724389.1:exon1:c.G6806T:p.G2269V,ORF1a:cds-YP_009725295.1:exon1:
c.G6806T:p.G2269V -3.3 -1.18052 0 0.23 T . Lumenal .
. . . .
.
1 7101 7101 C T exonic ORF1a. nonsynonymous SNV
ORF1a:cds-YP_009724389.1:exon1:c.C6836T:p.A2279V,ORF1a:cds-YP_009725295.1:exon1:
c.C6836T:p.A2279V -0.657 0.20526 0.771654 0.6 T . Lumenal
. . . . nCoV-2019_23_RIGHT
. . . . .
1 7119 7119 C T exonic ORF1a. nonsynonymous SNV
ORF1a:cds-YP_009724389.1:exon1:c.C6854T:p.S2285F,ORF1a:cds-YP_009725295.1:exon1:c
.C6854T:p.S2285F -1.25 0.525055 0.984252 0.01 D . Lumenal
. . . .
. .
1 7165 7165 C T exonic ORF1a. synonymous SNV
ORF1a:cds-YP_009724389.1:exon1:c.C6900T:p.T2300T,ORF1a:cds-YP_009725295.1:exon1:c
.C6900T:p.T2300T -3.3 -1.18052 0.480315 0.75 T . Lumenal
. . . . . orf1ab_YPSLETIQI 0.12 . . . .
. . . .
1 7210 7210 A G exonic ORF1a. synonymous SNV
ORF1a:cds-YP_009724389.1:exon1:c.A6945G:p.K2315K,ORF1a:cds-YP_009725295.1:exon1:
c.A6945G:p.K2315K -1.36 0.641291 0.929205 0.51 T . Lumenal
. . . orf1ab polyprotein_ISSFKWDLTAFGLVA 15 . . .
. . . .
1 7267 7267 C T exonic ORF1a. synonymous SNV
ORF1a:cds-YP_009724389.1:exon1:c.C7002T:p.F2334F,ORF1a:cds-YP_009725295.1:exon1:c
.C7002T:p.F2334F -3.3 -2.97342 0.292047 0.68 T . . .
Transmembrane . orf1ab polyprotein_EWFLAYILFTRFFYV 20
orf1ab_AEWFLAYILF -1 . . . .

1 7303 7303 C T exonic ORF1a. synonymous SNV

ORF1a:cds-YP_009724389.1:exon1:c.C7038T:p.I2346I,ORF1a:cds-YP_009725295.1:exon1:c.
C7038T:p.I2346I -0.725 -0.0312126 0.992134 0.59 T . . .
Transmembrane . orf1ab polyprotein_RFFYVLGLAAIMQLF 11 . .
. . . .
1 7319 7319 A G exonic ORF1a. nonsynonymous SNV
ORF1a:cds-YP_009724389.1:exon1:c.A7054G:p.S2352G,ORF1a:cds-YP_009725295.1:exon1:
c.A7054G:p.S2352G 0.695 1.73411 1 0.39 T . . .
Transmembrane . orf1ab polyprotein_IMQLFFSYFAVHFIS 18 . .
. . nCoV-2019_25_LEFT. . . . .

1 7321 7321 C T exonic ORF1a. synonymous SNV
ORF1a:cds-YP_009724389.1:exon1:c.C7056T:p.S2352S,ORF1a:cds-YP_009725295.1:exon1:
c.C7056T:p.S2352S -0.244 1.06161 1 0.51 T . . .
Transmembrane . orf1ab polyprotein_IMQLFFSYFAVHFIS 18 . .
. . nCoV-2019_25_LEFT. . . . .

1 7393 7393 G A exonic ORF1a. synonymous SNV
ORF1a:cds-YP_009724389.1:exon1:c.G7128A:p.P2376P,ORF1a:cds-YP_009725295.1:exon1:
c.G7128A:p.P2376P -1.52 -0.115276 0.992134 1 T . . .
Transmembrane . orf1ab polyprotein_WLIINLVQMAPISAM 5.9
orf1ab_QMAPISAMVRM -1 . . nCoV-2019_24_RIGHT . .
. . .
1 7463 7463 G A exonic ORF1a. nonsynonymous SNV
ORF1a:cds-YP_009724389.1:exon1:c.G7198A:p.V2400I,ORF1a:cds-YP_009725295.1:exon1:c
.G7198A:p.V2400I 0.555 0.89348 1 0.53 T . . . .
. orf1ab polyprotein_FFASFYYVWKSYVHV 15 orf1ab_YVWKSYVHV 0.13 .
. . . .
1 7498 7498 G A exonic ORF1a. nonsynonymous SNV
ORF1a:cds-YP_009724389.1:exon1:c.G7233A:p.M2411I,ORF1a:cds-YP_009725295.1:exon1:c
.G7233A:p.M2411I 1.65 4.256 1 0.05 D . . . . .
. . . .

1 7528 7528 C T exonic ORF1a. synonymous SNV
ORF1a:cds-YP_009724389.1:exon1:c.C7263T:p.V2421V,ORF1a:cds-YP_009725295.1:exon1:
c.C7263T:p.V2421V -3.3 -0.703717 0.968535 1 T . . .
. . . .
.
1 7561 7561 G A exonic ORF1a. synonymous SNV
ORF1a:cds-YP_009724389.1:exon1:c.G7296A:p.R2432R,ORF1a:cds-YP_009725295.1:exon1:
c.G7296A:p.R2432R 0.555 0.89348 1 1 T . . . .
. . . .

1 7564 7564 C T exonic ORF1a. synonymous SNV

ORF1a:cds-YP_009724389.1:exon1:c.C7299T:p.S2433S,ORF1a:cds-YP_009725295.1:exon1:
c.C7299T:p.S2433S -3.3 -0.703717 0.968535 0.75 T . . .
. . . .
.
1 7609 7609 C T exonic ORF1a. synonymous SNV
ORF1a:cds-YP_009724389.1:exon1:c.C7344T:p.H2448H,ORF1a:cds-YP_009725295.1:exon1:
c.C7344T:p.H2448H -1.22 0.473165 1 1 T . . . .
. . . .

1 7787 7787 G A exonic ORF1a. nonsynonymous SNV
ORF1a:cds-YP_009724389.1:exon1:c.G7522A:p.A2508T,ORF1a:cds-YP_009725295.1:exon1:
c.G7522A:p.A2508T 1.65 4.256 1 0.09 T . . . . . .
. . . .

1 7798 7798 G T exonic ORF1a. nonsynonymous SNV
ORF1a:cds-YP_009724389.1:exon1:c.G7533T:p.K2511N,ORF1a:cds-YP_009725295.1:exon1:
c.G7533T:p.K2511N -0.309 1.56598 1 0.02 D . . . .
. . . orf1ab_KTYERHSL 0.09 . . . . . .
. .
1 7844 7844 C T exonic ORF1a. synonymous SNV
ORF1a:cds-YP_009724389.1:exon1:c.C7579T:p.L2527L,ORF1a:cds-YP_009725295.1:exon1:c
.C7579T:p.L2527L -3.3 -0.115276 0.968535 1 T . . .
. . orf1ab polyprotein_HSLSHFVNLDNLRAN 13 . . . .
. . . . . .
1 7855 7855 T C exonic ORF1a. synonymous SNV
ORF1a:cds-YP_009724389.1:exon1:c.T7590C:p.N2530N,ORF1a:cds-YP_009725295.1:exon1:
c.T7590C:p.N2530N -3.3 -0.535591 0.968535 0.49 T . . .
. . orf1ab polyprotein_HSLSHFVNLDNLRAN 13 . . . .
. . . . . .
1 7858 7858 C T exonic ORF1a. synonymous SNV
ORF1a:cds-YP_009724389.1:exon1:c.C7593T:p.N2531N,ORF1a:cds-YP_009725295.1:exon1:
c.C7593T:p.N2531N 0.787 1.90224 1 0.58 T . . . .
. . . .

1 7860 7860 C T exonic ORF1a. nonsynonymous SNV
ORF1a:cds-YP_009724389.1:exon1:c.C7595T:p.T2532I,ORF1a:cds-YP_009725295.1:exon1:c.
C7595T:p.T2532I 0.756 1.90224 1 0.17 T . . . .
. . . .

1 7863 7863 A G exonic ORF1a. nonsynonymous SNV
ORF1a:cds-YP_009724389.1:exon1:c.A7598G:p.K2533R,ORF1a:cds-YP_009725295.1:exon1:
c.A7598G:p.K2533R 1.65 2.23849 1 0.05 D . . . .
. . . .

1 7869 7869 C T exonic ORF1a. nonsynonymous SNV
ORF1a:cds-YP_009724389.1:exon1:c.C7604T:p.S2535L,ORF1a:cds-YP_009725295.1:exon1:c
.C7604T:p.S2535L 0.761 1.90224 1 0.1 T . . . .
. . . .

1 8003 8003 G T exonic ORF1a. nonsynonymous SNV
ORF1a:cds-YP_009724389.1:exon1:c.G7738T:p.V2580F,ORF1a:cds-YP_009725295.1:exon1:
c.G7738T:p.V2580F 1.65 4.256 1 0 D . . . . .
orf1ab polyprotein_CQPILLLDQALVSDV 11 . . . .
nCoV-2019_26_RIGHT . . . . .
1 8017 8017 G A exonic ORF1a. synonymous SNV
ORF1a:cds-YP_009724389.1:exon1:c.G7752A:p.A2584A,ORF1a:cds-YP_009725295.1:exon1:
c.G7752A:p.A2584A -3.3 -0.955905 0.960669 0.49 T . . .
. . . . orf1ab_AEVAVKMFDAY -1 . .
nCoV-2019_26_RIGHT . . . . .
1 8027 8027 G T exonic ORF1a. nonsynonymous SNV
ORF1a:cds-YP_009724389.1:exon1:c.G7762T:p.V2588F,ORF1a:cds-YP_009725295.1:exon1:
c.G7762T:p.V2588F -0.258 1.73411 1 0.05 D . . . .
. . . orf1ab_AEVAVKMFDAY -1 . . . . .
. . .
1 8081 8081 A C exonic ORF1a. nonsynonymous SNV
ORF1a:cds-YP_009724389.1:exon1:c.A7816C:p.M2606L,ORF1a:cds-YP_009725295.1:exon1:
c.A7816C:p.M2606L 1.65 2.23849 1 0.4 T . . . .
. . . orf1ab_SSTFNVPMEKLK -1 . . . . .
. . .
1 8085 8085 A G exonic ORF1a. nonsynonymous SNV
ORF1a:cds-YP_009724389.1:exon1:c.A7820G:p.E2607G,ORF1a:cds-YP_009725295.1:exon1:
c.A7820G:p.E2607G 1.65 2.23849 1 0.01 D . . . .
. . . orf1ab_SSTFNVPMEKLK -1 . . . . .
. . .
1 8102 8102 G T exonic ORF1a. nonsynonymous SNV
ORF1a:cds-YP_009724389.1:exon1:c.G7837T:p.V2613F,ORF1a:cds-YP_009725295.1:exon1:
c.G7837T:p.V2613F -0.324 1.56598 0.134724 0.02 D . . .
. . . .
.
1 8123 8123 C T exonic ORF1a. nonsynonymous SNV
ORF1a:cds-YP_009724389.1:exon1:c.C7858T:p.L2620F,ORF1a:cds-YP_009725295.1:exon1:c
.C7858T:p.L2620F -3.3 -0.619654 0.236984 0.04 D . . .
. . . . orf1ab_ATAEAELAK -1 . . . . .
. . .
1 8131 8131 G T exonic ORF1a. nonsynonymous SNV
ORF1a:cds-YP_009724389.1:exon1:c.G7866T:p.K2622N,ORF1a:cds-YP_009725295.1:exon1:

c.G7866T:p.K2622N 0.646 2.23849 1 0.04 D . . . .
. orf1ab polyprotein_AKNVSLDNVLSTFIS 15 orf1ab_ATAEAELAK -1 .
. . . .
1 8139 8139 C T exonic ORF1a. nonsynonymous SNV
ORF1a:cds-YP_009724389.1:exon1:c.C7874T:p.S2625F,ORF1a:cds-YP_009725295.1:exon1:c
.C7874T:p.S2625F 1.65 3.33131 1 0.04 D . . . .
. orf1ab polyprotein_AKNVSLDNVLSTFIS 15 orf1ab_AEAELAKNVSL -1
. . . .
1 8140 8140 C T exonic ORF1a. synonymous SNV
ORF1a:cds-YP_009724389.1:exon1:c.C7875T:p.S2625S,ORF1a:cds-YP_009725295.1:exon1:
c.C7875T:p.S2625S -0.753 0.389102 1 0.91 T . . . .
. orf1ab polyprotein_AKNVSLDNVLSTFIS 15 orf1ab_AEAELAKNVSL -1
. . . .
1 8185 8185 G A exonic ORF1a. synonymous SNV
ORF1a:cds-YP_009724389.1:exon1:c.G7920A:p.G2640G,ORF1a:cds-YP_009725295.1:exon1:
c.G7920A:p.G2640G -3.12 -0.283402 0.992134 0.73 T . . .
. . orf1ab polyprotein_STFISAARQGFVDSD 20 . . . .
. . . . . .
1 8208 8208 C T exonic ORF1a. nonsynonymous SNV
ORF1a:cds-YP_009724389.1:exon1:c.C7943T:p.T2648I,ORF1a:cds-YP_009725295.1:exon1:c.
C7943T:p.T2648I 1.65 3.2943 0.992126 0 D . . . .
. . . .

1 8244 8244 A G exonic ORF1a. nonsynonymous SNV
ORF1a:cds-YP_009724389.1:exon1:c.A7979G:p.Q2660R,ORF1a:cds-YP_009725295.1:exon1:
c.A7979G:p.Q2660R 1.65 2.26391 1 0.61 T . . . .
. . . .

1 8247 8247 C T exonic ORF1a. nonsynonymous SNV
ORF1a:cds-YP_009724389.1:exon1:c.C7982T:p.S2661F,ORF1a:cds-YP_009725295.1:exon1:c
.C7982T:p.S2661F 1.65 3.2943 1 0.05 D . . . . .
. . . .

1 8320 8320 C T exonic ORF1a. synonymous SNV
ORF1a:cds-YP_009724389.1:exon1:c.C8055T:p.P2685P,ORF1a:cds-YP_009725295.1:exon1:
c.C8055T:p.P2685P -2.1 -0.346425 0.984252 0.24 T . . .
. . . . orf1ab_VENMTPRDL 0.16 . .
nCoV-2019_27_RIGHT . . . . . .
1 8327 8327 C T exonic ORF1a. nonsynonymous SNV
ORF1a:cds-YP_009724389.1:exon1:c.C8062T:p.L2688F,ORF1a:cds-YP_009725295.1:exon1:c
.C8062T:p.L2688F -0.197 1.09613 1 0.02 D . . . .
. . . orf1ab_VENMTPRDL 0.16 . . nCoV-2019_27_RIGHT
. . . . .

1 8386 8386 C T exonic ORF1a. synonymous SNV
ORF1a:cds-YP_009724389.1:exon1:c.C8121T:p.H2707H,ORF1a:cds-YP_009725295.1:exon1:
c.C8121T:p.H2707H 0.72 1.92044 1 0.1 T . . . .
. orf1ab polyprotein_SHNIALIWNVKDFMS 20 orf1ab_VAKSHNIAL -1 .
. . . .
1 8421 8421 T C exonic ORF1a. nonsynonymous SNV
ORF1a:cds-YP_009724389.1:exon1:c.T8156C:p.M2719T,ORF1a:cds-YP_009725295.1:exon1:
c.T8156C:p.M2719T 1.65 2.19521 0.992126 0.01 D . . .
. . orf1ab polyprotein_SHNIALIWNVKDFMS 20 orf1ab_NVKDFMSL 0.07
. . . .
1 8438 8438 C T exonic ORF1a. synonymous SNV
ORF1a:cds-YP_009724389.1:exon1:c.C8173T:p.L2725L,ORF1a:cds-YP_009725295.1:exon1:c
.C8173T:p.L2725L -2.16 0.340504 1 1 T . . . .
. . . .

1 8442 8442 G A exonic ORF1a. nonsynonymous SNV
ORF1a:cds-YP_009724389.1:exon1:c.G8177A:p.R2726Q,ORF1a:cds-YP_009725295.1:exon1:
c.G8177A:p.R2726Q 1.65 4.256 1 0.03 D . . . . .
. . . .

1 8498 8498 G T exonic ORF1a. nonsynonymous SNV
ORF1a:cds-YP_009724389.1:exon1:c.G8233T:p.A2745S,ORF1a:cds-YP_009725295.1:exon1:
c.G8233T:p.A2745S 1.65 4.256 1 0.73 T . . . . .
. . . .

1 8608 8608 T C exonic ORF1a. synonymous SNV
ORF1a:cds-YP_009724389.1:exon1:c.T8343C:p.L2781L,ORF1a:cds-YP_009725295.1:exon1:c
.T8343C:p.L2781L -3.3 -2.75068 0 0.31 T . . .
Transmembrane . orf1ab polyprotein_VTLVFLFVAAIFYLI 8.7
orf1ab_VFLFVAAIF 0.07 . . nCoV-2019_29_LEFT . . . .
.
1 8665 8665 T A exonic ORF1a. synonymous SNV
ORF1a:cds-YP_009724389.1:exon1:c.T8400A:p.T2800T,ORF1a:cds-YP_009725295.1:exon1:c
.T8400A:p.T2800T 0.48 0.95874 1 0.58 T . Lumenal .
. . orf1ab polyprotein_IFYLITPVHVMSKHT 17 orf1ab_HVMSKHTDF 0.16
. . . .
1 8673 8673 C T exonic ORF1a. nonsynonymous SNV
ORF1a:cds-YP_009724389.1:exon1:c.C8408T:p.S2803L,ORF1a:cds-YP_009725295.1:exon1:c
.C8408T:p.S2803L 1.65 3.2943 0.992126 0.06 T . Lumenal .
. . . . orf1ab_HTDFSSEIIGY 0.01 . . . .
. . . .
1 8683 8683 C T exonic ORF1a. synonymous SNV
ORF1a:cds-YP_009724389.1:exon1:c.C8418T:p.I2806I,ORF1a:cds-YP_009725295.1:exon1:c.

C8418T:p.I2806I -2.35 0.0657323 0.992126 1 T . Lumenal
. . . . . orf1ab_HTDFSSEIIGY 0.01 . . .
. . . . .
1 8692 8692 C T exonic ORF1a. synonymous SNV
ORF1a:cds-YP_009724389.1:exon1:c.C8427T:p.Y2809Y,ORF1a:cds-YP_009725295.1:exon1:
c.C8427T:p.Y2809Y -3.3 -0.0716535 0.992126 0.89 T . Lumenal
. . . . . orf1ab_HTDFSSEIIGY 0.01 . . .
. . . . .
1 8752 8752 C G exonic ORF1a. nonsynonymous SNV
ORF1a:cds-YP_009724389.1:exon1:c.C8487G:p.N2829K,ORF1a:cds-YP_009725295.1:exon1:
c.C8487G:p.N2829K -2 0.340504 1 0 D . Lumenal .
. . . .
.
1 8782 8782 C T exonic ORF1a. synonymous SNV
ORF1a:cds-YP_009724389.1:exon1:c.C8517T:p.S2839S,ORF1a:cds-YP_009725295.1:exon1:
c.C8517T:p.S2839S -1.35 0.271811 1 0.67 T . Lumenal .
. . . .
.
1 8818 8818 C T exonic ORF1a. synonymous SNV
ORF1a:cds-YP_009724389.1:exon1:c.C8553T:p.C2851C,ORF1a:cds-YP_009725295.1:exon1:
c.C8553T:p.C2851C -1.19 0.615276 1 1 T . Lumenal .
. . . .
.
1 8829 8829 C T exonic ORF1a. nonsynonymous SNV
ORF1a:cds-YP_009724389.1:exon1:c.C8564T:p.A2855V,ORF1a:cds-YP_009725295.1:exon1:
c.C8564T:p.A2855V 1.65 3.2943 1 1 T . Lumenal . .
. . . .

1 8854 8854 T C exonic ORF1a. synonymous SNV
ORF1a:cds-YP_009724389.1:exon1:c.T8589C:p.G2863G,ORF1a:cds-YP_009725295.1:exon1:
c.T8589C:p.G2863G -3.3 -1.5142 0.716535 1 T . Lumenal
. . . . . orf1ab_REVGFVVPGL 0.05 . . .
. . . . .
1 8882 8882 A C exonic ORF1a. nonsynonymous SNV
ORF1a:cds-YP_009724389.1:exon1:c.A8617C:p.I2873L,ORF1a:cds-YP_009725295.1:exon1:c.
A8617C:p.I2873L 0.695 1.71436 0.968504 0.02 D . Lumenal
. . . orf1ab polyprotein_GTILRTTNGDFLHFL 20 orf1ab_VVPGLPGTIL
0.16 . . . .
1 8917 8917 C T exonic ORF1a. synonymous SNV
ORF1a:cds-YP_009724389.1:exon1:c.C8652T:p.F2884F,ORF1a:cds-YP_009725295.1:exon1:c
.C8652T:p.F2884F -0.891 0.546583 1 1 T . Lumenal .
. . orf1ab polyprotein_GTILRTTNGDFLHFL 20 orf1ab_FLPRVFSAV 0.1
. . . .

1 8964 8964 C T exonic ORF1a. nonsynonymous SNV
ORF1a:cds-YP_009724389.1:exon1:c.C8699T:p.S2900L,ORF1a:cds-YP_009725295.1:exon1:c
.C8699T:p.S2900L 1.65 3.2943 0.984252 0.1 T . Lumenal .
. . orf1ab polyprotein_AVGNICYTPSKLIEY 18 . . . .
nCoV-2019_29_RIGHT . . . . .
1 9042 9042 C T exonic ORF1a. nonsynonymous SNV
ORF1a:cds-YP_009724389.1:exon1:c.C8777T:p.S2926F,ORF1a:cds-YP_009725295.1:exon1:c
.C8777T:p.S2926F -2.18 -0.140346 0.976378 0.03 D . Lumenal
. . . . . orf1ab_TIFKDASGK 0.1 . . . .
. . . .
1 9056 9056 C T exonic ORF1a. nonsynonymous SNV
ORF1a:cds-YP_009724389.1:exon1:c.C8791T:p.P2931S,ORF1a:cds-YP_009725295.1:exon1:
c.C8791T:p.P2931S 1.65 3.2943 1 0 D . Lumenal . .
. . . orf1ab_DASGKPVPY 0.07 . . . . . .
. .
1 9085 9085 A G exonic ORF1a. synonymous SNV
ORF1a:cds-YP_009724389.1:exon1:c.A8820G:p.E2940E,ORF1a:cds-YP_009725295.1:exon1:
c.A8820G:p.E2940E -0.363 0.683968 1 1 T . Lumenal .
. . . . orf1ab_NVLEGSVAY 0.01 . . . . .
. . .
1 9157 9157 T C exonic ORF1a. synonymous SNV
ORF1a:cds-YP_009724389.1:exon1:c.T8892C:p.F2964F,ORF1a:cds-YP_009725295.1:exon1:c
.T8892C:p.F2964F -0.385 0.890047 1 1 T . Lumenal .
. . orf1ab polyprotein_DTRYVLMDGSIIQFP 6.2 orf1ab_SIIQFPNTY 0.1
. . . .
1 9207 9207 C T exonic ORF1a. nonsynonymous SNV
ORF1a:cds-YP_009724389.1:exon1:c.C8942T:p.S2981F,ORF1a:cds-YP_009725295.1:exon1:c
.C8942T:p.S2981F 1.65 3.2943 1 0.01 D . Lumenal . .
. . . . nCoV-2019_31_LEFT . . .
. .
1 9291 9291 A G exonic ORF1a. nonsynonymous SNV
ORF1a:cds-YP_009724389.1:exon1:c.A9026G:p.D3009G,ORF1a:cds-YP_009725295.1:exon1:
c.A9026G:p.D3009G 1.65 2.23647 0.992134 0.05 D . Lumenal
. . . orf1ab polyprotein_SGRWVLNNDYYRSLP 19 . . .
. . . .
1 9341 9341 T C exonic ORF1a. synonymous SNV
ORF1a:cds-YP_009724389.1:exon1:c.T9076C:p.L3026L,ORF1a:cds-YP_009725295.1:exon1:c
.T9076C:p.L3026L 0.627 0.784937 0.952803 1 T . Lumenal
. . . orf1ab polyprotein_GVFCGVDAVNLLTNM 20 . . .
. . . .
1 9389 9389 G A exonic ORF1a. nonsynonymous SNV
ORF1a:cds-YP_009724389.1:exon1:c.G9124A:p.D3042N,ORF1a:cds-YP_009725295.1:exon1:
c.G9124A:p.D3042N 1.65 4.256 1 0.09 T . Lumenal . .

. . . .

1 9430 9430 C T exonic ORF1a. synonymous SNV
ORF1a:cds-YP_009724389.1:exon1:c.C9165T:p.I3055I,ORF1a:cds-YP_009725295.1:exon1:c.
C9165T:p.I3055I -1.02 0.595606 0.992134 0.96 T . . .
Transmembrane . . . orf1ab_IVAGGIVAI 0.14 . . .
. . . . .
1 9451 9451 C T exonic ORF1a. synonymous SNV
ORF1a:cds-YP_009724389.1:exon1:c.C9186T:p.Y3062Y,ORF1a:cds-YP_009725295.1:exon1:
c.C9186T:p.Y3062Y 0.787 1.98403 1 1 T . . .
Transmembrane . orf1ab polyprotein_AYYFMRFRRAFGEYS 17 . .
. . . .
1 9479 9479 G T exonic ORF1a. nonsynonymous SNV
ORF1a:cds-YP_009724389.1:exon1:c.G9214T:p.G3072C,ORF1a:cds-YP_009725295.1:exon1:
c.G9214T:p.G3072C 1.65 4.256 1 0 D . . . . .
orf1ab polyprotein_AYYFMRFRRAFGEYS 17 orf1ab_GEYSHVVAFNTLL -1 .
. nCoV-2019_32_LEFT. . . . . .
1 9541 9541 C T exonic ORF1a. synonymous SNV
ORF1a:cds-YP_009724389.1:exon1:c.C9276T:p.L3092L,ORF1a:cds-YP_009725295.1:exon1:c
.C9276T:p.L3092L -1.01 1.22671 1 1 T . . .
Transmembrane . orf1ab polyprotein_NTLLFLMSFTVLCLT 17 . .
. . . .
1 9565 9565 C T exonic ORF1a. synonymous SNV
ORF1a:cds-YP_009724389.1:exon1:c.C9300T:p.F3100F,ORF1a:cds-YP_009725295.1:exon1:c
.C9300T:p.F3100F -3.3 -0.477268 0.575228 0.35 T . . .
Transmembrane . orf1ab polyprotein_LMSFTVLCLTPVYSF 19
orf1ab_FLPGVYSVIY -1 . . nCoV-2019_31_RIGHT . . .
. .
1 9611 9611 C T exonic ORF1a. nonsynonymous SNV
ORF1a:cds-YP_009724389.1:exon1:c.C9346T:p.L3116F,ORF1a:cds-YP_009725295.1:exon1:c
.C9346T:p.L3116F 0.474 0.469386 1 1 T . . .
Transmembrane . orf1ab polyprotein_YLTFYLTNDVSFLAH 7.4
orf1ab_TFYLTNDVSFL -1 . . . .

1 9615 9615 C T exonic ORF1a. nonsynonymous SNV
ORF1a:cds-YP_009724389.1:exon1:c.C9350T:p.T3117I,ORF1a:cds-YP_009725295.1:exon1:c.
C9350T:p.T3117I 1.65 3.30935 1 0.03 D . . .
Transmembrane . orf1ab polyprotein_YLTFYLTNDVSFLAH 7.4
orf1ab_TFYLTNDVSFL -1 . . . .

1 9653 9653 G T exonic ORF1a. nonsynonymous SNV
ORF1a:cds-YP_009724389.1:exon1:c.G9388T:p.V3130F,ORF1a:cds-YP_009725295.1:exon1:
c.G9388T:p.V3130F 1.65 4.256 1 0 D . . .

Transmembrane . orf1ab polyprotein_SFLAHIQWMVMFTPL 18
orf1ab_FLAHIQWMV 0.03 . . . .

1 9679 9679 C T exonic ORF1a. synonymous SNV
ORF1a:cds-YP_009724389.1:exon1:c.C9414T:p.F3138F,ORF1a:cds-YP_009725295.1:exon1:c
.C9414T:p.F3138F -3.3 -0.0354961 0.992134 0.36 T . . .
Transmembrane . orf1ab polyprotein_IQWMVMFTPLVPFWI 13
orf1ab_MFTPLVPFW -1 . . . .

1 9693 9693 C T exonic ORF1a. nonsynonymous SNV
ORF1a:cds-YP_009724389.1:exon1:c.C9428T:p.A3143V,ORF1a:cds-YP_009725295.1:exon1:
c.C9428T:p.A3143V 0.454 0.595606 0.386441 1 T . . .
Transmembrane . orf1ab polyprotein_VPFWITIAYIICIST 7.3
orf1ab_VPFWITIAY 0.01 . . . .

1 9758 9758 G T exonic ORF1a. nonsynonymous SNV
ORF1a:cds-YP_009724389.1:exon1:c.G9493T:p.V3165L,ORF1a:cds-YP_009725295.1:exon1:c
.G9493T:p.V3165L 1.65 4.256 1 0 D . . .
Transmembrane . orf1ab polyprotein_KHFYWFFSNYLKRRV 2
orf1ab_NYLKRRVVF -1 . . . .

1 9810 9810 C T exonic ORF1a. nonsynonymous SNV
ORF1a:cds-YP_009724389.1:exon1:c.C9545T:p.T3182I,ORF1a:cds-YP_009725295.1:exon1:c.
C9545T:p.T3182I 1.65 3.30935 1 0.22 T . . .
Transmembrane . orf1ab polyprotein_CTFLLNKEMYLKLRS 16
orf1ab_ALCTFLLNK 0.14 . . . .

1 9856 9856 G T exonic ORF1a. synonymous SNV
ORF1a:cds-YP_009724389.1:exon1:c.G9591T:p.V3197V,ORF1a:cds-YP_009725295.1:exon1:
c.G9591T:p.V3197V -0.683 0.0276142 0.976402 0.73 T . . .
. . orf1ab polyprotein_NKEMYLKLRSDVLLP 11 orf1ab_YLKLRSDVL 0.01
. . nCoV-2019_32_RIGHT . . . . .

1 9857 9857 C T exonic ORF1a. synonymous SNV
ORF1a:cds-YP_009724389.1:exon1:c.C9592T:p.L3198L,ORF1a:cds-YP_009725295.1:exon1:c
.C9592T:p.L3198L -0.22 1.1636 0.976402 1 T . . . .
. orf1ab polyprotein_NKEMYLKLRSDVLLP 11 orf1ab_YLKLRSDVL 0.01 .
. nCoV-2019_32_RIGHT . . . . .

1 9861 9861 T C exonic ORF1a. nonsynonymous SNV
ORF1a:cds-YP_009724389.1:exon1:c.T9596C:p.L3199S,ORF1a:cds-YP_009725295.1:exon1:c
.T9596C:p.L3199S 1.65 2.17336 0.952803 0 D . . .
. . orf1ab polyprotein_NKEMYLKLRSDVLLP 11 orf1ab_LPLTQYNRY 0.03

. . . .
1 9866 9866 C T exonic ORF1a. nonsynonymous SNV
ORF1a:cds-YP_009724389.1:exon1:c.C9601T:p.L3201F,ORF1a:cds-YP_009725295.1:exon1:c
.C9601T:p.L3201F 0.737 1.7947 1 0.13 T . . . . .
. . orf1ab_LPLTQYNRY 0.03 . . . .
.
1 9870 9870 C T exonic ORF1a. nonsynonymous SNV
ORF1a:cds-YP_009724389.1:exon1:c.C9605T:p.T3202M,ORF1a:cds-YP_009725295.1:exon1:
c.C9605T:p.T3202M 1.65 3.30935 1 0.02 D . . . .
. . . orf1ab_LPLTQYNRY 0.03 . . . . . .
. .
1 9913 9913 T C exonic ORF1a. synonymous SNV
ORF1a:cds-YP_009724389.1:exon1:c.T9648C:p.Y3216Y,ORF1a:cds-YP_009725295.1:exon1:
c.T9648C:p.Y3216Y 0.446 0.974268 1 1 T . . . .
. . . .

1 9928 9928 G A exonic ORF1a. nonsynonymous SNV
ORF1a:cds-YP_009724389.1:exon1:c.G9663A:p.M3221I,ORF1a:cds-YP_009725295.1:exon1:c
.G9663A:p.M3221I -3.3 -0.0354961 0.992134 0.04 D . . .
. . . . orf1ab_GAMDTTSYR 0.09 . . . . .
. . .
1 9928 9928 G T exonic ORF1a. nonsynonymous SNV
ORF1a:cds-YP_009724389.1:exon1:c.G9663T:p.M3221I,ORF1a:cds-YP_009725295.1:exon1:c
.G9663T:p.M3221I -3.3 -0.0354961 0.992134 0.04 D . . .
. . . . orf1ab_GAMDTTSYR 0.09 . . . . .
. . .
1 9943 9943 C T exonic ORF1a. synonymous SNV
ORF1a:cds-YP_009724389.1:exon1:c.C9678T:p.Y3226Y,ORF1a:cds-YP_009725295.1:exon1:
c.C9678T:p.Y3226Y -0.78 0.406276 1 1 T . . . .
. . . orf1ab_GAMDTTSYR 0.09 . . . . . .
. .
1 9962 9962 C T exonic ORF1a. nonsynonymous SNV
ORF1a:cds-YP_009724389.1:exon1:c.C9697T:p.H3233Y,ORF1a:cds-YP_009725295.1:exon1:
c.C9697T:p.H3233Y 1.65 3.30935 1 0 D . . . .
. . . .

1 9996 9996 C T exonic ORF1a. nonsynonymous SNV
ORF1a:cds-YP_009724389.1:exon1:c.C9731T:p.S3244L,ORF1a:cds-YP_009725295.1:exon1:c
.C9731T:p.S3244L 1.65 3.30935 0.882008 0.01 D . . .
. . . . orf1ab_FSNSGSDVLY 0.02 . . . .
. . . .
1 10039 10039 C T exonic ORF1a. synonymous SNV
ORF1a:cds-YP_009724389.1:exon1:c.C9774T:p.T3258T,ORF1a:cds-YP_009725295.1:exon1:c

.C9774T:p.T3258T	-3.3	-0.351047	0.992134	1	T	.	.	.
.	.	.	.	orf1ab_SAVLQSGFRK	-1	.	.	.	.
.	.	.	.
1	10138	10138	C	T	exonic	ORF1a.	synonymous SNV
ORF1a:cds-YP_009724389.1:exon1:c.C9873T:p.N3291N,ORF1a:cds-YP_009725295.1:exon1:
c.C9873T:p.N3291N	-3.3	-0.477268	0.984268	1	T	.
Peptidase_C30	.	.	.	.	.	.	.	.	.
.	.	.	.	.
1	10156	10156	C	T	exonic	ORF1a.	synonymous SNV
ORF1a:cds-YP_009724389.1:exon1:c.C9891T:p.D3297D,ORF1a:cds-YP_009725295.1:exon1:
c.C9891T:p.D3297D	-2.35	0.0276142	0.992134	0.74	T	.
Peptidase_C30	.	.	.	.	.	.	.	.
nCoV-2019_33_RIGHT	.	.	.	.	.
1	10255	10255	G	A	exonic	ORF1a.	synonymous SNV
ORF1a:cds-YP_009724389.1:exon1:c.G9990A:p.L3330L,ORF1a:cds-YP_009725295.1:exon1:c
.G9990A:p.L3330L	-2.29	0.0580472	1	0.81	T	.	Peptidase_C30
.	.	.	orf1ab polyprotein_NYEDLLIRKSNHNFL	14
orf1ab_FLVQAGNVQL	0.14	.	.	.	.	.	.	.	.

1	10266	10266	G	T	exonic	ORF1a.	nonsynonymous SNV
ORF1a:cds-YP_009724389.1:exon1:c.G10001T:p.G3334V,ORF1a:cds-YP_009725295.1:exon1
:c.G10001T:p.G3334V	1.65	4.256	1	0.01	D	.	Peptidase_C30
.	.	.	orf1ab polyprotein_NHNFLVQAGNVQLRV	15
orf1ab_FLVQAGNVQL	0.14	.	.	.	.	.	.	.	.

1	10285	10285	T	C	exonic	ORF1a.	synonymous SNV
ORF1a:cds-YP_009724389.1:exon1:c.T10020C:p.V3340V,ORF1a:cds-YP_009725295.1:exon1
:c.T10020C:p.V3340V	-0.808	0.113283	0.992126	1	T	.
Peptidase_C30	.	.	.	orf1ab polyprotein_NHNFLVQAGNVQLRV	15
orf1ab_QLRVIGHSM	0.14	.	.	.	.	.	.	.	.

1	10369	10369	C	T	exonic	ORF1a.	synonymous SNV
ORF1a:cds-YP_009724389.1:exon1:c.C10104T:p.R3368R,ORF1a:cds-YP_009725295.1:exon1
:c.C10104T:p.R3368R	-2.22	-0.107661	0.992126	0.57	T	.
Peptidase_C30	.	.	.	orf1ab polyprotein_TPKYKFVRIQPGQTF	8
orf1ab_TPKYKFVRI	0.02	.	.	nCoV-2019_35_LEFT	.	.	.	.
.
1	10376	10376	C	T	exonic	ORF1a.	nonsynonymous SNV
ORF1a:cds-YP_009724389.1:exon1:c.C10111T:p.P3371S,ORF1a:cds-YP_009725295.1:exon1
:c.C10111T:p.P3371S	0.787	1.93608	1	0.06	T	.
Peptidase_C30	.	.	.	orf1ab polyprotein_TPKYKFVRIQPGQTF	8
orf1ab_KYKFVRIQPGQTF	-1	.	.	nCoV-2019_35_LEFT	.	.	.
.	.

1	10444	10444	G	A	exonic	ORF1a.	nonsynonymous SNV
ORF1a:cds-YP_009724389.1:exon1:c.G10179A:p.M3393I,ORF1a:cds-YP_009725295.1:exon1:
c.G10179A:p.M3393I	1.65	4.256	1	0.01	D	.	Peptidase_C30	.
.	.	.	.	orf1ab_SPSGVYQCAM	0.06	.	.
nCoV-2019_34_RIGHT	.	.	.	.	.
1	10455	10455	T	C	exonic	ORF1a.	nonsynonymous SNV
ORF1a:cds-YP_009724389.1:exon1:c.T10190C:p.F3397S,ORF1a:cds-YP_009725295.1:exon1
:c.T10190C:p.F3397S-0.954	0.00281102	0	0.39	T	.	Peptidase_C30
.	.	.	.	.	orf1ab_AMRPNFTIK	0.04	.	.
nCoV-2019_34_RIGHT	.	.	.	.	.
1	10476	10476	T	C	exonic	ORF1a.	nonsynonymous SNV
ORF1a:cds-YP_009724389.1:exon1:c.T10211C:p.L3404P,ORF1a:cds-YP_009725295.1:exon1:
c.T10211C:p.L3404P	1.65	2.21226	1	0	D	.	Peptidase_C30
.	.	.	.	.	.	.	.	.	.	.	.	.
.	.
1	10486	10486	A	G	exonic	ORF1a.	synonymous SNV
ORF1a:cds-YP_009724389.1:exon1:c.A10221G:p.S3407S,ORF1a:cds-YP_009725295.1:exon1
:c.A10221G:p.S3407S	-0.968	0.334228	1	1	T	.
Peptidase_C30	.	.	.	.	.	.	.	.	.	.
.	.	.	.	.
1	10543	10543	C	T	exonic	ORF1a.	synonymous SNV
ORF1a:cds-YP_009724389.1:exon1:c.C10278T:p.H3426H,ORF1a:cds-YP_009725295.1:exon1
:c.C10278T:p.H3426H	-3.3	-0.549551	0.968504	1	T	.
Peptidase_C30	.	.	.	.	.	.	.	.	.	.
.	.	.	.	.
1	10582	10582	C	T	exonic	ORF1a.	synonymous SNV
ORF1a:cds-YP_009724389.1:exon1:c.C10317T:p.D3439D,ORF1a:cds-YP_009725295.1:exon1
:c.C10317T:p.D3439D	-1.08	0.720882	1	1	T	.
Peptidase_C30	.	.	.	.	.	orf1ab_TGVHAGTDLEGNFY	0.01
.	.	.	.	.	.	.	.
1	10615	10615	C	T	exonic	ORF1a.	synonymous SNV
ORF1a:cds-YP_009724389.1:exon1:c.C10350T:p.D3450D,ORF1a:cds-YP_009725295.1:exon1
:c.C10350T:p.D3450D	-0.218	1.16277	1	1	T	.
Peptidase_C30	.	.	.	.	.	.	.	.	.	.
.	.	.	.	.
1	10626	10626	C	T	exonic	ORF1a.	nonsynonymous SNV
ORF1a:cds-YP_009724389.1:exon1:c.C10361T:p.A3454V,ORF1a:cds-YP_009725295.1:exon1
:c.C10361T:p.A3454V	1.65	3.31698	1	0.24	T	.
Peptidase_C30	.	.	.	.	.	.	.	.	.	.
.	.	.	.	.
1	10642	10642	G	T	exonic	ORF1a.	synonymous SNV
ORF1a:cds-YP_009724389.1:exon1:c.G10377T:p.T3459T,ORF1a:cds-YP_009725295.1:exon1
:c.G10377T:p.T3459T-2.87	0.16852	1	1	T	.	Peptidase_C30

. . . .
. .
1 10717 10717 T C exonic ORF1a. synonymous SNV
ORF1a:cds-YP_009724389.1:exon1:c.T10452C:p.N3484N,ORF1a:cds-YP_009725295.1:exon1
:c.T10452C:p.N3484N -2.17 0.0580472 0.992126 0.63 T .
Peptidase_C30 . . . orf1ab polyprotein_LYAAVINGDRWFLNR 19
orf1ab_FLNRFTTTL 0.03 . . . .

1 10726 10726 C T exonic ORF1a. synonymous SNV
ORF1a:cds-YP_009724389.1:exon1:c.C10461T:p.T3487T,ORF1a:cds-YP_009725295.1:exon1:
c.C10461T:p.T3487T -3.3 0.00281102 0.992126 1 T .
Peptidase_C30 . . . . . orf1ab_FLNRFTTTL 0.03 .
. . . .
1 10755 10755 C T exonic ORF1a. nonsynonymous SNV
ORF1a:cds-YP_009724389.1:exon1:c.C10490T:p.A3497V,ORF1a:cds-YP_009725295.1:exon1
:c.C10490T:p.A3497V 1.65 3.31698 1 0 D .
Peptidase_C30 . . . .
nCoV-2019_35_RIGHT . . . . .
1 10761 10761 A G exonic ORF1a. nonsynonymous SNV
ORF1a:cds-YP_009724389.1:exon1:c.A10496G:p.K3499R,ORF1a:cds-YP_009725295.1:exon1
:c.A10496G:p.K3499R 1.65 2.2675 1 0.13 T . Peptidase_C30
. . . . nCoV-2019_35_RIGHT
. . . . .
1 10769 10769 T C exonic ORF1a. nonsynonymous SNV
ORF1a:cds-YP_009724389.1:exon1:c.T10504C:p.Y3502H,ORF1a:cds-YP_009725295.1:exon1
:c.T10504C:p.Y3502H 1.65 2.21226 1 0 D .
Peptidase_C30 . . . .
. . . . .
1 10793 10793 G T exonic ORF1a. nonsynonymous SNV
ORF1a:cds-YP_009724389.1:exon1:c.G10528T:p.V3510F,ORF1a:cds-YP_009725295.1:exon1
:c.G10528T:p.V3510F 0.717 2.54368 1 0 D .
Peptidase_C30 . . . .
. . . . .
1 10798 10798 C T exonic ORF1a. synonymous SNV
ORF1a:cds-YP_009724389.1:exon1:c.C10533T:p.D3511D,ORF1a:cds-YP_009725295.1:exon1
:c.C10533T:p.D3511D 1.65 3.31698 1 1 T .
Peptidase_C30 . . . .
. . . . .
1 10833 10833 C T exonic ORF1a. nonsynonymous SNV
ORF1a:cds-YP_009724389.1:exon1:c.C10568T:p.A3523V,ORF1a:cds-YP_009725295.1:exon1
:c.C10568T:p.A3523V 0.788 1.93608 0.968504 0.02 D .
Peptidase_C30 . . . . . orf1ab_AVLDMCASLK 0.08
. . . .

1	10843	10843	T	C	exonic	ORF1a.	synonymous SNV
ORF1a:cds-YP_009724389.1:exon1:c.T10578C:p.D3526D,ORF1a:cds-YP_009725295.1:exon1
:c.T10578C:p.D3526D	1.65	2.21226	1	0.5	T	.
Peptidase_C30	.	.	.	.	.	orf1ab_AVLDMCASLK	0.08
.	.	.	.	.	.	.	.
1	10855	10855	A	C	exonic	ORF1a.	synonymous SNV
ORF1a:cds-YP_009724389.1:exon1:c.A10590C:p.S3530S,ORF1a:cds-YP_009725295.1:exon1
:c.A10590C:p.S3530S	-3.3	-0.549551	0.795276	0.09	T	.
Peptidase_C30	.	.	.	.	.	orf1ab_AVLDMCASLK	0.08
.	.	.	.	.	.	.	.
1	10969	10969	C	T	exonic	ORF1a.	synonymous SNV
ORF1a:cds-YP_009724389.1:exon1:c.C10704T:p.F3568F,ORF1a:cds-YP_009725295.1:exon1:
c.C10704T:p.F3568F	-3.3	-0.107661	0.992126	0.22	T	.
Peptidase_C30	.	.	.	.	.	orf1ab_VTFQSAVKR	0.09	.
.	.	.	.	.	.	.
1	11002	11002	A	G	exonic	ORF1a.	synonymous SNV
ORF1a:cds-YP_009724389.1:exon1:c.A10737G:p.T3579T,ORF1a:cds-YP_009725295.1:exon1
:c.A10737G:p.T3579T	-2.37	0.0580472	0.992126	0.34	T	.	.
.	.	.	.	.	.	.	.	.	nCoV-2019_37_LEFT.
.	.	.	.	.
1	11020	11020	C	T	exonic	ORF1a.	synonymous SNV
ORF1a:cds-YP_009724389.1:exon1:c.C10755T:p.L3585L,ORF1a:cds-YP_009725295.1:exon1:
c.C10755T:p.L3585L	-3.3	-0.770496	0.700787	0.69	T	.	.	.
.	.	orf1ab polyprotein_HWLLLTILTSLLVLV	19	.	.	.	.
nCoV-2019_37_LEFT.	.	.	.	.
1	11029	11029	G	T	exonic	ORF1a.	nonsynonymous SNV
ORF1a:cds-YP_009724389.1:exon1:c.G10764T:p.L3588F,ORF1a:cds-YP_009725295.1:exon1:
c.G10764T:p.L3588F	1.65	4.256	1	0.05	D	.	.	.
Transmembrane	.	orf1ab polyprotein_HWLLLTILTSLLVLV	19
orf1ab_ILTSLLVLV	0.14	.	.	.	.	.	.	.	.

1	11083	11083	G	T	exonic	ORF1a.	nonsynonymous SNV
ORF1a:cds-YP_009724389.1:exon1:c.G10818T:p.L3606F,ORF1a:cds-YP_009725295.1:exon1:
c.G10818T:p.L3606F	-3.3	-1.32286	0.92126	0.01	D	.	.	.
Transmembrane	.	orf1ab polyprotein_SLFFFLYENAFLPFA	18
orf1ab_FLYENAFLPFAM	-1	.	.	.	.	.	.	homoplasic
position	.
1	11109	11109	C	T	exonic	ORF1a.	nonsynonymous SNV
ORF1a:cds-YP_009724389.1:exon1:c.C10844T:p.A3615V,ORF1a:cds-YP_009725295.1:exon1
:c.C10844T:p.A3615V	1.65	3.31698	1	0.1	T	.	.	.
Transmembrane	.	orf1ab polyprotein_SLFFFLYENAFLPFA	18
orf1ab_FLYENAFLPFAM	-1	.	.	.	.	.	.	.	.

1	11149	11149	T	C	exonic	ORF1a.	synonymous SNV
ORF1a:cds-YP_009724389.1:exon1:c.T10884C:p.F3628F,ORF1a:cds-YP_009725295.1:exon1:
c.T10884C:p.F3628F	-0.942	0.389465	1	0.15	T	.	.	.
Transmembrane	.	orf1ab polyprotein_MGIIAMSAFAMMFVK	9.2
orf1ab_SAFAMMFVK	0.09	.	.	.	.	.	.	.	.

1	11152	11152	C	T	exonic	ORF1a.	synonymous SNV
ORF1a:cds-YP_009724389.1:exon1:c.C10887T:p.V3629V,ORF1a:cds-YP_009725295.1:exon1
:c.C10887T:p.V3629V	-1.02	0.0580472	1	1	T	.	.	.
Transmembrane	.	orf1ab polyprotein_MGIIAMSAFAMMFVK	9.2
orf1ab_SAFAMMFVK	0.09	.	.	.	.	.	.	.	.

1	11195	11195	C	T	exonic	ORF1a.	nonsynonymous SNV
ORF1a:cds-YP_009724389.1:exon1:c.C10930T:p.L3644F,ORF1a:cds-YP_009725295.1:exon1:
c.C10930T:p.L3644F	0.68	1.60466	1	0.03	D	.	.	.
Transmembrane	.	orf1ab polyprotein_LCLFLLPSLATVAYF	18
orf1ab_FLLPSLATVAY	-1	.	.	.	.	.	.	.	.

1	11208	11208	C	T	exonic	ORF1a.	nonsynonymous SNV
ORF1a:cds-YP_009724389.1:exon1:c.C10943T:p.A3648V,ORF1a:cds-YP_009725295.1:exon1
:c.C10943T:p.A3648V	1.65	3.31698	1	0.05	D	.	.	.
Transmembrane	.	orf1ab polyprotein_LCLFLLPSLATVAYF	18
orf1ab_FLLPSLATVAY	-1	.	.	.	.	.	.	.	.

1	11222	11222	G	T	exonic	ORF1a.	nonsynonymous SNV
ORF1a:cds-YP_009724389.1:exon1:c.G10957T:p.V3653F,ORF1a:cds-YP_009725295.1:exon1
:c.G10957T:p.V3653F	0.646	2.2675	1	0	D	.	.	.
Transmembrane	.	orf1ab polyprotein_NMVYMPASWVMRIMT	13
orf1ab_VYMPASWVMRIMTW	-1	.	.	.	.	.	.	.
.
1	11277	11277	A	G	exonic	ORF1a.	nonsynonymous SNV
ORF1a:cds-YP_009724389.1:exon1:c.A11012G:p.D3671G,ORF1a:cds-YP_009725295.1:exon
1:c.A11012G:p.D3671G	1.65	2.26436	1	0.37	T	.	Lumenal
.	.	.	.	.	orf1ab_WLDMVDTSL	0.12	.	.	.	.
.	.	.	.
1	11287	11287	G	T	exonic	ORF1a.	nonsynonymous SNV
ORF1a:cds-YP_009724389.1:exon1:c.G11022T:p.L3674F,ORF1a:cds-YP_009725295.1:exon1:
c.G11022T:p.L3674F	0.746	2.7451	1	0.05	D	.	.	.
Transmembrane	.	.	.	orf1ab_WLDMVDTSL	0.12	.	.	.
.	.	.	.	.
1	11298	11298	A	G	exonic	ORF1a.	nonsynonymous SNV
ORF1a:cds-YP_009724389.1:exon1:c.A11033G:p.K3678R,ORF1a:cds-YP_009725295.1:exon1
:c.A11033G:p.K3678R	0.695	1.71494	0.992157	0.61	T	.	.

. Transmembrane . . . .
. . . .
1 11511 11511 C T exonic ORF1a. nonsynonymous SNV
ORF1a:cds-YP_009724389.1:exon1:c.C11246T:p.T3749I,ORF1a:cds-YP_009725295.1:exon1:
c.C11246T:p.T3749I 1.65 3.29452 1 0.18 T . . .
Transmembrane . orf1ab polyprotein_LIISVTSNYSGVVTT 11
orf1ab_NYSGVVTTVMF 0.08 . . . .

1 11521 11521 G T exonic ORF1a. nonsynonymous SNV
ORF1a:cds-YP_009724389.1:exon1:c.G11256T:p.M3752I,ORF1a:cds-YP_009725295.1:exon1:
c.G11256T:p.M3752I 1.65 4.256 1 0.3 T . Lumenal . .
. . . orf1ab_NYSGVVTTVMF 0.08 . . . . .
. . .
1 11535 11535 G T exonic ORF1a. nonsynonymous SNV
ORF1a:cds-YP_009724389.1:exon1:c.G11270T:p.G3757V,ORF1a:cds-YP_009725295.1:exon1
:c.G11270T:p.G3757V 0.746 0.959496 1 0.24 T . Lumenal
. . . . . orf1ab_MFLARGIVF -1 . . . .
. . . .
1 11572 11572 C T exonic ORF1a. synonymous SNV
ORF1a:cds-YP_009724389.1:exon1:c.C11307T:p.F3769F,ORF1a:cds-YP_009725295.1:exon1:
c.C11307T:p.F3769F -3.3 -0.414047 0.584346 0.12 T . Lumenal
. . . orf1ab polyprotein_CPIFFITGNTLQCIM 20 orf1ab_IFFITGNTL
0.09 . . nCoV-2019_39_LEFT . . . . .

1 11580 11580 C T exonic ORF1a. nonsynonymous SNV
ORF1a:cds-YP_009724389.1:exon1:c.C11315T:p.T3772I,ORF1a:cds-YP_009725295.1:exon1:
c.C11315T:p.T3772I 1.65 3.29452 1 0.64 T . Lumenal .
. . orf1ab polyprotein_CPIFFITGNTLQCIM 20 orf1ab_IFFITGNTL 0.09
. . nCoV-2019_39_LEFT . . . . .

1 11595 11595 A G exonic ORF1a. nonsynonymous SNV
ORF1a:cds-YP_009724389.1:exon1:c.A11330G:p.Q3777R,ORF1a:cds-YP_009725295.1:exon
1:c.A11330G:p.Q3777R 1.65 2.26436 1 0.16 T . Lumenal
. . . orf1ab polyprotein_CPIFFITGNTLQCIM 20 . . .
. . . .
1 11692 11692 T C exonic ORF1a. synonymous SNV
ORF1a:cds-YP_009724389.1:exon1:c.T11427C:p.G3809G,ORF1a:cds-YP_009725295.1:exon
1:c.T11427C:p.G3809G 1.65 2.19569 1 1 T . . .
. . orf1ab polyprotein_NRYFRLTLGVYDYLV 19 . . . .
nCoV-2019_38_RIGHT . . . . .
1 11700 11700 A G exonic ORF1a. nonsynonymous SNV
ORF1a:cds-YP_009724389.1:exon1:c.A11435G:p.D3812G,ORF1a:cds-YP_009725295.1:exon
1:c.A11435G:p.D3812G 1.65 2.26436 1 0.01 D . . .

. . orf1ab polyprotein_NRYFRLTLGVYDYLV 19 orf1ab_YDYLVSTQEF
-1 . . . .
1 11704 11704 C T exonic ORF1a. synonymous SNV
ORF1a:cds-YP_009724389.1:exon1:c.C11439T:p.Y3813Y,ORF1a:cds-YP_009725295.1:exon1
:c.C11439T:p.Y3813Y -3.27 -0.482724 0.984315 1 T . .
. . . orf1ab polyprotein_NRYFRLTLGVYDYLV 19
orf1ab_YDYLVSTQEF -1 . . . .

1 11747 11747 C T exonic ORF1a. synonymous SNV
ORF1a:cds-YP_009724389.1:exon1:c.C11482T:p.L3828L,ORF1a:cds-YP_009725295.1:exon1:
c.C11482T:p.L3828L 0.776 1.92098 1 1 T . . . .
. orf1ab polyprotein_STQEFRYMNSQGLLP 3.3 . . . . .
. . . . .
1 11758 11758 C T exonic ORF1a. synonymous SNV
ORF1a:cds-YP_009724389.1:exon1:c.C11493T:p.P3831P,ORF1a:cds-YP_009725295.1:exon1
:c.C11493T:p.P3831P -0.864 0.0666929 1 1 T . . .
. . . .
.
1 11761 11761 G T exonic ORF1a. nonsynonymous SNV
ORF1a:cds-YP_009724389.1:exon1:c.G11496T:p.K3832N,ORF1a:cds-YP_009725295.1:exon1
:c.G11496T:p.K3832N 1.65 4.256 1 0.04 D . . . .
. . . .

1 11782 11782 A G exonic ORF1a. synonymous SNV
ORF1a:cds-YP_009724389.1:exon1:c.A11517G:p.K3839K,ORF1a:cds-YP_009725295.1:exon1
:c.A11517G:p.K3839K -1.57 0.753465 1 0.59 T . . .
. . orf1ab polyprotein_DAFKLNIKLLGVGGK 15 . . . .
. . . . . .
1 11801 11801 G A exonic ORF1a. nonsynonymous SNV
ORF1a:cds-YP_009724389.1:exon1:c.G11536A:p.G3846S,ORF1a:cds-YP_009725295.1:exon
1:c.G11536A:p.G3846S 1.65 4.256 1 0 D . . . .
. orf1ab polyprotein_DAFKLNIKLLGVGGK 15 . . . . .
. . . . .
1 11824 11824 C T exonic ORF1a. synonymous SNV
ORF1a:cds-YP_009724389.1:exon1:c.C11559T:p.I3853I,ORF1a:cds-YP_009725295.1:exon1:c
.C11559T:p.I3853I -3.15 -0.551402 0.90589 1 T . . .
. . . .
.
1 11866 11866 C T exonic ORF1a. synonymous SNV
ORF1a:cds-YP_009724389.1:exon1:c.C11601T:p.C3867C,ORF1a:cds-YP_009725295.1:exon1
:c.C11601T:p.C3867C 1.65 3.29452 1 1 T . . .
. . orf1ab polyprotein_KCTSVVLLSVLQQLR 13 . . . .
nCoV-2019_40_LEFT . . . . .

1 11916 11916 C T exonic ORF1a. nonsynonymous SNV
ORF1a:cds-YP_009724389.1:exon1:c.C11651T:p.S3884L,ORF1a:cds-YP_009725295.1:exon1:
c.C11651T:p.S3884L 1.65 3.29452 1 0 D . . . .
. orf1ab polyprotein_VLLSVLQQLRVESSS 20 . . . . .
. . . . .
1 11962 11962 C T exonic ORF1a. synonymous SNV
ORF1a:cds-YP_009724389.1:exon1:c.C11697T:p.L3899L,ORF1a:cds-YP_009725295.1:exon1:
c.C11697T:p.L3899L -2.58 0.0666929 0.733354 1 T . . .
. . . . orf1ab_LAKDTTEAF 0.03 . . . . .
. . .
1 11995 11995 A G exonic ORF1a. synonymous SNV
ORF1a:cds-YP_009724389.1:exon1:c.A11730G:p.K3910K,ORF1a:cds-YP_009725295.1:exon1
:c.A11730G:p.K3910K -0.283 1.64627 1 1 T . . .
. . orf1ab polyprotein_EAFEKMVSLLSVLLS 18 orf1ab_TEAFEKMVSL
-1 . . . .
1 12015 12015 T G exonic ORF1a. nonsynonymous SNV
ORF1a:cds-YP_009724389.1:exon1:c.T11750G:p.V3917G,ORF1a:cds-YP_009725295.1:exon1
:c.T11750G:p.V3917G 1.65 2.19569 0.913732 0.01 D . .
. . . orf1ab polyprotein_EAFEKMVSLLSVLLS 18 . . .
. . . .
1 12020 12020 C T exonic ORF1a. nonsynonymous SNV
ORF1a:cds-YP_009724389.1:exon1:c.C11755T:p.L3919F,ORF1a:cds-YP_009725295.1:exon1:
c.C11755T:p.L3919F 0.773 1.92098 1 0.02 D . . . .
. orf1ab polyprotein_EAFEKMVSLLSVLLS 18 . . . . .
. . . . .
1 12022 12022 T C exonic ORF1a. synonymous SNV
ORF1a:cds-YP_009724389.1:exon1:c.T11757C:p.L3919L,ORF1a:cds-YP_009725295.1:exon1:
c.T11757C:p.L3919L -3.3 -0.757433 0.976472 1 T . . .
. . orf1ab polyprotein_EAFEKMVSLLSVLLS 18 orf1ab_SMQGAVDINK
0.1 . . . .
1 12034 12034 T C exonic ORF1a. synonymous SNV
ORF1a:cds-YP_009724389.1:exon1:c.T11769C:p.G3923G,ORF1a:cds-YP_009725295.1:exon
1:c.T11769C:p.G3923G -0.892 0.410079 1 0.66 T . . .
. . . . orf1ab_SMQGAVDINK 0.1 . . . . .
. . . .
1 12038 12038 G C exonic ORF1a. nonsynonymous SNV
ORF1a:cds-YP_009724389.1:exon1:c.G11773C:p.V3925L,ORF1a:cds-YP_009725295.1:exon1
:c.G11773C:p.V3925L 0.728 2.7451 1 0.03 D . . . .
. . . orf1ab_SMQGAVDINK 0.1 . . . . .
. . .
1 12046 12046 A G exonic ORF1a. nonsynonymous SNV
ORF1a:cds-YP_009724389.1:exon1:c.A11781G:p.I3927M,ORF1a:cds-YP_009725295.1:exon1:
c.A11781G:p.I3927M -0.819 0.410079 0.984315 0.01 D . . .

. . . . orf1ab_SMQGAVDINK 0.1 . . . .
. . . .
1 12053 12053 C T exonic ORF1a. nonsynonymous SNV
ORF1a:cds-YP_009724389.1:exon1:c.C11788T:p.L3930F,ORF1a:cds-YP_009725295.1:exon1:
c.C11788T:p.L3930F -2.33 -0.00198425 0.992157 0 D . . .
. . . .
.
1 12068 12068 C T exonic ORF1a. synonymous SNV
ORF1a:cds-YP_009724389.1:exon1:c.C11803T:p.L3935L,ORF1a:cds-YP_009725295.1:exon1:
c.C11803T:p.L3935L 1.65 3.29452 1 1 T . . . .
. . . orf1ab_EMLDNRATL 0.17 . . . . . .
. .
1 12075 12075 A G exonic ORF1a. nonsynonymous SNV
ORF1a:cds-YP_009724389.1:exon1:c.A11810G:p.N3937S,ORF1a:cds-YP_009725295.1:exon1
:c.A11810G:p.N3937S 1.65 2.26436 1 0.05 D . . .
. . . . orf1ab_EMLDNRATL 0.17 . . . . .
. . .
1 12116 12116 C T exonic ORF1a. nonsynonymous SNV
ORF1a:cds-YP_009724389.1:exon1:c.C11851T:p.L3951F,ORF1a:cds-YP_009725295.1:exon1:
c.C11851T:p.L3951F -0.891 0.547433 1 0 D . . . .
. orf1ab polyprotein_SEFSSLPSYAAFATA 14 orf1ab_ASEFSSLPSY -1
. . nCoV-2019_41_LEFT . . . . .

1 12243 12243 G T exonic ORF1a. nonsynonymous SNV
ORF1a:cds-YP_009724389.1:exon1:c.G11978T:p.R3993L,ORF1a:cds-YP_009725295.1:exon1
:c.G11978T:p.R3993L 1.65 2.7451 1 0.01 D . . . .
. . . orf1ab_KSEFDRDAAM -1 . .
nCoV-2019_40_RIGHT . . . . .
1 12295 12295 C T exonic ORF1a. synonymous SNV
ORF1a:cds-YP_009724389.1:exon1:c.C12030T:p.T4010T,ORF1a:cds-YP_009725295.1:exon1:
c.C12030T:p.T4010T 1.65 3.31557 1 1 T . . . .
. . . orf1ab_MADQAMTQMY -1 . . . . . .
. . .
1 12315 12315 G A exonic ORF1a. nonsynonymous SNV
ORF1a:cds-YP_009724389.1:exon1:c.G12050A:p.R4017K,ORF1a:cds-YP_009725295.1:exon1
:c.G12050A:p.R4017K 1.65 4.256 1 0 D . . . .
. . . .

1 12322 12322 G A exonic ORF1a. synonymous SNV
ORF1a:cds-YP_009724389.1:exon1:c.G12057A:p.E4019E,ORF1a:cds-YP_009725295.1:exon1
:c.G12057A:p.E4019E -2.06 -0.0430866 0.992142 1 T . .
. . . .
. .

1 12343 12343 T G exonic ORF1a. synonymous SNV
ORF1a:cds-YP_009724389.1:exon1:c.T12078G:p.T4026T,ORF1a:cds-YP_009725295.1:exon1
:c.T12078G:p.T4026T1.65 2.17363 1 0.29 T . . . .
. . . .

1 12374 12374 C T exonic ORF1a. nonsynonymous SNV
ORF1a:cds-YP_009724389.1:exon1:c.C12109T:p.L4037F,ORF1a:cds-YP_009725295.1:exon1:
c.C12109T:p.L4037F 1.65 3.31557 1 0.01 D . . . .
. . . orf1ab_QTMLFTMLR 0.09 . . . . . .
. .
1 12439 12439 C A exonic ORF1a. synonymous SNV
ORF1a:cds-YP_009724389.1:exon1:c.C12174A:p.P4058P,ORF1a:cds-YP_009725295.1:exon1
:c.C12174A:p.P4058P 1.65 0.964512 1 1 T . . .
. . orf1ab polyprotein_CVPLNIIPLTTAAKL 11 . . . .
nCoV-2019_42_LEFT. . . . .
1 12439 12439 C T exonic ORF1a. synonymous SNV
ORF1a:cds-YP_009724389.1:exon1:c.C12174T:p.P4058P,ORF1a:cds-YP_009725295.1:exon1
:c.C12174T:p.P4058P 1.65 0.964512 1 1 T . . .
. . orf1ab polyprotein_CVPLNIIPLTTAAKL 11 . . . .
nCoV-2019_42_LEFT. . . . .
1 12459 12459 C T exonic ORF1a. nonsynonymous SNV
ORF1a:cds-YP_009724389.1:exon1:c.C12194T:p.T4065I,ORF1a:cds-YP_009725295.1:exon1:
c.C12194T:p.T4065I 1.65 3.31557 1 0.11 T . . . .
. orf1ab polyprotein_CVPLNIIPLTTAAKL 11 orf1ab_IPLTTAAKL 0.07 .
. . . .
1 12473 12473 C A exonic ORF1a. nonsynonymous SNV
ORF1a:cds-YP_009724389.1:exon1:c.C12208A:p.L4070I,ORF1a:cds-YP_009725295.1:exon1:
c.C12208A:p.L4070I 0.776 1.90494 1 0 D . . . .
. orf1ab polyprotein_CVPLNIIPLTTAAKL 11 orf1ab_IPLTTAAKL 0.07 .
. nCoV-2019_41_RIGHT . . . . .

1 12484 12484 C T exonic ORF1a. synonymous SNV
ORF1a:cds-YP_009724389.1:exon1:c.C12219T:p.V4073V,ORF1a:cds-YP_009725295.1:exon1
:c.C12219T:p.V4073V -0.944 0.359953 1 1 T . . .
. . orf1ab polyprotein_IIPLTTAAKLMVVIP 18 orf1ab_VVIPDYNTYK-1
. . nCoV-2019_41_RIGHT . . . . .

1 12488 12488 C T exonic ORF1a. nonsynonymous SNV
ORF1a:cds-YP_009724389.1:exon1:c.C12223T:p.P4075S,ORF1a:cds-YP_009725295.1:exon1
:c.C12223T:p.P4075S 1.65 3.31557 1 0 D . . .
. . orf1ab polyprotein_IIPLTTAAKLMVVIP 18 orf1ab_VVIPDYNTYK-1
. . nCoV-2019_41_RIGHT . . . . .

1 12513 12513 C T exonic ORF1a. nonsynonymous SNV
ORF1a:cds-YP_009724389.1:exon1:c.C12248T:p.T4083M,ORF1a:cds-YP_009725295.1:exon1
:c.C12248T:p.T4083M 1.65 3.31557 1 0.02 D . . .
. . . . orf1ab_NTYKNTCDGTTFTY 0.04 . . . .
. . . .
1 12547 12547 A T exonic ORF1a. synonymous SNV
ORF1a:cds-YP_009724389.1:exon1:c.A12282T:p.A4094A,ORF1a:cds-YP_009725295.1:exon1
:c.A12282T:p.A4094A-3.3 -0.378953 0.992142 0.72 T . . .
. . . . orf1ab_TYASALWEI -1 . . . . .
. . .
1 12565 12565 G T exonic ORF1a. nonsynonymous SNV
ORF1a:cds-YP_009724389.1:exon1:c.G12300T:p.Q4100H,ORF1a:cds-YP_009725295.1:exon
1:c.G12300T:p.Q4100H -0.687 1.5019 1 0.03 D . . . .
. . . orf1ab_ALWEIQQVV 0.02 . . . . . .
. .
1 12578 12578 G T exonic ORF1a. nonsynonymous SNV
ORF1a:cds-YP_009724389.1:exon1:c.G12313T:p.D4105Y,ORF1a:cds-YP_009725295.1:exon1
:c.G12313T:p.D4105Y 1.65 4.256 1 0 D . . . .
. . . .

1 12631 12631 A G exonic ORF1a. synonymous SNV
ORF1a:cds-YP_009724389.1:exon1:c.A12366G:p.L4122L,ORF1a:cds-YP_009725295.1:exon1:
c.A12366G:p.L4122L -2.61 0.561472 1 1 T . . . .
. . . orf1ab_SEISMDNSPNL -1 . . . . .
. . .
1 12671 12671 G T exonic ORF1a. nonsynonymous SNV
ORF1a:cds-YP_009724389.1:exon1:c.G12406T:p.A4136S,ORF1a:cds-YP_009725295.1:exon1
:c.G12406T:p.A4136S 1.65 4.256 1 0.09 T . . . .
. orf1ab polyprotein_LIVTALRANSAVKLQ 11 orf1ab_ALRANSAVK 0.11 .
. . . .
1 12741 12741 C T exonic ORF1a. nonsynonymous SNV
ORF1a:cds-YP_009724389.1:exon1:c.C12476T:p.T4159I,ORF1a:cds-YP_009725295.1:exon1:
c.C12476T:p.T4159I 1.65 3.31557 1 0.01 D . . . .
. . . orf1ab_TTQTACTDDNALAY 0.01 . . . . . .
. . .
1 12786 12786 C T exonic ORF1a. nonsynonymous SNV
ORF1a:cds-YP_009724389.1:exon1:c.C12521T:p.T4174I,ORF1a:cds-YP_009725295.1:exon1:
c.C12521T:p.T4174I -0.778 1.2332 0.858551 0.06 T . . . .
. . . orf1ab_ALAYYNTTK -1 . . nCoV-2019_42_RIGHT
. . . . .
1 12793 12793 G T exonic ORF1a. nonsynonymous SNV
ORF1a:cds-YP_009724389.1:exon1:c.G12528T:p.K4176N,ORF1a:cds-YP_009725295.1:exon1
:c.G12528T:p.K4176N 1.65 4.256 1 0.02 D . . . .

.	.	.	orf1ab_ALAYYNTTK	-1	.	.	nCoV-2019_42_RIGHT
.	.	.	.	.
1	12815	12815	C	T	exonic	ORF1a.	synonymous SNV
ORF1a:cds-YP_009724389.1:exon1:c.C12550T:p.L4184L,ORF1a:cds-YP_009725295.1:exon1:c.C12550T:p.L4184L	0.484	0.29278	0.984283	0.67	T	.	.	.
.	.	.	.	orf1ab_ALLSDLQDL	0.15	.	.	.	.	.
.	.	.
1	12823	12823	C	T	exonic	ORF1a.	synonymous SNV
ORF1a:cds-YP_009724389.1:exon1:c.C12558T:p.S4186S,ORF1a:cds-YP_009725295.1:exon1:c.C12558T:p.S4186S	-3.3	-0.983512	0.968567	1	T	.	.
.	.	.	.	.	orf1ab_ALLSDLQDL	0.15	.	.	.	.
.	.	.	.
1	12835	12835	T	C	exonic	ORF1a.	synonymous SNV
ORF1a:cds-YP_009724389.1:exon1:c.T12570C:p.D4190D,ORF1a:cds-YP_009725295.1:exon1:c.T12570C:p.D4190D	-3.3	-0.580472	0.984283	0.96	T	.	.
.	.	.	.	.	orf1ab_ALLSDLQDL	0.15	.	.	.	.
.	.	.	.
1	12845	12845	G	T	exonic	ORF1a.	nonsynonymous SNV
ORF1a:cds-YP_009724389.1:exon1:c.G12580T:p.A4194S,ORF1a:cds-YP_009725295.1:exon1:c.G12580T:p.A4194S	0.745	2.71102	1	0	D	.	.	.
.	.	.	.	orf1ab_WARFPKSDGTGTIY	-1	.	.	.	.
.	.	.	.
1	12854	12854	C	T	exonic	ORF1a.	nonsynonymous SNV
ORF1a:cds-YP_009724389.1:exon1:c.C12589T:p.P4197S,ORF1a:cds-YP_009725295.1:exon1:c.C12589T:p.P4197S	1.65	3.31557	1	0.05	D	.	.	.
.	.	.	.	orf1ab_WARFPKSDGTGTIY	-1	.	.	.	.
.	.	.	.
1	12880	12880	C	T	exonic	ORF1a.	synonymous SNV
ORF1a:cds-YP_009724389.1:exon1:c.C12615T:p.I4205I,ORF1a:cds-YP_009725295.1:exon1:c.C12615T:p.I4205I	-3.3	-0.0430866	0.968567	1	T	.	.	.
.	.	.	.	orf1ab_WARFPKSDGTGTIY	-1	.	.	.	.
.	.	.	.
1	12883	12883	T	C	exonic	ORF1a.	synonymous SNV
ORF1a:cds-YP_009724389.1:exon1:c.T12618C:p.Y4206Y,ORF1a:cds-YP_009725295.1:exon1:c.T12618C:p.Y4206Y	-3.3	-2.05828	0.277039	1	T	.	.
.	.	.	.	.	orf1ab_WARFPKSDGTGTIY	-1	.	.	.
.	.	.	.	.
1	12885	12885	C	T	exonic	ORF1a.	nonsynonymous SNV
ORF1a:cds-YP_009724389.1:exon1:c.C12620T:p.T4207I,ORF1a:cds-YP_009725295.1:exon1:c.C12620T:p.T4207I	1.65	3.31557	0.992142	0.7	T	.	.	.
.	.	.	.	orf1ab_TELEPPCRF	-1	.	.	.	.	.
.	.	.
1	12886	12886	A	G	exonic	ORF1a.	synonymous SNV

ORF1a:cds-YP_009724389.1:exon1:c.A12621G:p.T4207T,ORF1a:cds-YP_009725295.1:exon1
:c.A12621G:p.T4207T 1.65 2.2408 1 0.22 T . . . .
. . . orf1ab_TELEPPCRF -1 . . . . . .
. .
1 12923 12923 C T exonic ORF1a. nonsynonymous SNV
ORF1a:cds-YP_009724389.1:exon1:c.C12658T:p.P4220S,ORF1a:cds-YP_009725295.1:exon1
:c.C12658T:p.P4220S 1.65 3.31557 1 0 D . . .
. . . . orf1ab_VTDTPKGPKVKYLY 0.02 . . . .
. . . .
1 12924 12924 C T exonic ORF1a. nonsynonymous SNV
ORF1a:cds-YP_009724389.1:exon1:c.C12659T:p.P4220L,ORF1a:cds-YP_009725295.1:exon1:
c.C12659T:p.P4220L 1.65 3.31557 1 0 D . . . .
. . . orf1ab_VTDTPKGPKVKYLY 0.02 . . . . .
. . .
1 12940 12940 G T exonic ORF1a. synonymous SNV
ORF1a:cds-YP_009724389.1:exon1:c.G12675T:p.V4225V,ORF1a:cds-YP_009725295.1:exon1
:c.G12675T:p.V4225V 1.65 4.256 1 0.91 T . . . .
. orf1ab polyprotein_KYLYFIKGLNNLNRG 17 orf1ab_VTDTPKGPKVKYLY 0.02
. . . .
1 12953 12953 T C exonic ORF1a. nonsynonymous SNV
ORF1a:cds-YP_009724389.1:exon1:c.T12688C:p.F4230L,ORF1a:cds-YP_009725295.1:exon1:
c.T12688C:p.F4230L 1.65 2.17363 0.992142 0 D . . .
. . orf1ab polyprotein_KYLYFIKGLNNLNRG 17 orf1ab_GPKVKYLYF 0.11
. . . .
1 12970 12970 C T exonic ORF1a. synonymous SNV
ORF1a:cds-YP_009724389.1:exon1:c.C12705T:p.N4235N,ORF1a:cds-YP_009725295.1:exon1
:c.C12705T:p.N4235N -3.3 0.0240866 0.984283 1 T . .
. . . orf1ab polyprotein_KYLYFIKGLNNLNRG 17 orf1ab_YFIKGLNNL
0.08 . . . .
1 12999 12999 G A exonic ORF1a. nonsynonymous SNV
ORF1a:cds-YP_009724389.1:exon1:c.G12734A:p.S4245N,ORF1a:cds-YP_009725295.1:exon1
:c.G12734A:p.S4245N 1.65 4.256 1 0.23 T . . . .
. orf1ab polyprotein_MVLGSLAATVRLQAG 18 . . . . .
. . . . .
1 13051 13051 C T exonic ORF1a. synonymous SNV
ORF1a:cds-YP_009724389.1:exon1:c.C12786T:p.A4262A,ORF1a:cds-YP_009725295.1:exon1
:c.C12786T:p.A4262A 1.65 3.31557 1 0.49 T . . .
. . . . orf1ab_TEVPANSTVL 0.07 . . . .
. . . .
1 13059 13059 C T exonic ORF1a. nonsynonymous SNV
ORF1a:cds-YP_009724389.1:exon1:c.C12794T:p.T4265I,ORF1a:cds-YP_009725295.1:exon1:
c.C12794T:p.T4265I 1.65 3.31557 1 0.01 D . . . .
. . . orf1ab_TEVPANSTVL 0.07 . . . . .

.	.	.
1	13114	13114	T	C	exonic	ORF1a.		synonymous SNV
ORF1a:cds-YP_009724389.1:exon1:c.T12849C:p.Y4283Y,ORF1a:cds-YP_009725295.1:exon1
:c.T12849C:p.Y4283Y	-2.23	0.225606	0.889984	1	T	.	.
.	.	.	orf1ab polyprotein_AFAVDAAKAYKDYLA	20	orf1ab_YLASGGQPI
0.12	.	.	.	.	.	.	.	.
1	13153	13153	G	A	exonic	ORF1a.		synonymous SNV
ORF1a:cds-YP_009724389.1:exon1:c.G12888A:p.K4296K,ORF1a:cds-YP_009725295.1:exon1
:c.G12888A:p.K4296K	1.65	4.256	1	1	T	.	.	.	.
.	.	.	.	.	.	.	.	.	.	.	.	.

1	13180	13180	T	C	exonic	ORF1a.		synonymous SNV
ORF1a:cds-YP_009724389.1:exon1:c.T12915C:p.G4305G,ORF1a:cds-YP_009725295.1:exon
1:c.T12915C:p.G4305G	-0.927	0.225606	1	1	T	.	.	.
.	.	.	.	.	.	.	.	.	.	.	.	.
.
1	13211	13211	A	G	exonic	ORF1a.		nonsynonymous SNV
ORF1a:cds-YP_009724389.1:exon1:c.A12946G:p.M4316V,ORF1a:cds-YP_009725295.1:exon
1:c.A12946G:p.M4316V	1.65	2.2408	0.992142	0.66	T	.	.	.
.	.	.	.	orf1ab_EANMDQESF	0.09	.	.	.	.	.
.	.	.
1	13327	13327	A	G	exonic	ORF1a.		synonymous SNV
ORF1a:cds-YP_009724389.1:exon1:c.A13062G:p.T4354T,ORF1a:cds-YP_009725295.1:exon1
:c.A13062G:p.T4354T	-1.17	0.255748	1	0.67	T	.	.	.
.	.	.	.	.	.	.	.	nCoV-2019_45_LEFT_alt2	.
.	.	.	.
1	13384	13384	C	T	exonic	ORF1a.		synonymous SNV
ORF1a:cds-YP_009724389.1:exon1:c.C13119T:p.C4373C,ORF1a:cds-YP_009725295.1:exon1
:c.C13119T:p.C4373C	-0.12	1.19331	1	1	T	.	.	.
.	.	.	.	.	.	.	.	nCoV-2019_44_RIGHT_alt0	.
.	.	.	.
1	13459	13459	G	T	exonic	ORF1a.		synonymous SNV
ORF1a:cds-YP_009724389.1:exon1:c.G13194T:p.S4398S,ORF1a:cds-YP_009725295.1:exon1
:c.G13194T:p.S4398S	0.731	2.6934	1	0.53	T	.	.	.	.
.	.	.	.	.	.	.	.	ORF1ab gene Reverse
GenScript; China CDC; PerkinElmer, Inc.	.	.	.
1	13517	13517	C	T	exonic	ORF1b.		nonsynonymous SNV
ORF1b:cds-YP_009724389.1:exon1:c.C50T:p.T17I	-1.07	0.693276	1	0.45	T
.	.	.	.	.	.	.	orf1ab_GTSTDVVYR	0.05	.	.
.	.	.	.	.	.
1	13604	13604	G	T	exonic	ORF1b.		nonsynonymous SNV
ORF1b:cds-YP_009724389.1:exon1:c.G137T:p.R46L	1.65	4.256	1	0	D
.	.	.	.	.	.	.	.	.	.	.

nCoV-2019_46_LEFT . . . . .
1 13680 13680 C T exonic ORF1b. synonymous SNV
ORF1b:cds-YP_009724389.1:exon1:c.C213T:p.Y71Y 0.68 1.63083 1 1
T . . . .
nCoV-2019_45_RIGHT_alt7 . . . . .
1 13695 13695 A G exonic ORF1b. synonymous SNV
ORF1b:cds-YP_009724389.1:exon1:c.A228G:p.T76T -0.87 0.380756 1 0.69
T . . . . orf1ab_HEETIYNLL 0.03 .
. nCoV-2019_45_RIGHT . . . . .

1 13712 13712 A G exonic ORF1b. nonsynonymous SNV
ORF1b:cds-YP_009724389.1:exon1:c.A245G:p.K82R 1.65 2.25587 1 0.07
T . . . . orf1ab_LLKDCPAVAK 0.13
. . . .
1 13721 13721 C T exonic ORF1b. nonsynonymous SNV
ORF1b:cds-YP_009724389.1:exon1:c.C254T:p.P85L 1.65 3.31844 1 0.05
D . . . . orf1ab_LLKDCPAVAK 0.13
. . . .
1 13730 13730 C T exonic ORF1b. nonsynonymous SNV
ORF1b:cds-YP_009724389.1:exon1:c.C263T:p.A88V 1.65 3.31844 1 0
D . . . . orf1ab_LLKDCPAVAK 0.13
. . . .
1 13860 13860 C T exonic ORF1b. synonymous SNV
ORF1b:cds-YP_009724389.1:exon1:c.C393T:p.D131D -3.3 -2.86945 0.323512
0.58 T . . . .
. . . . . .
1 13862 13862 C T exonic ORF1b. nonsynonymous SNV
ORF1b:cds-YP_009724389.1:exon1:c.C395T:p.T132I 0.761 1.88085 0.984268
0.24 T . . . .
. . . . . .
1 13884 13884 A G exonic ORF1b. synonymous SNV
ORF1b:cds-YP_009724389.1:exon1:c.A417G:p.T139T 0.46 0.943291 1 0.5
T . . . .
. . . . . .
1 13989 13989 C T exonic ORF1b. synonymous SNV
ORF1b:cds-YP_009724389.1:exon1:c.C522T:p.R174R -1.76 0.318252 1 1
T . . . . . orf1ab polyprotein_PDILRVYANLGERVR 15
. . . .

1 14120 14120 C T exonic ORF1b. nonsynonymous SNV
ORF1b:cds-YP_009724389.1:exon1:c.C653T:p.P218L -0.127 1.19331 1 0
D . . . .
SARS-CoV-2_IBS_RdRP3_R Korea . . .

1 14171 14171 T C exonic ORF1b. nonsynonymous SNV
ORF1b:cds-YP_009724389.1:exon1:c.T704C:p.I235T 1.65 2.19337 0.535898
0.02 D . . . . . orf1ab polyprotein_VDSYYSLLMPILTLT
20 orf1ab_SLLMPILTL 0.11 . . . ORF1ab Forward Singapore
. . .
1 14178 14178 C T exonic ORF1b. synonymous SNV
ORF1b:cds-YP_009724389.1:exon1:c.C711T:p.T237T -3.3 -1.49436 0.001 1
T . . . . . orf1ab polyprotein_VDSYYSLLMPILTLT 20
orf1ab_SLLMPILTL 0.11 . . . ORF1ab Forward Singapore .
. .
1 14183 14183 C T exonic ORF1b. nonsynonymous SNV
ORF1b:cds-YP_009724389.1:exon1:c.C716T:p.T239I 1.65 3.31844 0.944937
0.05 D . . . . . orf1ab polyprotein_VDSYYSLLMPILTLT
20 orf1ab_MPILTLTRAL 0.1 . . . .

1 14184 14184 C T exonic ORF1b. synonymous SNV
ORF1b:cds-YP_009724389.1:exon1:c.C717T:p.T239T -3.3 -0.556803 0.921339
1 T . . . . . orf1ab polyprotein_VDSYYSLLMPILTLT
20 orf1ab_MPILTLTRAL 0.1 . . . .

1 14268 14268 G A exonic ORF1b. synonymous SNV
ORF1b:cds-YP_009724389.1:exon1:c.G801A:p.T267T 1.65 4.256 1 1 T
. . . .
. . . .
1 14277 14277 G T exonic ORF1b. nonsynonymous SNV
ORF1b:cds-YP_009724389.1:exon1:c.G810T:p.R270S 0.52 1.0683 0.992134 0
D . . . .
nCoV-2019_47_RIGHT . . . . .
1 14318 14318 C T exonic ORF1b. nonsynonymous SNV
ORF1b:cds-YP_009724389.1:exon1:c.C851T:p.T284I -1.11 0.380756 1 0.04
D . . . .
. . . . .
1 14331 14331 T C exonic ORF1b. synonymous SNV
ORF1b:cds-YP_009724389.1:exon1:c.T864C:p.N288N 0.487 1.0058 1 1 T
. . . .
. . . .
1 14408 14408 C T exonic ORF1b. nonsynonymous SNV
ORF1b:cds-YP_009724389.1:exon1:c.C941T:p.P314L 1.65 3.30748 1 0.31
T . . . . orf1ab_TVFPPTSFGPLVRK -1
. . . .
1 14512 14512 G A exonic ORF1b. nonsynonymous SNV
ORF1b:cds-YP_009724389.1:exon1:c.G1045A:p.D349N 1.65 4.256 1 0 D
. . . .

. . . .
1 14599 14599 C T exonic ORF1b. synonymous SNV
ORF1b:cds-YP_009724389.1:exon1:c.C1132T:p.L378L 0.431 0.294535 0.992646
0.21 T . . . .
nCoV-2019_48_RIGHT . . . . .
1 14621 14621 C T exonic ORF1b. nonsynonymous SNV
ORF1b:cds-YP_009724389.1:exon1:c.C1154T:p.T385M 1.65 3.30748 1 0.05
D . . . .
. . . . .
1 14645 14645 C T exonic ORF1b. nonsynonymous SNV
ORF1b:cds-YP_009724389.1:exon1:c.C1178T:p.T393I 1.65 3.30748 1 0.01
D . . . .
. . . . .
1 14743 14743 G A exonic ORF1b. nonsynonymous SNV
ORF1b:cds-YP_009724389.1:exon1:c.G1276A:p.V426I 1.65 4.256 1 0.32 T
. . . .
. . . . .
1 14767 14767 G T exonic ORF1b. nonsynonymous SNV
ORF1b:cds-YP_009724389.1:exon1:c.G1300T:p.A434S 1.65 4.256 1 0.11 T
. . . .
. . . . .
1 14786 14786 C T exonic ORF1b. nonsynonymous SNV
ORF1b:cds-YP_009724389.1:exon1:c.C1319T:p.A440V 1.65 3.30748 1 0
D . . . . orf1ab_NAAISDYDY 0.07 .
. . . . . . hypermutable low-fitness site

1 14790 14790 C T exonic ORF1b. synonymous SNV
ORF1b:cds-YP_009724389.1:exon1:c.C1323T:p.I441I -1.36 0.517717 1 1
T . . . . orf1ab_NAAISDYDY 0.07 .
. . . .
1 14829 14829 G T exonic ORF1b. nonsynonymous SNV
ORF1b:cds-YP_009724389.1:exon1:c.G1362T:p.M454I 1.65 4.256 1 0.09 T
. . . .
. . . . .
1 14837 14837 T C exonic ORF1b. nonsynonymous SNV
ORF1b:cds-YP_009724389.1:exon1:c.T1370C:p.I457T 1.65 2.19157 1 0
D . . . .
. . . . .
1 14857 14857 G T exonic ORF1b. nonsynonymous SNV
ORF1b:cds-YP_009724389.1:exon1:c.G1390T:p.V464F 0.741 2.74953 1 0.06
T . . . . orf1ab_FVVEVVDKY 0.06 .
. . . .
1 14874 14874 G T exonic ORF1b. nonsynonymous SNV

ORF1b:cds-YP_009724389.1:exon1:c.G1407T:p.K469N -3.3 0.0713543 0.992646
0.01 D . . . . orf1ab_FVVEVVDKY 0.06
. . nCoV-2019_50_LEFT . . . . .

1 14877 14877 C T exonic ORF1b. synonymous SNV
ORF1b:cds-YP_009724389.1:exon1:c.C1410T:p.Y470Y -2.3 0.23874 1 1
T . . . . orf1ab_FVVEVVDKY 0.06 .
. nCoV-2019_50_LEFT . . . . .
1 15138 15138 G T exonic ORF1b. nonsynonymous SNV
ORF1b:cds-YP_009724389.1:exon1:c.G1671T:p.M557I 1.65 4.256 1 0 D
. . . . orf1ab_MTNRQFHQKLLK -1 .
. . . .
1 15147 15147 A G exonic ORF1b. synonymous SNV
ORF1b:cds-YP_009724389.1:exon1:c.A1680G:p.R560R 1.65 2.24737 1 1
T . . . . . orf1ab polyprotein_RQFHQKLLKSIAATR 16
orf1ab_MTNRQFHQKLLK -1 . . . .

1 15194 15194 C T exonic ORF1b. nonsynonymous SNV
ORF1b:cds-YP_009724389.1:exon1:c.C1727T:p.A576V 1.65 3.30748 1 0.49
T . . . . . orf1ab polyprotein_KLLKSIAATRGATVV 8.6
orf1ab_IAATRGATV 0.17 . . . .

1 15202 15202 G T exonic ORF1b. nonsynonymous SNV
ORF1b:cds-YP_009724389.1:exon1:c.G1735T:p.V579L 1.65 4.256 1 0 D
. . . . . orf1ab polyprotein_KLLKSIAATRGATVV 8.6
orf1ab_IAATRGATV 0.17 . . . .

1 15214 15214 A G exonic ORF1b. nonsynonymous SNV
ORF1b:cds-YP_009724389.1:exon1:c.A1747G:p.S583G 1.65 2.24737 1 0
D . . . . orf1ab_ATVVIGTSK -1 .
. . . .
1 15237 15237 C T exonic ORF1b. synonymous SNV
ORF1b:cds-YP_009724389.1:exon1:c.C1770T:p.H590H 0.477 0.294535 0.992646
0.16 T . . . . orf1ab_FYGGWHNML
-1 . . nCoV-2019_50_RIGHT Australia_RDRP1 Australia .
. .
1 15267 15267 A T exonic ORF1b. synonymous SNV
ORF1b:cds-YP_009724389.1:exon1:c.A1800T:p.V600V -0.867 0.350331 1 1
T . . . . orf1ab_TVYSDVENPHLMGW
0.14 . . . .
1 15277 15277 C T exonic ORF1b. nonsynonymous SNV
ORF1b:cds-YP_009724389.1:exon1:c.C1810T:p.H604Y -0.305 0.908283 1 0.14
T . RdRp_catalytic . . . . .

orf1ab_TVYSDVENPHLMGW 0.14 . . . .
.
1 15324 15324 C T exonic ORF1b. synonymous SNV
ORF1b:cds-YP_009724389.1:exon1:c.C1857T:p.N619N -3.3 0.0713543 0.992646
1 T . RdRp_catalytic . . . orf1ab
polyprotein_RAMPNMLRIMASLVL 3.6 . . . .
. homoplasic position .
1 15348 15348 T C exonic ORF1b. synonymous SNV
ORF1b:cds-YP_009724389.1:exon1:c.T1881C:p.L627L -2.69 -0.0960315 0.992646
1 T . RdRp_catalytic . . . orf1ab
polyprotein_RAMPNMLRIMASLVL 3.6 . . . .
. . .
1 15396 15396 C T exonic ORF1b. synonymous SNV
ORF1b:cds-YP_009724389.1:exon1:c.C1929T:p.F643F 0.782 1.96783 1 1
T . RdRp_catalytic . . . . . orf1ab_SLSHRFYRL
0.06 . . . .
1 15418 15418 G T exonic ORF1b. nonsynonymous SNV
ORF1b:cds-YP_009724389.1:exon1:c.G1951T:p.A651S 1.65 4.256 1 0 D
. RdRp_catalytic . . . . . orf1ab_RLANECAQV 0.16
. . . .
1 15444 15444 G T exonic ORF1b. nonsynonymous SNV
ORF1b:cds-YP_009724389.1:exon1:c.G1977T:p.M659I 1.65 4.256 1 0.21 T
. RdRp_catalytic . . . . . orf1ab_LSEMVMCGGSLY
-1 . . . RdRP gene Forward | CDCV_GERMANY_RdRP_1 |
SARS-CoV-2_IBS_RdRP1_F GenScript, National Institute of Virology, Pune, India | Christian
Drosten Charite Virology, Berlin, Germany | Korea . . .

1 15451 15451 G A exonic ORF1b. nonsynonymous SNV
ORF1b:cds-YP_009724389.1:exon1:c.G1984A:p.G662S 1.65 4.256 1 0 D
. RdRp_catalytic . . . . . orf1ab_LSEMVMCGGSLY
-1 . . . RdRP gene Forward | CDCV_GERMANY_RdRP_1 |
SARS-CoV-2_IBS_RdRP1_F GenScript, National Institute of Virology, Pune, India | Christian
Drosten Charite Virology, Berlin, Germany | Korea . . .

1 15535 15535 G T exonic ORF1b. nonsynonymous SNV
ORF1b:cds-YP_009724389.1:exon1:c.G2068T:p.A690S 1.65 4.256 1 0 D
. RdRp_catalytic . . . .
. . . . . .
1 15543 15543 G A exonic ORF1b. synonymous SNV
ORF1b:cds-YP_009724389.1:exon1:c.G2076A:p.T692T -0.837 0.442378 1 0.55
T . RdRp_catalytic . . . .
. nCoV-2019_51_RIGHT SARS-CoV-2_IBS_RdRP1_R Korea . .
.

1 15553 15553 A G exonic ORF1b. nonsynonymous SNV
ORF1b:cds-YP_009724389.1:exon1:c.A2086G:p.N696D 1.65 2.24518 1 0.28
T . RdRp_catalytic . . . orf1ab
polyprotein_VNALLSTDGNKIADK 17 . . . .
nCoV-2019_51_RIGHT SARS-CoV-2_IBS_RdRP1_R Korea . . .

1 15615 15615 C T exonic ORF1b. synonymous SNV
ORF1b:cds-YP_009724389.1:exon1:c.C2148T:p.H716H -0.218 1.13576 1 0.26
T . RdRp_catalytic . . . orf1ab
polyprotein_KIADKYVRNLQHRLY 20 . . . .
. . .
1 15654 15654 C T exonic ORF1b. synonymous SNV
ORF1b:cds-YP_009724389.1:exon1:c.C2187T:p.D729D 0.475 0.997087 1 1
T . RdRp_catalytic . . . . .
orf1ab_NRDVDTDFVNEFY 0.06 . . . .

1 15726 15726 T A exonic ORF1b. synonymous SNV
ORF1b:cds-YP_009724389.1:exon1:c.T2259A:p.A753A -1.4 0.789071 1 0.18
T . RdRp_catalytic . . . orf1ab
polyprotein_LRKHFSMMILSDDAV 18 orf1ab_LSDDAVVCFNSTY 0.01 . .
. . . . . .
1 15755 15755 C A exonic ORF1b. nonsynonymous SNV
ORF1b:cds-YP_009724389.1:exon1:c.C2288A:p.S763Y 1.65 3.28526 1 0.01
D . RdRp_catalytic . . . .
. . . .
1 15768 15768 G T exonic ORF1b. synonymous SNV
ORF1b:cds-YP_009724389.1:exon1:c.G2301T:p.V767V -2.13 0.303701 1 1
T . . . . . orf1ab polyprotein_GLVASIKNFKSVLYY 8
orf1ab_LVASIKNFK -1 . . . .

1 15798 15798 T C exonic ORF1b. synonymous SNV
ORF1b:cds-YP_009724389.1:exon1:c.T2331C:p.L777L 1.65 2.17584 1 1
T . . . . . orf1ab polyprotein_GLVASIKNFKSVLYY 8
orf1ab_SIKNFKSVL 0.07 . . . .

1 15810 15810 C T exonic ORF1b. synonymous SNV
ORF1b:cds-YP_009724389.1:exon1:c.C2343T:p.N781N -3.3 -0.181669 0.992189
1 T . . . .
. . . . . .
1 15828 15828 A C exonic ORF1b. nonsynonymous SNV
ORF1b:cds-YP_009724389.1:exon1:c.A2361C:p.E787D -0.283 1.62113 1 0.27
T . . . .
nCoV-2019_53_LEFT . . . . .

1	15848	15848	C	T	exonic	ORF1b.	nonsynonymous SNV
ORF1b:cds-YP_009724389.1:exon1:c.C2381T:p.T794I	1.65	3.28526	1	0.07
T	.	.	.	.	.	.	.	.	.	.
nCoV-2019_53_LEFT	.	.	.	.	.
1	15853	15853	C	A	exonic	ORF1b.	nonsynonymous SNV
ORF1b:cds-YP_009724389.1:exon1:c.C2386A:p.L796I	1.65	3.28526	1	0.81
T	.	.	.	.	.	.	.	.	.	.	.
.	.	.	.	.
1	15946	15946	A	C	exonic	ORF1b.	synonymous SNV
ORF1b:cds-YP_009724389.1:exon1:c.A2479C:p.R827R	0.485	0.997087	0.945323
1	T	.	.	.	.	.	.	.	orf1ab_YLPYPDPSRIL
-1	.	.	.	.	.	.	.	.
1	15957	15957	G	T	exonic	ORF1b.	synonymous SNV
ORF1b:cds-YP_009724389.1:exon1:c.G2490T:p.G830G	-1.17	0.650394	1	1
T	.	.	.	.	.	.	.	.	.	.	.
.	.	.	.	.
1	15958	15958	G	T	exonic	ORF1b.	nonsynonymous SNV
ORF1b:cds-YP_009724389.1:exon1:c.G2491T:p.A831S	1.65	4.256	1	0	D
.	.	.	.	.	.	.	.	.	.	.	.	.
.	.	.	.
1	15960	15960	C	T	exonic	ORF1b.	synonymous SNV
ORF1b:cds-YP_009724389.1:exon1:c.C2493T:p.A831A	-3.3	-0.944394	0.968756
1	T	.	.	.	.	.	.	.	.	.	.
.	.	.	.	.	.
1	15982	15982	G	T	exonic	ORF1b.	nonsynonymous SNV
ORF1b:cds-YP_009724389.1:exon1:c.G2515T:p.V839L	1.65	4.256	1	0.66	T
.	.	.	.	.	orf1ab polyprotein_VDDIVKTDGTLMIER	15	.
.	.	.	.	.	.	.	.	.
1	16035	16035	T	C	exonic	ORF1b.	synonymous SNV
ORF1b:cds-YP_009724389.1:exon1:c.T2568C:p.D856D	-3.3	-1.42976	0.812535
1	T	.	.	.	.	.	orf1ab polyprotein_LMIERFVSLAIDAYP
9.6	orf1ab_FVSLAIDAY	0.08	.	.	.	.	.	.	.	.

1	16049	16049	C	T	exonic	ORF1b.	nonsynonymous SNV
ORF1b:cds-YP_009724389.1:exon1:c.C2582T:p.T861I	1.65	3.28526	1	0.06
T	.	.	.	.	.	.	.	orf1ab_AIDAYPLTK	-1	.
.	.	.	.	.	.	.
1	16065	16065	G	T	exonic	ORF1b.	nonsynonymous SNV
ORF1b:cds-YP_009724389.1:exon1:c.G2598T:p.Q866H	-0.431	1.82915	1	0.04
D	.	.	.	.	.	.	.	orf1ab_YPLTKHPNQEY	0.07
.	.	.	.	.	.	.	.
1	16075	16075	G	T	exonic	ORF1b.	nonsynonymous SNV
ORF1b:cds-YP_009724389.1:exon1:c.G2608T:p.D870Y	1.65	4.256	1	0.02	D

. . . . orf1ab_HPNQEYADVFHLY -1 .
. . . .
1 16082 16082 T C exonic ORF1b. nonsynonymous SNV
ORF1b:cds-YP_009724389.1:exon1:c.T2615C:p.F872S 1.65 2.17584 1 0
D . . . . orf1ab_HPNQEYADVFHLY -1
. . . .
1 16092 16092 C T exonic ORF1b. synonymous SNV
ORF1b:cds-YP_009724389.1:exon1:c.C2625T:p.Y875Y -3.3 -0.112331 0.992189
1 T . . . . orf1ab_HPNQEYADVFHLY
-1 . . . .
1 16111 16111 C T exonic ORF1b. synonymous SNV
ORF1b:cds-YP_009724389.1:exon1:c.C2644T:p.L882L 0.453 0.303701 1 1
T . . . . orf1ab_HLYLQYIRKL -1 .
. . . .
1 16146 16146 G A exonic ORF1b. nonsynonymous SNV
ORF1b:cds-YP_009724389.1:exon1:c.G2679A:p.M893I 1.65 4.256 1 0.02 D
. . . . orf1ab_MLDMYSVML 0.08 . .
. . . .
1 16208 16208 C T exonic ORF1b. nonsynonymous SNV
ORF1b:cds-YP_009724389.1:exon1:c.C2741T:p.A914V 1.65 3.28526 1 0.01
D . . . . orf1ab_YEAMYTPHTVL 0.09
. . nCoV-2019_53_RIGHT . . . . .

1 16260 16260 C T exonic ORF1b. synonymous SNV
ORF1b:cds-YP_009724389.1:exon1:c.C2793T:p.C931C -3.3 -0.736378 0.960945
1 T . CV_ZBD . . . .
. . . .
1 16289 16289 C T exonic ORF1b. nonsynonymous SNV
ORF1b:cds-YP_009724389.1:exon1:c.C2822T:p.A941V 1.65 3.28526 0.984378
0.05 D . CV_ZBD . . . .
. . . .
1 16308 16308 C T exonic ORF1b. synonymous SNV
ORF1b:cds-YP_009724389.1:exon1:c.C2841T:p.F947F 1.65 3.28526 1 0.74
T . CV_ZBD . . . .
. . . . . .
1 16323 16323 C T exonic ORF1b. synonymous SNV
ORF1b:cds-YP_009724389.1:exon1:c.C2856T:p.C952C 1.65 3.28526 1 1
T . CV_ZBD . . . .
. . . . . .
1 16368 16368 T C exonic ORF1b. synonymous SNV
ORF1b:cds-YP_009724389.1:exon1:c.T2901C:p.S967S 0.431 0.927748 1 1
T . CV_ZBD . . . orf1ab polyprotein_TSHKLVLSVNPYVCN
9.5 . . . .

1 16377 16377 G T exonic ORF1b. synonymous SNV
ORF1b:cds-YP_009724389.1:exon1:c.G2910T:p.P970P 0.753 1.82915 1 1
T . CV_ZBD . . . orf1ab polyprotein_TSHKLVLSVNPYVCN
9.5 . . . .

1 16396 16396 G T exonic ORF1b. nonsynonymous SNV
ORF1b:cds-YP_009724389.1:exon1:c.G2929T:p.G977C 1.65 4.256 1 0 D
. CV_ZBD . . . .
. . . . .
1 16508 16508 G T exonic ORF1b. nonsynonymous SNV
ORF1b:cds-YP_009724389.1:exon1:c.G3041T:p.G1014V 1.65 4.256 1 0 D
. . . .
nCoV-2019_54_RIGHT . . . . .
1 16528 16528 G A exonic ORF1b. nonsynonymous SNV
ORF1b:cds-YP_009724389.1:exon1:c.G3061A:p.V1021I 1.65 4.256 1 0.07 T
. . . .
. . . .
1 16575 16575 C T exonic ORF1b. synonymous SNV
ORF1b:cds-YP_009724389.1:exon1:c.C3108T:p.D1036D -3.3 -1.18765 0.889984
1 T . . . .
. . . . . .
1 16616 16616 C T exonic ORF1b. nonsynonymous SNV
ORF1b:cds-YP_009724389.1:exon1:c.C3149T:p.T1050I 1.65 3.34872 1 0.17
T . . . . . orf1ab polyprotein_TERLKLFAAETLKAT 5.1
. . . .

1 16626 16626 C T exonic ORF1b. synonymous SNV
ORF1b:cds-YP_009724389.1:exon1:c.C3159T:p.L1053L -1.24 0.551291 1 1
T . . . . . orf1ab polyprotein_TERLKLFAAETLKAT 5.1
. . . .

1 16641 16641 A G exonic ORF1b. synonymous SNV
ORF1b:cds-YP_009724389.1:exon1:c.A3174G:p.A1058A -3.3 -0.658409 0.976425
1 T . . . . . orf1ab polyprotein_TERLKLFAAETLKAT
5.1 orf1ab_KLFAAETLK -1 . . . .

1 16699 16699 C T exonic ORF1b. nonsynonymous SNV
ORF1b:cds-YP_009724389.1:exon1:c.C3232T:p.R1078C 1.65 3.34872 1 0
D . . . . . orf1ab polyprotein_EETFKLSYGIATVRE 12
orf1ab_KLSYGIATV 0.04 . . . .

1 16707 16707 G A exonic ORF1b. synonymous SNV

ORF1b:cds-YP_009724389.1:exon1:c.G3240A:p.V1080V -2.02 0.77811 1 1
T . . . . orf1ab_REVLSDRELHLSW -1
. . . .
1 16726 16726 C T exonic ORF1b. nonsynonymous SNV
ORF1b:cds-YP_009724389.1:exon1:c.C3259T:p.H1087Y -1.25 0.626898 0.976425
0.11 T . . . . orf1ab_REVLSDRELHLSW
-1 . . . .
1 16733 16733 C T exonic ORF1b. nonsynonymous SNV
ORF1b:cds-YP_009724389.1:exon1:c.C3266T:p.S1089L 1.65 3.34872 1 0.41
T . . . . orf1ab_REVLSDRELHLSW -1
. . . .
1 16738 16738 G C exonic ORF1b. nonsynonymous SNV
ORF1b:cds-YP_009724389.1:exon1:c.G3271C:p.E1091Q 1.65 4.256 1 0 D
. . . . orf1ab_REVLSDRELHLSW -1 .
. . . .
1 16762 16762 C T exonic ORF1b. nonsynonymous SNV
ORF1b:cds-YP_009724389.1:exon1:c.C3295T:p.L1099F -3.3 0.0220472 0.992142
0 D . . . . orf1ab_VGKPRPPL 0.1
. . nCoV-2019_56_LEFT . . . . .

1 16887 16887 C T exonic ORF1b. synonymous SNV
ORF1b:cds-YP_009724389.1:exon1:c.C3420T:p.Y1140Y -3.3 -0.204772 0.984283
1 T . . . . . orf1ab polyprotein_DAVVYRGTTTYKLNV
9 orf1ab_VVYRGTTTYK -1 . . . .
.
1 16915 16915 C T exonic ORF1b. synonymous SNV
ORF1b:cds-YP_009724389.1:exon1:c.C3448T:p.L1150L -0.737 0.400079 1 1
T . . . . . orf1ab polyprotein_GDYFVLTSHTVMPLS 15
orf1ab_TYKLNVGDYFV -1 . . . .

1 16917 16917 G T exonic ORF1b. synonymous SNV
ORF1b:cds-YP_009724389.1:exon1:c.G3450T:p.L1150L -3.3 -0.0535591 0.992142
1 T . . . . . orf1ab polyprotein_GDYFVLTSHTVMPLS
15 . . . .

1 16952 16952 C T exonic ORF1b. nonsynonymous SNV
ORF1b:cds-YP_009724389.1:exon1:c.C3485T:p.T1162I 1.65 3.34872 1 0.11
T . . . . orf1ab_MPLSAPTL 0.14 .
. . . .
1 16975 16975 G T exonic ORF1b. nonsynonymous SNV
ORF1b:cds-YP_009724389.1:exon1:c.G3508T:p.V1170F 1.65 4.256 1 0.07 T
. . . . orf1ab_TLVPQEHYV 0.04 . .
. . . . . .

1 17010 17010 C T exonic ORF1b. synonymous SNV
ORF1b:cds-YP_009724389.1:exon1:c.C3543T:p.I1181I -3.3 -0.582803 0.944992
0.62 T . #ERROR! . . . . . orf1ab_YPTLNISDEF
0.06 . . . .
1 17014 17014 G T exonic ORF1b. nonsynonymous SNV
ORF1b:cds-YP_009724389.1:exon1:c.G3547T:p.D1183Y 1.65 2.74387 0.984283
0 D . #ERROR! . . . . . orf1ab_YPTLNISDEF
0.06 . . . .
1 17019 17019 G T exonic ORF1b. nonsynonymous SNV
ORF1b:cds-YP_009724389.1:exon1:c.G3552T:p.E1184D -0.251 1.76099 1 0.57
T . #ERROR! . . . . . orf1ab_YPTLNISDEF 0.06
. . . .
1 17036 17036 C T exonic ORF1b. nonsynonymous SNV
ORF1b:cds-YP_009724389.1:exon1:c.C3569T:p.A1190V 0.783 1.9122 1 0.76 T
. #ERROR! . . . . . orf1ab_ISDEFSSNVANY -1
. . . .
1 17067 17067 T C exonic ORF1b. synonymous SNV
ORF1b:cds-YP_009724389.1:exon1:c.T3600C:p.Y1200Y -1.52 0.77811 1 1
T . #ERROR! . . . . . orf1ab_VGMQKYSTL 0.02
. . nCoV-2019_57_LEFT . . . . .

1 17122 17122 G T exonic ORF1b. nonsynonymous SNV
ORF1b:cds-YP_009724389.1:exon1:c.G3655T:p.A1219S 1.65 4.256 1 0.02 D
. #ERROR! . . . orf1ab polyprotein_GKSHFAIGLALYYPS 16
orf1ab_FAIGLALYY 0.04 . . . .

1 17135 17135 C T exonic ORF1b. nonsynonymous SNV
ORF1b:cds-YP_009724389.1:exon1:c.C3668T:p.P1223L 1.65 3.34872 1 0.13
T . #ERROR! . . . orf1ab polyprotein_GKSHFAIGLALYYPS
16 orf1ab_YPSARIVY 0.04 . . nCoV-2019_56_RIGHT . .
. . .
1 17241 17241 T A exonic ORF1b. synonymous SNV
ORF1b:cds-YP_009724389.1:exon1:c.T3774A:p.P1258P 0.484 1.00493 1 1
T . #ERROR! . . . . . orf1ab_IPARARVECF0.12
. . . .
1 17278 17278 G T exonic ORF1b. nonsynonymous SNV
ORF1b:cds-YP_009724389.1:exon1:c.G3811T:p.V1271L 1.65 4.256 1 0.21 T
. #ERROR! . . . .
. . . . .
1 17280 17280 G T exonic ORF1b. synonymous SNV
ORF1b:cds-YP_009724389.1:exon1:c.G3813T:p.V1271V -3.3 -0.129165 0.984283
1 T . #ERROR! . . . .
. . . .

1 17288 17288 C T exonic ORF1b. nonsynonymous SNV
ORF1b:cds-YP_009724389.1:exon1:c.C3821T:p.T1274I 0.786 1.98781 1 0
D . #ERROR! . . . .
. . . . . .
1 17304 17304 C T exonic ORF1b. synonymous SNV
ORF1b:cds-YP_009724389.1:exon1:c.C3837T:p.V1279V 0.475 1.00493 1 1
T . #ERROR! . . . orf1ab polyprotein_LEQYVFCTVNALPET
14 . . . .

1 17331 17331 G T exonic ORF1b. nonsynonymous SNV
ORF1b:cds-YP_009724389.1:exon1:c.G3864T:p.E1288D 0.52 1.08054 1 0
D . #ERROR! . . . orf1ab polyprotein_LEQYVFCTVNALPET
14 . . . .

1 17334 17334 G T exonic ORF1b. synonymous SNV
ORF1b:cds-YP_009724389.1:exon1:c.G3867T:p.T1289T -3.3 -0.204772 0.992142
0.34 T . #ERROR! . . . orf1ab
polyprotein_LEQYVFCTVNALPET 14 . . . .
. . .
1 17336 17336 C T exonic ORF1b. nonsynonymous SNV
ORF1b:cds-YP_009724389.1:exon1:c.C3869T:p.T1290I 1.65 3.34872 1 0.07
T . #ERROR! . . . .
. . . . . .
1 17339 17339 C T exonic ORF1b. nonsynonymous SNV
ORF1b:cds-YP_009724389.1:exon1:c.C3872T:p.A1291V 1.65 3.34872 1 0.39
T . #ERROR! . . . .
. . . . . .
1 17358 17358 T C exonic ORF1b. synonymous SNV
ORF1b:cds-YP_009724389.1:exon1:c.T3891C:p.D1297D 0.431 0.929323 1 1
T . #ERROR! . . . . . orf1ab_DEISMATNY -1
. . . .
1 17410 17410 C T exonic ORF1b. nonsynonymous SNV
ORF1b:cds-YP_009724389.1:exon1:c.C3943T:p.R1315C 1.65 3.32576 1 0.01
D . #ERROR! . . . orf1ab polyprotein_YDLSVVNARLRAKHY
20 orf1ab_VVNARLRAK 0.08 . . . .

1 17474 17474 C T exonic ORF1b. nonsynonymous SNV
ORF1b:cds-YP_009724389.1:exon1:c.C4007T:p.T1336I 1.65 3.32576 0.984457
0.08 T . #ERROR! . . . . .
orf1ab_APRTLLTKGTL -1 . . . .

1 17487 17487 A T exonic ORF1b. synonymous SNV
ORF1b:cds-YP_009724389.1:exon1:c.A4020T:p.L1340L -3.3 -0.00758268 1 1

T . #ERROR! . . . . . orf1ab_APRTLLTKGTL
-1 . . . .
1 17491 17491 C T exonic ORF1b. nonsynonymous SNV
ORF1b:cds-YP_009724389.1:exon1:c.C4024T:p.P1342S 1.65 3.32576 1 0
D . #ERROR! . . . .
. . . . . .
1 17518 17518 C T exonic ORF1b. nonsynonymous SNV
ORF1b:cds-YP_009724389.1:exon1:c.C4051T:p.L1351F 1.65 3.32576 1 0.09
T . #ERROR! . . . .
. . . . . .
1 17550 17550 C T exonic ORF1b. synonymous SNV
ORF1b:cds-YP_009724389.1:exon1:c.C4083T:p.L1361L -0.809 -0.00758268 0.992228
1 T . #ERROR! . . . .
. . . .
1 17562 17562 G T exonic ORF1b. synonymous SNV
ORF1b:cds-YP_009724389.1:exon1:c.G4095T:p.R1365R -3.21 -0.00758268 0.992228
0.19 T . #ERROR! . . . .
. . . .
1 17566 17566 T A exonic ORF1b. nonsynonymous SNV
ORF1b:cds-YP_009724389.1:exon1:c.T4099A:p.C1367S 1.65 2.16297 0.984457
0 D . #ERROR! . . . .
. . . .
1 17649 17649 C T exonic ORF1b. synonymous SNV
ORF1b:cds-YP_009724389.1:exon1:c.C4182T:p.C1394C -2.42 0.147457 1 1
T . #ERROR! . . . .
. . . . . .
1 17675 17675 T C exonic ORF1b. nonsynonymous SNV
ORF1b:cds-YP_009724389.1:exon1:c.T4208C:p.I1403T 1.65 2.16297 1 0.53
T . #ERROR! . . . orf1ab polyprotein_FKMFYKGVITHDVSS
17 . . . . nCoV-2019_59_LEFT . . . . .

1 17676 17676 C T exonic ORF1b. synonymous SNV
ORF1b:cds-YP_009724389.1:exon1:c.C4209T:p.I1403I 0.397 0.224976 1 0.3
T . #ERROR! . . . orf1ab polyprotein_FKMFYKGVITHDVSS
17 . . . . nCoV-2019_59_LEFT . . . . .

1 17678 17678 C T exonic ORF1b. nonsynonymous SNV
ORF1b:cds-YP_009724389.1:exon1:c.C4211T:p.T1404M 1.65 3.32576 1 0.02
D . #ERROR! . . . orf1ab polyprotein_FKMFYKGVITHDVSS
17 . . . . nCoV-2019_59_LEFT . . . . .

1 17731 17731 T C exonic ORF1b. nonsynonymous SNV
ORF1b:cds-YP_009724389.1:exon1:c.T4264C:p.F1422L 1.65 2.16297 1 0.05

D . #ERROR! . . . . . orf1ab_RPQIGVVREF
0.08 . . . .
1 17761 17761 G T exonic ORF1b. nonsynonymous SNV
ORF1b:cds-YP_009724389.1:exon1:c.G4294T:p.A1432S 0.735 2.70561 1 0
D . #ERROR! . . . . . orf1ab_RNPAWRKAVF
-1 . . nCoV-2019_58_RIGHT . . . . .

1 17766 17766 C T exonic ORF1b. synonymous SNV
ORF1b:cds-YP_009724389.1:exon1:c.C4299T:p.V1433V -0.763 0.380016 1 1
T . #ERROR! . . . . . orf1ab_RNPAWRKAVF
-1 . . . .
1 17885 17885 C T exonic ORF1b. nonsynonymous SNV
ORF1b:cds-YP_009724389.1:exon1:c.C4418T:p.T1473I 1.65 3.32576 1 0
D . #ERROR! . . . .
. . . . . .
1 17893 17893 G T exonic ORF1b. nonsynonymous SNV
ORF1b:cds-YP_009724389.1:exon1:c.G4426T:p.A1476S 1.65 4.256 1 0.04 D
. #ERROR! . . . .
. . . . .
1 17922 17922 T C exonic ORF1b. synonymous SNV
ORF1b:cds-YP_009724389.1:exon1:c.T4455C:p.N1485N 0.48 1.00017 1 1
T . #ERROR! . . . orf1ab polyprotein_NVNRFNVAITRAKVG
19 . . . .

1 17944 17944 G A exonic ORF1b. nonsynonymous SNV
ORF1b:cds-YP_009724389.1:exon1:c.G4477A:p.V1493I -0.687 1.46529 1 0.18
T . #ERROR! . . . orf1ab polyprotein_NVNRFNVAITRAKVG
19 . . . .

1 17969 17969 A G exonic ORF1b. nonsynonymous SNV
ORF1b:cds-YP_009724389.1:exon1:c.A4502G:p.D1501G 1.65 2.24049 1 0.08
T . #ERROR! . . . .
nCoV-2019_60_LEFT. . . . .
1 17979 17979 T C exonic ORF1b. synonymous SNV
ORF1b:cds-YP_009724389.1:exon1:c.T4512C:p.L1504L -0.832 0.380016 1 1
T . #ERROR! . . . .
nCoV-2019_60_LEFT. . . . .
1 17999 17999 C T exonic ORF1b. nonsynonymous SNV
ORF1b:cds-YP_009724389.1:exon1:c.C4532T:p.T1511I 0.78 1.77537 0.976685
0.47 T . #ERROR! . . . . . orf1ab_YDKLQFTSL
0.12 . . . .
1 18002 18002 G A exonic ORF1b. nonsynonymous SNV
ORF1b:cds-YP_009724389.1:exon1:c.G4535A:p.S1512N 1.65 4.256 1 0.15 T

. #ERROR! . . . . . orf1ab_YDKLQFTSL 0.12 .
. . . .
1 18021 18021 G T exonic ORF1b. nonsynonymous SNV
ORF1b:cds-YP_009724389.1:exon1:c.G4554T:p.R1518S -3.3 -2.48821 0.362724
0.54 T . #ERROR! . . . . . orf1ab_IPRRNVATL
-1 . . . .
1 18032 18032 C T exonic ORF1b. nonsynonymous SNV
ORF1b:cds-YP_009724389.1:exon1:c.C4565T:p.T1522I 0.68 1.62033 1 0.19
T . #ERROR! . . . . . orf1ab_IPRRNVATL -1
. . . .
1 18048 18048 T C exonic ORF1b. synonymous SNV
ORF1b:cds-YP_009724389.1:exon1:c.T4581C:p.N1527N 0.484 1.00017 1 0.5
T . #ERROR! . . . . . orf1ab_AENVTGLF 0.14
. . nCoV-2019_59_RIGHT . . . . .

1 18102 18102 A G exonic ORF1b. synonymous SNV
ORF1b:cds-YP_009724389.1:exon1:c.A4635G:p.T1545T 1.65 2.24049 1 0.29
T . . . . orf1ab_HPTQAPTHL -1 .
. . . .
1 18109 18109 C T exonic ORF1b. nonsynonymous SNV
ORF1b:cds-YP_009724389.1:exon1:c.C4642T:p.P1548S 1.65 3.32576 1 0.21
T . . . . orf1ab_HPTQAPTHL -1 .
. . . .
1 18149 18149 G T exonic ORF1b. nonsynonymous SNV
ORF1b:cds-YP_009724389.1:exon1:c.G4682T:p.G1561V 1.65 4.256 1 0.01 D
. . . .
. . . .
1 18186 18186 G T exonic ORF1b. nonsynonymous SNV
ORF1b:cds-YP_009724389.1:exon1:c.G4719T:p.M1573I 1.65 4.256 1 0.41 T
. . . . . orf1ab polyprotein_PKDMTYRRLISMMGF 9.4
orf1ab_IPGIPKDMTY 0.03 . . . .

1 18255 18255 G T exonic ORF1b. nonsynonymous SNV
ORF1b:cds-YP_009724389.1:exon1:c.G4788T:p.M1596I 1.65 4.256 1 0.02 D
. . . . . orf1ab polyprotein_NMFITREEAIRHVRA 14 .
. . . nCoV-2019_61_LEFT . . . . .

1 18266 18266 G T exonic ORF1b. nonsynonymous SNV
ORF1b:cds-YP_009724389.1:exon1:c.G4799T:p.R1600L 1.65 4.256 1 0 D
. . . . . orf1ab polyprotein_NMFITREEAIRHVRA 14
orf1ab_REEAIRHVRAW -1 . . nCoV-2019_61_LEFT . . .
. .
1 18281 18281 G T exonic ORF1b. nonsynonymous SNV

ORF1b:cds-YP_009724389.1:exon1:c.G4814T:p.R1605I 0.646 2.24049 1 0
D . . . . . orf1ab polyprotein_NMFITREEAIRHVRA 14
orf1ab_REEAIRHVRAW -1 . . . .

1 18309 18309 T C exonic ORF1b. synonymous SNV
ORF1b:cds-YP_009724389.1:exon1:c.T4842C:p.D1614D -3.3 -0.55022 0.976685
1 T . . . . . orf1ab polyprotein_REEAIRHVRAWIGFD
16 . . . .

1 18395 18395 C G exonic ORF1b. nonsynonymous SNV
ORF1b:cds-YP_009724389.1:exon1:c.C4928G:p.A1643G 1.65 3.32576 1 0.01
D . . . .
. . . . .
1 18395 18395 C T exonic ORF1b. nonsynonymous SNV
ORF1b:cds-YP_009724389.1:exon1:c.C4928T:p.A1643V 1.65 3.32576 1 1
T . . . .
. . . . .
1 18456 18456 A G exonic ORF1b. synonymous SNV
ORF1b:cds-YP_009724389.1:exon1:c.A4989G:p.K1663K 0.529 0.997417 1 1
T . . . .
. . . . .
1 18483 18483 C T exonic ORF1b. synonymous SNV
ORF1b:cds-YP_009724389.1:exon1:c.C5016T:p.H1672H 0.488 0.264236 1 1
T . . . . . orf1ab polyprotein_PPGDQFKHLIPLMYK 19
. . . .

1 18486 18486 C T exonic ORF1b. synonymous SNV
ORF1b:cds-YP_009724389.1:exon1:c.C5019T:p.L1673L 0.412 0.182772 1 1
T . . . . . orf1ab polyprotein_PPGDQFKHLIPLMYK 19
. . . .

1 18498 18498 G T exonic ORF1b. nonsynonymous SNV
ORF1b:cds-YP_009724389.1:exon1:c.G5031T:p.M1677I 1.65 4.256 1 0.02 D
. . . . . orf1ab polyprotein_PPGDQFKHLIPLMYK 19 .
. . . .
1 18508 18508 C T exonic ORF1b. nonsynonymous SNV
ORF1b:cds-YP_009724389.1:exon1:c.C5041T:p.L1681F -2.2 0.182772 0.929134
0.03 D . . . . . orf1ab polyprotein_FKHLIPLMYKGLPWN
20 . . . .

1 18555 18555 C T exonic ORF1b. synonymous SNV
ORF1b:cds-YP_009724389.1:exon1:c.C5088T:p.D1696D -3.3 -1.0392 0.952756
1 T . . . . . orf1ab polyprotein_VVRIKIVQMLSDTLK

19	orf1ab_MLSDTLKNL	0.16	.	.	.	.	.	.	.	.

1	18570	18570	C	T	exonic	ORF1b.	synonymous SNV
ORF1b:cds-YP_009724389.1:exon1:c.C5103T:p.L1701L	-3.3	-0.468945	0.944882
1	T	.	.	.	.	.	orf1ab polyprotein_IVQMLSDTLKNLSDR
15	orf1ab_MLSDTLKNL	0.16	.	.	.	.	.	.	.	.

1	18576	18576	C	T	exonic	ORF1b.	synonymous SNV
ORF1b:cds-YP_009724389.1:exon1:c.C5109T:p.D1703D	-0.187	1.16035	1	1
T	.	.	.	.	.	orf1ab polyprotein_IVQMLSDTLKNLSDR	15
orf1ab_NLSDRVVFV	0.03	.	.	.	.	.	.	.	.

1	18590	18590	T	C	exonic	ORF1b.	nonsynonymous SNV
ORF1b:cds-YP_009724389.1:exon1:c.T5123C:p.V1708A	1.65	2.21939	1	0
D	.	.	.	.	.	.	.	orf1ab_NLSDRVVFV	0.03	.
.	.	.	.	.	.	.
1	18651	18651	G	T	exonic	ORF1b.	nonsynonymous SNV
ORF1b:cds-YP_009724389.1:exon1:c.G5184T:p.E1728D	-1.93	0.101307	1	0.16
T	.	.	.	.	.	.	.	.	.	.	.
nCoV-2019_61_RIGHT	.	.	.	.	.
1	18677	18677	G	T	exonic	ORF1b.	nonsynonymous SNV
ORF1b:cds-YP_009724389.1:exon1:c.G5210T:p.R1737L	1.65	4.256	1	0.03	D
.	.	.	.	.	.	.	.	.	.	.	.	.
.	.	.	.
1	18687	18687	C	T	exonic	ORF1b.	synonymous SNV
ORF1b:cds-YP_009724389.1:exon1:c.C5220T:p.C1740C	-2.38	0.0198425	0.992126
1	T	.	.	.	.	.	.	.	.	.	.	.
.	.	.	.	.	.
1	18699	18699	T	C	exonic	ORF1b.	synonymous SNV
ORF1b:cds-YP_009724389.1:exon1:c.T5232C:p.A1744A	0.485	0.915953	1	0.73
T	.	.	.	.	.	.	.	.	.	.	.	.
.	.	.	.	.
1	18747	18747	C	T	exonic	ORF1b.	synonymous SNV
ORF1b:cds-YP_009724389.1:exon1:c.C5280T:p.V1760V	1.65	3.27843	1	1
T	.	.	.	.	.	orf1ab polyprotein_IGFDYVYNPFMIDVQ	16
orf1ab_HSIGFDYVY	0.07	.	.	.	.	.	.	.	.

1	18802	18802	A	C	exonic	ORF1b.	nonsynonymous SNV
ORF1b:cds-YP_009724389.1:exon1:c.A5335C:p.S1779R	1.65	2.21939	1	0.27
T	.	.	.	.	.	.	.	.	.	.	.	.
.	.	.	.	.
1	18828	18828	C	T	exonic	ORF1b.	synonymous SNV
ORF1b:cds-YP_009724389.1:exon1:c.C5361T:p.V1787V	-1.67	0.590095	1	1

T . . . .
. . . . .
1 18877 18877 C T exonic ORF1b. synonymous SNV
ORF1b:cds-YP_009724389.1:exon1:c.C5410T:p.L1804L -3.3 0.101307 0.96063
1 T . . . .
. . . . . .
1 18928 18928 C T exonic ORF1b. nonsynonymous SNV
ORF1b:cds-YP_009724389.1:exon1:c.C5461T:p.P1821S 1.65 3.27843 1 0
D . . . . orf1ab_IEYPIIGDEL 0.04 .
. . . .
1 18929 18929 C T exonic ORF1b. nonsynonymous SNV
ORF1b:cds-YP_009724389.1:exon1:c.C5462T:p.P1821L 1.65 3.27843 0.984252
0 D . . . . orf1ab_IEYPIIGDEL 0.04
. . . .
1 18932 18932 T A exonic ORF1b. nonsynonymous SNV
ORF1b:cds-YP_009724389.1:exon1:c.T5465A:p.I1822K 1.65 2.21939 0.952756
0.17 T . . . . orf1ab_IEYPIIGDEL 0.04
. . . .
1 18972 18972 G T exonic ORF1b. nonsynonymous SNV
ORF1b:cds-YP_009724389.1:exon1:c.G5505T:p.K1835N -0.711 0.50863 1 0.34
T . . . . . orf1ab polyprotein_KVQHMVVKAALLADK 16
. . . . nCoV-2019_62_RIGHT . . . . .

1 18974 18974 T C exonic ORF1b. nonsynonymous SNV
ORF1b:cds-YP_009724389.1:exon1:c.T5507C:p.V1836A 1.65 2.21939 1 0.01
D . . . . . orf1ab polyprotein_KVQHMVVKAALLADK 16
. . . . nCoV-2019_62_RIGHT . . . . .

1 18984 18984 G T exonic ORF1b. nonsynonymous SNV
ORF1b:cds-YP_009724389.1:exon1:c.G5517T:p.M1839I 1.65 4.256 1 0.59 T
. . . . . orf1ab polyprotein_KVQHMVVKAALLADK 16
orf1ab_HMVVKAAL 0.09 . . . .

1 19009 19009 G T exonic ORF1b. nonsynonymous SNV
ORF1b:cds-YP_009724389.1:exon1:c.G5542T:p.D1848Y 1.65 4.256 1 1 T
. . . . . orf1ab polyprotein_KVQHMVVKAALLADK 16
orf1ab_LLADKFPVL -1 . . . .

1 19026 19026 T C exonic ORF1b. synonymous SNV
ORF1b:cds-YP_009724389.1:exon1:c.T5559C:p.L1853L -0.818 0.427165 1 0.38
T . . . . orf1ab_LLADKFPVL -1 .
. . . .
1 19029 19029 C T exonic ORF1b. synonymous SNV

ORF1b:cds-YP_009724389.1:exon1:c.C5562T:p.H1854H -3.3 -0.306016 0.992126
0.53 T . . . . orf1ab_VLHDIGNPK 0.07
. . . .
1 19072 19072 G T exonic ORF1b. nonsynonymous SNV
ORF1b:cds-YP_009724389.1:exon1:c.G5605T:p.D1869Y 1.65 4.256 1 0.01 D
. . . .
. . . .
1 19109 19109 G T exonic ORF1b. nonsynonymous SNV
ORF1b:cds-YP_009724389.1:exon1:c.G5642T:p.S1881I 0.745 2.70817 1 0.06
T . . . .
. . . . .
1 19173 19173 A G exonic ORF1b. synonymous SNV
ORF1b:cds-YP_009724389.1:exon1:c.A5706G:p.T1902T -1.05 0.264236 1 0.67
T . . . . orf1ab_KFTDGVCLF -1 .
. . . .
1 19190 19190 T A exonic ORF1b. nonsynonymous SNV
ORF1b:cds-YP_009724389.1:exon1:c.T5723A:p.F1908Y 1.65 2.21939 1 0
D . . . . orf1ab_KFTDGVCLF -1 .
. . . .
1 19197 19197 T C exonic ORF1b. synonymous SNV
ORF1b:cds-YP_009724389.1:exon1:c.T5730C:p.N1910N -3.3 -0.713339 0.92126
1 T . . . .
. . . . . .
1 19203 19203 T C exonic ORF1b. synonymous SNV
ORF1b:cds-YP_009724389.1:exon1:c.T5736C:p.N1912N -0.134 1.16035 1 1
T . . . .
. . . . .
1 19308 19308 A G exonic ORF1b. synonymous SNV
ORF1b:cds-YP_009724389.1:exon1:c.A5841G:p.K1947K -3.3 0.101307 0.992126
1 T . . . . orf1ab_SLYVNKHAF 0.12
. . . .
1 19524 19524 C T exonic ORF1b. synonymous SNV
ORF1b:cds-YP_009724389.1:exon1:c.C6057T:p.L2019L -3.3 -0.932575 0.944882
1 T . . . . . orf1ab polyprotein_YRLYLDAYNMMISAG
17 orf1ab_HANEYRLYL 0.19 . . . .

1 19525 19525 G T exonic ORF1b. nonsynonymous SNV
ORF1b:cds-YP_009724389.1:exon1:c.G6058T:p.D2020Y 0.746 2.68964 1 0.01
D . . . . . orf1ab polyprotein_YRLYLDAYNMMISAG 17
orf1ab_HANEYRLYL 0.19 . . . .

1 19542 19542 G T exonic ORF1b. nonsynonymous SNV
ORF1b:cds-YP_009724389.1:exon1:c.G6075T:p.M2025I 1.65 4.256 1 0.63 T

. . . . . orf1ab polyprotein_YRLYLDAYNMMISAG 17
orf1ab_LYLDAYNMM -1 . . . .

1 19581 19581 T C exonic ORF1b. synonymous SNV
ORF1b:cds-YP_009724389.1:exon1:c.T6114C:p.F2038F 0.482 0.92748 1 1
T . . . . orf1ab_VYKQFDTYNLW -1
. . . .
1 19632 19632 T C exonic ORF1b. synonymous SNV
ORF1b:cds-YP_009724389.1:exon1:c.T6165C:p.N2055N 1.65 2.20015 1 1
T . . . . orf1ab_SLENVAFNV 0.12 .
. . . .
1 19645 19645 G T exonic ORF1b. nonsynonymous SNV
ORF1b:cds-YP_009724389.1:exon1:c.G6178T:p.V2060F 1.65 4.256 1 0.09 T
. . . . orf1ab_SLENVAFNV 0.12 . .
. . . . . .
1 19718 19718 C T exonic ORF1b. nonsynonymous SNV
ORF1b:cds-YP_009724389.1:exon1:c.C6251T:p.T2084I 0.791 1.90646 1 0.21
T . . . . orf1ab_SIINNTVYTK -1 .
. . . .
1 19720 19720 A G exonic ORF1b. nonsynonymous SNV
ORF1b:cds-YP_009724389.1:exon1:c.A6253G:p.K2085E 1.65 2.29805 0.992126
0.03 D . . . . orf1ab_SIINNTVYTK -1
. . . .
1 19723 19723 G T exonic ORF1b. nonsynonymous SNV
ORF1b:cds-YP_009724389.1:exon1:c.G6256T:p.V2086F -0.258 1.61276 1 0.71
T . . . . orf1ab_SIINNTVYTK -1 .
. . . .
1 19763 19763 C T exonic ORF1b. nonsynonymous SNV
ORF1b:cds-YP_009724389.1:exon1:c.C6296T:p.T2099I 0.788 1.90646 1 0.09
T . . . . orf1ab_VELFENKTTL -1 .
. . . .
1 19795 19795 G T exonic ORF1b. nonsynonymous SNV
ORF1b:cds-YP_009724389.1:exon1:c.G6328T:p.A2110S 1.65 4.256 1 0.01 D
. . . .
. . . .
1 19802 19802 G A exonic ORF1b. nonsynonymous SNV
ORF1b:cds-YP_009724389.1:exon1:c.G6335A:p.R2112H 1.65 4.256 1 0 D
. . . .
. . . .
1 19806 19806 C T exonic ORF1b. synonymous SNV
ORF1b:cds-YP_009724389.1:exon1:c.C6339T:p.N2113N -0.14 1.22117 1 0.69
T . . . .
. . . . .

1 19813 19813 C T exonic ORF1b. nonsynonymous SNV
ORF1b:cds-YP_009724389.1:exon1:c.C6346T:p.P2116S 0.749 1.80856 1 0.74
T . . . . orf1ab_KPVPEVKIL 0.04 .
. . . .
1 19868 19868 C T exonic ORF1b. nonsynonymous SNV
ORF1b:cds-YP_009724389.1:exon1:c.C6401T:p.T2134I 1.65 1.90646 1 0.33
T . . . . . orf1ab polyprotein_VKILNNLGVDIAANT 16
. . . .

1 19889 19889 G A exonic ORF1b. nonsynonymous SNV
ORF1b:cds-YP_009724389.1:exon1:c.G6422A:p.R2141K 0.739 2.68964 1 0.85
T . . . . . orf1ab polyprotein_VIWDYKRDAPAHIST 12
. . . .

1 19947 19947 G T exonic ORF1b. nonsynonymous SNV
ORF1b:cds-YP_009724389.1:exon1:c.G6480T:p.K2160N 1.65 4.256 1 0.34 T
. . . .
. . . .
1 19960 19960 A G exonic ORF1b. nonsynonymous SNV
ORF1b:cds-YP_009724389.1:exon1:c.A6493G:p.T2165A 0.46 0.92748 0.976378
0.18 T . . . .
. . . . . .
1 19962 19962 G T exonic ORF1b. synonymous SNV
ORF1b:cds-YP_009724389.1:exon1:c.G6495T:p.T2165T -2.14 -0.247291 0.968504
0.26 T . . . .
. . . . . .
1 19963 19963 A G exonic ORF1b. nonsynonymous SNV
ORF1b:cds-YP_009724389.1:exon1:c.A6496G:p.I2166V -0.222 1.80856 0.992126
1 T . . . .
. . . . . .
1 19980 19980 T C exonic ORF1b. synonymous SNV
ORF1b:cds-YP_009724389.1:exon1:c.T6513C:p.T2171T -3.3 -1.42206 0.866142
0.36 T . . . .
. . . . . .
1 19983 19983 C T exonic ORF1b. synonymous SNV
ORF1b:cds-YP_009724389.1:exon1:c.C6516T:p.V2172V 1.65 3.27702 1 1
T . . . . orf1ab_VFFDGRVDGQVDLF 0.08
. . . .
1 20011 20011 G T exonic ORF1b. nonsynonymous SNV
ORF1b:cds-YP_009724389.1:exon1:c.G6544T:p.V2182L 1.65 4.256 1 1 T
. . . . . orf1ab polyprotein_GQVDLFRNARNGVLI 9
orf1ab_VFFDGRVDGQVDLF 0.08 . . . .

1 20055 20055 A G exonic ORF1b. synonymous SNV
ORF1b:cds-YP_009724389.1:exon1:c.A6588G:p.E2196E -0.718 0.437992 1 1
T . . . .
. . . . .
1 20079 20079 A G exonic ORF1b. synonymous SNV
ORF1b:cds-YP_009724389.1:exon1:c.A6612G:p.P2204P -3.3 -0.638882 0.944882
0.29 T . . . . orf1ab_GLQPSVGPK 0.12
. . . .
1 20178 20178 C T exonic ORF1b. synonymous SNV
ORF1b:cds-YP_009724389.1:exon1:c.C6711T:p.V2237V -2.16 -0.345189 0.976378
1 T . . . .
nCoV-2019_67_LEFT . . . . .
1 20205 20205 T C exonic ORF1b. synonymous SNV
ORF1b:cds-YP_009724389.1:exon1:c.T6738C:p.T2246T -0.817 0.340094 1 1
T . . . .
. . . . .
1 20275 20275 G T exonic ORF1b. nonsynonymous SNV
ORF1b:cds-YP_009724389.1:exon1:c.G6808T:p.D2270Y 1.65 4.256 1 0.02 D
. . . . orf1ab_MEIDFLELAM 0.12 . .
. . . . . .
1 20380 20380 C T exonic ORF1b. synonymous SNV
ORF1b:cds-YP_009724389.1:exon1:c.C6913T:p.L2305L -2.64 0.144299 0.866142
1 T . . . . . orf1ab polyprotein_QLGGLHLLIGLAKRF
19 . . . .

1 20389 20389 C T exonic ORF1b. nonsynonymous SNV
ORF1b:cds-YP_009724389.1:exon1:c.C6922T:p.R2308C -1.52 0.437992 0.984252
0.01 D . . . . . orf1ab polyprotein_QLGGLHLLIGLAKRF
19 . . . .

1 20390 20390 G T exonic ORF1b. nonsynonymous SNV
ORF1b:cds-YP_009724389.1:exon1:c.G6923T:p.R2308L 0.75 2.78754 0.968504
0.3 T . . . . . orf1ab polyprotein_QLGGLHLLIGLAKRF
19 . . . .

1 20405 20405 C T exonic ORF1b. nonsynonymous SNV
ORF1b:cds-YP_009724389.1:exon1:c.C6938T:p.P2313L -1.52 0.242197 0.834646
0.86 T . . . .
. . . . . .
1 20434 20434 G T exonic ORF1b. nonsynonymous SNV
ORF1b:cds-YP_009724389.1:exon1:c.G6967T:p.D2323Y 1.65 4.256 1 0 D
. . . . . orf1ab polyprotein_ELEDFIPMDSTVKNY 20
orf1ab_FELEDFIPM 0.08 . . . .

1 20458 20458 A T exonic ORF1b. nonsynonymous SNV
ORF1b:cds-YP_009724389.1:exon1:c.A6991T:p.I2331L 0.48 1.02538 1 0.02
D . . . .
. . . . .
1 20474 20474 C T exonic ORF1b. nonsynonymous SNV
ORF1b:cds-YP_009724389.1:exon1:c.C7007T:p.T2336I 1.65 3.27702 1 0.05
D . . . .
nCoV-2019_68_LEFT. . . . .
1 20477 20477 G T exonic ORF1b. nonsynonymous SNV
ORF1b:cds-YP_009724389.1:exon1:c.G7010T:p.G2337V 1.65 4.256 1 0.09 T
. . . .
nCoV-2019_68_LEFT. . . . .
1 20480 20480 C T exonic ORF1b. nonsynonymous SNV
ORF1b:cds-YP_009724389.1:exon1:c.C7013T:p.S2338L 1.65 3.27702 1 0.01
D . . . .
nCoV-2019_68_LEFT. . . . .
1 20555 20555 C T exonic ORF1b. nonsynonymous SNV
ORF1b:cds-YP_009724389.1:exon1:c.C7088T:p.S2363F 1.65 3.34955 1 0.01
D . . . . . orf1ab polyprotein_EIIKSQDLSVVSKVV 18
. . . . nCoV-2019_67_RIGHT . . . . .

1 20568 20568 G A exonic ORF1b. synonymous SNV
ORF1b:cds-YP_009724389.1:exon1:c.G7101A:p.K2367K -3.3 -0.175528 0.984252
1 T . . . . . orf1ab polyprotein_EIIKSQDLSVVSKVV
18 . . . . nCoV-2019_67_RIGHT . . . .
.
1 20580 20580 G T exonic ORF1b. synonymous SNV
ORF1b:cds-YP_009724389.1:exon1:c.G7113T:p.V2371V -3.3 -0.276244 0.992126
1 T . . . .
. . . . . .
1 20616 20616 G T exonic ORF1b. nonsynonymous SNV
ORF1b:cds-YP_009724389.1:exon1:c.G7149T:p.W2383C 1.65 4.256 1 0 D
. . . . orf1ab_TEISFMLW -1 . .
. . . . . .
1 20621 20621 A G exonic ORF1b. nonsynonymous SNV
ORF1b:cds-YP_009724389.1:exon1:c.A7154G:p.K2385R 1.65 2.24167 0.992126
0.2 T . . . .
. . . . . .
1 20623 20623 G T exonic ORF1b. nonsynonymous SNV
ORF1b:cds-YP_009724389.1:exon1:c.G7156T:p.D2386Y 0.739 2.74525 0.992126
0 D . . . .
. . . . . .

1 20663 20663 G T exonic ORF1b. nonsynonymous SNV
ORF1b:cds-YP_009724389.1:exon1:c.G7196T:p.S2399I 0.715 2.64454 1 0.09
T . . . .
. . . . .
1 20679 20679 G T exonic ORF1b. synonymous SNV
ORF1b:cds-YP_009724389.1:exon1:c.G7212T:p.P2404P -3.3 -0.578394 0.897638
0.95 T . . . . orf1ab_QAWQPGVAM
0.05 . . . .
1 20759 20759 C T exonic ORF1b. nonsynonymous SNV
ORF1b:cds-YP_009724389.1:exon1:c.C7292T:p.A2431V 1.65 3.34955 1 0.68
T . . . .
. . . . .
1 20772 20772 A C exonic ORF1b. nonsynonymous SNV
ORF1b:cds-YP_009724389.1:exon1:c.A7305C:p.K2435N -1.99 0.126622 1 0.49
T . . . . . orf1ab polyprotein_KGIMMNVAKYTQLCQ 20
orf1ab_LPKGIMMNV 0.15 . . . .

1 20806 20806 C A exonic ORF1b. nonsynonymous SNV
ORF1b:cds-YP_009724389.1:exon1:c.C7339A:p.L2447M 1.65 3.34955 1 0
D . . . . . orf1ab polyprotein_KGIMMNVAKYTQLCQ 20
orf1ab_NVAKYTQL 0.1 . . nCoV-2019_69_LEFT . . . .
.
1 20922 20922 G T exonic ORF1b. nonsynonymous SNV
ORF1b:cds-YP_009724389.1:exon1:c.G7455T:p.W2485C 1.65 4.256 1 0 D
. . . .
. . . .
1 20976 20976 T C exonic ORF1b. synonymous SNV
ORF1b:cds-YP_009724389.1:exon1:c.T7509C:p.D2503D 0.627 0.730921 1 1
T . . . .
. . . . .
1 21001 21001 T C exonic ORF1b. nonsynonymous SNV
ORF1b:cds-YP_009724389.1:exon1:c.T7534C:p.C2512R 1.65 2.14095 1 0
D . . . . orf1ab_TLIGDCATV 0.09 .
. . . .
1 21034 21034 C T exonic ORF1b. nonsynonymous SNV
ORF1b:cds-YP_009724389.1:exon1:c.C7567T:p.L2523F 1.65 3.34955 0.984252
0.06 T . . . .
. . . . . .
1 21035 21035 T C exonic ORF1b. nonsynonymous SNV
ORF1b:cds-YP_009724389.1:exon1:c.T7568C:p.L2523P 1.65 2.14095 0.992126
0 D . . . .
. . . . . .
1 21108 21108 C T exonic ORF1b. synonymous SNV

ORF1b:cds-YP_009724389.1:exon1:c.C7641T:p.F2547F -0.171 1.13379 1 1
T . . . . orf1ab_KENDSKEGFFTY -1
. . . .
1 21204 21204 G A exonic ORF1b. synonymous SNV
ORF1b:cds-YP_009724389.1:exon1:c.G7737A:p.K2579K 1.65 4.256 1 0.76 T
. . . . orf1ab_HSWNADLYK 0.06 . .
. . . . . .
1 21220 21220 G C exonic ORF1b. nonsynonymous SNV
ORF1b:cds-YP_009724389.1:exon1:c.G7753C:p.A2585P -0.889 0.126622 1 0.05
D . . . .
. . . . .
1 21296 21296 G T exonic ORF1b. nonsynonymous SNV
ORF1b:cds-YP_009724389.1:exon1:c.G7829T:p.G2610V 1.65 4.256 0.992126 0
D . . . .
. . . . .
1 21304 21304 C T exonic ORF1b. nonsynonymous SNV
ORF1b:cds-YP_009724389.1:exon1:c.C7837T:p.R2613C 0.509 0.328055 0.0314961
0.06 T . . . . orf1ab_KPREQIDGYVM
-1 . . . .
1 21306 21306 C T exonic ORF1b. synonymous SNV
ORF1b:cds-YP_009724389.1:exon1:c.C7839T:p.R2613R -3.3 -1.28341 0.0314961
0.39 T . . . . orf1ab_KPREQIDGYVM
-1 . . . .
1 21334 21334 G T exonic ORF1b. nonsynonymous SNV
ORF1b:cds-YP_009724389.1:exon1:c.G7867T:p.A2623S 1.65 4.256 1 0 D
. . . . . orf1ab polyprotein_QIDGYVMHANYIFWR 6.7 .
. . . .
1 21339 21339 T C exonic ORF1b. synonymous SNV
ORF1b:cds-YP_009724389.1:exon1:c.T7872C:p.N2624N -1.31 0.227339 1 1
T . . . . . orf1ab polyprotein_QIDGYVMHANYIFWR 6.7
. . . .

1 21575 21575 C T exonic S . nonsynonymous SNV
S:cds-YP_009724390.1:exon1:c.C13T:p.L5F -3.25 -7.36907 0 0.05 D
. . . .
. . homoplasic position .
1 21614 21614 C T exonic S . nonsynonymous SNV
S:cds-YP_009724390.1:exon1:c.C52T:p.L18F -3.16 -3.40888 0 0.04 D
disulf_bond BetaCoV_S1-NTD . . Surface glycoprotein_LVSSQCVNLTTRT
. . surface glycoprotein_NLTTRTQL 0.12 . . . . .
. . .
1 21620 21620 A G exonic S . nonsynonymous SNV
S:cds-YP_009724390.1:exon1:c.A58G:p.T20A -3.1 -1.36491 0 0.56 T

disulf_bond	BetaCoV_S1-NTD	.	.	Surface glycoprotein_LVSSQCVNLTTRT
.	.	surface glycoprotein_NLTTRTQL	0.12	.	.	.	.	.
.	.	.
1	21621	21621	C	T	exonic	S	.	nonsynonymous SNV
S:cds-YP_009724390.1:exon1:c.C59T:p.T20I	-3.1	-5.19735	0	0.12	T
disulf_bond	BetaCoV_S1-NTD	.	.	Surface glycoprotein_LVSSQCVNLTTRT
.	.	surface glycoprotein_NLTTRTQL	0.12	.	.	.	.	.
.	.	.
1	21624	21624	G	C	exonic	S	.	nonsynonymous SNV
S:cds-YP_009724390.1:exon1:c.G62C:p.R21T	-3.1	-4.81411	0	0.4	T
disulf_bond	BetaCoV_S1-NTD	.	.	Surface glycoprotein_LVSSQCVNLTTRT
.	.	surface glycoprotein_NLTTRTQL	0.12	.	.	.	.	.
.	.	.
1	21637	21637	C	T	exonic	S	.	synonymous SNV
S:cds-YP_009724390.1:exon1:c.C75T:p.P25P	-3.29	-1.10942	0	0.35	T
disulf_bond	BetaCoV_S1-NTD	.	.	.	.	.	surface
glycoprotein_LPPAYTNSF	0.04	.	.	.	.	.	.	.	.

1	21638	21638	C	T	exonic	S	.	nonsynonymous SNV
S:cds-YP_009724390.1:exon1:c.C76T:p.P26S	-3.3	0.168063	0	0.55	T
disulf_bond	BetaCoV_S1-NTD	.	.	.	.	.	surface
glycoprotein_LPPAYTNSF	0.04	.	.	.	.	.	.	.	.

1	21641	21641	G	T	exonic	S	.	nonsynonymous SNV
S:cds-YP_009724390.1:exon1:c.G79T:p.A27S	-3.3	0.040315	0.00787402	0.99
T	disulf_bond	BetaCoV_S1-NTD	.	.	.	.	.	surface
glycoprotein_LPPAYTNSF	0.04	.	.	.	.	.	.	.	.

1	21646	21646	C	T	exonic	S	.	synonymous SNV
S:cds-YP_009724390.1:exon1:c.C84T:p.Y28Y	-3.2	0.040315	0.00787402	1
T	disulf_bond	BetaCoV_S1-NTD	.	.	.	.	.	surface
glycoprotein_LPPAYTNSF	0.04	.	.	.	.	.	.	.	.

1	21654	21654	C	T	exonic	S	.	nonsynonymous SNV
S:cds-YP_009724390.1:exon1:c.C92T:p.S31F	1.65	3.23402	0.0944882	0.01
D	disulf_bond	BetaCoV_S1-NTD	.	.	.	.	.	surface
glycoprotein_LPPAYTNSF	0.04	.	.	.	.	.	.	.	.

1	21658	21658	C	T	exonic	S	.	synonymous SNV
S:cds-YP_009724390.1:exon1:c.C96T:p.F32F	-3.3	-4.04762	0	0.7	T
disulf_bond	BetaCoV_S1-NTD	.	.	.	.	.	surface
glycoprotein_LPPAYTNSF	0.04	.	.	nCoV-2019_72_LEFT	.	.	.
.	.

1 21660 21660 C T exonic S . nonsynonymous SNV
S:cds-YP_009724390.1:exon1:c.C98T:p.T33I -3.3 -0.853921 0 0.35 T
disulf_bond BetaCoV_S1-NTD . . . .
. nCoV-2019_72_LEFT . . . . .
1 21691 21691 C T exonic S . synonymous SNV
S:cds-YP_009724390.1:exon1:c.C129T:p.F43F -3.3 -0.342929 0 1 T
disulf_bond BetaCoV_S1-NTD . . . . . surface
glycoprotein_KVFRSSVLH 0.14 . . . .

1 21724 21724 G A exonic S . synonymous SNV
S:cds-YP_009724390.1:exon1:c.G162A:p.L54L -3.3 -6.73033 0 1 T
disulf_bond BetaCoV_S1-NTD . . . surface
glycoprotein_TQDLFLPFFSNVTWF 19 . . . .
nCoV-2019_71_RIGHT . . . . .
1 21724 21724 G C exonic S . nonsynonymous SNV
S:cds-YP_009724390.1:exon1:c.G162C:p.L54F -3.3 -6.73033 0 0.69 T
disulf_bond BetaCoV_S1-NTD . . . surface
glycoprotein_TQDLFLPFFSNVTWF 19 . . . .
nCoV-2019_71_RIGHT . . . . .
1 21724 21724 G T exonic S . nonsynonymous SNV
S:cds-YP_009724390.1:exon1:c.G162T:p.L54F -3.3 -6.73033 0 0.69 T
disulf_bond BetaCoV_S1-NTD . . . surface
glycoprotein_TQDLFLPFFSNVTWF 19 . . . .
nCoV-2019_71_RIGHT . . . . .
1 21736 21736 C T exonic S . synonymous SNV
S:cds-YP_009724390.1:exon1:c.C174T:p.F58F -3.3 -1.62041 0 1 T
disulf_bond BetaCoV_S1-NTD . . . surface
glycoprotein_TQDLFLPFFSNVTWF 19 surface glycoprotein_LPFFSNVTW 0.08 .
. nCoV-2019_71_RIGHT . . . . .

1 21737 21737 T C exonic S . nonsynonymous SNV
S:cds-YP_009724390.1:exon1:c.T175C:p.F59L -3.3 -4.43087 0 0.72 T
disulf_bond BetaCoV_S1-NTD . . . surface
glycoprotein_TQDLFLPFFSNVTWF 19 surface glycoprotein_LPFFSNVTW 0.08 .
. nCoV-2019_71_RIGHT . . . . .

1 21742 21742 C A exonic S . synonymous SNV
S:cds-YP_009724390.1:exon1:c.C180A:p.S60S -3.3 -9.54079 0 1 T
disulf_bond BetaCoV_S1-NTD glyco . . surface
glycoprotein_TQDLFLPFFSNVTWF 19 surface glycoprotein_LPFFSNVTW 0.08 .
. nCoV-2019_71_RIGHT . . . . .

1 21742 21742 C T exonic S . synonymous SNV

S:cds-YP_009724390.1:exon1:c.C180T:p.S60S	-3.3	-9.54079	0	1	T
disulf_bond	BetaCoV_S1-NTD	glyco	.	.	surface
glycoprotein_TQDLFLPFFSNVTWF	19	surface glycoprotein_LPFFSNVTW	0.08	.
.	nCoV-2019_71_RIGHT	.	.	.	.	.

1	21745	21745	T	C	exonic	S	.	synonymous SNV
S:cds-YP_009724390.1:exon1:c.T183C:p.N61N	-3.3	-0.853921	0	1	T
disulf_bond	BetaCoV_S1-NTD	glyco	.	.	surface
glycoprotein_TQDLFLPFFSNVTWF	19	surface glycoprotein_LPFFSNVTW	0.08	.
.	.	.	.	.	.	.
1	21761	21761	G	T	exonic	S	.	nonsynonymous SNV
S:cds-YP_009724390.1:exon1:c.G199T:p.A67S	-3.3	-3.28113	0	0.81	T
disulf_bond	BetaCoV_S1-NTD	.	.	Surface glycoprotein_FHAIHVSGTNG
surface glycoprotein_LPFFSNVTWFHAIHV	16	.	.	.	.	.	.
.	.	.	.
1	21767	21767	C	T	exonic	S	.	nonsynonymous SNV
S:cds-YP_009724390.1:exon1:c.C205T:p.H69Y	-2.31	0.040315	0	1	T
disulf_bond	BetaCoV_S1-NTD	.	.	Surface glycoprotein_FHAIHVSGTNG
surface glycoprotein_LPFFSNVTWFHAIHV	16	.	.	.	.	.	.
.	.	.	.
1	21770	21770	G	T	exonic	S	.	nonsynonymous SNV
S:cds-YP_009724390.1:exon1:c.G208T:p.V70F	-3.18	-0.726173	0	0.08	T
disulf_bond	BetaCoV_S1-NTD	.	.	Surface glycoprotein_FHAIHVSGTNG
surface glycoprotein_LPFFSNVTWFHAIHV	16	.	.	.	.	.	.
.	.	.	.
1	21774	21774	C	T	exonic	S	.	nonsynonymous SNV
S:cds-YP_009724390.1:exon1:c.C212T:p.S71F	-3.3	0.168063	0	0.03	D
disulf_bond	BetaCoV_S1-NTD	.	.	Surface glycoprotein_FHAIHVSGTNG
.	.	.	.	.	.	.	.	.	.	.	.

1	21786	21786	G	T	exonic	S	.	nonsynonymous SNV
S:cds-YP_009724390.1:exon1:c.G224T:p.G75V	-2.66	-0.726173	0	0.45	T
disulf_bond	BetaCoV_S1-NTD	.	.	Surface glycoprotein_FHAIHVSGTNG
.	.	.	.	.	.	.	.	.	.	.	.

1	21792	21792	A	T	exonic	S	.	nonsynonymous SNV
S:cds-YP_009724390.1:exon1:c.A230T:p.K77M	0.154	-3.28113	0	0.29	T
disulf_bond	BetaCoV_S1-NTD	.	.	.	.	.	.	.	.
.	.	.	.	.	.	.
1	21802	21802	T	C	exonic	S	.	synonymous SNV
S:cds-YP_009724390.1:exon1:c.T240C:p.D80D	-3.25	-1.74816	0	0.73	T
disulf_bond	BetaCoV_S1-NTD	.	.	.	.	.	surface
glycoprotein_RFDNPVLPF	-1	.	.	.	.	.	.	.	.

1 21846 21846 C T exonic S . nonsynonymous SNV
S:cds-YP_009724390.1:exon1:c.C284T:p.T95I -0.152 0.679055 0 0.07 T
disulf_bond BetaCoV_S1-NTD . . . . . surface
glycoprotein_GVYFASTEK -1 . . . .

1 21859 21859 C T exonic S . synonymous SNV
S:cds-YP_009724390.1:exon1:c.C297T:p.N99N -2.23 -0.342929 0.0551181 0.35
T disulf_bond BetaCoV_S1-NTD . . . . . surface
glycoprotein_TEKSNIIRGW -1 . . . .

1 21886 21886 T C exonic S . synonymous SNV
S:cds-YP_009724390.1:exon1:c.T324C:p.T108T -3.3 -7.62457 0 0.84 T
disulf_bond BetaCoV_S1-NTD . . Surface
glycoprotein_TLDSKTQSLLIVNNATNV . . surface glycoprotein_TLDSKTQSL
0.14 . . . .
1 21936 21936 A C exonic S . nonsynonymous SNV
S:cds-YP_009724390.1:exon1:c.A374C:p.N125T -0.669 0.040315 0 0.22 T
disulf_bond BetaCoV_S1-NTD . . Surface
glycoprotein_TLDSKTQSLLIVNNATNV surface glycoprotein_SLLIVNNATNVVIKV 14
. . . .

1 21952 21952 C T exonic S . synonymous SNV
S:cds-YP_009724390.1:exon1:c.C390T:p.V130V -3.3 -2.51465 0 1 T
disulf_bond BetaCoV_S1-NTD . . . surface
glycoprotein_SLLIVNNATNVVIKV 14 . . . .
. . .
1 21974 21974 G C exonic S . nonsynonymous SNV
S:cds-YP_009724390.1:exon1:c.G412C:p.D138H -2.36 -0.215181 0 0.38 T
disulf_bond BetaCoV_S1-NTD . . . .
. nCoV-2019_73_LEFT . . . . .
1 22020 22020 T C exonic S . nonsynonymous SNV
S:cds-YP_009724390.1:exon1:c.T458C:p.M153T -0.866 0.168063 0.0708661 0.88
T disulf_bond BetaCoV_S1-NTD . . Surface
glycoprotein_HKNNKSWMESEFRVYSSANNCTF . . . . . .
nCoV-2019_72_RIGHT . . . . .
1 22021 22021 G T exonic S . nonsynonymous SNV
S:cds-YP_009724390.1:exon1:c.G459T:p.M153I -0.866 0.168063 0.0787402 0.49
T disulf_bond BetaCoV_S1-NTD . . Surface
glycoprotein_HKNNKSWMESEFRVYSSANNCTF . . . . . .
nCoV-2019_72_RIGHT . . . . .
1 22024 22024 A G exonic S . synonymous SNV
S:cds-YP_009724390.1:exon1:c.A462G:p.E154E -0.308 0.168063 0.0866142 1

T disulf_bond BetaCoV_S1-NTD . . Surface
glycoprotein_HKNNKSWMESEFRVYSSANNCTF . . . . . .
nCoV-2019_72_RIGHT . . . . .
1 22029 22029 A T exonic S . nonsynonymous SNV
S:cds-YP_009724390.1:exon1:c.A467T:p.E156V 0.154 1.0623 0.0314961 0.23 T
disulf_bond BetaCoV_S1-NTD . . Surface
glycoprotein_HKNNKSWMESEFRVYSSANNCTF surface glycoprotein_EFRVYSSANNCTFEY
18 . . . . nCoV-2019_72_RIGHT . . . .
.
1 22030 22030 G T exonic S . nonsynonymous SNV
S:cds-YP_009724390.1:exon1:c.G468T:p.E156D -0.308 0.934551 0.023622 0.95
T disulf_bond BetaCoV_S1-NTD . . Surface
glycoprotein_HKNNKSWMESEFRVYSSANNCTF surface glycoprotein_EFRVYSSANNCTFEY
18 . . . . nCoV-2019_72_RIGHT . . . .
.
1 22088 22088 C T exonic S . nonsynonymous SNV
S:cds-YP_009724390.1:exon1:c.C526T:p.L176F -3.3 -1.36491 0 0.7 T
. BetaCoV_S1-NTD . . . . . surface
glycoprotein_FEYVSQPFL 0.04 . . . .

1 22093 22093 G C exonic S . nonsynonymous SNV
S:cds-YP_009724390.1:exon1:c.G531C:p.M177I -3.3 -6.85808 0 0.28 T
. BetaCoV_S1-NTD . . . . . surface
glycoprotein_EYVSQPFLM -1 . . . .

1 22225 22225 G T exonic S . synonymous SNV
S:cds-YP_009724390.1:exon1:c.G663T:p.S221S -3.3 -10.6905 0 1 T
. BetaCoV_S1-NTD . . . . . surface
glycoprotein_LPQGFSAL 0.1 . . . .

1 22227 22227 C T exonic S . nonsynonymous SNV
S:cds-YP_009724390.1:exon1:c.C665T:p.A222V -3.3 -1.23717 0 1 T
. BetaCoV_S1-NTD . . . . . surface
glycoprotein_LPQGFSAL 0.1 . . . .

1 22244 22244 G C exonic S . nonsynonymous SNV
S:cds-YP_009724390.1:exon1:c.G682C:p.D228H 0.509 0.551307 0 0.06 T
. BetaCoV_S1-NTD . . . .
. . . . . .
1 22249 22249 G T exonic S . nonsynonymous SNV
S:cds-YP_009724390.1:exon1:c.G687T:p.L229F -3.3 -5.3251 0 0.03 D
. BetaCoV_S1-NTD . . . . . surface
glycoprotein_LPIGINITRF 0.06 . . . .

1 22273 22273 G A exonic S . synonymous SNV
S:cds-YP_009724390.1:exon1:c.G711A:p.R237R -3.3 -0.726173 0 0.82 T
. BetaCoV_S1-NTD . . . surface glycoprotein_IGINITRFQTLLALH
18 surface glycoprotein_LPIGINITRF 0.06 . . nCoV-2019_74_LEFT .
. . . .
1 22281 22281 C T exonic S . nonsynonymous SNV
S:cds-YP_009724390.1:exon1:c.C719T:p.T240I -3.06 -2.00365 0 0.38 T
. BetaCoV_S1-NTD . . . surface glycoprotein_IGINITRFQTLLALH
18 surface glycoprotein_INITRFQTL 0.06 . . nCoV-2019_74_LEFT .
. . . .
1 22323 22323 C T exonic S . nonsynonymous SNV
S:cds-YP_009724390.1:exon1:c.C761T:p.S254F -3.13 1.3178 0 0.1 T .
BetaCoV_S1-NTD . . Surface glycoprotein_PGDSSSGWTAGA . .
. . . .

1 22344 22344 G T exonic S . nonsynonymous SNV
S:cds-YP_009724390.1:exon1:c.G782T:p.G261V -0.308 -0.0874331 0 0.62 T
. BetaCoV_S1-NTD . . Surface glycoprotein_PGDSSSGWTAGA .
. surface glycoprotein_WTAGAAAYY 0.03 . . nCoV-2019_73_RIGHT
SARS-CoV-2_IBS_S2_F Korea . . .
1 22348 22348 T A exonic S . synonymous SNV
S:cds-YP_009724390.1:exon1:c.T786A:p.A262A -0.308 1.19005 0.629921 0.46
T . BetaCoV_S1-NTD . . Surface glycoprotein_PGDSSSGWTAGA
. . surface glycoprotein_WTAGAAAYY 0.03 . . .
SARS-CoV-2_IBS_S2_F Korea . . .
1 22361 22361 G T exonic S . nonsynonymous SNV
S:cds-YP_009724390.1:exon1:c.G799T:p.V267L 0.747 2.72302 0.15748 0.01
D . BetaCoV_S1-NTD . . . . . surface
glycoprotein_YYVGYLQPRTF -1 . . . .
.
1 22363 22363 G T exonic S . synonymous SNV
S:cds-YP_009724390.1:exon1:c.G801T:p.V267V -3.3 -5.06961 0 1 T
. BetaCoV_S1-NTD . . . . . surface
glycoprotein_YYVGYLQPRTF -1 . . . .
.
1 22388 22388 C T exonic S . synonymous SNV
S:cds-YP_009724390.1:exon1:c.C826T:p.L276L -0.778 1.19005 0.110236 1
T . BetaCoV_S1-NTD . . . . . surface
glycoprotein_YLQPRTFL 0.05 . . . .

1 22408 22408 T C exonic S . synonymous SNV
S:cds-YP_009724390.1:exon1:c.T846C:p.N282N 0.446 0.934551 0.992126 1

T	.	BetaCoV_S1-NTD	glyco	.	Surface glycoprotein_NENGTITDA	.
.	.	.	.	.	.	.	.	.	.	.

1	22444	22444	C	T	exonic	S	.	synonymous SNV
S:cds-YP_009724390.1:exon1:c.C882T:p.D294D	-3.3	-1.23717	0.0314961	1
T	disulf_bond	BetaCoV_S1-NTD	.	.	.	.	.	.	.
.	.	.	SARS-CoV-2_IBS_S2_R	Korea	.	.	.

1	22468	22468	G	T	exonic	S	.	synonymous SNV
S:cds-YP_009724390.1:exon1:c.G906T:p.T302T	-3.3	-8.13556	0	1	T
.	BetaCoV_S1-NTD	.	.	.	.	.	surface
glycoprotein_SETKCTLKSF	-1	.	.	.	.	.	.	.	.

1	22480	22480	C	T	exonic	S	.	synonymous SNV
S:cds-YP_009724390.1:exon1:c.C918T:p.F306F	-2.19	0.040315	0	1	T
.	.	.	.	.	surface glycoprotein_FTVEKGIYQTSNFRV	18
surface glycoprotein_SETKCTLKSF	-1	.	.	.	.	.	.	.
.
1	22482	22482	C	T	exonic	S	.	nonsynonymous SNV
S:cds-YP_009724390.1:exon1:c.C920T:p.T307I	-0.555	0.168063	0	0.15	T
.	.	.	.	.	surface glycoprotein_FTVEKGIYQTSNFRV	18
surface glycoprotein_TLKSFTVEK	-1	.	.	.	.	.	.	.
.
1	22529	22529	A	G	exonic	S	.	nonsynonymous SNV
S:cds-YP_009724390.1:exon1:c.A967G:p.T323A	-3.3	-3.11017	0	0.29	T
.	.	.	.	.	surface glycoprotein_SNFRVQPTESIVRFP	16
surface glycoprotein_QPTESIVRF	0.02	.	.	nCoV-2019_75_LEFT	.	.
.	.	.
1	22550	22550	C	T	exonic	S	.	nonsynonymous SNV
S:cds-YP_009724390.1:exon1:c.C988T:p.P330S	1.65	3.2572	0.76378	0.04	D
.	.	.	.	.	surface glycoprotein_SNFRVQPTESIVRFP	16
surface glycoprotein_RFPNITNLCPF	0.07	Spike glycoprotein
VRFPNITNLCPFGEVFN	.	.	.	.	.	.

1	22591	22591	C	T	exonic	S	.	synonymous SNV
S:cds-YP_009724390.1:exon1:c.C1029T:p.N343N	0.475	1.00989	1	1	T
disulf_bond	BetaCoV_S1-CTD	glyco	.	.	surface
glycoprotein_VFNATRFASVYAWNR	9.3	surface glycoprotein_GEVFNATRF	-1	.
.	.	.	.	.	.	.
1	22624	22624	C	T	exonic	S	.	synonymous SNV
S:cds-YP_009724390.1:exon1:c.C1062T:p.N354N	-3.3	-3.85928	0	0.1	T
disulf_bond	BetaCoV_S1-CTD	.	.	.	surface
glycoprotein_VFNATRFASVYAWNR	9.3	.	.	.	.	.	.	.

. . .
1 22681 22681 A G exonic S . synonymous SNV
S:cds-YP_009724390.1:exon1:c.A1119G:p.S373S -3.3 -3.23502 0 0.78 T
. BetaCoV_S1-CTD . . Surface
glycoprotein_KRISNCVADYSVLYNSASFST. . surface glycoprotein_LYNSASFSTF
-1 . . . .
1 22689 22689 C T exonic S . nonsynonymous SNV
S:cds-YP_009724390.1:exon1:c.C1127T:p.T376I 1.65 3.2572 0.984252 0.02 D
. BetaCoV_S1-CTD . . Surface
glycoprotein_KRISNCVADYSVLYNSASFST. . surface glycoprotein_LYNSASFSTF
-1 . . . .
1 22691 22691 T C exonic S . nonsynonymous SNV
S:cds-YP_009724390.1:exon1:c.T1129C:p.F377L 1.65 2.13354 0.952756 0.35
T . BetaCoV_S1-CTD . . . . . surface
glycoprotein_LYNSASFSTF -1 . . . .

1 22708 22708 G T exonic S . synonymous SNV
S:cds-YP_009724390.1:exon1:c.G1146T:p.V382V -3.3 -1.36227 0.669291 0.7
T disulf_bond BetaCoV_S1-CTD . . . .
. . . .
1 22713 22713 C T exonic S . nonsynonymous SNV
S:cds-YP_009724390.1:exon1:c.C1151T:p.P384L 1.65 3.2572 0.480315 0.15 T
disulf_bond BetaCoV_S1-CTD . . . .
. . S Forward China . . .
1 22747 22747 C T exonic S . synonymous SNV
S:cds-YP_009724390.1:exon1:c.C1185T:p.V395V -2.83 -0.862866 0.692913 1
T disulf_bond BetaCoV_S1-CTD . . . .
. . . .
1 22777 22777 T C exonic S . synonymous SNV
S:cds-YP_009724390.1:exon1:c.T1215C:p.D405D -3.3 -9.85209 0 0.72 T
disulf_bond BetaCoV_S1-CTD . . . .
. . . .
1 22801 22801 G T exonic S . synonymous SNV
S:cds-YP_009724390.1:exon1:c.G1239T:p.G413G -3.3 -3.23502 0 1 T
disulf_bond BetaCoV_S1-CTD . . . . . surface
glycoprotein_RQIAPGQTGK 0.03 . . nCoV-2019_76_LEFT. . .
. .
1 22820 22820 G T exonic S . nonsynonymous SNV
S:cds-YP_009724390.1:exon1:c.G1258T:p.D420Y 1.65 4.256 0.992126 0.05 D
disulf_bond BetaCoV_S1-CTD . . . . . surface
glycoprotein_KIADYNYKL 0.04 . . nCoV-2019_76_LEFT_alt3 . .
. . .
1 22858 22858 C T exonic S . synonymous SNV

S:cds-YP_009724390.1:exon1:c.C1296T:p.C432C -2.18 -0.238614 0.976378 1
T disulf_bond BetaCoV_S1-CTD . . . .
. . . S Reverse China . . .
1 22882 22882 T G exonic S . nonsynonymous SNV
S:cds-YP_009724390.1:exon1:c.T1320G:p.N440K -3.3 0.135937 0 0.73 T
disulf_bond BetaCoV_S1-CTD . . Surface glycoprotein_IAWNSNNLDSK
. . . . . . nCoV-2019_75_RIGHT . . .
. .
1 22899 22899 G T exonic S . nonsynonymous SNV
S:cds-YP_009724390.1:exon1:c.G1337T:p.G446V . 0.385638 0 0.33 T
disulf_bond BetaCoV_S1-CTD . . . .
. nCoV-2019_75_RIGHT . . . . .

1 22912 22912 T G exonic S . nonsynonymous SNV
S:cds-YP_009724390.1:exon1:c.T1350G:p.N450K -3.3 0.260787 0.244094 0.09
T disulf_bond BetaCoV_S1-CTD . . . surface
glycoprotein_YLYRLFRKSNLKPFE 9.2 surface glycoprotein_NYNYLYRLF -1 .
. . . .
1 22918 22918 G T exonic S . synonymous SNV
S:cds-YP_009724390.1:exon1:c.G1356T:p.L452L -3.3 -0.363465 0.0314961 0.65
T disulf_bond BetaCoV_S1-CTD . . . surface
glycoprotein_YLYRLFRKSNLKPFE 9.2 surface glycoprotein_NYNYLYRLF -1 .
. . . .
1 23071 23071 T C exonic S . synonymous SNV
S:cds-YP_009724390.1:exon1:c.T1509C:p.V503V -3.3 -3.98413 0 0.93 T
disulf_bond BetaCoV_S1-CTD . . . .
. . . .
1 23248 23248 C T exonic S . synonymous SNV
S:cds-YP_009724390.1:exon1:c.C1686T:p.F562F -3.3 -0.363465 0.811024 0.57
T disulf_bond . . . .
. . . .
1 23277 23277 C T exonic S . nonsynonymous SNV
S:cds-YP_009724390.1:exon1:c.C1715T:p.T572I 0.475 1.00989 0 0.57 T
disulf_bond . . . .
. . . . . .
1 23311 23311 G T exonic S . nonsynonymous SNV
S:cds-YP_009724390.1:exon1:c.G1749T:p.E583D -3.3 -3.60957 0 0.33 T
disulf_bond . . . .
. . . . . .
1 23350 23350 T C exonic S . synonymous SNV
S:cds-YP_009724390.1:exon1:c.T1788C:p.S596S 1.65 2.13354 1 1 T
. . . . Surface glycoprotein_FGGVSVITPGTNTSNQVAVLYQDVNCTEV
. . . .

1 23371 23371 T C exonic S . synonymous SNV
S:cds-YP_009724390.1:exon1:c.T1809C:p.N603N -3.3 -0.738016 0.00787402 0.63
T . . glyco . Surface
glycoprotein_FGGVSVITPGTNTSNQVAVLYQDVNCTEV . . surface
glycoprotein_TSNQVAVLY 0.04 . . . .

1 23381 23381 C G exonic S . nonsynonymous SNV
S:cds-YP_009724390.1:exon1:c.C1819G:p.Q607E -0.394 1.38444 0.015748 1
T . . . . Surface
glycoprotein_FGGVSVITPGTNTSNQVAVLYQDVNCTEV . . surface
glycoprotein_TSNQVAVLY 0.04 . . . .

1 23403 23403 A G exonic S . nonsynonymous SNV
S:cds-YP_009724390.1:exon1:c.A1841G:p.D614G 1.65 2.25839 1 0.3 T
. . . . Surface glycoprotein_FGGVSVITPGTNTSNQVAVLYQDVNCTEV
. . . .

1 23416 23416 A T exonic S . synonymous SNV
S:cds-YP_009724390.1:exon1:c.A1854T:p.T618T -3.3 -1.11257 0.952756 0.55
T disulf_bond . . . Surface
glycoprotein_FGGVSVITPGTNTSNQVAVLYQDVNCTEV . . . . .
. . . .
1 23426 23426 G T exonic S . nonsynonymous SNV
S:cds-YP_009724390.1:exon1:c.G1864T:p.V622F -3.22 -0.613165 0 0.73 T
disulf_bond . . . .
. . . . .
1 23525 23525 C T exonic S . nonsynonymous SNV
S:cds-YP_009724390.1:exon1:c.C1963T:p.H655Y 0.68 1.63414 0.976378 1
T . . . . Surface glycoprotein_GAEHVNNSYE . .
surface glycoprotein_AEHVNNSY 0.11 . . . .
.
1 23530 23530 C T exonic S . synonymous SNV
S:cds-YP_009724390.1:exon1:c.C1968T:p.V656V -3.3 -0.488315 0.00787402 1
T . . glyco . Surface glycoprotein_GAEHVNNSYE . .
surface glycoprotein_AEHVNNSY 0.11 . . . .
.
1 23533 23533 C T exonic S . synonymous SNV
S:cds-YP_009724390.1:exon1:c.C1971T:p.N657N -3.3 -2.36107 0 1 T
. . glyco . Surface glycoprotein_GAEHVNNSYE . .
surface glycoprotein_AEHVNNSY 0.11 . . . .
.
1 23540 23540 T C exonic S . nonsynonymous SNV

S:cds-YP_009724390.1:exon1:c.T1978C:p.Y660H 1.65 2.13354 0.905512 0.52
T . . . . Surface glycoprotein_GAEHVNNSYE . .
surface glycoprotein_AEHVNNSY 0.11 . . . .
.
1 23580 23580 G C exonic S . nonsynonymous SNV
S:cds-YP_009724390.1:exon1:c.G2018C:p.S673T 0.646 2.28422 0.968504 0.39
T . . . . Surface
glycoprotein_SYQTQTNSPRRARSVASQSIIAYTMSLGAENSVAYSNNSIA . .
surface glycoprotein_IPIGAGICASY 0.06 Spike glycoprotein DIPIGAGICASYHTVSLL
. . . . .
1 23590 23590 T C exonic S . synonymous SNV
S:cds-YP_009724390.1:exon1:c.T2028C:p.T676T -0.956 0.0933543 0.795276 1
T . . . . Surface
glycoprotein_SYQTQTNSPRRARSVASQSIIAYTMSLGAENSVAYSNNSIA . .
. . Spike glycoprotein DIPIGAGICASYHTVSLL . . . .
. .
1 23593 23593 G T exonic S . nonsynonymous SNV
S:cds-YP_009724390.1:exon1:c.G2031T:p.Q677H -3.09 -1.33071 0 0.11 T
. . . . Surface
glycoprotein_SYQTQTNSPRRARSVASQSIIAYTMSLGAENSVAYSNNSIA . .
. . Spike glycoprotein DIPIGAGICASYHTVSLL . . . .
. .
1 23608 23608 G A exonic S . synonymous SNV
S:cds-YP_009724390.1:exon1:c.G2046A:p.R682R . -0.016189 0.0708661 1
T . . . . Surface
glycoprotein_SYQTQTNSPRRARSVASQSIIAYTMSLGAENSVAYSNNSIA . .
surface glycoprotein_SPRRARSVA 0.08 . . . .
.
1 23608 23608 G T exonic S . synonymous SNV
S:cds-YP_009724390.1:exon1:c.G2046T:p.R682R . -0.016189 0.0708661 1
T . . . . Surface
glycoprotein_SYQTQTNSPRRARSVASQSIIAYTMSLGAENSVAYSNNSIA . .
surface glycoprotein_SPRRARSVA 0.08 . . . .
.
1 23643 23643 C T exonic S . nonsynonymous SNV
S:cds-YP_009724390.1:exon1:c.C2081T:p.A694V 1.65 3.27011 0.992126 0.55
T . . . . Surface
glycoprotein_SYQTQTNSPRRARSVASQSIIAYTMSLGAENSVAYSNNSIA . .
surface glycoprotein_VASQSIIAY 0.02 . . . .
.
1 23678 23678 G C exonic S . nonsynonymous SNV
S:cds-YP_009724390.1:exon1:c.G2116C:p.A706P 1.65 4.256 0.929134 0.33 T
. . . . Surface

glycoprotein_SYQTQTNSPRRARSVASQSIIAYTMSLGAENSVAYSNNSIA . .
surface glycoprotein_LGAENSVAY 0.07 . . . .
.
1 23707 23707 C T exonic S . synonymous SNV
S:cds-YP_009724390.1:exon1:c.C2145T:p.P715P -2.46 -0.454362 0.96063 1
T . . . . surface
glycoprotein_AYSNNSIAIPTNF 0.09 . . . .
.
1 23780 23780 A T exonic S . nonsynonymous SNV
S:cds-YP_009724390.1:exon1:c.A2218T:p.M740L 1.65 2.28422 0.937008 0.4
T disulf_bond . . . .
. . . . . .
1 23876 23876 G A exonic S . nonsynonymous SNV
S:cds-YP_009724390.1:exon1:c.G2314A:p.V772I -3.3 -3.30249 0 0.19 T
. . . . Surface glycoprotein_AVEQDKNTQE . . .
. . . .
1 23923 23923 A G exonic S . synonymous SNV
S:cds-YP_009724390.1:exon1:c.A2361G:p.Q787Q -3.3 -0.125732 0.992126 0.19
T . . . . surface
glycoprotein_QEVFAQVKQIY -1 . . . .
.
1 23929 23929 C T exonic S . synonymous SNV
S:cds-YP_009724390.1:exon1:c.C2367T:p.Y789Y -3.3 -1.11162 0.496063 1
T . . . . surface
glycoprotein_QEVFAQVKQIY -1 . . . .
.
1 23948 23948 G C exonic S . nonsynonymous SNV
S:cds-YP_009724390.1:exon1:c.G2386C:p.D796H 0.741 2.72239 0.976378 0.12
T . . . . surface glycoprotein_IYKTPPIKDF
-1 . . . .
1 24000 24000 G T exonic S . nonsynonymous SNV
S:cds-YP_009724390.1:exon1:c.G2438T:p.S813I 0.746 0.969701 0.338583 0.01
D . . . .
. . . . .
1 24095 24095 G T exonic S . nonsynonymous SNV
S:cds-YP_009724390.1:exon1:c.G2533T:p.A845S -0.735 0.421984 0.850394 0.62
T . . . .
nCoV-2019_80_LEFT . . . . .
1 24096 24096 C T exonic S . nonsynonymous SNV
S:cds-YP_009724390.1:exon1:c.C2534T:p.A845V -3.3 -0.782992 0.716535 0.07
T . . . .
nCoV-2019_80_LEFT . . . . .
1 24116 24116 G T exonic S . nonsynonymous SNV

S:cds-YP_009724390.1:exon1:c.G2554T:p.A852S 1.65 4.256 1 0.04 D .
. . . .
. . .
1 24130 24130 C T exonic S . synonymous SNV
S:cds-YP_009724390.1:exon1:c.C2568T:p.N856N -3.3 -0.235276 0.992126 1
T . . . .
. . . . .
1 24230 24230 G A exonic S . nonsynonymous SNV
S:cds-YP_009724390.1:exon1:c.G2668A:p.A890T 1.65 4.256 1 0.1 T .
. . . .
. . .
1 24319 24319 C T exonic S . synonymous SNV
S:cds-YP_009724390.1:exon1:c.C2757T:p.N919N -3.3 -1.33071 0.590551 1
T . . . . Surface
glycoprotein_VLYENQKLIANQFNSAIGKIQDSLSSTASALG surface
glycoprotein_VTQNVLYENQKLIAN 17 . . . .
. . .
1 24322 24322 A G exonic S . synonymous SNV
S:cds-YP_009724390.1:exon1:c.A2760G:p.Q920Q -3.25 -0.125732 0.96063 1
T . . . . Surface
glycoprotein_VLYENQKLIANQFNSAIGKIQDSLSSTASALG surface
glycoprotein_VTQNVLYENQKLIAN 17 . . . .
. . .
1 24368 24368 G T exonic S . nonsynonymous SNV
S:cds-YP_009724390.1:exon1:c.G2806T:p.D936Y 1.65 4.256 1 0.01 D .
. . . Surface glycoprotein_VLYENQKLIANQFNSAIGKIQDSLSSTASALG
. . . .

1 24370 24370 C T exonic S . synonymous SNV
S:cds-YP_009724390.1:exon1:c.C2808T:p.D936D -3.3 -0.125732 0.992126 0.24
T . . . . Surface
glycoprotein_VLYENQKLIANQFNSAIGKIQDSLSSTASALG . . . .
. . . .
1 24378 24378 C T exonic S . nonsynonymous SNV
S:cds-YP_009724390.1:exon1:c.C2816T:p.S939F 0.74 1.7365 0.0551181 0.02 D
. . . . Surface
glycoprotein_VLYENQKLIANQFNSAIGKIQDSLSSTASALG . . surface
glycoprotein_SSTASALGK 0.09 . . . .
hypermutable low-fitness site
1 24386 24386 G T exonic S . nonsynonymous SNV
S:cds-YP_009724390.1:exon1:c.G2824T:p.A942S -2.28 0.312441 0.755906 0.53
T . . . . Surface
glycoprotein_VLYENQKLIANQFNSAIGKIQDSLSSTASALG . . surface

glycoprotein_SSTASALGK 0.09 . . . .

1 24406 24406 T C exonic S . synonymous SNV
S:cds-YP_009724390.1:exon1:c.T2844C:p.L948L -1.64 0.860157 1 0.36 T
. . . . Surface glycoprotein_LQDVVNQNAQALNTLVKQLSSNFGAISS
. . . . . . nCoV-2019_81_LEFT . . . .
.
1 24406 24406 T G exonic S . synonymous SNV
S:cds-YP_009724390.1:exon1:c.T2844G:p.L948L -1.64 0.860157 1 0.36 T
. . . . Surface glycoprotein_LQDVVNQNAQALNTLVKQLSSNFGAISS
. . . . . . nCoV-2019_81_LEFT . . . .
.
1 24529 24529 T C exonic S . synonymous SNV
S:cds-YP_009724390.1:exon1:c.T2967C:p.A989A -3.3 -3.0834 0.181102 1
T . . . . surface glycoprotein_RLDKVEAEV
0.08 . . . .
1 24621 24621 C T exonic S . nonsynonymous SNV
S:cds-YP_009724390.1:exon1:c.C3059T:p.A1020V 1.65 3.2969 1 0.79 T .
. . . Surface glycoprotein_RASANLAATKMSECVLGQ surface
glycoprotein_AEIRASANLAATKMS 13 surface glycoprotein_AEIRASANL -1 Spike
glycoprotein RASANLAATKMSECVLG, AATKMSECVLGQSKRVD . . .
. . .
1 24652 24652 A T exonic S . synonymous SNV
S:cds-YP_009724390.1:exon1:c.A3090T:p.S1030S -3.3 -0.326378 0.992126 0.35
T . . . . Surface glycoprotein_RASANLAATKMSECVLGQ
surface glycoprotein_AEIRASANLAATKMS 13 . . Spike glycoprotein
RASANLAATKMSECVLG, AATKMSECVLGQSKRVD . . . . .
.
1 24718 24718 C T exonic S . synonymous SNV
S:cds-YP_009724390.1:exon1:c.C3156T:p.F1052F -3.3 -0.326378 0.661417 0.52
T . . . . surface glycoprotein_HLMSFPQSA
0.16 . . nCoV-2019_82_LEFT . . . . .

1 24721 24721 T C exonic S . synonymous SNV
S:cds-YP_009724390.1:exon1:c.T3159C:p.P1053P -3.3 -2.67085 0.220472 0.32
T . . . . surface glycoprotein_HLMSFPQSA
0.16 . . nCoV-2019_82_LEFT . . . . .

1 24751 24751 G T exonic S . nonsynonymous SNV
S:cds-YP_009724390.1:exon1:c.G3189T:p.L1063F -2.03 0.313024 0.92126 0.36
T . . . . . surface glycoprotein_VFLHVTYVPAQEKNF 20
surface glycoprotein_APHGVVFL 0.12 Spike glycoprotein VVFLHVTYV . .
. . . .

1	24770	24770	G	T	exonic	S	.	nonsynonymous SNV
S:cds-YP_009724390.1:exon1:c.G3208T:p.A1070S	-0.883	0.419591	0.787402	0.34
T	.	.	.	.	.	surface glycoprotein_VFLHVTYVPAQEKNF	20
surface glycoprotein_VTYVPAQEK	-1	.	.	nCoV-2019_81_RIGHT	.
.	.	.	.
1	24793	24793	T	C	exonic	S	.	synonymous SNV
S:cds-YP_009724390.1:exon1:c.T3231C:p.T1077T	-3.3	-4.48249	0.0551181	0.9
T	.	.	.	.	.	.	.	.	.	.	.	.
.	.	.	.	.
1	24794	24794	G	T	exonic	S	.	nonsynonymous SNV
S:cds-YP_009724390.1:exon1:c.G3232T:p.A1078S	1.65	4.256	0.850394	0.99	T
.	.	.	.	.	.	.	.	.	.	.	.	.
.	.	.	.
1	24815	24815	G	C	exonic	S	.	nonsynonymous SNV
S:cds-YP_009724390.1:exon1:c.G3253C:p.G1085R	1.65	4.256	1	0.03	D
disulf_bond	.	.	.	.	.	.	.	.	.	.	.
.	.	.	.	.
1	24817	24817	A	G	exonic	S	.	synonymous SNV
S:cds-YP_009724390.1:exon1:c.A3255G:p.G1085G	-1.76	0.0998898	0.992126
1	T	disulf_bond	.	.	.	.	.	.	.	.	.
.	.	.	.	.	.	.
1	24822	24822	C	T	exonic	S	.	nonsynonymous SNV
S:cds-YP_009724390.1:exon1:c.C3260T:p.A1087V	1.65	3.2969	1	0.25	T
disulf_bond	.	.	.	.	.	.	.	.	.	.	.
.	.	.	.	.
1	24833	24833	C	T	exonic	S	.	nonsynonymous SNV
S:cds-YP_009724390.1:exon1:c.C3271T:p.R1091C	0.737	1.80496	0.992126
0.02	D	disulf_bond	.	.	.	.	.	.	surface
glycoprotein_REGVFVSNGTHW	-1	.	.	.	.	.	.	.
.
1	24836	24836	G	T	exonic	S	.	stopgain
S:cds-YP_009724390.1:exon1:c.G3274T:p.E1092X	1.65	4.256	1	.	.
disulf_bond	.	.	.	.	.	.	surface
glycoprotein_REGVFVSNGTHW	-1	.	.	.	.	.	.	.
.
1	24844	24844	C	T	exonic	S	.	synonymous SNV
S:cds-YP_009724390.1:exon1:c.C3282T:p.V1094V	-3.3	-1.07235	0.84252	0.6
T	disulf_bond	.	.	.	.	.	.	surface
glycoprotein_REGVFVSNGTHW	-1	.	.	.	.	.	.	.
.
1	24872	24872	G	T	exonic	S	.	nonsynonymous SNV
S:cds-YP_009724390.1:exon1:c.G3310T:p.V1104L	0.509	0.526157	0.165354	0.3
T	disulf_bond	.	.	.	.	.	.	surface

glycoprotein_GTHWFVTQR 0.09 . . . .

1 24903 24903 T C exonic S . nonsynonymous SNV
S:cds-YP_009724390.1:exon1:c.T3341C:p.I1114T -2.14 -0.00667717 0.0708661 0.59
T disulf_bond . . . .
. . . . . .
1 24956 24956 A G exonic S . nonsynonymous SNV
S:cds-YP_009724390.1:exon1:c.A3394G:p.I1132V 1.65 2.23123 1 0.5 T
. . . .
. . . .
1 24959 24959 G T exonic S . nonsynonymous SNV
S:cds-YP_009724390.1:exon1:c.G3397T:p.V1133F -0.79 0.632724 0.992126 0.03
D . . . .
. . . . .
1 25096 25096 C T exonic S . synonymous SNV
S:cds-YP_009724390.1:exon1:c.C3534T:p.N1178N -3.3 -3.94965 0.0314961
0.53 T . . . . Surface glycoprotein_GINASVVNIQ . .
. . Spike glycoprotein KNHTSPDVDLGDISGIN, DLGDISGINASVVNIQK,
EIDRLNEVAKNLNESLIDLQELGKYEQY . . . . . .

1 25135 25135 G T exonic S . nonsynonymous SNV
S:cds-YP_009724390.1:exon1:c.G3573T:p.K1191N 1.65 0.845858 0.992126
0.15 T . . . . Surface glycoprotein_EVAKNLNESL . .
. . Spike glycoprotein KNHTSPDVDLGDISGIN, DLGDISGINASVVNIQK,
EIDRLNEVAKNLNESLIDLQELGKYEQY . . . . . .

1 25157 25157 G T exonic S . nonsynonymous SNV
S:cds-YP_009724390.1:exon1:c.G3595T:p.D1199Y 1.65 4.256 1 0 D
. . . . Spike glycoprotein
KNHTSPDVDLGDISGIN, DLGDISGINASVVNIQK, EIDRLNEVAKNLNESLIDLQELGKYEQY
. . . . . .
1 25169 25169 C T exonic S . nonsynonymous SNV
S:cds-YP_009724390.1:exon1:c.C3607T:p.L1203F -0.194 -0.113244 0.944882 0.01
D . . . . surface
glycoprotein_QELGKYEQYIKW -1 Spike glycoprotein KNHTSPDVDLGDISGIN,
DLGDISGINASVVNIQK, EIDRLNEVAKNLNESLIDLQELGKYEQY . . . .
. .
1 25207 25207 C T exonic S . synonymous SNV
S:cds-YP_009724390.1:exon1:c.C3645T:p.Y1215Y -3.3 -2.45772 0.165354 1
T . . . Transmembrane . surface
glycoprotein_IWLGFIAGLIAIVMV 18 surface glycoprotein_QYIKWPWYI -1 .
. . . .
1 25217 25217 G T exonic S . nonsynonymous SNV

S:cds-YP_009724390.1:exon1:c.G3655T:p.G1219C 1.65 4.256 1 0.08 T
. . . Transmembrane . surface glycoprotein_IWLGFIAGLIAIVMV
18 . . . .

1 25218 25218 G T exonic S . nonsynonymous SNV
S:cds-YP_009724390.1:exon1:c.G3656T:p.G1219V 1.65 4.256 1 0.13 T
. . . Transmembrane . surface glycoprotein_IWLGFIAGLIAIVMV
18 . . . .

1 25249 25249 G T exonic S . nonsynonymous SNV
S:cds-YP_009724390.1:exon1:c.G3687T:p.M1229I 1.65 4.256 0.992126 0.16 T
. . . Transmembrane Surface glycoprotein_MVTIMLCCMTS
surface glycoprotein_IWLGFIAGLIAIVMV 18 . . . . . .
. . . .
1 25266 25266 G T exonic S . nonsynonymous SNV
S:cds-YP_009724390.1:exon1:c.G3704T:p.C1235F1.65 4.256 1 0.18 T .
. . . Surface glycoprotein_MVTIMLCCMTS . . . .
. . . .
1 25273 25273 G C exonic S . nonsynonymous SNV
S:cds-YP_009724390.1:exon1:c.G3711C:p.M1237I -0.274 1.80496 1 0.05 D
. . . . Surface glycoprotein_MVTIMLCCMTS . . .
. . . .
1 25284 25284 G T exonic S . nonsynonymous SNV
S:cds-YP_009724390.1:exon1:c.G3722T:p.C1241F1.65 4.256 1 0.04 D .
. . . . nCoV-2019_84_LEFT
. . . . .
1 25339 25339 C T exonic S . synonymous SNV
S:cds-YP_009724390.1:exon1:c.C3777T:p.D1259D -3.3 -1.71175 0.346457
1 T . . . . Spike
glycoprotein CKFDEDDSEPVLKGVKLHYT . . . . . .

1 25361 25361 G A exonic S . nonsynonymous SNV
S:cds-YP_009724390.1:exon1:c.G3799A:p.G1267R 1.65 4.256 0.992126 0.02
D . . . . surface glycoprotein_SEPVLKGVKL
-1 Spike glycoprotein CKFDEDDSEPVLKGVKLHYT nCoV-2019_83_RIGHT
. . . . .
1 25364 25364 G A exonic S . nonsynonymous SNV
S:cds-YP_009724390.1:exon1:c.G3802A:p.V1268I 1.65 4.256 1 0.56 T .
. . . . . . surface glycoprotein_SEPVLKGVKL -1 Spike
glycoprotein CKFDEDDSEPVLKGVKLHYT nCoV-2019_83_RIGHT . .
. . .
1 25429 25429 G T exonic ORF3a. nonsynonymous SNV
ORF3a:cds-YP_009724391.1:exon1:c.G37T:p.V13L 0.509 0.526157 0.0708661

0.1 T . . . . . ORF3a protein_MDLFMRIFTIGTVTL
16 ORF3a protein_RIFTIGTVTLK -1 . . . . . .
. .
1 25437 25437 G T exonic ORF3a. nonsynonymous SNV
ORF3a:cds-YP_009724391.1:exon1:c.G45T:p.L15F -3.06 -3.84309 0 0.24
T . . . . . ORF3a protein_MDLFMRIFTIGTVTL 16
ORF3a protein_RIFTIGTVTLK -1 . . . .
.
1 25452 25452 C T exonic ORF3a. synonymous SNV
ORF3a:cds-YP_009724391.1:exon1:c.C60T:p.I20I -1.02 -0.219811 0.0944882 1
T . . . .
. . . . .
1 25461 25461 T C exonic ORF3a. synonymous SNV
ORF3a:cds-YP_009724391.1:exon1:c.T69C:p.A23A 1.65 2.12466 0.992126
1 T . . . .
. . . . . .
1 25494 25494 G T exonic ORF3a. synonymous SNV
ORF3a:cds-YP_009724391.1:exon1:c.G102T:p.T34T -0.584 1.48526 1 1
T . . glyco Transmembrane . ORF3a
protein_SDFVRATATIPIQAS 14 ORF3a protein_IPIQASLPF -1 . . .
. . . . .
1 25496 25496 T C exonic ORF3a. nonsynonymous SNV
ORF3a:cds-YP_009724391.1:exon1:c.T104C:p.I35T 1.65 2.12466 1 0
D . . . Transmembrane . ORF3a
protein_SDFVRATATIPIQAS 14 ORF3a protein_IPIQASLPF -1 . . .
. . . . .
1 25507 25507 G A exonic ORF3a. nonsynonymous SNV
ORF3a:cds-YP_009724391.1:exon1:c.G115A:p.A39T 1.65 4.256 1 0 D
. . . Transmembrane . ORF3a protein_SDFVRATATIPIQAS
14 ORF3a protein_IPIQASLPF -1 . . . .
.
1 25521 25521 C T exonic ORF3a. synonymous SNV
ORF3a:cds-YP_009724391.1:exon1:c.C129T:p.F43F 0.791 1.91153 1 1
T . . . Transmembrane . . . ORF3a
protein_IPIQASLPF -1 . . . .

1 25528 25528 C T exonic ORF3a. nonsynonymous SNV
ORF3a:cds-YP_009724391.1:exon1:c.C136T:p.L46F 1.65 3.2969 1 0 D
. . . Transmembrane . . . .
. . . . . .
1 25549 25549 C T exonic ORF3a. nonsynonymous SNV
ORF3a:cds-YP_009724391.1:exon1:c.C157T:p.L53F 0.787 1.91153 1 0
D . . . Transmembrane . ORF3a protein_ALLAVFQSASKIITL

11 . . . .

1 25552 25552 G T exonic ORF3a. nonsynonymous SNV
ORF3a:cds-YP_009724391.1:exon1:c.G160T:p.A54S 0.738 2.76406 1 0
D . . . Transmembrane . ORF3a protein_ALLAVFQSASKIITL
11 . . . .

1 25556 25556 T G exonic ORF3a. nonsynonymous SNV
ORF3a:cds-YP_009724391.1:exon1:c.T164G:p.V55G 1.65 2.12466 1 0
D . . . Transmembrane . ORF3a protein_ALLAVFQSASKIITL
11 . . . .

1 25563 25563 G T exonic ORF3a. nonsynonymous SNV
ORF3a:cds-YP_009724391.1:exon1:c.G171T:p.Q57H -3.3 -1.17891 0.929134
0 D . . . . . ORF3a protein_ALLAVFQSASKIITL 11
ORF3a protein_SASKIITLK -1 . . . .
hypermutable low-fitness site
1 25565 25565 G T exonic ORF3a. nonsynonymous SNV
ORF3a:cds-YP_009724391.1:exon1:c.G173T:p.S58I 0.72 2.55093 1 0
D . . . . . ORF3a protein_ALLAVFQSASKIITL 11 ORF3a
protein_SASKIITLK -1 . . . .

1 25571 25571 C T exonic ORF3a. nonsynonymous SNV
ORF3a:cds-YP_009724391.1:exon1:c.C179T:p.S60F 1.65 3.2969 0.968504 0.01
D . . . . . ORF3a protein_ALLAVFQSASKIITL 11 ORF3a
protein_SASKIITLK -1 . . . .

1 25572 25572 C T exonic ORF3a. synonymous SNV
ORF3a:cds-YP_009724391.1:exon1:c.C180T:p.S60S -2.75 0.0998898 0.96063
1 T . . . . . ORF3a protein_ALLAVFQSASKIITL 11
ORF3a protein_SASKIITLK -1 . . . .

1 25634 25634 G T exonic ORF3a. nonsynonymous SNV
ORF3a:cds-YP_009724391.1:exon1:c.G242T:p.C81F 1.65 4.256 0.952756 0
D . . . Transmembrane . ORF3a
protein_GVHFVCNLLLLFVTV 20 . . . .
. . .
1 25644 25644 G T exonic ORF3a. synonymous SNV
ORF3a:cds-YP_009724391.1:exon1:c.G252T:p.L84L -1.62 0.892693 0.952756
1 T . . . Transmembrane . ORF3a
protein_GVHFVCNLLLLFVTV 20 . . . .
. . .
1 25647 25647 G T exonic ORF3a. nonsynonymous SNV

ORF3a:cds-YP_009724391.1:exon1:c.G255T:p.L85F -3.3 -1.91006 0 0
D . . . Transmembrane . ORF3a
protein_GVHFVCNLLLLFVTV 20 . . . .
nCoV-2019_84_RIGHT . . . . .
1 25654 25654 G T exonic ORF3a. nonsynonymous SNV
ORF3a:cds-YP_009724391.1:exon1:c.G262T:p.V88L -3.3 0.425567 0.0787402
0 D . . . Transmembrane . ORF3a
protein_GVHFVCNLLLLFVTV 20 . . . .
nCoV-2019_84_RIGHT . . . . .
1 25665 25665 C T exonic ORF3a. synonymous SNV
ORF3a:cds-YP_009724391.1:exon1:c.C273T:p.Y91Y -3.3 -3.21802 0 1
T . . . Transmembrane . . . ORF3a
protein_TVYSHLLLV 0.14 . . nCoV-2019_84_RIGHT . . .
. .
1 25677 25677 G T exonic ORF3a. nonsynonymous SNV
ORF3a:cds-YP_009724391.1:exon1:c.G285T:p.L95F -3.09 -0.882386 0 0
D . . . Transmembrane . . . ORF3a
protein_TVYSHLLLV 0.14 . . . .

1 25681 25681 G T exonic ORF3a. nonsynonymous SNV
ORF3a:cds-YP_009724391.1:exon1:c.G289T:p.V97F -3.3 -2.93774 0 0.09
T . . . Transmembrane . . . ORF3a
protein_TVYSHLLLV 0.14 . . . .

1 25688 25688 C T exonic ORF3a. nonsynonymous SNV
ORF3a:cds-YP_009724391.1:exon1:c.C296T:p.A99V 0.759 1.82694 0.984252
0 D . . . Transmembrane . . . . .
. . . .
1 25690 25690 G T exonic ORF3a. nonsynonymous SNV
ORF3a:cds-YP_009724391.1:exon1:c.G298T:p.G100C 1.65 2.7612 0.984252 0.2
T . . . Transmembrane . . . ORF3a
protein_GLEAPFLYLY -1 . . . .

1 25693 25693 C T exonic ORF3a. nonsynonymous SNV
ORF3a:cds-YP_009724391.1:exon1:c.C301T:p.L101F -1.02 0.612417 0.929134
0.06 T . . . Transmembrane . . . ORF3a
protein_GLEAPFLYLY -1 . . . .

1 25701 25701 C T exonic ORF3a. synonymous SNV
ORF3a:cds-YP_009724391.1:exon1:c.C309T:p.A103A -3.3 -2.84432 0.141732
1 T . . . Transmembrane . . . ORF3a
protein_GLEAPFLYLY -1 . . . .

1	25707	25707	T	C	exonic	ORF3a.	synonymous SNV
ORF3a:cds-YP_009724391.1:exon1:c.T315C:p.F105F	-3.3	-0.508685	0.661417
1	T	.	.	.	Transmembrane	.	ORF3a
protein_LYLYALVYFLQSINF 16	ORF3a protein_GLEAPFLYLY	-1	.	.
.	.	.	.	.	.

1	25726	25726	G	A	exonic	ORF3a.	nonsynonymous SNV
ORF3a:cds-YP_009724391.1:exon1:c.G334A:p.V112I	0.509	0.518992	0	1
T	.	.	.	Transmembrane	.	ORF3a protein_LYLYALVYFLQSINF
16	ORF3a protein_LYLYALVYF	-1	.	.	.	.	.	.	.
.

1	25733	25733	T	C	exonic	ORF3a.	nonsynonymous SNV
ORF3a:cds-YP_009724391.1:exon1:c.T341C:p.F114S	0.424	0.986118	0.220472
0.04	D	.	.	.	Transmembrane	.	ORF3a
protein_LYLYALVYFLQSINF 16	ORF3a protein_LYLYALVYF	-1	.	.	.
.	.	.	.	.

1	25745	25745	T	C	exonic	ORF3a.	nonsynonymous SNV
ORF3a:cds-YP_009724391.1:exon1:c.T353C:p.I118T	0.457	0.986118	0	0.03
D	.	.	.	.	.	ORF3a protein_LYLYALVYFLQSINF16	ORF3a
protein_VYFLQSINF	-1	.	.	.	.	.	.	.	.

1	25762	25762	A	T	exonic	ORF3a.	nonsynonymous SNV
ORF3a:cds-YP_009724391.1:exon1:c.A370T:p.I124L	-3.3	-1.81664	0	0
D	.	.	.	.	.	ORF3a protein_LVYFLQSINFVRIIM	7.9	.
.	.	.	.	.	.	.	.	.

1	25769	25769	G	T	exonic	ORF3a.	nonsynonymous SNV
ORF3a:cds-YP_009724391.1:exon1:c.G377T:p.R126M	1.65	4.256	1	0	D
.	.	.	.	.	ORF3a protein_QSINFVRIIMRLWLC	5.4	.
.	.	.	.	.	.	.	.	.

1	25780	25780	T	A	exonic	ORF3a.	nonsynonymous SNV
ORF3a:cds-YP_009724391.1:exon1:c.T388A:p.C130S	1.65	2.20065	0.992126
0	D	.	.	.	.	.	ORF3a protein_QSINFVRIIMRLWLC
5.4	.	.	.	.	.	.	.	.	.	.

1	25784	25784	G	T	exonic	ORF3a.	nonsynonymous SNV
ORF3a:cds-YP_009724391.1:exon1:c.G392T:p.W131L	1.65	4.256	1	0	D
.	.	.	.	.	.	.	.	.	.	.	.
.	.	.	.

1	25785	25785	G	T	exonic	ORF3a.	nonsynonymous SNV
ORF3a:cds-YP_009724391.1:exon1:c.G393T:p.W131C	0.748	2.7612	1	0	D
.	.	.	.	.	.	.	.	.	.	.	.
.	.	.	.

1	25803	25803	C	T	exonic	ORF3a.	synonymous SNV
ORF3a:cds-YP_009724391.1:exon1:c.C411T:p.N137N	-3.3	-0.41526	0.88189

1	T	.	.	.	.	.	ORF3a protein_KNPLLYDANYFLCWH
13	ORF3a protein_NPLLYDANY0.03	.	.	.	.	.	.	.
.
1	25819	25819	G	T	exonic	ORF3a.	nonsynonymous SNV
ORF3a:cds-YP_009724391.1:exon1:c.G427T:p.A143S	0.698	2.57435	1	0
D	.	.	.	.	.	ORF3a protein_KNPLLYDANYFLCWH	13
ORF3a protein_NPLLYDANY0.03	.	.	.	.	.	.	.	.

1	25836	25836	C	T	exonic	ORF3a.	synonymous SNV
ORF3a:cds-YP_009724391.1:exon1:c.C444T:p.C148C	-3.3	-0.60211	0.968504
1	T	.	.	.	.	.	ORF3a protein_KNPLLYDANYFLCWH
13	.	.	.	.	.	.	.	.	.	.

1	25855	25855	G	T	exonic	ORF3a.	nonsynonymous SNV
ORF3a:cds-YP_009724391.1:exon1:c.G463T:p.D155Y	1.65	4.256	1	0	D
.	.	.	.	.	.	.	.	.	.	.	.	.
.	.	.	.
1	25861	25861	T	C	exonic	ORF3a.	nonsynonymous SNV
ORF3a:cds-YP_009724391.1:exon1:c.T469C:p.C157R	1.65	2.20065	1	0
D	.	.	.	.	.	.	.	.	.	.	.	.
.	.	.	.	.
1	25878	25878	T	C	exonic	ORF3a.	synonymous SNV
ORF3a:cds-YP_009724391.1:exon1:c.T486C:p.S162S	-3.3	0.0518661	0.992126
1	T	.	.	.	.	.	.	.	ORF3a protein_PYNSVTSSI
0.08	.	.	.	.	.	.	.	.
1	25904	25904	C	T	exonic	ORF3a.	nonsynonymous SNV
ORF3a:cds-YP_009724391.1:exon1:c.C512T:p.S171L	-0.194	1.07954	0.92126
0.05	D	.	.	.	.	.	.	.	.	.	.	.
nCoV-2019_86_LEFT.	.	.	.	.
1	25906	25906	G	T	exonic	ORF3a.	nonsynonymous SNV
ORF3a:cds-YP_009724391.1:exon1:c.G514T:p.G172C	0.744	2.7612	1	0	D
.	.	.	.	.	.	.	.	.	.	.
nCoV-2019_86_LEFT.	.	.	.	.
1	25910	25910	A	G	exonic	ORF3a.	nonsynonymous SNV
ORF3a:cds-YP_009724391.1:exon1:c.A518G:p.D173G	1.65	2.29407	1	0
D	.	.	.	.	.	.	.	.	.	.	.
nCoV-2019_86_LEFT.	.	.	.	.
1	25916	25916	C	T	exonic	ORF3a.	nonsynonymous SNV
ORF3a:cds-YP_009724391.1:exon1:c.C524T:p.T175I	-3.3	-0.228409	0	0.44
T	.	.	.	.	.	.	.	.	.	.	.
nCoV-2019_86_LEFT.	.	.	.	.
1	25934	25934	A	G	exonic	ORF3a.	nonsynonymous SNV
ORF3a:cds-YP_009724391.1:exon1:c.A542G:p.E181G	0.505	0.986118	0.472441

0.05 D ORF3a
protein_SEHDYQIGGYTEKW -1 . . . .
.
1 25947 25947 G T exonic ORF3a. nonsynonymous SNV
ORF3a:cds-YP_009724391.1:exon1:c.G555T:p.Q185H 0.52 1.07954 1 0
D ORF3a
protein_SEHDYQIGGYTEKW -1 . . . .
.
1 26028 26028 C T exonic ORF3a. synonymous SNV
ORF3a:cds-YP_009724391.1:exon1:c.C636T:p.Y212Y 1.65 3.32175 1 1
T . . . . . ORF3a protein_YFTSDYYQLYSTQLS 16
ORF3a protein_YFTSDYYQLY -1 . . . .
.
1 26029 26029 C A exonic ORF3a. nonsynonymous SNV
ORF3a:cds-YP_009724391.1:exon1:c.C637A:p.Q213K 1.65 3.32175 1 0
D . . . . . ORF3a protein_YFTSDYYQLYSTQLS 16
ORF3a protein_YFTSDYYQLY -1 . . . .
.
1 26042 26042 C T exonic ORF3a. nonsynonymous SNV
ORF3a:cds-YP_009724391.1:exon1:c.C650T:p.T217I 1.65 3.32175 0.992126
0 D . . . . . ORF3a protein_YFTSDYYQLYSTQLS
16 ORF3a protein_YYQLYSTQL-1 . . . .
.
1 26060 26060 C T exonic ORF3a. nonsynonymous SNV
ORF3a:cds-YP_009724391.1:exon1:c.C668T:p.T223I 1.65 3.32175 1 0
D . . . . ORF3a protein_STDTGVEHVTFFIY
0.02 . . . .
1 26062 26062 G T exonic ORF3a. nonsynonymous SNV
ORF3a:cds-YP_009724391.1:exon1:c.G670T:p.G224C 1.65 4.256 1 0 D
. . . . ORF3a protein_STDTGVEHVTFFIY 0.02
. . . .
1 26065 26065 G T exonic ORF3a. nonsynonymous SNV
ORF3a:cds-YP_009724391.1:exon1:c.G673T:p.V225F -3.3 -0.0415591 0.944882
0.07 T ORF3a
protein_STDTGVEHVTFFIY 0.02 . . . .

1 26067 26067 T C exonic ORF3a. synonymous SNV
ORF3a:cds-YP_009724391.1:exon1:c.T675C:p.V225V 0.464 0.986118 0.976378
1 T ORF3a
protein_STDTGVEHVTFFIY 0.02 . . . .

1 26106 26106 T C exonic ORF3a. synonymous SNV
ORF3a:cds-YP_009724391.1:exon1:c.T714C:p.D238D -0.809 0.0518661 0.992126

0.59 T . . . .
. . . . . .
1 26111 26111 C T exonic ORF3a. nonsynonymous SNV
ORF3a:cds-YP_009724391.1:exon1:c.C719T:p.P240L -3.3 -0.508685 0 0
D . . . .
. . . . .
1 26113 26113 G C exonic ORF3a. nonsynonymous SNV
ORF3a:cds-YP_009724391.1:exon1:c.G721C:p.E241Q 0.154 0.238717 0 0.28
T . . . . ORF3a protein_EEHVQIHTI -1
. . . .
1 26152 26152 G A exonic ORF3a. nonsynonymous SNV
ORF3a:cds-YP_009724391.1:exon1:c.G760A:p.G254R 1.65 4.256 1 0 D
. . . .
. . . .
1 26152 26152 G T exonic ORF3a. stopgain
ORF3a:cds-YP_009724391.1:exon1:c.G760T:p.G254X 1.65 4.256 1 . .
. . . .
. . . .
1 26171 26171 T A exonic ORF3a. nonsynonymous SNV
ORF3a:cds-YP_009724391.1:exon1:c.T779A:p.M260K 1.33 1.82694 1 0
D . . . .
. . . . .
1 26173 26173 G T exonic ORF3a. stopgain
ORF3a:cds-YP_009724391.1:exon1:c.G781T:p.E261X 1.33 3.41517 1 .
. . . .
. . . . .
1 26185 26185 G T exonic ORF3a. nonsynonymous SNV
ORF3a:cds-YP_009724391.1:exon1:c.G793T:p.D265Y 1.65 4.256 1 0 D
. . . .
. . . .
1 26187 26187 T C exonic ORF3a. synonymous SNV
ORF3a:cds-YP_009724391.1:exon1:c.T795C:p.D265D 0.491 0.986118 1 1
T . . . .
. . . . .
1 26199 26199 G T exonic ORF3a. synonymous SNV
ORF3a:cds-YP_009724391.1:exon1:c.G807T:p.T269T 1.65 4.256 1 1 T
. . . .
nCoV-2019_87_LEFT. . . . .
1 26201 26201 C T exonic ORF3a. nonsynonymous SNV
ORF3a:cds-YP_009724391.1:exon1:c.C809T:p.T270I 1.65 3.32175 1 0
D . . . .
nCoV-2019_87_LEFT. . . . .
1 26204 26204 C T exonic ORF3a. nonsynonymous SNV

ORF3a:cds-YP_009724391.1:exon1:c.C812T:p.T271I 1.65 3.32175 1 0
D . . . .
nCoV-2019_87_LEFT . . . . .
1 26215 26215 T C exonic ORF3a . synonymous SNV
ORF3a:cds-YP_009724391.1:exon1:c.T823C:p.L275L 0.454 1.07954 1 1
T . . . .
nCoV-2019_87_LEFT . . . . .
1 26217 26217 G T exonic ORF3a . nonsynonymous SNV
ORF3a:cds-YP_009724391.1:exon1:c.G825T:p.L275F -0.309 1.54667 1 0
D . . . .
nCoV-2019_87_LEFT . . . . .
1 26333 26333 C T exonic E . nonsynonymous SNV
E:cds-YP_009724392.1:exon1:c.C89T:p.T30I 1.65 3.32175 1 0.17 T
. . . Transmembrane . envelope protein_SVLLFLAFVVFLLVT
17 . . . .

1 26439 26439 G T exonic E . synonymous SNV
E:cds-YP_009724392.1:exon1:c.G195T:p.L65L 1.65 4.256 1 1 T .
Intravirion . . . envelope protein_LVKPSFYVYSRVKNL 17
envelope protein_YVYSRVKNL 0.02 . . . .
.
1 26447 26447 C T exonic E . nonsynonymous SNV
E:cds-YP_009724392.1:exon1:c.C203T:p.S68F 1.65 3.32175 1 0 D
. Intravirion . . . envelope protein_FYVYSRVKNLNSSRV 15
. . . .

1 26456 26456 C T exonic E . nonsynonymous SNV
E:cds-YP_009724392.1:exon1:c.C212T:p.P71L -0.0954 1.26639 1 0
D . Intravirion . . . .
. . . . . .
1 26469 26469 C A exonic E . synonymous SNV
E:cds-YP_009724392.1:exon1:c.C225A:p.V75V 0.786 1.92037 1 1 T
. Intravirion . . . .
. . . . . .
1 26490 26490 T C upstream;downstream M;ORF6;ORF7a;E;ORF3a
dist=33;dist=18 . . 0.154 2.20065 1 . . . . .
. . . .
. .
1 26492 26492 A T upstream;downstream M;ORF6;ORF7a;E;ORF3a
dist=31;dist=20 . . 0.154 2.20065 1 . . . . .
. . . .
. .
1 26497 26497 T C upstream;downstream M;ORF6;ORF7a;E;ORF3a

dist=26;dist=25 . . 1.65 2.20065 1 . . . .
. . . .
. .
1 26555 26555 A G exonic M . synonymous SNV
M:cds-YP_009724393.1:exon1:c.A33G:p.E11E -1.74 0.238717 0.992126 1
T . Virion_surface . . . . . membrane
glycoprotein_GTITVEELK 0.09 . . . .

1 26690 26690 G T exonic M . synonymous SNV
M:cds-YP_009724393.1:exon1:c.G168T:p.L56L -3.13 -0.164252 0.96063 1
T . . . Transmembrane . . . .
. . . .
1 26735 26735 C T exonic M . synonymous SNV
M:cds-YP_009724393.1:exon1:c.C213T:p.Y71Y -0.165 1.12948 1 1 T
. Virion_surface . Transmembrane . membrane
glycoprotein_TLACFVLAAVYRINW 11 . . . .
. . .
1 26801 26801 C T exonic M . synonymous SNV
M:cds-YP_009724393.1:exon1:c.C279T:p.L93L 0.438 0.374803 1 0.5 T
. . . Transmembrane . membrane
glycoprotein_MWLSYFIASFRLFAR 3.9 membrane glycoprotein_SYFIASFRLF -1
. . . .
1 26831 26831 T C exonic M . synonymous SNV
M:cds-YP_009724393.1:exon1:c.T309C:p.F103F -0.966 0.374803 1 1 T
. Intravirion . . . membrane glycoprotein_MWLSYFIASFRLFAR
3.9 membrane glycoprotein_SYFIASFRLF -1 . . . . .
. . .
1 26873 26873 C T exonic M . synonymous SNV
M:cds-YP_009724393.1:exon1:c.C351T:p.N117N -3.03 0.0513701 0.992126 1
T . Intravirion . . Membrane glycoprotein_RTRSMWSFNPETN
. . . .

1 26882 26882 C T exonic M . synonymous SNV
M:cds-YP_009724393.1:exon1:c.C360T:p.L120L -1.46 0.482614 1 1 T
. Intravirion . . . .
. . . . .
1 26927 26927 A G exonic M . synonymous SNV
M:cds-YP_009724393.1:exon1:c.A405G:p.E135E 0.555 0.374803 1 1 T
. Intravirion . . . membrane glycoprotein_SELVIGAVILRGHLR
20 membrane glycoprotein_SELVIGAVIL 0.08 . . . . .
. . .
1 26951 26951 G C exonic M . synonymous SNV
M:cds-YP_009724393.1:exon1:c.G429C:p.V143V 0.712 2.63883 1 0.98 T

. Intravirion . . . membrane glycoprotein_SELVIGAVILRGHLR
20 membrane glycoprotein_SELVIGAVIL 0.08 . . . . .
. . .
1 26951 26951 G T exonic M . synonymous SNV
M:cds-YP_009724393.1:exon1:c.G429T:p.V143V 0.712 2.63883 1 0.98 T
. Intravirion . . . membrane glycoprotein_SELVIGAVILRGHLR
20 membrane glycoprotein_SELVIGAVIL 0.08 . . . . .
. . .
1 27005 27005 C T exonic M . synonymous SNV
M:cds-YP_009724393.1:exon1:c.C483T:p.I161I -3.3 -5.87824 0 1 T
. Intravirion . . . . Membrane
glycoprotein GRCDIKDLPKEITVATSR, PKEITVATSRTLSYYKL, TSRTLSYYKLGASQRV
. . . . . .
1 27092 27092 C T exonic M . synonymous SNV
M:cds-YP_009724393.1:exon1:c.C570T:p.D190D -3.3 -0.487685 0.984252 0.9
T . Intravirion . . Membrane
glycoprotein_ITVATSRTLSYYKLGASQRVAGDSGFAA membrane
glycoprotein_LSYYKLGASQRVAGD 12 membrane glycoprotein_ASQRVAGDSGFAAY
0.05 . . . .
1 27110 27110 C T exonic M . synonymous SNV
M:cds-YP_009724393.1:exon1:c.C588T:p.Y196Y 1.65 3.2857 1 1 T .
Intravirion . . . . . membrane
glycoprotein_ASQRVAGDSGFAAY 0.05 . . . .
.
1 27128 27128 C T exonic M . synonymous SNV
M:cds-YP_009724393.1:exon1:c.C606T:p.G202G -3.3 -0.703307 0.944882 1
T . Intravirion . . Membrane glycoprotein_NYKLNTDHSSSSDNIA
. . . .

1 27131 27131 C T exonic M . synonymous SNV
M:cds-YP_009724393.1:exon1:c.C609T:p.N203N -3.3 -1.13455 0.716535 1
T . Intravirion . . Membrane glycoprotein_NYKLNTDHSSSSDNIA
. . . .

1 27147 27147 G T exonic M . nonsynonymous SNV
M:cds-YP_009724393.1:exon1:c.G625T:p.D209Y 1.65 4.256 1 0.04 D .
Intravirion . . Membrane glycoprotein_NYKLNTDHSSSSDNIA . .
. . . . nCoV-2019_90_LEFT . . . . .

1 27213 27213 C T exonic ORF6 . synonymous SNV
ORF6:cds-YP_009724394.1:exon1:c.C12T:p.L4L -3.3 -1.67361 0.740157 1
T . . . . . ORF6 protein_MFHLVDFQVTIAEIL 17 ORF6
protein_HLVDFQVTI 0.03 . . nCoV-2019_89_RIGHT_alt4 . . .

. .
1 27214 27214 G T exonic ORF6 . nonsynonymous SNV
ORF6:cds-YP_009724394.1:exon1:c.G13T:p.V5F 1.65 4.256 0.992126 0 D
. . . . . ORF6 protein_MFHLVDFQVTIAEIL 17 ORF6
protein_HLVDFQVTI 0.03 . . nCoV-2019_89_RIGHT_alt4 . . .
. .
1 27263 27263 C T exonic ORF6 . nonsynonymous SNV
ORF6:cds-YP_009724394.1:exon1:c.C62T:p.T21I 1.65 3.2857 1 0 D .
. . . . ORF6 protein_IAEILLIIMRTFKVS 11 . . .
. . . .
1 27384 27384 T C exonic ORF6 . synonymous SNV
ORF6:cds-YP_009724394.1:exon1:c.T183C:p.D61D 0.462 0.913858 0.944882
1 T . . . .
. . . . . .
1 27389 27389 C T upstream;downstream
N;ORF7a;ORF7b;ORF8;E;M;ORF6 dist=5;dist=2 . . 0.791 1.99197 1
. . . .
. . . . . .
1 27434 27434 C T exonic ORF7a. nonsynonymous SNV
ORF7a:cds-YP_009724395.1:exon1:c.C41T:p.T14I -0.191 1.12948 0.125984 0.02
D . . . . . ORF7a protein_MKIILFLALITLATC 13 .
. . . .
1 27442 27442 C T exonic ORF7a. nonsynonymous SNV
ORF7a:cds-YP_009724395.1:exon1:c.C49T:p.L17F -3.3 -0.379874 0.984252
0 D . X4e . . . .
. . . . . .
1 27476 27476 C T exonic ORF7a. nonsynonymous SNV
ORF7a:cds-YP_009724395.1:exon1:c.C83T:p.T28I 1.65 3.2857 1 0 D
disulf_bond X4e . . . . . ORF7a protein_QECVRGTTVL
0.19 . . . .
1 27484 27484 T C exonic ORF7a. synonymous SNV
ORF7a:cds-YP_009724395.1:exon1:c.T91C:p.L31L -3.3 -0.272063 0.944882
1 T disulf_bond X4e . . . .
. . . .
1 27490 27490 G T exonic ORF7a. stopgain
ORF7a:cds-YP_009724395.1:exon1:c.G97T:p.E33X 1.65 4.256 1 . .
disulf_bond X4e . . . .
. . . . .
1 27504 27504 T C exonic ORF7a. synonymous SNV
ORF7a:cds-YP_009724395.1:exon1:c.T111C:p.S37S -3.3 -0.918929 0.913386
1 T disulf_bond X4e . . . .
. . . .
1 27600 27600 C T exonic ORF7a. synonymous SNV

ORF7a:cds-YP_009724395.1:exon1:c.C207T:p.D69D 0.761 1.88416 1 1
T . X4e . . . . . ORF7a protein_FAFACPDGVKHVY
0.1 . . . .
1 27688 27688 C T exonic ORF7a. nonsynonymous SNV
ORF7a:cds-YP_009724395.1:exon1:c.C295T:p.P99S 1.65 3.27687 0.976378
0 D . . . Transmembrane . ORF7a
protein_LYSPIFLIVAAIVFI 11 ORF7a protein_QELYSPIFL 0.03 . . .
. . . . .
1 27691 27691 A G exonic ORF7a. nonsynonymous SNV
ORF7a:cds-YP_009724395.1:exon1:c.A298G:p.I100V -0.271 0.535291 0.984252
0.11 T . . . Transmembrane . ORF7a
protein_LYSPIFLIVAAIVFI 11 ORF7a protein_QELYSPIFL 0.03 . . .
. . . . .
1 27705 27705 T G exonic ORF7a. synonymous SNV
ORF7a:cds-YP_009724395.1:exon1:c.T312G:p.V104V 0.411 0.926945 1 1
T . . . Transmembrane . ORF7a protein_LYSPIFLIVAAIVFI
11 . . . .

1 27708 27708 G T exonic ORF7a. synonymous SNV
ORF7a:cds-YP_009724395.1:exon1:c.G315T:p.A105A -3.3 -0.835496 0.937008
1 T . . . Transmembrane . ORF7a
protein_LYSPIFLIVAAIVFI 11 . . . .
. .
1 27741 27741 C T exonic ORF7a. synonymous SNV
ORF7a:cds-YP_009724395.1:exon1:c.C348T:p.L116L 0.219 0.241551 1 0.45
T . . . Transmembrane . . . . . .
. . . .
1 27752 27752 C T exonic ORF7a. nonsynonymous SNV
ORF7a:cds-YP_009724395.1:exon1:c.C359T:p.T120I -2.77 0.241551 0.992126
0 D . . . .
. . . . . .
1 27777 27777 G T exonic ORF7b. nonsynonymous SNV
ORF7b:cds-YP_009725318.1:exon1:c.G22T:p.D8Y 1.65 4.256 1 0 D .
. . . .
. . .
1 27798 27798 G T exonic ORF7b. nonsynonymous SNV
ORF7b:cds-YP_009725318.1:exon1:c.G43T:p.A15S 0.656 2.19982 1 0
D . . . Transmembrane . . . . . .
. nCoV-2019_92_LEFT . . . . .
1 27877 27877 G T exonic ORF7b. nonsynonymous SNV
ORF7b:cds-YP_009725318.1:exon1:c.G122T:p.C41F -0.666 1.12277 0.976378
0 D . . . .
. . . . . .

1 27879 27879 C T exonic ORF7b. nonsynonymous SNV
ORF7b:cds-YP_009725318.1:exon1:c.C124T:p.H42Y -1.05 -0.443843 0.92126
0.06 T . . . .
. . . . . .
1 27881 27881 C T exonic ORF7b. synonymous SNV
ORF7b:cds-YP_009725318.1:exon1:c.C126T:p.H42H 0.441 0.731118 0.976378
0.4 T . . . .
. . . . . .
1 27915 27915 G T exonic ORF8 . stopgain
ORF8:cds-YP_009724396.1:exon1:c.G22T:p.G8X -2.67 1.71025 0.992126 .
. . . . ORF8 protein_FLGIITTVAAFHQEC 17 .
. . . .
1 27922 27922 T C exonic ORF8 . nonsynonymous SNV
ORF8:cds-YP_009724396.1:exon1:c.T29C:p.I10T 1.33 1.71025 0 0.03 D
. . . . . . ORF8 protein_FLGIITTVAAFHQEC 17 . .
. . . .
1 27942 27942 C T exonic ORF8 . nonsynonymous SNV
ORF8:cds-YP_009724396.1:exon1:c.C49T:p.H17Y 0.983 0.143638 0.0314961 0
D . . . . . ORF8 protein_FLGIITTVAAFHQEC 17 .
. . . .
1 27958 27958 T C exonic ORF8 . nonsynonymous SNV
ORF8:cds-YP_009724396.1:exon1:c.T65C:p.L22S 1.33 -1.22715 0 0.01 D
. . . .
. . . .
1 27972 27972 C T exonic ORF8 . stopgain
ORF8:cds-YP_009724396.1:exon1:c.C79T:p.Q27X 0.364 0.829031 0.015748 .
. . . .
. . . . .
1 27978 27978 C T exonic ORF8 . stopgain
ORF8:cds-YP_009724396.1:exon1:c.C85T:p.Q29X -2.67 -1.22715 0 . .
. . . .
. . . .
1 27982 27982 C T exonic ORF8 . nonsynonymous SNV
ORF8:cds-YP_009724396.1:exon1:c.C89T:p.P30L 1.33 2.68939 0.645669 0
D . . . .
. . . . .
1 27992 27992 T C exonic ORF8 . synonymous SNV
ORF8:cds-YP_009724396.1:exon1:c.T99C:p.V33V -1.13 -0.150102 0.0551181 0.69
T . . . . ORF8 protein_VVDDPCPIHFY
0.02 . . . .
1 27998 27998 C T exonic ORF8 . synonymous SNV
ORF8:cds-YP_009724396.1:exon1:c.C105T:p.D35D -2.62 -1.03132 0.228346
1 T ORF8

protein_VVDDPCPIHFY 0.02 . . . .

1 27999 27999 C T exonic ORF8 . nonsynonymous SNV
ORF8:cds-YP_009724396.1:exon1:c.C106T:p.P36S 1.31 2.68939 0.448819
0 D ORF8
protein_VVDDPCPIHFY 0.02 . . . .

1 28001 28001 G T exonic ORF8 . synonymous SNV
ORF8:cds-YP_009724396.1:exon1:c.G108T:p.P36P -2.62 -3.87081 0.0629921
1 T ORF8
protein_VVDDPCPIHFY 0.02 . . . .

1 28005 28005 C T exonic ORF8 . nonsynonymous SNV
ORF8:cds-YP_009724396.1:exon1:c.C112T:p.P38S 0.434 2.59147 0.96063
0 D ORF8
protein_VVDDPCPIHFY 0.02 . . . .

1 28041 28041 G T exonic ORF8 . stopgain
ORF8:cds-YP_009724396.1:exon1:c.G148T:p.G50X -2.67 0.0457244 0 .
. . . . . . ORF8 protein_FYSKWYIRVGARKSA 13
. . . .

1 28045 28045 C T exonic ORF8 . nonsynonymous SNV
ORF8:cds-YP_009724396.1:exon1:c.C152T:p.A51V -0.5 0.241551 0 0.07
T . . . . . ORF8 protein_FYSKWYIRVGARKSA 13
. . . .

1 28077 28077 G C exonic ORF8 . nonsynonymous SNV
ORF8:cds-YP_009724396.1:exon1:c.G184C:p.V62L 0.427 1.02486 0 0.34
T . . . .
. . . . hypermutable low-fitness site
1 28077 28077 G T exonic ORF8 . nonsynonymous SNV
ORF8:cds-YP_009724396.1:exon1:c.G184T:p.V62L 0.427 1.02486 0 0.34
T . . . .
. . . . hypermutable low-fitness site
1 28079 28079 G T exonic ORF8 . synonymous SNV
ORF8:cds-YP_009724396.1:exon1:c.G186T:p.V62V -2.67 -0.835496 0 0.6
T . . . .
. . . . .
1 28086 28086 G T exonic ORF8 . nonsynonymous SNV
ORF8:cds-YP_009724396.1:exon1:c.G193T:p.A65S -2.67 -0.150102 0.00787402
0.47 T . . . .
nCoV-2019_93_LEFT . . . . .

1 28139 28139 C T exonic ORF8 . synonymous SNV
ORF8:cds-YP_009724396.1:exon1:c.C246T:p.S82S -2.67 -1.32506 0 1
T . . . .
. . . . .
1 28183 28183 G T exonic ORF8 . nonsynonymous SNV
ORF8:cds-YP_009724396.1:exon1:c.G290T:p.S97I-2.67 -1.32506 0 0 D
. . . .
. . . .
1 28195 28195 G T exonic ORF8 . nonsynonymous SNV
ORF8:cds-YP_009724396.1:exon1:c.G302T:p.R101L 1.33 3.37478 0.00787402
0 D . . . .
. . . . . .
1 28221 28221 G T exonic ORF8 . stopgain
ORF8:cds-YP_009724396.1:exon1:c.G328T:p.E110X -2.67 -0.345929 0 .
. . . . ORF8 protein_YEDFLEYHDVRVVL
-1 . . . .
1 28237 28237 G T exonic ORF8 . nonsynonymous SNV
ORF8:cds-YP_009724396.1:exon1:c.G344T:p.R115L -2.62 -0.933409 0 0
D . . . . ORF8 protein_YEDFLEYHDVRVVL
-1 . . . .
1 28253 28253 C T exonic ORF8 . synonymous SNV
ORF8:cds-YP_009724396.1:exon1:c.C360T:p.F120F 0.114 0.241551 0.992126
1 T . . . .
. . . . . .
1 28277 28277 T C exonic N . nonsynonymous SNV
N:cds-YP_009724397.2:exon1:c.T4C:p.S2P0.404 0.829031 1 0 D .
. . . .
. . .
1 28291 28291 C T exonic N . synonymous SNV
N:cds-YP_009724397.2:exon1:c.C18T:p.P6P 0.701 1.51443 1 0 D
. . . .
2019-nCoV_N1-F Centers for Disease Control and Prevention (CDC); KRISHGEN
BIOSYSTEMS. . .
1 28300 28300 G T exonic N . nonsynonymous SNV
N:cds-YP_009724397.2:exon1:c.G27T:p.Q9H -3.3 -0.835496 0.23622 0.03
D . . . .
2019-nCoV_N1-F Centers for Disease Control and Prevention (CDC); KRISHGEN
BIOSYSTEMS. . .
1 28310 28310 C A exonic N . nonsynonymous SNV
N:cds-YP_009724397.2:exon1:c.C37A:p.P13T -0.112 1.22069 1 0.02 D
. 9b . . . .
. . . .
1 28310 28310 C T exonic N . nonsynonymous SNV

N:cds-YP_009724397.2:exon1:c.C37T:p.P13S -0.112 1.22069 1 0.2 T
. 9b . . . .
. . . .
1 28311 28311 C T exonic N . nonsynonymous SNV
N:cds-YP_009724397.2:exon1:c.C38T:p.P13L 1.65 3.27687 1 0 D
. 9b . . . .
. . . .
1 28325 28325 G A exonic N . nonsynonymous SNV
N:cds-YP_009724397.2:exon1:c.G52A:p.G18S 1.65 4.256 1 0.22 T .
9b . . . . WH-NIC
N-Forward Thailand . . .
1 28326 28326 G T exonic N . nonsynonymous SNV
N:cds-YP_009724397.2:exon1:c.G53T:p.G18V 1.65 4.256 1 0 D .
9b . . . . WH-NIC
N-Forward Thailand . . .
1 28360 28360 T C exonic N . synonymous SNV
N:cds-YP_009724397.2:exon1:c.T87C:p.N29N 0.326 1.02486 0.992126 0
D . 9b . . Nucleocapsid phosphoprotein_DSTGSNQNGE .
. . . . . . WH-NIC N-Reverse Thailand . .
.
1 28368 28368 G T exonic N . nonsynonymous SNV
N:cds-YP_009724397.2:exon1:c.G95T:p.R32L 1.65 4.256 1 0 D .
9b . . . . WH-NIC
N-Reverse Thailand . . .
1 28373 28373 G T exonic N . nonsynonymous SNV
N:cds-YP_009724397.2:exon1:c.G100T:p.G34W 1.65 4.256 1 0.01 D .
9b . . . . WH-NIC
N-Reverse Thailand . . .
1 28399 28399 C T exonic N . synonymous SNV
N:cds-YP_009724397.2:exon1:c.C126T:p.P42P -2.55 0.241551 1 1 T
. 9b . . . .
nCoV-2019_94_LEFT . . . . . .
1 28473 28473 C T exonic N . nonsynonymous SNV
N:cds-YP_009724397.2:exon1:c.C200T:p.P67L 1.65 3.27687 1 0 D
. 9b . . . . . nucleocapsid
phosphoprotein_KFPRGQGVPI 0.04 . . . .
.
1 28476 28476 G C exonic N . nonsynonymous SNV
N:cds-YP_009724397.2:exon1:c.G203C:p.R68P -0.211 1.80817 1 0 D
. 9b . . . . . nucleocapsid
phosphoprotein_KFPRGQGVPI 0.04 . . . .
.
1 28487 28487 G A exonic N . nonsynonymous SNV

N:cds-YP_009724397.2:exon1:c.G214A:p.V72I 1.65 4.256 1 0.01 D .
9b . . . . . nucleocapsid phosphoprotein_KFPRGQGVPI
0.04 . . . .
1 28507 28507 C T exonic N . synonymous SNV
N:cds-YP_009724397.2:exon1:c.C234T:p.S78S 0.474 0.437378 0.984252 0.15
T . 9b . . . . . nucleocapsid
phosphoprotein_NTNSSPDDQIGYY -1 . . . .
.
1 28515 28515 A G exonic N . nonsynonymous SNV
N:cds-YP_009724397.2:exon1:c.A242G:p.D81G 1.65 2.29773 1 0 D
. 9b . . . nucleocapsid phosphoprotein_DDQIGYYRRATRRIR
9.2 nucleocapsid phosphoprotein_NTNSSPDDQIGYY -1 . . . .
. . . .
1 28621 28621 G T exonic N . synonymous SNV
N:cds-YP_009724397.2:exon1:c.G348T:p.G116G -0.415 0.339465 1 1 T
. . . .
. . . .
1 28628 28628 G T exonic N . nonsynonymous SNV
N:cds-YP_009724397.2:exon1:c.G355T:p.A119S 1.65 4.256 1 0.01 D .
. . . .
. . .
1 28651 28651 C T exonic N . synonymous SNV
N:cds-YP_009724397.2:exon1:c.C378T:p.N126N -2.24 0.241551 1 0.66 T
. . . .
. . . .
1 28657 28657 C T exonic N . synonymous SNV
N:cds-YP_009724397.2:exon1:c.C384T:p.D128D -2.4 -0.052189 1 1 T
. . . .
. . . .
1 28690 28690 G T exonic N . nonsynonymous SNV
N:cds-YP_009724397.2:exon1:c.G417T:p.L139F -1.73 0.803039 0.992126 0.22
T . . . .
nCoV-2019_95_LEFT 2019-nCoV_N3-F Centers for Disease Control and Prevention (CDC);
KRISHGEN BIOSYSTEMS . . .
1 28692 28692 A T exonic N . nonsynonymous SNV
N:cds-YP_009724397.2:exon1:c.A419T:p.N140I 1.65 2.25106 0.937008 0.11
T . . . .
nCoV-2019_95_LEFT 2019-nCoV_N3-F Centers for Disease Control and Prevention (CDC);
KRISHGEN BIOSYSTEMS . . .
1 28693 28693 T C exonic N . synonymous SNV
N:cds-YP_009724397.2:exon1:c.T420C:p.N140N -3.3 -4.87764 0 0.5 T
. . . .
nCoV-2019_95_LEFT 2019-nCoV_N3-F Centers for Disease Control and Prevention (CDC);

KRISHGEN BIOSYSTEMS . . .
1 28706 28706 C T exonic N . nonsynonymous SNV
N:cds-YP_009724397.2:exon1:c.C433T:p.H145Y 1.65 3.25353 0.984252 0.09
T . . . .
NIID-N1-F | CDCV_GERMANY_N gene_1 National Institute of Infectious Disease (NIID), Yamanashi Central Hospital (YCH) | Christian Drosten Charite Virology, Berlin, Germany
. . .
1 28712 28712 G A exonic N . nonsynonymous SNV
N:cds-YP_009724397.2:exon1:c.G439A:p.G147S 1.65 4.256 1 0.51 T .
. . . . NIID-N1-F |
CDCV_GERMANY_N gene_1 National Institute of Infectious Disease (NIID), Yamanashi Central Hospital (YCH) | Christian Drosten Charite Virology, Berlin, Germany . .
.
1 28712 28712 G T exonic N . nonsynonymous SNV
N:cds-YP_009724397.2:exon1:c.G439T:p.G147C 1.65 4.256 1 0.09 T .
. . . . NIID-N1-F |
CDCV_GERMANY_N gene_1 National Institute of Infectious Disease (NIID), Yamanashi Central Hospital (YCH) | Christian Drosten Charite Virology, Berlin, Germany . .
.
1 28723 28723 T C exonic N . synonymous SNV
N:cds-YP_009724397.2:exon1:c.T450C:p.N150N -0.778 0.357496 1 0.58 T
. . . .
NIID-N1-F | CDCV_GERMANY_N gene_1 National Institute of Infectious Disease (NIID), Yamanashi Central Hospital (YCH) | Christian Drosten Charite Virology, Berlin, Germany
. . .
1 28728 28728 C T exonic N . nonsynonymous SNV
N:cds-YP_009724397.2:exon1:c.C455T:p.A152V 0.509 0.357496 1 0.03 D
. . . .
. . . .
1 28775 28775 C T exonic N . nonsynonymous SNV
N:cds-YP_009724397.2:exon1:c.C502T:p.P168S 1.65 3.25353 1 0 D
. . . .
nCoV-2019_94_RIGHT . . . . .
1 28806 28806 G T exonic N . nonsynonymous SNV
N:cds-YP_009724397.2:exon1:c.G533T:p.G178V 0.707 2.6966 1 0 D .
. . . Nucleocapsid
phosphoprotein_RGGSQASSRSSSRSRNSSRNSTPGSSRGTSPARMAGNGG . .
. . Nucleocapsid phosphoprotein SRGGSQASSRSSSRSR . .
. . . .
1 28809 28809 G T exonic N . nonsynonymous SNV
N:cds-YP_009724397.2:exon1:c.G536T:p.G179V 1.65 4.256 1 0 D .
. . . Nucleocapsid
phosphoprotein_RGGSQASSRSSSRSRNSSRNSTPGSSRGTSPARMAGNGG . .

. . Nucleocapsid phosphoprotein SRGGSQASSRSSSRSR . .
. . . .
1 28821 28821 C A exonic N . nonsynonymous SNV
N:cds-YP_009724397.2:exon1:c.C548A:p.S183Y 1.65 3.25353 1 0 D
. . . . Nucleocapsid
phosphoprotein_RGGSQASSRSSSRSRNSSRNSTPGSSRGTSPARMAGNGG . .
. . Nucleocapsid phosphoprotein SRGGSQASSRSSSRSR .
NIID-N1-R | CDCV_GERMANY_N gene_2 National Institute of Infectious Disease (NIID); Yamanashi Central Hospital (YCH) | Christian Drosten Charite Virology, Berlin, Germany
. . .
1 28847 28847 A T exonic N . nonsynonymous SNV
N:cds-YP_009724397.2:exon1:c.A574T:p.N192Y 0.695 1.69413 1 0.01 D
. . . . Nucleocapsid
phosphoprotein_RGGSQASSRSSSRSRNSSRNSTPGSSRGTSPARMAGNGG . .
. . . . . SARS-CoV-2_IBS_N1_R | SARS-CoV-2_IBS_N2_R
Korea | Korea . . .
1 28851 28851 G T exonic N . nonsynonymous SNV
N:cds-YP_009724397.2:exon1:c.G578T:p.S193I 0.509 0.580268 0.992126 0.03
D . . . . Nucleocapsid
phosphoprotein_RGGSQASSRSSSRSRNSSRNSTPGSSRGTSPARMAGNGG . .
. . . . . SARS-CoV-2_IBS_N2_R Korea . . .

1 28854 28854 C T exonic N . nonsynonymous SNV
N:cds-YP_009724397.2:exon1:c.C581T:p.S194L 1.65 3.25353 1 . .
. . . . Nucleocapsid
phosphoprotein_RGGSQASSRSSSRSRNSSRNSTPGSSRGTSPARMAGNGG . .
. . . . . SARS-CoV-2_IBS_N2_R Korea . .
hypermutable low-fitness site
1 28857 28857 G T exonic N . nonsynonymous SNV
N:cds-YP_009724397.2:exon1:c.G584T:p.R195I 0.745 2.6966 1 0 D .
. . . Nucleocapsid
phosphoprotein_RGGSQASSRSSSRSRNSSRNSTPGSSRGTSPARMAGNGG . .
. . . .

1 28866 28866 C T exonic N . nonsynonymous SNV
N:cds-YP_009724397.2:exon1:c.C593T:p.T198I 1.65 3.25353 1 0 D
. . . . Nucleocapsid
phosphoprotein_RGGSQASSRSSSRSRNSSRNSTPGSSRGTSPARMAGNGG . .
. . . .

1 28871 28871 G T exonic N . nonsynonymous SNV
N:cds-YP_009724397.2:exon1:c.G598T:p.G200C 0.646 2.25106 1 0.01 D
. . . Transmembrane Nucleocapsid

phosphoprotein_RGGSQASSRSSSRSRNSSRNSTPGSSRGTSPARMAGNGG . .
. . . .

1 28878 28878 G A exonic N . nonsynonymous SNV
N:cds-YP_009724397.2:exon1:c.G605A:p.S202N 1.65 4.256 1 0 D .
. . Transmembrane Nucleocapsid
phosphoprotein_RGGSQASSRSSSRSRNSSRNSTPGSSRGTSPARMAGNGG . .
. . . .

1 28881 28881 G A exonic N . nonsynonymous SNV
N:cds-YP_009724397.2:exon1:c.G608A:p.R203K 1.65 4.256 1 0 D .
. . Transmembrane Nucleocapsid
phosphoprotein_RGGSQASSRSSSRSRNSSRNSTPGSSRGTSPARMAGNGG . .
. . . . . N gene Forward GenScript . . .

1 28895 28895 G T exonic N . nonsynonymous SNV
N:cds-YP_009724397.2:exon1:c.G622T:p.A208S 1.65 4.256 1 0.82 T .
. . Transmembrane Nucleocapsid
phosphoprotein_RGGSQASSRSSSRSRNSSRNSTPGSSRGTSPARMAGNGG . .
. . . . . N gene Forward GenScript . . .

1 28903 28903 G T exonic N . nonsynonymous SNV
N:cds-YP_009724397.2:exon1:c.G630T:p.M210I -0.336 2.6966 1 0 D .
. . Transmembrane Nucleocapsid
phosphoprotein_RGGSQASSRSSSRSRNSSRNSTPGSSRGTSPARMAGNGG . .
. . . .

1 28906 28906 T C exonic N . synonymous SNV
N:cds-YP_009724397.2:exon1:c.T633C:p.A211A 0.456 0.914425 1 0 D
. . . Transmembrane Nucleocapsid
phosphoprotein_RGGSQASSRSSSRSRNSSRNSTPGSSRGTSPARMAGNGG . .
. . . .

1 28980 28980 G T exonic N . nonsynonymous SNV
N:cds-YP_009724397.2:exon1:c.G707T:p.G236V 1.65 4.256 1 0.04 D .
. . . Nucleocapsid phosphoprotein_SKMSGKGQQQQGQTVTKKSA .
. . . .

1 28981 28981 T C exonic N . synonymous SNV
N:cds-YP_009724397.2:exon1:c.T708C:p.G236G 0.326 1.02581 1 1 T
. . . . Nucleocapsid phosphoprotein_SKMSGKGQQQQGQTVTKKSA
. . . .

1 28987 28987 C T exonic N . synonymous SNV
N:cds-YP_009724397.2:exon1:c.C714T:p.G238G 0.701 1.47135 1 1 T
. . . . Nucleocapsid phosphoprotein_SKMSGKGQQQQGQTVTKKSA
. . . . . . nCoV-2019_96_LEFT . . . .
.
1 28990 28990 A T exonic N . nonsynonymous SNV
N:cds-YP_009724397.2:exon1:c.A717T:p.Q239H 1.53 2.25106 1 0.04 D
. . . . Nucleocapsid phosphoprotein_SKMSGKGQQQQGQTVTKKSA
. . . . . . nCoV-2019_96_LEFT . . . .
.
1 29047 29047 T C exonic N . synonymous SNV
N:cds-YP_009724397.2:exon1:c.T774C:p.P258P -0.807 0.468882 1 0.37 T
. . . . Nucleocapsid
phosphoprotein QQQGQTVTKKSAAEASKK, KKSAAEASKKPRQKRTA
nCoV-2019_95_RIGHT . . . . .
1 29137 29137 C T exonic N . synonymous SNV
N:cds-YP_009724397.2:exon1:c.C864T:p.D288D 0.776 1.9169 1 1 T .
. . . . . . nucleocapsid phosphoprotein_QELIRQGTDYKHW
0.11 . . . NIID_N2_F National Institute of Infectious Disease (NIID);
Yamanashi Central Hospital (YCH) . . .
1 29177 29177 C T exonic N . nonsynonymous SNV
N:cds-YP_009724397.2:exon1:c.C904T:p.P302S 1.65 3.25353 1 0.02 D
. . . . . nucleocapsid phosphoprotein_WPQIAQFAPSASAFF
11 . . Nucleocapsid phosphoprotein IRQGTDYKHWPQIAQFA,
QFAPSASAFFGMSRIGM, FFGMSRIGMEVTPSGTW . 2019-nCoV_N2-F
Centers for Disease Control and Prevention (CDC); KRISHGEN BIOSYSTEMS . .
.
1 29179 29179 G T exonic N . synonymous SNV
N:cds-YP_009724397.2:exon1:c.G906T:p.P302P -0.391 1.47135 1 1 T
. . . . . nucleocapsid phosphoprotein_WPQIAQFAPSASAFF
11 . . Nucleocapsid phosphoprotein IRQGTDYKHWPQIAQFA,
QFAPSASAFFGMSRIGM, FFGMSRIGMEVTPSGTW . 2019-nCoV_N2-F
Centers for Disease Control and Prevention (CDC); KRISHGEN BIOSYSTEMS . .
.
1 29218 29218 C T exonic N . synonymous SNV
N:cds-YP_009724397.2:exon1:c.C945T:p.F315F -3.3 -0.199433 0.992126 1
T . . . . . nucleocapsid
phosphoprotein_WPQIAQFAPSASAFF 11 nucleocapsid
phosphoprotein_ASAFFGMSR 0.05 Nucleocapsid phosphoprotein
IRQGTDYKHWPQIAQFA, QFAPSASAFFGMSRIGM, FFGMSRIGMEVTPSGTW .
2019-nCoV_N2-R Centers for Disease Control and Prevention (CDC); KRISHGEN
BIOSYSTEMS . . .
1 29227 29227 G T exonic N . synonymous SNV

N:cds-YP_009724397.2:exon1:c.G954T:p.S318S -2.29 0.134724 1 1 T
. . . . . nucleocapsid phosphoprotein_QFAPSASAFFGMSRI
17 nucleocapsid phosphoprotein_ASAFFGMSR 0.05 Nucleocapsid
phosphoprotein IRQGTDYKHWPQIAQFA, QFAPSASAFFGMSRIGM,
FFGMSRIGMEVTPSGTW . 2019-nCoV_N2-R Centers for Disease Control and
Prevention (CDC); KRISHGEN BIOSYSTEMS . . .

1 29254 29254 G T exonic N . synonymous SNV
N:cds-YP_009724397.2:exon1:c.G981T:p.S327S 0.646 2.25106 1 0.66 T
. . . . . nucleocapsid phosphoprotein_PSGTWLTYTGAIKLD
17 nucleocapsid phosphoprotein_SRIGMEVTPSGTW 0.08 Nucleocapsid
phosphoprotein IRQGTDYKHWPQIAQFA, QFAPSASAFFGMSRIGM,
FFGMSRIGMEVTPSGTW . HKU_N_Reverse HongKong University . .
.
1 29260 29260 G T exonic N . synonymous SNV
N:cds-YP_009724397.2:exon1:c.G987T:p.T329T -2.28 0.0233386 1 0.5 T
. . . . . nucleocapsid phosphoprotein_PSGTWLTYTGAIKLD
17 nucleocapsid phosphoprotein_SRIGMEVTPSGTW 0.08 Nucleocapsid
phosphoprotein IRQGTDYKHWPQIAQFA, QFAPSASAFFGMSRIGM,
FFGMSRIGMEVTPSGTW . . . . . .

1 29272 29272 C T exonic N . synonymous SNV
N:cds-YP_009724397.2:exon1:c.C999T:p.Y333Y 0.482 0.24611 1 1 T
. . . . . nucleocapsid phosphoprotein_PSGTWLTYTGAIKLD
17 nucleocapsid phosphoprotein_TPSGTWLTY 0.01 . . .
NIID_N2_R National Institute of Infectious Disease (NIID); Yamanashi Central Hospital (YCH
) . . .
1 29337 29337 A G exonic N . nonsynonymous SNV
N:cds-YP_009724397.2:exon1:c.A1064G:p.K355R 1.65 2.25106 0.992126 0.02
D . . . . . . nucleocapsid phosphoprotein_FKDQVILLNKHIDAY
15 . . . .

1 29353 29353 C T exonic N . synonymous SNV
N:cds-YP_009724397.2:exon1:c.C1080T:p.Y360Y -1.36 0.468882 1 1 T
. . . . . nucleocapsid phosphoprotein_FKDQVILLNKHIDAY 15
nucleocapsid phosphoprotein_AYKTFPPTEPK -1 Nucleocapsid phosphoprotein
YKTFPPTEPKKDKKKK . . . . . .

1 29379 29379 A T exonic N . nonsynonymous SNV
N:cds-YP_009724397.2:exon1:c.A1106T:p.K369I 1.65 2.25106 1 0.08 T
. . . . nucleocapsid
phosphoprotein_AYKTFPPTEPK -1 Nucleocapsid phosphoprotein
YKTFPPTEPKKDKKKK . . . . . .

1 29386 29386 C T exonic N . synonymous SNV
N:cds-YP_009724397.2:exon1:c.C1113T:p.D371D 0.789 1.9169 1 0.68 T .
. . . . Nucleocapsid phosphoprotein
YKTFPPTEPKKDKKKK . . . . . .

1 29389 29389 A G exonic N . synonymous SNV
N:cds-YP_009724397.2:exon1:c.A1116G:p.K372K -0.742 0.468882 1 1 T
. . . . Nucleocapsid
phosphoprotein YKTFPPTEPKKDKKKK . . . . . .

1 29389 29389 A T exonic N . nonsynonymous SNV
N:cds-YP_009724397.2:exon1:c.A1116T:p.K372N -0.742 0.468882 1 0.07 T
. . . . Nucleocapsid
phosphoprotein YKTFPPTEPKKDKKKK . . . . . .

1 29403 29403 A T exonic N . nonsynonymous SNV
N:cds-YP_009724397.2:exon1:c.A1130T:p.D377V 1.65 2.25106 1 0.13 T
. . . .
. . . .
1 29427 29427 G T exonic N . nonsynonymous SNV
N:cds-YP_009724397.2:exon1:c.G1154T:p.R385I 1.65 4.256 1 0.04 D .
. . . .
. . .
1 29449 29449 G T exonic N . synonymous SNV
N:cds-YP_009724397.2:exon1:c.G1176T:p.V392V 0.695 2.47383 1 1 T
. . . .
. . . .
1 29462 29462 G T exonic N . nonsynonymous SNV
N:cds-YP_009724397.2:exon1:c.G1189T:p.A397S 1.65 4.256 1 0.52 T .
. . . . . . nucleocapsid phosphoprotein_LPAADLDDF 0.06
. . . .
1 29474 29474 G T exonic N . nonsynonymous SNV
N:cds-YP_009724397.2:exon1:c.G1201T:p.D401Y 1.65 4.256 1 0.01 D .
. . . . . . nucleocapsid phosphoprotein_LPAADLDDF 0.06
. . . .
1 29523 29523 C T exonic N . nonsynonymous SNV
N:cds-YP_009724397.2:exon1:c.C1250T:p.T417I 1.65 3.25353 1 0 D
. . . .
. . . .
1 29540 29540 G T upstream;downstream ORF10;N dist=18;dist=7
. . 1.65 4.256 1 . . . .
. . . .

1 29543 29543 G T upstream;downstream ORF10;N
dist=15;dist=10 . . 1.65 4.256 1 . . . . .
. . . .
.

1 29555 29555 C T upstream;downstream ORF10;N dist=3;dist=22
. . 0.786 1.9169 1 . . . .
. . . .

1 29600 29600 A G exonic ORF10. nonsynonymous SNV
ORF10:cds-YP_009725255.1:exon1:c.A43G:p.S15G 1.6 2.25106 1 .
. . . . . . ORF10 protein_MGYINVFAFPFTIYS 9.1
ORF10 protein_AFPFTIYSL 0.09 . . . .

1 29630 29630 A G exonic ORF10. nonsynonymous SNV
ORF10:cds-YP_009725255.1:exon1:c.A73G:p.N25D 1.6 2.25106 1 .
. . . .
. . . . .

1 29632 29632 C A exonic ORF10. nonsynonymous SNV
ORF10:cds-YP_009725255.1:exon1:c.C75A:p.N25K 1.6 3.25353 1 .
. . . .
. . . . .

1 29645 29645 G A exonic ORF10. nonsynonymous SNV
ORF10:cds-YP_009725255.1:exon1:c.G88A:p.V30I1.6 4.14461 1 . .
. . . .
. . . .

1 29675 29675 C T downstream N;ORF10 dist=1 . . 1.6
3.25353 1 . . . .
. . . .

1 29679 29679 C T downstream N;ORF10 dist=5 . . 1.6
3.25353 1 . . . .
. . . .

1 29686 29686 C T downstream N;ORF10 dist=12. . -1.19
0.580268 1 . . . .
. . . .

1 29688 29688 G T downstream N;ORF10 dist=14. . -0.815
0.580268 1 . . . .
. . . .

1 29715 29715 G T downstream N;ORF10 dist=41. . 1.58
4.076 1 . . . .
. . . .

1 29721 29721 C T downstream N;ORF10 dist=47. . 1.58
3.20945 1 . . . .

.	.	.	.	.	.	.	.	.				
1	29730	29730	C	T	downstream		N;ORF10		dist=56.		.	1.58
3.20945		1	.	.	.	.	.	.	.	.	.	.
.	.	.	.	.	.	.	.	.				
1	29732	29732	C	T	downstream		N;ORF10		dist=58.		.	0.714
1.86148		1	.	.	.	.	.	.	.	.	.	.
.	.	.	.	.	.	.	.	.				
1	29733	29733	C	T	downstream		N;ORF10		dist=59.		.	1.58
3.20945		1	.	.	.	.	.	.	.	.	.	.
.	.	.	.	.	.	.	.	.				
1	29734	29734	G	T	downstream		N;ORF10		dist=60.		.	1.58
4.076	1	.	.	.	.	.	.	.	.	.	.	.
.	.	.	.	.	.	.	.					
1	29736	29736	G	T	downstream		N;ORF10		dist=62.		.	1.58
4.076	1	.	.	.	.	.	.	.	.	.	.	.
.	.	.	.	.	.	.	.					
1	29741	29741	C	T	downstream		N;ORF10		dist=67.		.	1.54
3.11317		1	.	.	.	.	.	.	.	.	.	.
.	.	.	.	.	.	.	.	.				
1	29742	29742	G	A	downstream		N;ORF10		dist=68.		.	1.54
3.97972		1	.	.	.	.	.	.	.	.	.	.
.	.	.	.	.	.	.	.	.				
1	29742	29742	G	C	downstream		N;ORF10		dist=68.		.	1.54
3.97972		1	.	.	.	.	.	.	.	.	.	.
.	.	.	.	.	.	.	.	.				
1	29753	29753	T	C	downstream		N;ORF10		dist=79.		.	1.54
2.08614		1	.	.	.	.	.	.	.	.	.	.
.	.	.	.	.	.	.	.	.				
1	29754	29754	C	T	downstream		N;ORF10		dist=80.		.	1.54
3.11317		1	.	.	.	.	.	.	.	.	.	.
.	.	.	.	.	.	.	.	.				
1	29755	29755	G	T	downstream		N;ORF10		dist=81.		.	1.54
3.97972		1	.	.	.	.	.	.	.	.	.	.
.	.	.	.	.	.	.	.	.				
1	29760	29760	T	C	downstream		N;ORF10		dist=86.		.	1.54
2.08614		1	.	.	.	.	.	.	.	.	.	.
.	.	.	.	.	.	.	.	.				
1	29762	29762	C	T	downstream		N;ORF10		dist=88.		.	1.54
3.11317		1	.	.	.	.	.	.	.	.	.	.
.	.	.	.	.	.	.	.	.				
1	29764	29764	G	A	downstream		N;ORF10		dist=90.		.	1.54
3.97972		1	.	.	.	.	.	.	.	.	.	.
.	.	.	.	.	.	.	.	.				

1 29766 29766 G C downstream N;ORF10 dist=92. . 0.628
2.40709 1 . . . .
. . . .
1 29773 29773 G T downstream N;ORF10 dist=99. . 0.631
2.50337 1 . . . .
. . . .
1 29779 29779 G T downstream N;ORF10 dist=105 . .
1.54 3.97972 1 . . . .
. . . .

1 29781 29781 G T downstream N;ORF10 dist=107 . .
1.54 3.97972 1 . . . .
. . . .

S3 Table: Summary of functional annotation of the genetic variants reported in the study.
