# Supplementary material for: High throughput detection and genetic epidemiology of SARS-CoV-2 using COVIDSeq next-generation sequencing
Source: PLoS One. 2021 Feb 17;16(2):e0247115. doi: 10.1371/journal.pone.0247115 (PMC7888613; doi:10.1371/journal.pone.0247115)
Supplement: S4 Table — (PDF) [file pone.0247115.s005.pdf]

Genomic Variant	Position	Ref	Alt	Gene	Variant_Type	Amino Acid Change	Reported functional Consequence
1059C>T	1059	C	T	ORF1ab	Single Nucleotide Variation	T265I	T265I mutation occurring in the nsp2 domain, which is predicted to modulate host cel survival and signalling incorporates a change of a polar amino acid (threonine) to a nonpolar one (isoleucine) thereby making it hydrophobic and induces structural alteration in that domain.
11083G>T	11083	G	T	ORF1ab	Single Nucleotide Variation	L3606F	Low abundance of L3606F mutation is considered surrogate to viral viablility, which is assumed to impact protein stability and host-pathogen interaction.
14408C>T	14408	C	T	ORF1ab	Single Nucleotide Variation	P314L	P314L mutation is found to result in significant alterations of Nsp12 domain
15324C>T	15324	C	T	ORF1ab	Single Nucleotide Variation	N619N	15324C>T mutation is found to be decrease the mutation rates of M and E genes
18877C>T	18877	C	T	ORF1ab	Single Nucleotide Variation	L1804L	18877C>T mutation is reported to be associated with increase in both genome-wide mutational load, as well as mutation density in M and E genes, which is an alternative indicator of mutational rate and virus evolution.
21575C>T	21575	C	T	S	Single Nucleotide Variation	L5F	L5F co-occurring with D614G were found to have significantly increased infectivity in comparison to the reference strain
21774C>T	21774	C	T	S	Single Nucleotide Variation	S71F	S71F is found to possess increased antigenicity using computational methods
22444C>T	22444	C	T	S	Single Nucleotide Variation	D294D	Mutation spanning diagnostic primer or probe site
22882T>G	22882	T	G	S	Single Nucleotide Variation	N440K	N440 mutants were found resistant to C135 mAb, but retained full sensitivity to both C121 and C144 antibodies.
22899G>T	22899	G	T	S	Single Nucleotide Variation	G446V	G446V was found to have significantly decreased infectivity in comparison to the reference strain. Also, naturally occurring mutations that conferred complete or partial resistance to C135 were at positions R346, N439, N440, K444, V445 and G446.
23277C>T	23277	C	T	S	Single Nucleotide Variation	T572I	T572I was predicted to stabilize the protein structure with the ΔΔG value of 0.296 kcal/mol.
23311G>T	23311	G	T	S	Single Nucleotide Variation	E583D	E583D was predicted to destabilize the protein structure with the ΔΔG value of −0.143kcal/mol.
23403A>G	23403	A	G	S	Single Nucleotide Variation	D614G	Variant found to be associated with higher fatality rate
23608G>T	23608	G	T	S	Single Nucleotide Variation	R682R	R682R mutation predicted computationally is found to possess reduced tropism and transmissibility
24368G>T	24368	G	T	S	Single Nucleotide Variation	D936Y	D936Y mutation is reported to have destabilizing effects on the fusion core of heptad repeat (HR1) owing to the direct contact of aromatic residues with the solvent. D936Y co-occurring with D614G is found to possess significantly increased infectivity in comparison to the reference

strain.
24378C>T 24378 C T S Single Nucleotide Variation S939F S939F mutation is reported to have destabilizing effects on the fusion core of heptad repeat (HR1) owing to the direct contact of aromatic residues with the solvent. S939F co-occurring with D614G is found to possess significantly increased infectivity in comparison to the reference strain.
24872G>T 24872 G T S Single Nucleotide Variation V1104L V1104L mutation has been found to be critical in the Ganglioside Binding Domain (GBD) of SARS-CoV-2 S involved in very hydrophobic recognition with Hydroxychloroquine.
25273G>C 25273 G C S Single Nucleotide Variation M1237I M1237I mutation co-occurring with D614G is reported to have decreased infectivity in comparison to the wildtype strain
25429G>T 25429 G T ORF3a Single Nucleotide Variation V13L V13L is a disease mutation with no changes in polarity and predicted impact in chemical properties of the protein
25552G>T 25552 G T ORF3a Single Nucleotide Variation A54S A54S mutation with a change in polarity is predicted to contribute to structural changes of ORF3a protein
25563G>T 25563 G T ORF3a Single Nucleotide Variation Q57H Q57H mutation co-occurring with D155Y has been suggested to have potential impact on inflammasome activation and also in decreasing the stability of the protein. Q57H was predicted to have DDG value of 0.12 kcal/mol
25785G>T 25785 G T ORF3a Single Nucleotide Variation W131C Mutation with a change in polarity from hydrophobic to hydrophilic is predicted contribute to structural changes of the ORF3a protein.
25819G>T 25819 G T ORF3a Single Nucleotide Variation A143S A143S mutation is reported to enhance the viral uptake by the host, thereby increasing the infectivity rate.
25855G>T 25855 G T ORF3a Single Nucleotide Variation D155Y D155Y mutation co-occurring with Q57H has been suggested to have potential impact on inflammasome activation and also in increased infectivity by facilitating the tetramerization process of SARS-CoV-2.
25904C>T 25904 C T ORF3a Single Nucleotide Variation S171L S171L mutation co-occurring with Q57H has been suggested to have potential impact on inflammasome activation.
25906G>T 25906 G T ORF3a Single Nucleotide Variation G172C G172C is found to decrease the stability of the protein. G172C was predicted to have DDG value of -0.83 kcal/mol
25947G>T 25947 G T ORF3a Single Nucleotide Variation Q185H Q185H mutation with predicted impact on ORF3a protein structure.
26062G>T 26062 G T ORF3a Single Nucleotide Variation G224C Mutation with a change in polarity from hydrophobic to hydrophilic is predicted contribute to structural changes of the ORF3a protein.

26152G>A	26152	G	A	ORF3a	Single Nucleotide Variation	G254R	This mutation was found to result in decreased intensity of specific epitopes
26204C>T	26204	C	T	ORF3a	Single Nucleotide Variation	T271I	This mutation was found to result in decreased intensity of specific epitopes
26447C>T	26447	C	T	E	Single Nucleotide Variation	S68F	S68F mutation was found to possess changes in R group side chain and potentially alter solvent accessibility through computational solvent accessibility prediction
26456C>T	26456	C	T	E	Single Nucleotide Variation	P71L	P71Lmutation was found to possess changes in R group side chain and potentially alter solvent accessibility through computational solvent accessibility prediction
27147G>T	27147	G	T	M	Single Nucleotide Variation	D209Y	This distinct nonsynonymous mutation was predicted to impact membrane protein structure
28077G>T	28077	G	T	ORF8	Single Nucleotide Variation	V62L	V62L substitution to have minor stabilization effects on ORF8 architecture.
28373G>T	28373	G	T	N	Single Nucleotide Variation	G34W	This variation was found have a potential to change both protein structure and solvent accessibility through computational analysis
28854C>T	28854	C	T	N	Single Nucleotide Variation	S194L	S194L was found to exhibit significant difference in protein structural morphologyin comparison with wildtype
28878G>A	28878	G	A	N	Single Nucleotide Variation	S202N	This mutation is found to increase the stability of the protein. S202N was predicted to have DDG value of -0.78 kcal/mol
28881G>A	28881	G	A	N	Single Nucleotide Variation	R203K	The triple site mutation 28881-28883 that brings change in two amino acid 203-204:RG>KR, is known to play a critical role in virion assembly and structure. The mutated 203/204 region of N protein is reported to affect the SR (serine-arginine)-rich motif of the protein (a crucial region for controlling viral transcription and replication)

S4 Table: Compilation of details of genetic variants with reported functional relevance
