# Supplementary material for: High throughput detection and genetic epidemiology of SARS-CoV-2 using COVIDSeq next-generation sequencing
Source: PLoS One. 2021 Feb 17;16(2):e0247115. doi: 10.1371/journal.pone.0247115 (PMC7888613; doi:10.1371/journal.pone.0247115)
Supplement: S5 Table — (PDF) [file pone.0247115.s006.pdf]

	COVIDSeq Positive	COVIDSeq Negative	Invalid	TOTAL
RT positive	633	16	6	655
RT Negative	6	13	0	19
Inconclusive	21	14	0	35
Pan Sarbeco	16	25	2	43
**TOTAL**	676	68	8	**752**

S5 Table: Summary of the COVIDSeq assay comparison with RT-PCR.
