# Supplementary material for: High throughput detection and genetic epidemiology of SARS-CoV-2 using COVIDSeq next-generation sequencing
Source: PLoS One. 2021 Feb 17;16(2):e0247115. doi: 10.1371/journal.pone.0247115 (PMC7888613; doi:10.1371/journal.pone.0247115)
Supplement: S6 Table — (PDF) [file pone.0247115.s007.pdf]

Isolation Method	RT-PCR Postive			RT-PCR Negative			pan-sarbeco			Inconclusive			Total
	COVIDSeq Detected	COVIDSeq Not Detected	COVIDSeq Invalid	COVIDSeq Detected	COVIDSeq Not Detected	COVIDSeq Invalid	COVIDSeq Detected	COVIDSeq Not Detected	COVIDSeq Invalid	COVIDSeq Detected	COVIDSeq Not Detected	COVIDSeq Invalid	
Nextractor	263	1	0	4	7	0	12	19	0	13	3	0	322
Trueprep	195	5	1	1	5	0	3	4	2	5	7	0	228
QIAamp	168	10	4	1	1	0	1	2	0	3	4	0	194
TRizol	7	0	1	0	0	0	0	0	0	0	0	0	8
Total	**633**	**16**	**6**	**6**	**13**	**0**	**16**	**25**	**2**	**21**	**14**	**0**	**752**

S6 Table : Comparison of different RNA extraction methods and detection of the SARS-CoV-2 with RT-PCR and COVIDSeq test.
